# Structural Reassignment of Covalent Organic Framework-Supported Palladium Species: Heterogenized Palladacycles as Efficient Catalysts for Sustainable C–H Activation

**DOI:** 10.1021/acscentsci.4c00660

**Published:** 2024-08-28

**Authors:** Meng-Ying Sun, Sheung Chit Cheung, Xue-Zhi Wang, Ji-Kang Jin, Jun Guo, Dan Li, Jian He

**Affiliations:** † Department of Chemistry, The University of Hong Kong, Pokfulam Road, Hong Kong 999077, P.R. China; ‡ State Key Laboratory of Synthetic Chemistry, The University of Hong Kong, Pokfulam Road, Hong Kong 999077, P.R. China; § College of Chemistry and Materials Science, and Guangdong Provincial Key Laboratory of Functional Supramolecular Coordination Materials and Applications, Jinan University, Guangzhou 510632, P.R. China

## Abstract

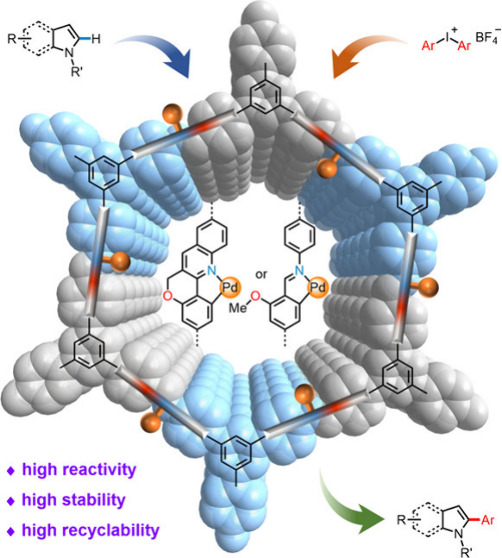

Recent decades have witnessed remarkable progress in ligand-promoted C–H activation with palladium catalysts. While a number of transformations have been achieved with a fairly broad substrate scope, the general requirements for high palladium loadings and enormous challenges in catalyst recycling severely limit the practical applications of C–H activation methodologies in organic synthesis. Herein, we incorporate N,C-ligand-chelated palladacycles into rigid, porous, and crystalline covalent organic frameworks for the C–H arylation of indole and pyrrole derivatives. These heterogeneous palladium catalysts exhibit superior stability and recyclability compared to their homogeneous counterparts. We not only produce several highly reactive palladacycles embedded on new framework supports to facilitate C–H activation/C–C bond-forming reactions but also reassign heterogenized palladium species on frameworks containing a benzaldehyde-derived imine moiety as imine-based palladacycles via comprehensive characterization. Our findings provide guidance for the rational design of framework-supported metallacycles in the development of heterogeneous transition-metal catalysis.

## Abstract 2

We incorporate N,C-ligand-chelated palladacycles into rigid, porous, and crystalline covalent organic frameworks for C−H activation/C−C bond-forming reactions with excellent catalyst recyclability.

## 1 Introduction

C–H bonds are ubiquitous in nature; the development of potent synthetic methods to directly functionalize such bonds is essential for bulk chemical production, drug discovery, and the exploration of novel functional materials.^1^ Due to its enriched redox manifolds and high compatibility with various coupling reagents, palladium redox catalysis is widely used to promote C–H activation/C–C and C–heteroatom bond formation.^2^ More importantly, different types of ligand scaffolds can be utilized to improve the efficiency and control the chemo-, regio-, and stereoselectivity of palladium-mediated C–H cleavage.^3^ Despite the vast array of transformations achieved via ligand-enabled approaches, palladium catalysts tend to deactivate rapidly in homogeneous catalytic systems, resulting in low turnover numbers (TONs < 10) for the majority of C–H activation reactions.^2,3^ In order to significantly increase the TONs of palladium catalysts for more practical synthetic applications, there are two major avenues to pursue: one is to improve catalyst reactivity through sophisticated ligand design;^4^ the other is to improve catalyst stability through the introduction of suitable catalyst supports.^5^ The latter strategy, which employs simple ligand scaffolds to produce effective and recyclable palladium catalysts, meets the requirements for developing sustainable C–H activation processes.

Since nitrogen-donor ligands, such as pyridine and quinoline derivatives, are highly compatible with Pd(II)/Pd(IV) catalytic cycles,^6^ they have been incorporated into polymer and micelle supports to demonstrate the viability of reusing Pd(II) and ligand.^7^ However, the recycled catalyst proved ineffective after several runs, presumably due to the high flexibility of polymer backbones and the relatively weak binding ability of monodentate ligands to Pd(II), making it difficult to prevent catalyst deactivation and palladium leaching. We envisioned that the construction of nitrogen-donor ligand motifs in the rigid linkers of porous crystalline covalent organic frameworks (COFs)^8^ and the subsequent introduction of a chelating metal coordination mode through postsynthetic cyclometalation would fundamentally address the aforementioned issues. Compared to metal–organic framework (MOF) materials,^9^ which are built through coordination interactions between organic linkers and metal nodes, COF supports typically exhibit superior chemical stability, including water stability. In addition, it is anticipated that other commonly used heterogeneous catalysts, such as palladium on charcoal, are unable to prevent palladium leaching due to a lack of suitable ligands to stabilize Pd(II). Therefore, we primarily focused on the modification of the COF materials to explore heterogeneous palladium-catalyzed C–H activation.

**Scheme 1:**
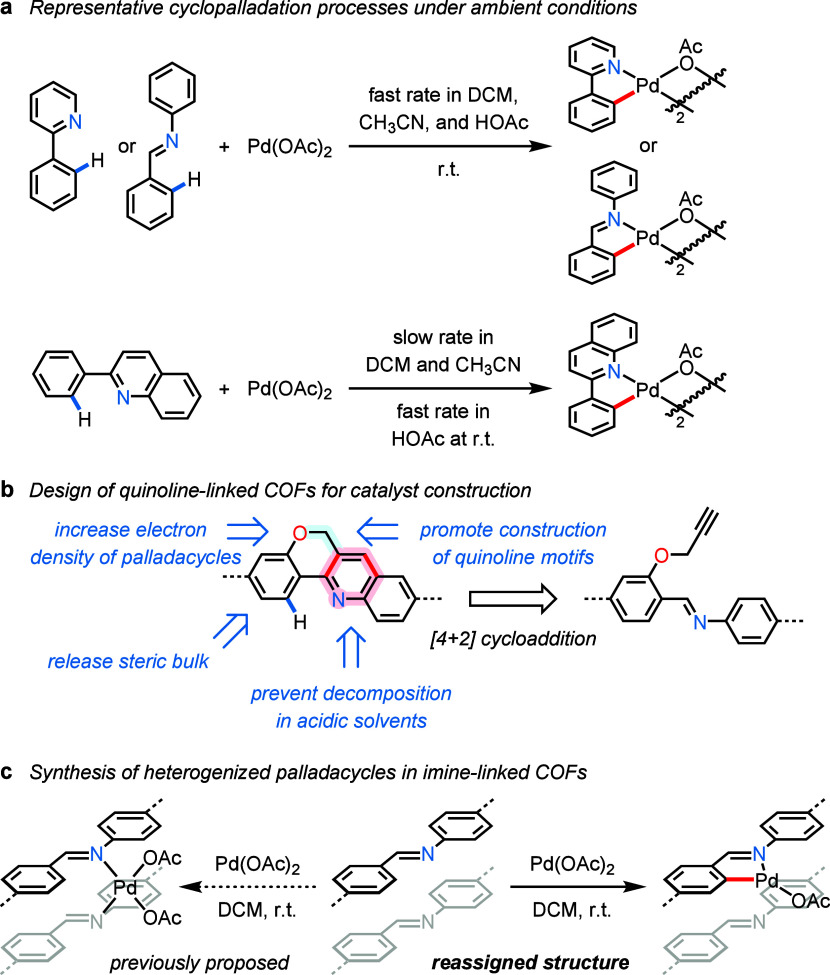
Development of Catalytically Active Palladacycles in COF Supports with Nitrogen-Based Linkages

Whereas cyclopalladation of 2-arylpyridines and benzaldehyde-derived imines proceeds rapidly in a variety of organic solvents, 2-arylquinolines react with Pd(OAc)_2_ at a significantly slower rate in many solvents other than acetic acid under ambient conditions (Scheme 1a; see Figures S1–S5).^10^ In this article, we present the synthesis of a new quinoline-linked COF material that is exceptionally stable in acidic solvents, via the intramolecular aza-[4 + 2] cycloaddition of a propargyloxy group with an imine linkage. The purposeful release of steric bulk from the aryl ring at the 2-position of the quinoline unit and the incorporation of an alkoxy functional group result in the successful formation of electron-rich palladacycles on the COF support (Scheme 1b). Notably, because Pd(II)-mediated C–H cleavage in benzaldehyde-derived imines is favored both kinetically and thermodynamically, the postsynthetic palladation of imine-linked COF materials produces supported imine-containing palladacycle catalysts (Scheme 1c), rather than heterogenized palladium species located in the COF interlayers, as supported by a series of characterization data. In addition to improving the stability of high-valent palladium intermediates and preventing the formation of inactive Pd(0) nanoparticles, the integration of palladacycles into different types of COF matrices provides high catalyst tunability for promoting the C–H arylation of heterocyclic substrates with diverse electronic properties.

## 2 Results and Discussion

**Figure 1:**
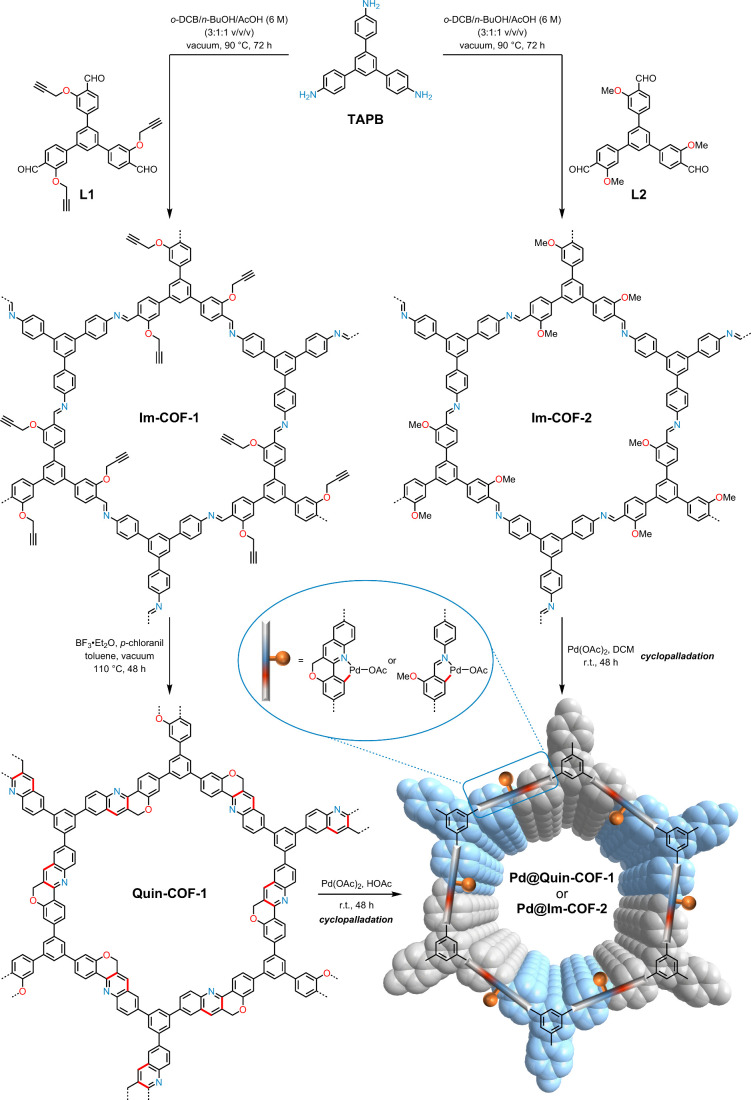
Schematic diagram for the synthesis of COF-supported cyclopalladated complexes with nitrogen-based ligands.

To build stable framework structures with sufficient pore sizes, we employed 1,3,5-tris(4-aminophenyl)benzene (**TAPB**) and the *C*_3_-symmetric trisaldehyde monomers derived from 1,3,5-tris(4-formylphenyl)benzene (**L1** and **L2**) in solvothermal synthesis (Figure 1).^11^ With three propargyloxy groups introduced to the 3-positions of the 4-formylphenyl groups, the imine-linked COF material (**Im-COF-1**) was successfully prepared for the further construction of quinoline moieties to react with Pd(II). Using BF_3_·Et_2_O as the Lewis-acid promoter and *p*-chloranil as the external oxidant,^12^**Im-COF-1** was efficiently converted into the targeted quinoline-linked COF support (**Quin-COF-1**) via an intramolecular aza-[4 + 2] cycloaddition between the aromatic imine and the terminal alkyne.^13,14^ On account of the strong resonance effect of the anisole oxygen atom, methoxy groups were incorporated into **Im-COF-2** to prepare a highly electron-rich imine-based palladium catalyst. The cyclometalation of **Quin-COF-1** and **Im-COF-2** with Pd(OAc)_2_ in acetic acid and dichloromethane, respectively, gave COF-supported palladacycles **Pd@Quin-COF-1** and **Pd@Im-COF-2** at room temperature (Figure 1).^15^

The chemical compositions of the resulting COFs were investigated by using extensive spectroscopic measurements. As shown in Figures S12 and S13, the simultaneous attenuation of the characteristic N–H stretching bands of **TAPB** (at 3434 and 3354 cm^–1^) and the C=O stretching vibrations of aldehydes around 1677 cm^–1^ in the FT-IR spectra indicates the effective condensation between amino and formyl groups during the formation of **Im-COF-1** and **Im-COF-2**.^16^ The remaining carbonyl signals, which are prevalent in other imine-linked COF materials,^14a,15a,17^ could be attributed to a small fraction of unreacted aldehydes on the COF surface.^17a^ Notably, the successful conversion of **Im-COF-1** to **Quin-COF-1** can be confirmed by observing the disappearance of the stretching vibration modes of the propargyl group (3297 cm^–1^ for the ≡C—H stretch and 2132 cm^–1^ for the C≡C stretch).^12a,14a^ X-ray photoelectron spectroscopy (XPS) data also support the formation of the quinoline linkage (Figure S15): following intramolecular cycloaddition, the N 1s peak at 398.7 eV attributed to imine nitrogen atoms shifts to a higher binding-energy region (around 401.0 eV). The degree of quinoline formation is estimated to be 77% based on the XPS peak area.^18^

In addition, the solid-state ^13^C NMR spectra of **Im-COF-1** and **Im-COF-2** suggest that both COF materials contain alkoxy groups (Figures S17 and S19). While the alkynyl moiety is responsible for the carbon signals around 78 ppm, the sp^3^-hybridized carbon centers adjacent to oxygen give chemical shifts of 56 and 54 ppm, respectively. Upon the cycloaddition/oxidation processes, the carbon signals of the propargyl group were weakened significantly (Figure S18), indicating its high conversion to the quinoline moiety in **Quin-COF-1**.

**Figure 2:**
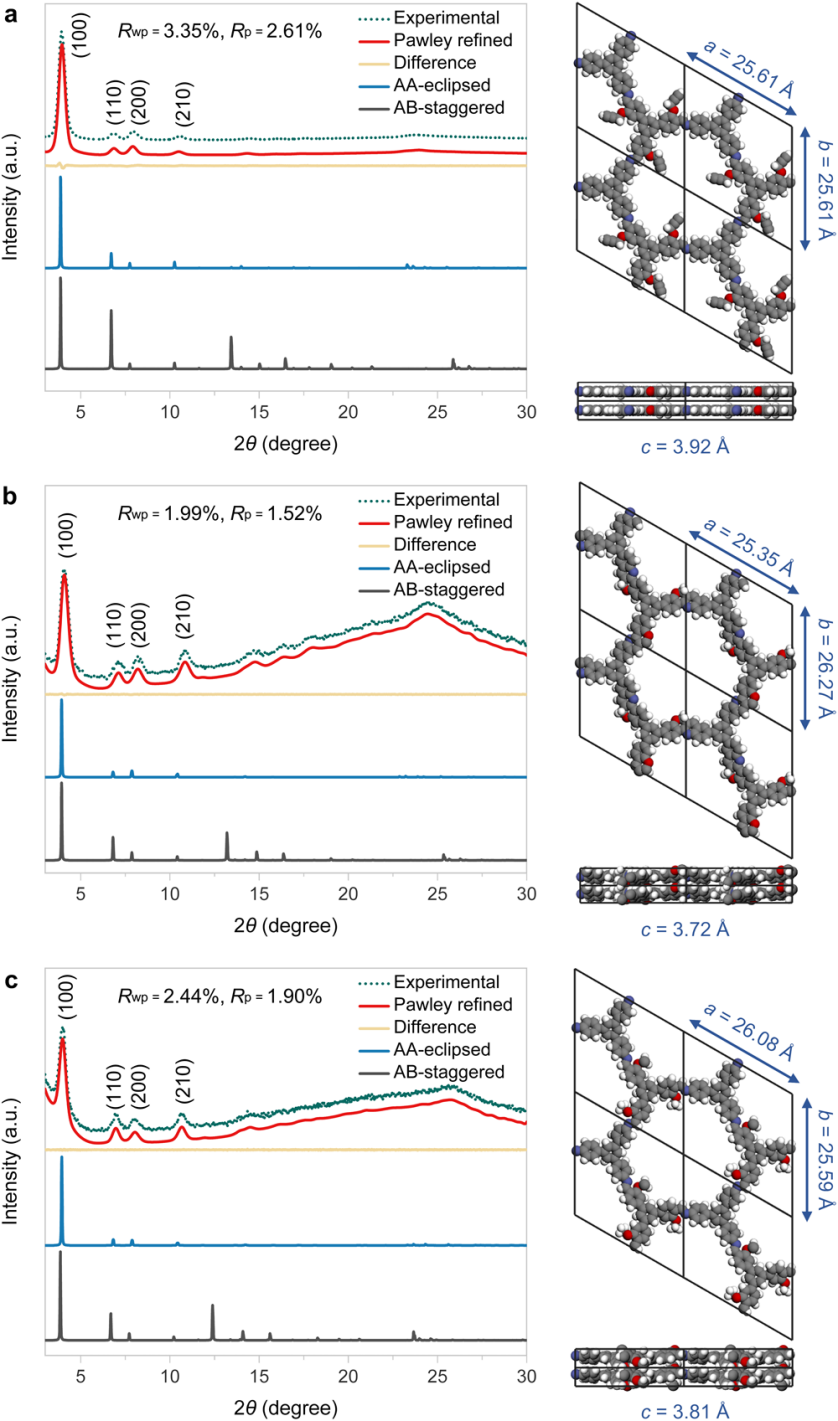
PXRD patterns and refined modeling profiles of **Im-COF-1** (a), **Quin-COF-1** (b), and **Im-COF-2** (c). Color labels: C, gray; N, blue; O, red; and H, white.

The crystallinity of these newly synthesized COF materials was examined by powder X-ray diffraction (PXRD). As depicted in Figure 2, the PXRD patterns feature a prominent peak at 3.92° and three relatively weak peaks between 6° and 11°, which correspond to the (100), (110), (200), and (210) reflection planes, respectively.^11b^ The PXRD data revealed that the imine- and quinoline-linked COFs both had good crystallinity and reasonably large crystal grain sizes, with narrow full width at half-maximum values for the sharp (100) peaks. Notably, there are no obvious peak shifts from **Im-COF-1** to **Quin-COF-1** (Figure 2a,b), implying that the highly ordered framework structures are preserved during the intramolecular cycloaddition. In all three cases, the Pawley-refined PXRD profiles based on the AA-eclipsed mode match well with their experimentally observed profiles, as evidenced by the negligible signals in difference curves, with *R*_wp_ and *R*_p_ values converged to 3.35% and 2.61% for **Im-COF-1**, 1.99% and 1.52% for **Quin-COF-1**, and 2.44% and 1.90% for **Imine-COF-2**, respectively. In terms of optimized unit cell parameters after Pawley refinement, the *a*-axis of **Quin-COF-1** is found to be smaller than that of **Im-COF-1**, which may be due to the elimination of propargyloxy side chains and an increase in the overall rigidity.^14a^

**Figure 3:**
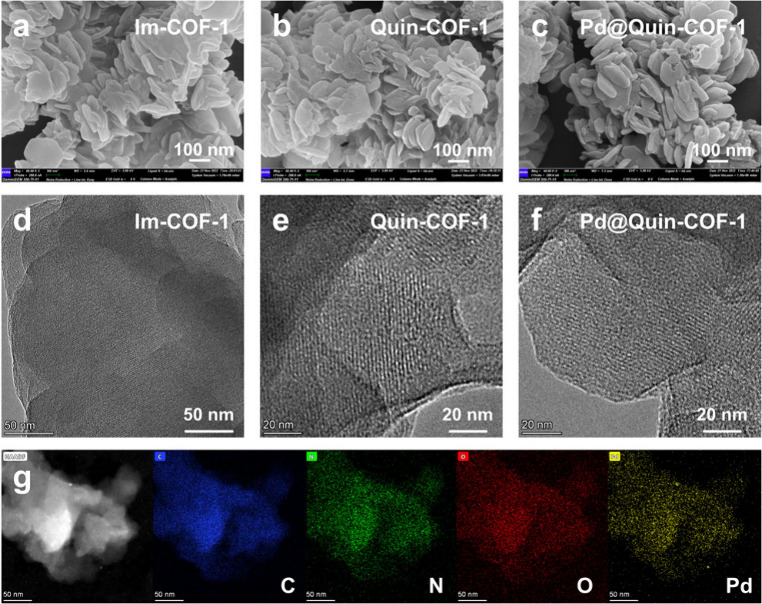
SEM and TEM images of **Im-COF-1** (a, d), **Quin-COF-1** (b, e), and **Pd@Quin-COF-1** (c, f). (g) Elemental mapping of **Pd@Quin-COF-1**. Scale bar: 50 nm. EDS mapping images of C, N, O, and Pd are colored blue, green, red, and yellow, respectively.

To visualize the periodic structures of the COF materials, we utilized scanning electron microscopy (SEM) and transmission electron microscopy (TEM) for further characterization. **Im-COF-1**, **Quin-COF-1**, and **Im-COF-2** all have a stacked layered-sheet-like morphology, as seen in SEM images (Figures 3a,b, S20, and S21). The identification of lattice fringes in TEM images demonstrates their high crystallinity and microporous structures (Figure 3d,e and S25). After testing the stability of the framework supports in various solvents (Figures S27–S32), we optimized the conditions for postsynthetic metalation in order to prepare the COF-supported palladium catalysts and discovered that acetic acid and dichloromethane are the optimal solvents for the cyclopalladation of the quinoline- and imine-linked COFs, respectively, which are analogous to the conditions used to synthesize the corresponding cyclopalladated complexes (**Quin-Palladacycle** and **Im-Palladacycle**, see the Supporting Information for details). Given that each palladacycle unit contains a nitrogen- and carbon-based bidentate ligand and a Pd(II) center, the theoretical Pd/N ratios in both COF materials after cyclopalladation should be 1:1. However, because of the obvious steric bulk of 2-arylquinoline moieties, the Pd/N ratio in **Pd@Quin-COF-1** was determined to be 1:13 (Table S1, entry 1), while this ratio was substantially higher in **Pd@Im-COF-2** (1:4.1; Table S1, entry 3). According to the SEM and TEM images of **Pd@Quin-COF-1** and **Pd@Im-COF-2** (Figures 3c,f, S21, and S26), the morphology of the COF supports remains nearly unchanged upon treatment with Pd(OAc)_2_ solutions. Energy dispersive spectroscopy (EDS) mapping revealed uniform distributions of the elements C, N, O, and Pd throughout the entire frameworks, confirming the uniform compositions of the COF-supported palladacycle catalysts (Figures 3g and S36).

**Figure 4:**
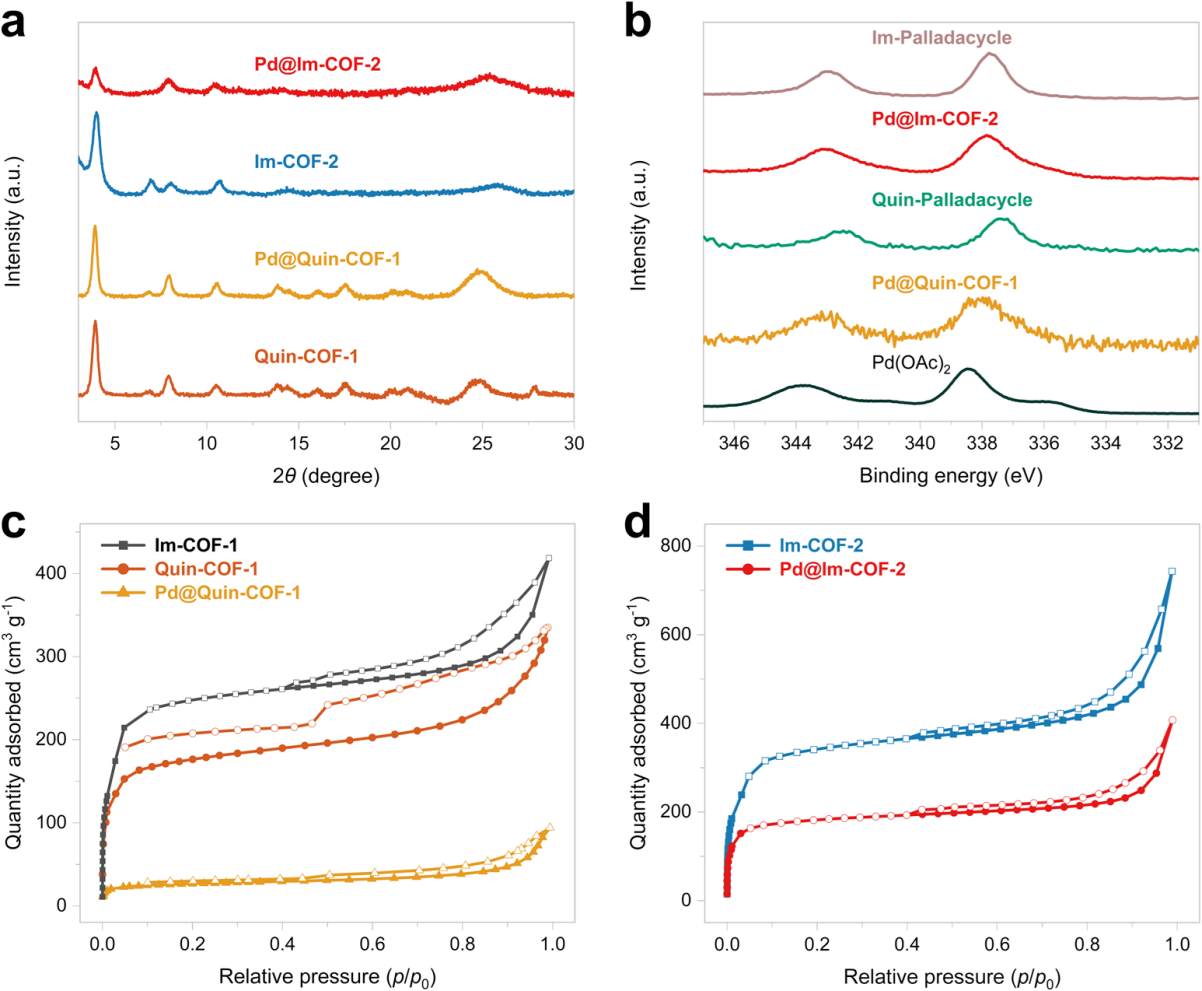
(a) PXRD patterns before and after postsynthetic metalation. (b) Pd 3d XPS spectra of Pd(OAc)_2_, **Pd@Quin-COF-1**, **Quin-Palladacycle**, **Pd@Im-COF-2**, and **Im-Palladacycle**. (c) N_2_ adsorption/desorption isotherms of **Im-COF-1**, **Quin-COF-1**, and **Pd@Quin-COF-1** collected at 77 K. (d) N_2_ adsorption/desorption isotherms of **Im-COF-2** and **Pd@Im-COF-2** collected at 77 K.

The PXRD patterns of **Pd@Quin-COF-1** and **Pd@Im-COF-2** are almost identical to those of **Quin-COF-1** and **Im-COF-2** (Figure 4a), which indicates that the COFs retained their structural integrity after reacting with Pd(OAc)_2_. Additionally, thermogravimetric analysis traces and UV–vis spectra revealed no discernible difference before and after palladium incorporation (Figures S37–S40). The high-resolution Pd 3d XPS spectra showed that the palladium centers in the COF-supported catalysts are in a +2 oxidation state (Figure 4b).^15a,19^ Compared to Pd(OAc)_2_, the decrease in binding energy reveals that the Pd(II) centers in both the COF-supported palladacycles and the cyclopalladated complexes become more electron-rich.^14c^ Because imine moieties are a stronger nitrogen-based electron donor than quinoline moieties in COF materials (Figure S15), **Pd@Im-COF-2** displays a Pd 3d_3/2_ peak at 343.03 eV and a Pd 3d_5/2_ peak at 337.83 eV, both of which are in lower regions than the corresponding peaks for **Pd@Quin-COF-1**. The XPS data strongly support the palladacycle formation in the case of **Im-COF-2**, which is consistent with the computational studies (see the Supporting Information for details). The changes in COF porosity following postsynthetic metalation were demonstrated by gas adsorption/desorption measurements conducted at 77 K (Figure 4c,d). Based on the Brunauer–Emmett–Teller model, the surface areas of **Im-COF-1**, **Quin-COF-1**, **Pd@Quin-COF-1**, **Im-COF-2**, and **Pd@Im-COF-2** were calculated to be 973.9, 565.9, 99.6, 1318.1, and 705.3 m^2^ g^–1^, respectively. The reduced N_2_ adsorption and surface areas of **Pd@Quin-COF-1** and **Pd@Im-COF-2** strongly support the successful incorporation of palladium species.^20^ Compared with **Im-COF-2**, the introduction of bulkier propargyloxy groups to the COF linkers decreased the surface area of **Im-COF-1**. To investigate their catalytic activity in C–H arylation reactions, we determined the palladium loadings in **Pd@Quin-COF-1** and **Pd@Im-COF-2** using inductively coupled plasma atomic emission spectroscopy (ICP-AES) (see the Supporting Information for details).

**Figure 5:**
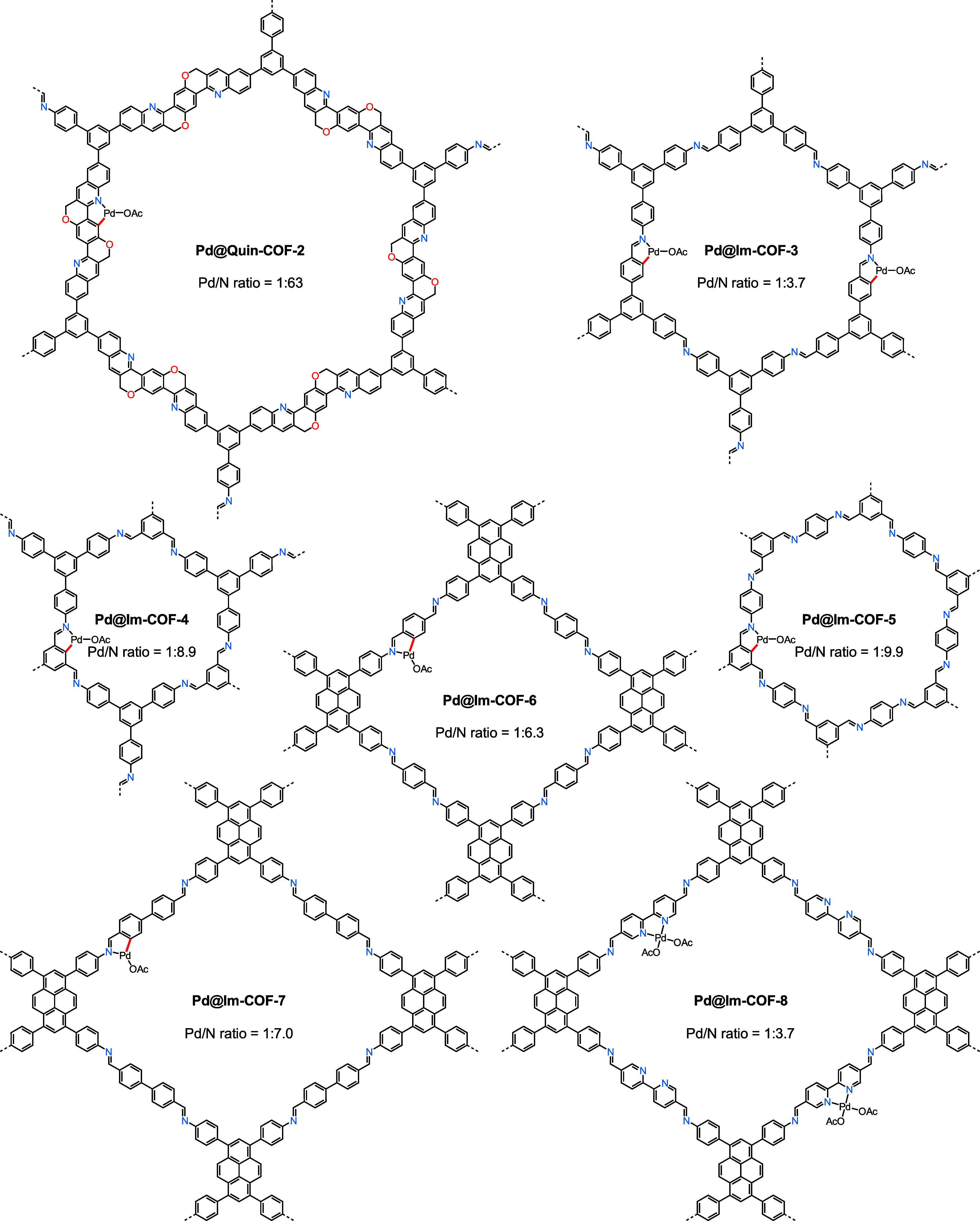
Structure of different Pd(II)-incorporated COF materials.

To further illustrate the steric and electronic effects of nitrogen-based COF linkers on cyclopalladation, we prepared several previously reported COF materials and subjected them to the established postsynthetic metalation conditions (Figure 5). When the quinoline-based framework (**Quin-COF-2**)^14a^ synthesized from **TAPB** and 2,5-bis(propargyloxy)terephthalaldehyde was utilized as the solid matrix, the cyclopalladation process turned to be very sluggish, giving a Pd/N ratio of only 1:63 in an acetic acid solution. The scenario is well explained by slow C–H cleavage at the 2-position of the aryl ring when an adjacent substituent is present. This steric effect would not be as pronounced if the metalation step was simply a palladium adsorption in the interlayers of **Quin-COF-2**. After methoxyl groups were removed from **Im-COF-2**, the corresponding imine-linked COF (**Im-COF-3**) showed a similar palladium incorporation efficiency, indicating that the electronic effect on imine-directed C–H cleavage is somewhat negligible. The substitution of 1,3,5-tris(4-formylphenyl)benzene for benzene-1,3,5-tricarbaldehyde^21^ resulted in much lower Pd/N ratios in **Pd@Im-COF-4** and **Pd@Im-COF-5**, most likely due to increased steric bulk of the aryl rings for cyclopalladation. The release of steric influence by employing a pyrene-based tetraaniline^8d,22^ and aromatic dialdehydes (e.g., terephthalaldehyde and [1,1′-biphenyl]-4,4′-dicarbaldehyde) in the construction of **Im-COF-6** and **Im-COF-7** provided moderate cyclometalation efficiencies of approximately 15%. Because of a strong coordination ability of bipyridines and the difficulties in activating pyridine C–H bonds (Figure S49), postsynthetic palladation of imine-linked COF material **Im-COF-8** derived from [2,2′-bipyridine]-5,5′-diamine efficiently produced a heterogenized bipyridine-ligated Pd(II) complex without a possible palladacycle formation.

With a series of COF-supported palladium catalysts in hand, we began to test our hypothesis using the C–H arylation of *N*-methyl indole (**1a**) with diphenyliodonium tetrafluoroborate (**2a**) as a template reaction.^23^ While the corresponding homogeneous palladium catalysis could be promoted through the use of a *N*-heterocyclic carbene ligand, catalyst recycling remains quite difficult to achieve, even with diverse solid matrices. As a result, it is vital to employ the novel heterogenized palladacycles to explore sustainable C–H arylation with high catalyst recyclability. After a systematic survey of various reaction parameters (Tables S5–S7), we were delighted to discover that *N*-methyl 2-phenylindole (**3a**) could be obtained in 97% yield with only 2 mol % **Pd@Quin-COF-1** (Table 1, entry 1). On the contrary, **Pd@Quin-COF-2** exhibited drastically poor reactivity after the first run of catalysis (Table 1, entry 2). Given that the crystallinity of **Quin-COF-2**, which had a large pore around 2.8 nm,^14a^ was completely lost during the postsynthetic metalation step (Figure S53), the COF support could no longer stabilize palladacycle intermediates in the arylation reaction. This control experiment unequivocally demonstrates the importance of using novel quinoline-linked COFs with excellent structural stability in the development of robust catalysts for C–H activation. When the framework support was replaced with imine-linked ones, the yield of **3a** decreased in the heterogeneous palladium catalysis (entries 3–8). The incorporation of methoxy groups in the imine-linked COF synthesis is beneficial to the palladium-catalyzed C–H arylation (entries 3 and 4). The COF-supported palladacycles with increased steric hindrance at the 3-position of the aryl ring resulted in a lower product yield (entries 5 and 6). Upon further enlargement of imine-linked COF pores with 1,3,6,8-tetrakis(4-aminophenyl)pyrene monomer, the catalytic activity of immobilized palladium catalysts decreased dramatically in the second cycles (entries 7–9). The inferior performance of **Pd@Im-COF-6**, **Pd@Im-COF-7**, and **Pd@Im-COF-8** is attributed to the instability of the imine-linked framework supports under the standard reaction conditions (Figure S54). In the recycling experiments, all of these heterogeneous palladium catalysts with large COF pores demonstrated a reactivity trend similar to that of **Pd@Quin-COF-2** (entry 2). Notably, the strong binding of bipyridine ligands to Pd(II) on a fragile COF support is insufficient to prevent the deactivation of the heterogeneous catalyst in the C–H arylation (entry 9), indicating the need for a new framework design. Moreover, the introduction of Pd(OAc)_2_ into MOF supports^24^ failed to maintain its reactivity after catalysis (entries 10 and 11). Significantly, although commercially available palladium on carbon initially showed some catalytic activity in the template reaction, it was incapable of driving product formation in the second run (entry 12). It is noteworthy that the corresponding homogeneous catalytic systems proved unrecyclable, and none of the recovered palladium species gave any desired products (entries 13–17). In the first run of the palladium-catalyzed C–H arylation of **1a**, the cyclopalladated complexes outperformed the homogeneous Pd(OAc)_2_ catalysts with and without bidentate nitrogen-based ligands.

**Table 1:**
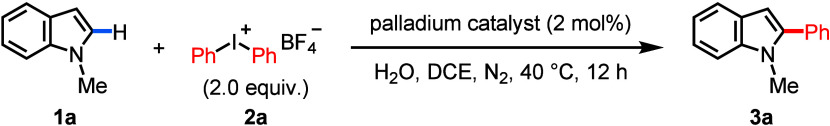
Effects of Different Catalytic Systems on the C–H Arylation of **1a**^a^

entry	catalyst	1st run yield (%)	2nd run yield (%)
1	**Pd@Quin-COF-1**	97	97
2	**Pd@Quin-COF-2**	55	5
3	**Pd@Im-COF-2**	82	77
4	**Pd@Im-COF-3**	76	70
5	**Pd@Im-COF-4**	68	46
6	**Pd@Im-COF-5**	62	38
7	**Pd@Im-COF-6**	26	10
8	**Pd@Im-COF-7**	10	<1
9	**Pd@Im-COF-8**	92	3
10	**Pd@UiO-67-bpy**	39	<1
11	**Pd@MOF-253-bpy**	3	<1
12	Pd/C	36	<1
13	Pd(OAc)_2_	32	<1
14^b^	Pd(OAc)_2_	10	<1
15^c^	Pd(OAc)_2_	13	<1
16	**Quin-Palladacycle**	88	<1
17	**Im-Palladacycle**	74	<1

a Reaction conditions: *N*-methyl indole **1a** (0.1 mmol, 1 equiv), diphenyliodonium salt **2a** (2.0 equiv), palladium catalyst (2 mol %), and H_2_O (50 μL) in anhydrous DCE (1 mL) under nitrogen atmosphere at 40 °C for 12 h. Yield was determined by ^1^H NMR of the crude product using dibromomethane as an internal standard.

b 2,2′-Bipyridine (2 mol %) was added as a ligand.

c 1,10-Phenanthroline (2 mol %) was added as a ligand.

After establishing robust heterogeneous palladium catalysis for C–H arylation, we examined the substrate scope for both substituted *N*-methyl indoles and diaryliodonium tetrafluoroborates (Table 2). A wide range of electron-donating and electron-withdrawing functional groups, including a Weinreb amide (**3g**) and halogen atoms with the exception of an iodo group (**3h**–**3j**), are well tolerated at various positions of indole rings. Despite increased steric hindrance during C–H cleavage, the C2-selective C–H arylation proceeded smoothly upon the introduction of a methyl substituent at the 3-position (**3k**). When the 2-position was blocked, the 3-position of the indole derivative underwent selective C–H arylation instead (**3l**). Other diaryliodonium coupling partners with distinct steric and electronic properties all exhibited excellent reactivity, providing the arylated products in yields of 74–96% (**3m**–**3t**). Moreover, the heterogeneous palladium catalysis can be applied to a 2 mmol synthesis of **3a** in a high yield (see the Supporting Information for details).

**Table 2:**
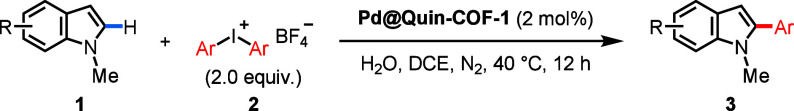

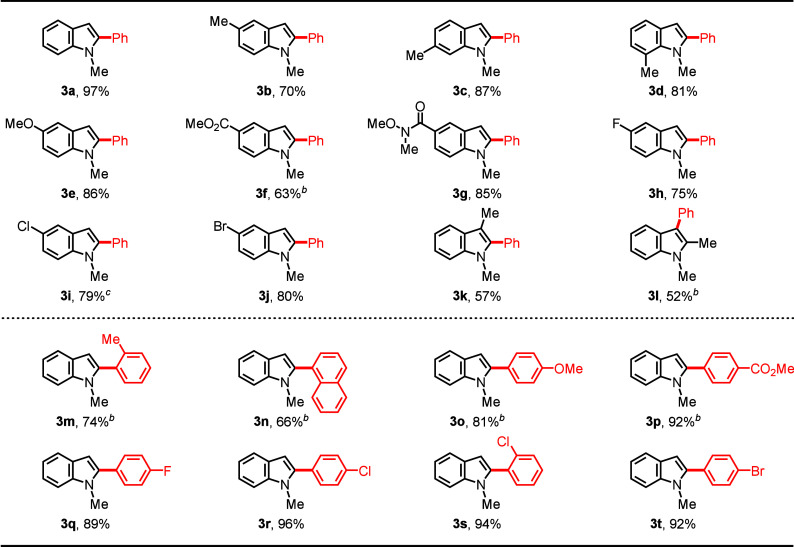
C2-Selective C–H Arylation of *N*-Methyl Indoles Using COF-Based Heterogeneous Palladium Catalysts^a^

a Reaction conditions: *N*-methylindole **1** (0.1 mmol, 1.0 equiv), diaryliodonium salt **2** (2.0 equiv), **Pd@Quin-COF-1** (2 mol %), and H_2_O (50 μL) in anhydrous DCE (1 mL) under nitrogen atmosphere at 40 °C for 12 h. Data are reported as isolated yields.

b 24 h.

c **Pd@Im-COF-2** (5 mol %), 24 h.

While **Pd@Quin-COF-1** outperformed the majority of COF-supported palladium catalysts for the C–H arylation of free indoles (Table S9), which are less electron-rich than *N*-methyl indoles, **Pd@Im-COF-2** demonstrated the best catalytic performance due to **Im-COF-2**’s electron-donating nature (Figure 4b). Upon slight modification of water loadings (Table S10), a variety of the indole substrates could be arylated in good yields (Table 3). In contrast to homogeneous catalytic systems, the arylation reaction is more susceptible to steric hindrance at the 3-position of indoles (**5h**). When the methyl group was replaced with a larger substituent in *N*-acetyl-tryptophan methyl ester, the heterogeneous palladium catalysis became less effective, revealing size selectivity in COF-based reaction systems (Table S11). In addition, when excess pyrroles were present, the monoarylation proceeded with high chemoselectivity (**7a** and **7b**). 2-Arylpyrrole derivative **7b** also serves as a suitable substrate, furnishing the heterodiarylated products with high TONs (**7c** and **7d**).

**Table 3:**
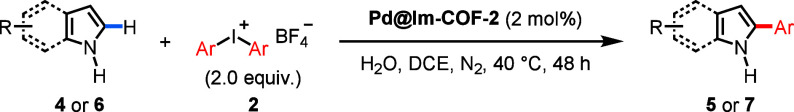

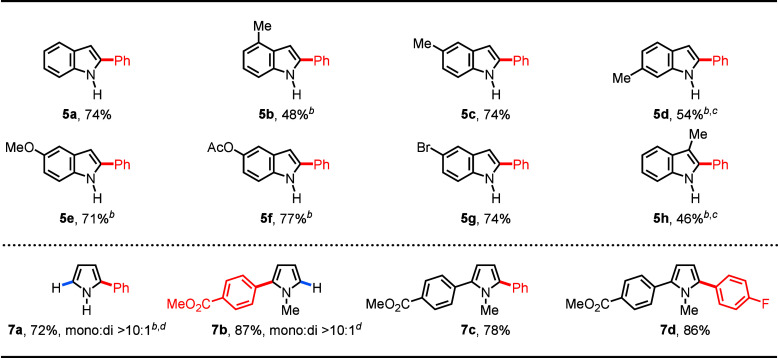
C2-Selective C–H Arylation of Indoles and Pyrroles Using COF-Based Heterogeneous Palladium Catalysts^a^

a Reaction conditions: indole **4** or pyrrole **6** (0.1 mmol, 1.0 equiv), diaryliodonium salt **2** (2.0 equiv), **Pd@Im-COF-2** (2 mol %), and H_2_O (75 μL) in anhydrous DCE (1 mL) under nitrogen atmosphere at 40 °C for 48 h. Data are reported as isolated yields.

b **Pd@Im-COF-2** (5 mol %).

c H_2_O (100 μL).

d Pyrrole **6** (1.0 mmol, 10 equiv) and diaryliodonium salt **2** (0.1 mmol, 1.0 equiv). Ratios of mono- and diarylated products were determined by ^1^H NMR analysis of the crude reaction mixture.

**Figure 6:**
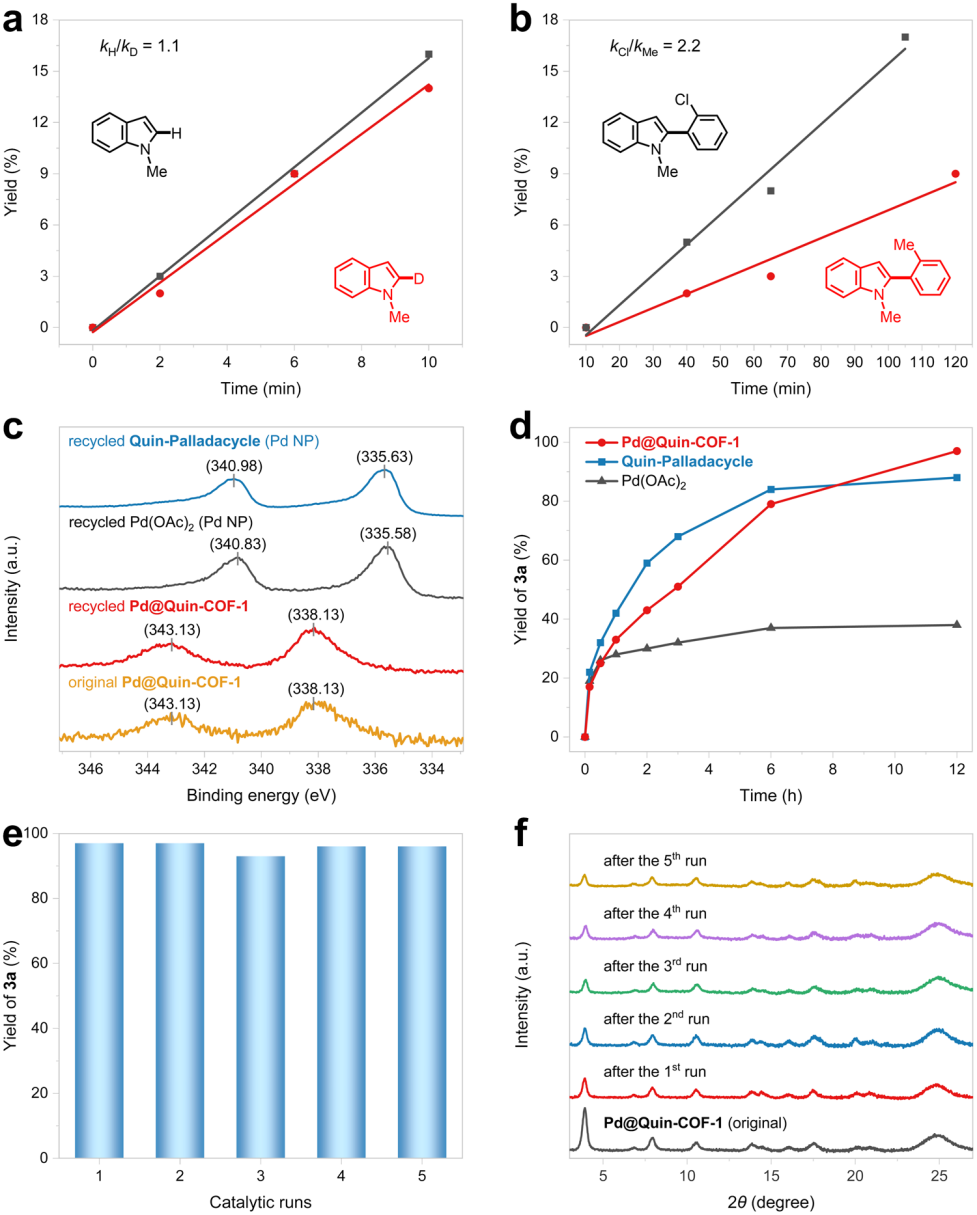
(a) KIE of the heterogeneous palladium-catalyzed C–H arylation. (b) Effects of diaryliodonium salts on the reaction rate. (c) Pd 3d XPS spectra of **Pd@Quin-COF-1** after 5 catalytic runs (red line) and recovered palladium species after the first run of homogeneous catalysis (black and blue lines), revealing that the Pd centers are in the +2 and 0 oxidation states, respectively. (d) Reaction profiles with the addition of **Pd@Quin-COF-1** (2 mol %), **Quin-Palladacycle** (2 mol %), and Pd(OAc)_2_ (2 mol %), respectively. (e) Recycling experiments for the synthesis of **3a**. (f) PXRD patterns of **Pd@Quin-COF-1** before and after catalysis.

**Scheme 2:**
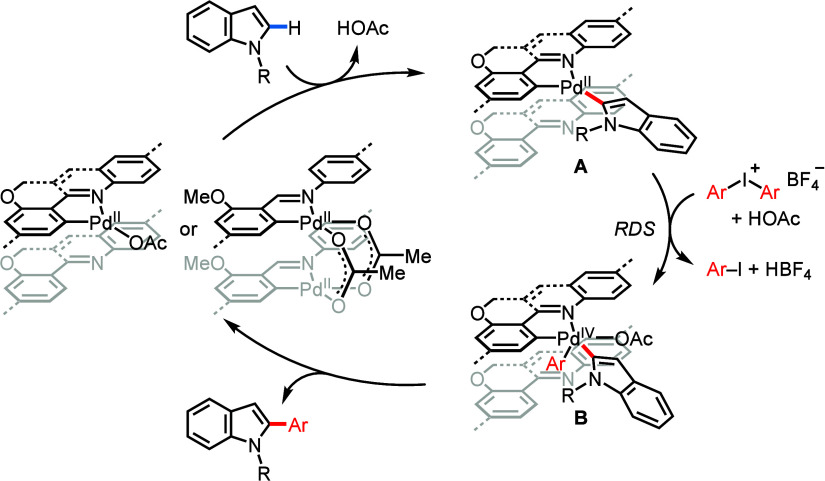
Plausible Mechanism for C–H Arylation via COF-Supported Palladacycle Intermediates

To gain in-depth mechanistic insights into the C–H arylation with **Pd@Quin-COF-1**, we studied the intermolecular kinetic isotope effect (KIE) of **1a** and **1a**-*d* by comparing their initial rates in the parallel reactions with **2a** (Figure 6a). The observed *k*_H_/*k*_D_ value of 1.1 suggests that the C–H cleavage of a *N*-methyl indole by a quinoline-containing cyclopallated complex is not the rate-determining step (RDS) in the heterogeneous palladium catalysis (Scheme 2). The same KIE was also observed in the arylation catalyzed by **Pd@Im-COF-2** (Figure S61), indicating that a monomeric or dimeric palladacycle embedded on **Im-COF-2** can rapidly break the indole C–H bond.^25^ A bimetallic reaction pathway is unlikely in the catalytic system of **Pd@Quin-COF-1** due to a low Pd/N ratio. Since the reaction rate with electron-deficient coupling partners is noticeably higher than that with electron-rich coupling partners (e.g., *k*_Cl_/*k*_Me_ = 2.2; Figure 6b), the oxidative addition of aryl–palladium intermediate **A** with diaryliodonium salts to generate highly reactive Pd(IV)^26^ intermediate **B** is most likely the RDS (Scheme 2). In order to provide electron-rich Ar–Pd^II^–L for promoting the oxidative addition step, a more electron-donating LX-type ligand is preferred when the aryl group is derived from free indoles as opposed to *N*-methyl indoles. Therefore, **Pd@Im-COF-2**, with 3-methoxy-2-((arylimino)methyl)phenyl moieties attached to Pd(II), becomes the optimal COF-based palladium catalyst for the C–H arylation of free indoles. After reductive elimination to produce the arylated heterocycles, the active Pd(II) catalyst is regenerated, as confirmed by XPS analysis of the recycled **Pd@Quin-COF-1** (Figure 6c). In sharp contrast to the homogeneous catalytic systems, no Pd(0) species, such as palladium black or palladium nanoparticles, were produced in the arylation reactions. Without stabilization on a rigid COF support, soluble Pd(OAc)_2_ and **Quin-Palladacycle** would decompose into Pd(0) nanoparticles, which was confirmed by both XPS spectra and PXRD patterns (Figures 6c and S62). Similar scenarios were also observed when Pd(II) catalytic centers were incorporated into MOF materials.^27^

**Scheme 3:**
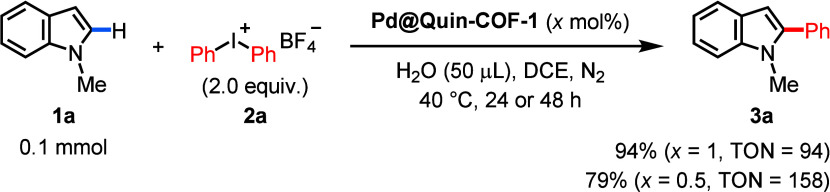
Highly Efficient Heterogeneous Palladium Catalysis for C–H Arylation

According to a comprehensive analysis of the reaction profiles (Figure 6d), the initial rates for the C–H arylation of **1a** were determined in both heterogeneous and homogeneous catalytic systems. **Quin-Palladacycle** with a strong ligand chelation effect achieved the highest rate in the first 2 h, revealing its remarkable catalytic performance in the nondirected C–H activation by stabilizing the critical Pd(IV) intermediate and suppressing the generation of inactive Pd(0) species.^28^ The reaction with Pd(OAc)_2_ slowed down considerably after 1 h, and the catalyst completely lost its activity after being subjected to the arylation conditions for 3 h, owing to the inevitable and rapid formation of palladium nanoparticles (Figure 6c). A similar deactivation scenario was also observed in the previous homogeneous catalysis (Figure S52), which utilized acetic acid as an optimal solvent to achieve a relatively higher initial rate.^23^ On the other hand, **Pd@Quin-COF-1** maintained high catalytic activity throughout the entire reaction process. Importantly, no palladium leaching was detected through ICP-AES measurements of the reaction mixture and the recycled catalyst (Table S14). The catalytic activity of the COF-supported palladacycle remained constant after a 5 run recycling experiment, and the sharp reflection peaks were retained in the PXRD patterns, albeit with a minor decrease in intensity (Figure 6e,f). Nevertheless, the COF support displayed nearly identical pores after the first run of heterogeneous catalysis (Figures S63 and S64). To further illustrate the effectiveness of heterogeneous palladium-catalyzed C–H arylation, we carried out the template reaction with 0.5–1 mol % **Pd@Quin-COF-1** and obtained TONs as high as 158 (Scheme 3). The exceptionally low palladium catalyst loadings underline the importance of the heterogenization strategy for the future development of C–H activation reactions. To demonstrate its broad applicability, we employed **Pd@Quin-COF-1** to achieve C–H bromination and acetoxylation of 2-phenylpyridine in synthetically useful yields (see the Supporting Information for details), showcasing the potentials of COF-supported palladacycles in catalyzing directed C–H activation/C–heteroatom bond-forming reactions. The PXRD patterns were well maintained after the catalytic reactions (Figure S70).

## 3 Conclusions

We report the synthesis and characterization of new quinoline- and imine-linked COF materials that can be used as suitable solid supports to prepare robust heterogeneous palladium catalysts with high stability and reactivity. In addition to producing a catalytically active palladacycle with a 2-arylquinoline moiety on the COF, we revealed that in situ cyclopalladation rather than direct Pd(II) adsorption dominates the postsynthetic metalation of many other imine-linked COFs. Significantly, the palladacycle species have been employed for the first time to develop C–H activation in heterogeneous catalytic systems. In accordance with Pd(II)/Pd(IV) redox manifolds, the immobilized cyclopalladated complex with a quinoline ligand promotes the C2-selective C–H arylation of *N*-methylindoles, whereas the COF-supported electron-rich imine-containing palladacycle exhibits exceptional reactivity in the transformations of free indoles and pyrroles. The recovered heterogeneous palladium catalysts can be reused for multiple cycles with identical activity. Our current research offers alternative and promising strategies to establish practical and sustainable C–H activation processes for green chemical synthesis.

## Supporting Information Available

Experimental details for the preparation and characterization of COFs, catalysis, and additional data (PDF)
Transparent Peer Review report available (PDF)
