# Supplementary material for: Structural Reassignment of Covalent Organic Framework-Supported Palladium Species: Heterogenized Palladacycles as Efficient Catalysts for Sustainable C–H Activation
Source: ACS Cent Sci. 2024 Aug 28;10(10):1848–60. doi: 10.1021/acscentsci.4c00660 (PMC11503496; doi:10.1021/acscentsci.4c00660)
Supplement: Supplementary file 1 — oc4c00660_si_001.pdf [file oc4c00660_si_001.pdf]

Supporting Information

Structural Reassignment of Covalent Organic Framework-Supported Palladium Species: Heterogenized Palladacycles as Efficient Catalysts for Sustainable C–H Activation

Meng-Ying Sun,[1] Sheung Chit Cheung,[1] Xue-Zhi Wang,[3] Ji-Kang Jin,[1,3] Jun Guo,[1] Dan Li,[3]* and Jian He[1,2]*

[1]Department of Chemistry, The University of Hong Kong, Pokfulam Road, Hong Kong, P. R. China

[2]State Key Laboratory of Synthetic Chemistry, The University of Hong Kong, Pokfulam Road, Hong Kong, P. R. China

[3]College of Chemistry and Materials Science, and Guangdong Provincial Key Laboratory of Functional Supramolecular Coordination Materials and Applications, Jinan University, Guangzhou 510632, China

Email: jianhe@hku.hk

Email: danli@jnu.edu.cn

Table of Contents

1. General information S–3
2. Investigation of room-temperature C−H cleavage triggered by palladium acetate S–5
3. Preparation of ligands S–10
4. Synthesis of COF materials S–12
   - 4.1 Synthesis of **Im-COF-1** S–12
   - 4.2 Synthesis of **Quin-COF-1** S–13
   - 4.3 Synthesis of **Im-COF-2** S–14
   - 4.4 Synthesis of other COF supports S–15
   - 4.5 Synthesis and characterization of cyclopalladated complexes S–17
   - 4.6 General procedure for the synthesis of COF-supported palladacycle catalysts S–21
5. Characterization of framework supports and heterogeneous palladium catalysts S–23
   - 5.1 Characterizations of COF materials S–23
   - 5.2 DFT calculation S–54
   - 5.3 Preparation of MOF-supported palladium catalysts S–56
6 Catalytic activity test of heterogeneous palladium catalysts S–58
   - 6.1 Synthesis of diaryliodonium salts S–58
   - 6.2 Synthesis of indole substrates S–64
   - 6.3 Heterogeneous palladium-catalyzed C–H arylation S–67
   - 6.4 Mechanistic studies S–98
   - 6.5 Recycling experiments of **Pd@Quin-COF-1** and **Pd@Im-COF-2** S–105
   - 6.6 Large-scale synthetic application S–109
   - 6.7 Investigation of heterogeneous catalysis with low palladium loadings S–110
   - 6.8 Investigation of C–H bromination and acetoxylation with **Pd@Quin-COF-1** S–111
7. References S–115
8. NMR spectra S–117

1. General information

Unless otherwise noted, materials were either purchased from commercial suppliers and used as received or prepared via literature procedures. Palladium 10% on carbon (wetted with ca. 55% water; Product Number: P1785; abbreviated as Pd/C) was purchased from TCI.

^{1}H NMR spectra were recorded on a Bruker 500 (500 MHz) or Bruker 400 (400 MHz) spectrometer in DMSO-d_6 or $CDCl_3$. Chemical shifts were quoted in parts per million (ppm) referenced to 0.0 ppm for tetramethylsilane. The following abbreviations (or combinations thereof) were used to explain multiplicities: s = singlet, d = doublet, t = triplet, q = quartet, m = multiplet, br = broad. Coupling constants, *J*, were reported in Hertz unit (Hz). ^{13}C NMR spectra were recorded on a Bruker 500 (125 MHz) or Bruker 400 (100 MHz) spectrometer in $CDCl_3$ and were fully decoupled by broad band proton decoupling. Chemical shifts were reported in ppm referenced to the center line of a triplet at 77.0 ppm of $CDCl_3$.

Powder X-ray diffraction (PXRD) patterns were recorded on a Rigaku MiniFlex600 X-ray diffractometer (CuKα, λ = 1.5418 Å), operating at 40 kV and 30 mA. The measurement parameters included a scan speed of 10° min^{-1}, a step size of 0.01°.

Scanning electron microscopy (SEM) were collected on a Zeiss Gemini 300 scanning electron microscope. TEM and EDS mapping were collected on a FEI Talos F200X scanning transmission electron Microscope with a Super X EDS detector.

X-ray photoelectron spectra (XPS) was carried out on a Thermo ESCALAB 250XI system, and the spectra were analyzed using the Thermo Scientific Avantage Data System software.

Inductively coupled plasma atomic emission spectroscopy (ICP-AES) measurements were conducted on a Thermo Scientific iCAP 7000 ICP-AES analyzer.

The thermogravimetry analyses (TGA) were carried out on TGA Q50 V20.6 with a heating rate of 10 °C min^{-1} from 40 to 800 °C in N_2 atmosphere.

Fourier transform infrared (FT-IR) spectra were collected on a Thermo Nicolet iS10 FT-IR spectrometer.

The solid-state ultraviolet-visible absorption spectra were recorded on a Bio-Logic MOS-500 multifunctional circular dichroism spectrometer using pressed KBr pellets and measured at a scan speed of 300 nm·min^{-1} with a bandwidth of 2 nm. All spectroscopy studies were

equipped with Biokine software V4.80.

High-resolution EI mass spectra were recorded on a Thermo Scientific DFS Magnetic Sector GC-HRMS system. High-resolution ESI-MS measurements were performed on a Bruker impact II high-resolution LC-QTOF mass spectrometer. Accurate masses from high-resolution mass spectra were reported for the molecular ion $[M]^+$ or $[M+H]^+$.

The ^{13}C cross-polarization magic angle spinning (CP-MAS) NMR spectra were recorded on a JEOL JNM-ECZR (600 MHz) JEOL ECZ600R spectrometer equipped with a 3.2 mm double-resonance MAS probe (JEOL RESONANCE Inc., Japan).

Nitrogen gas sorption experiments were performed on an ASAP 2460 surface area and porosimetry analyzer (Micromeritics). The samples were dried upon heating at 70 °C for 12 h under vacuum before the gas sorption experiments. Surface areas of the materials were obtained based on adsorption data analyzed by Brunauer-Emmett-Teller (BET) methods. The pore size distributions were calculated from the adsorption branches by density functional theory (DFT) method.

The Rietveld refinements were performed using Materials Studio 2018.

2. Investigation of room-temperature C−H cleavage triggered by palladium acetate

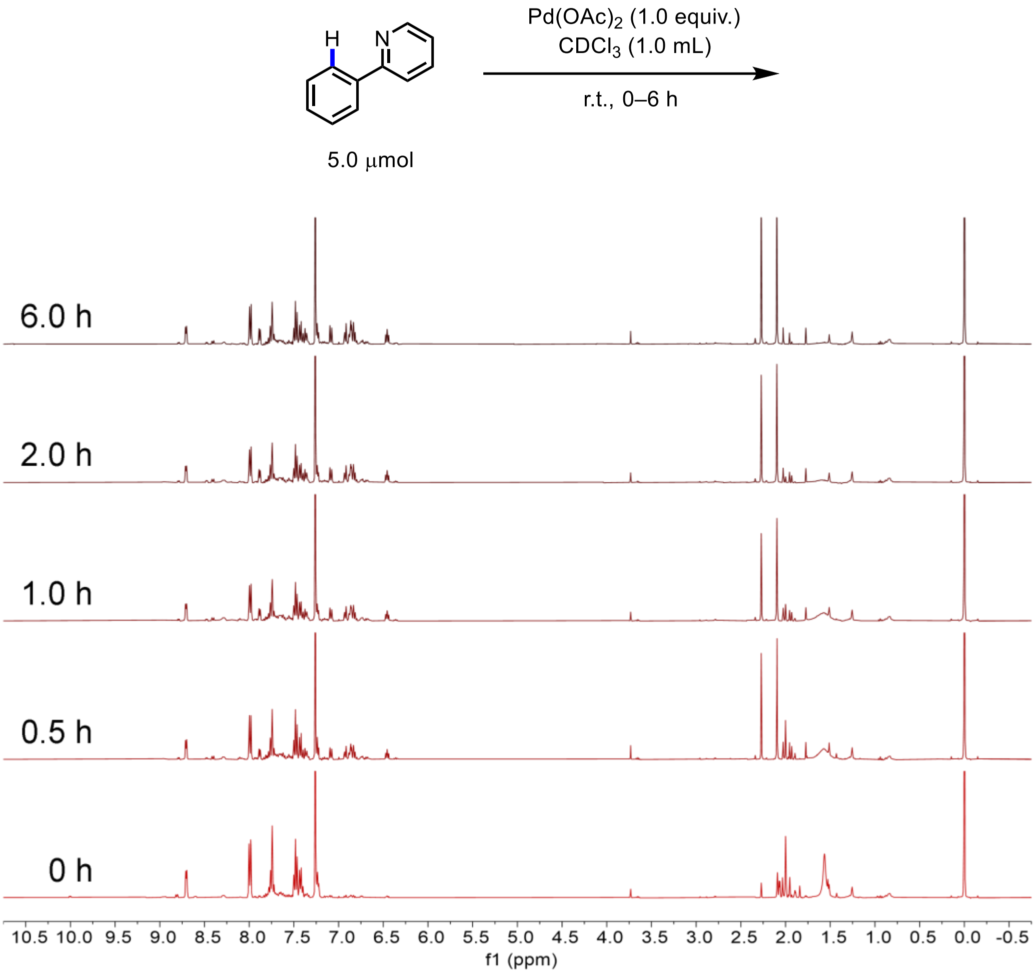

Figure S1. ^{1}H NMR monitoring of the reaction with a pyridine moiety as a directing group.

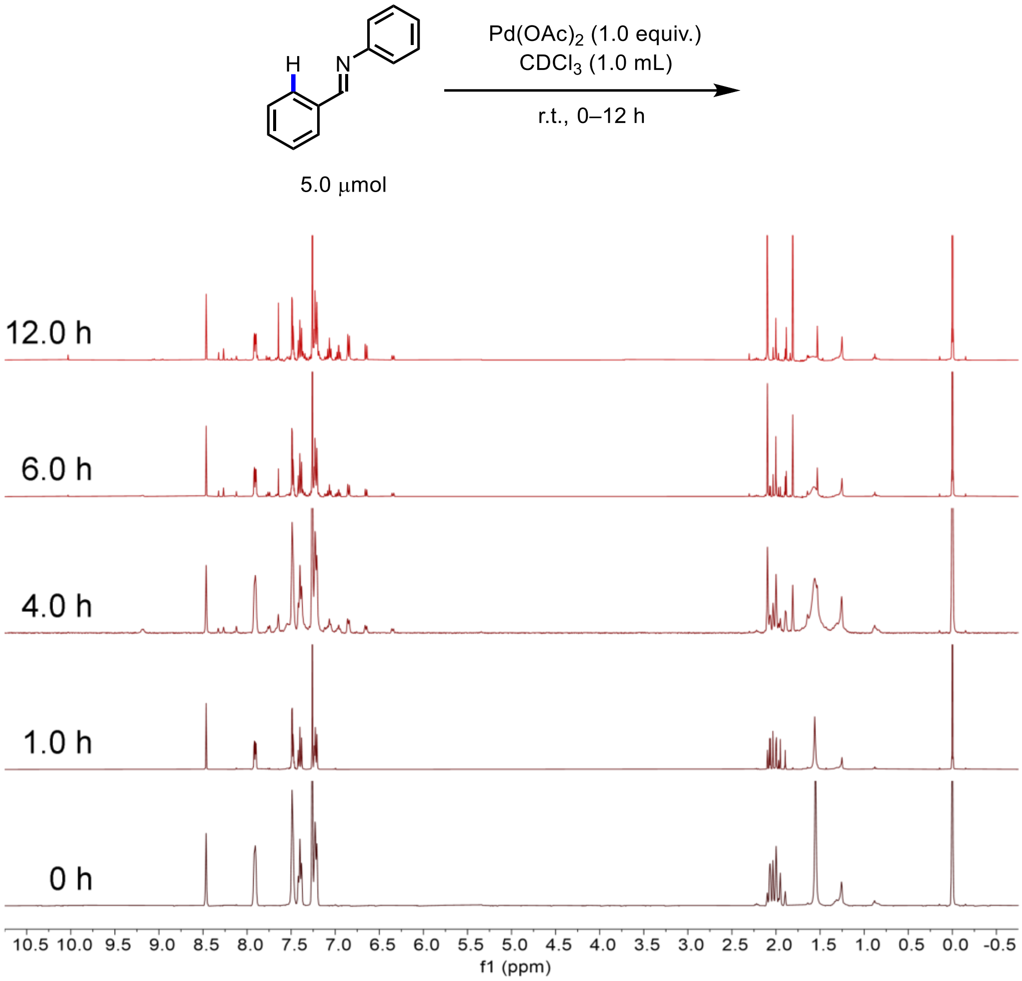

Figure S2. ^{1}H NMR monitoring of the reaction with an imine moiety as a directing group.

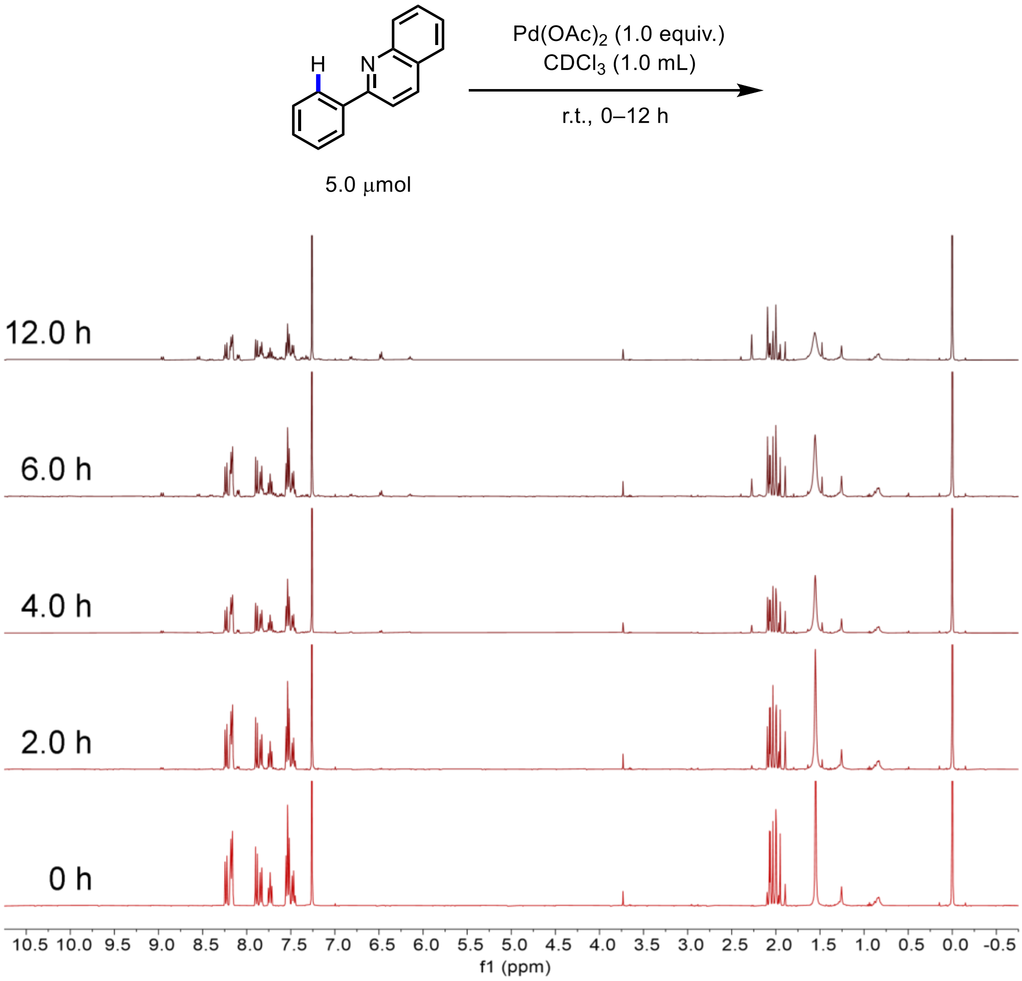

Figure S3. ^{1}H NMR monitoring of the reaction with a quinoline moiety as a directing group.

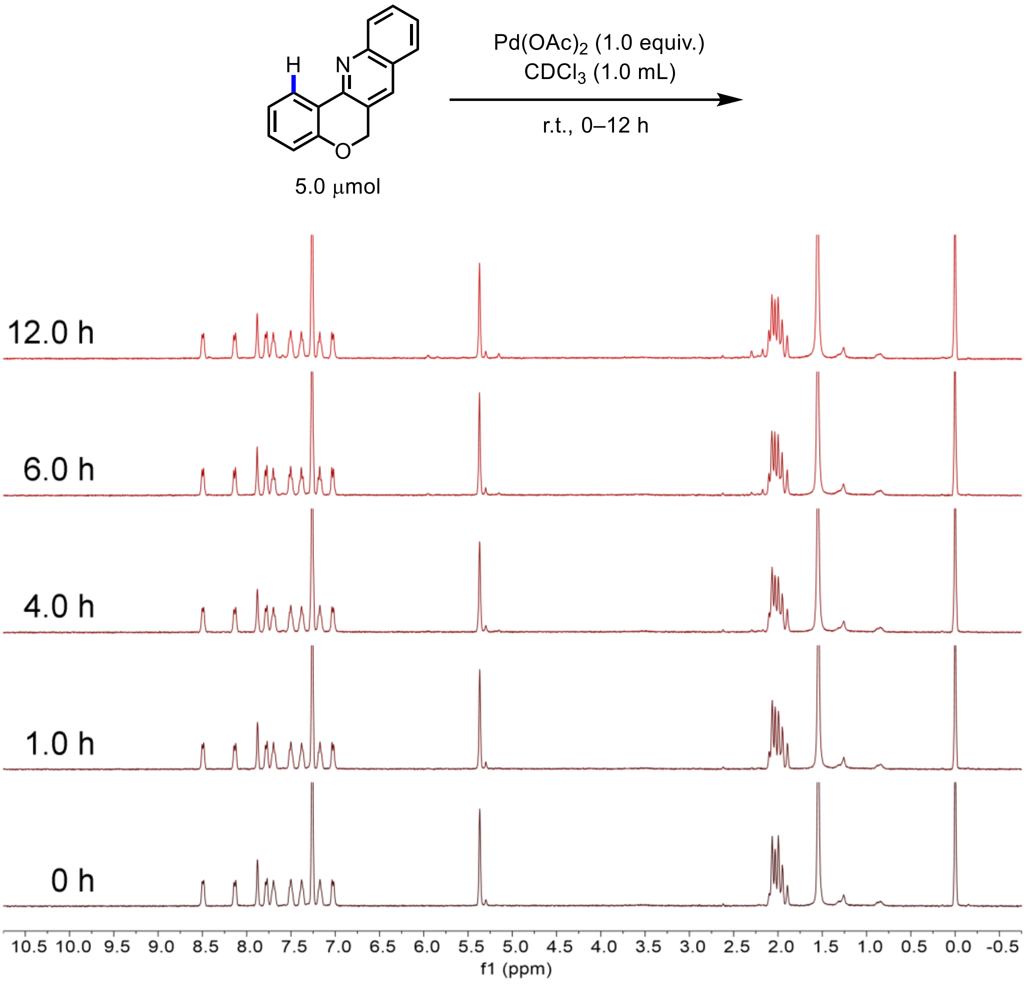

Figure S4. ^{1}H NMR monitoring of the reaction with a quinoline moiety in a designed COF matrix as a directing group.

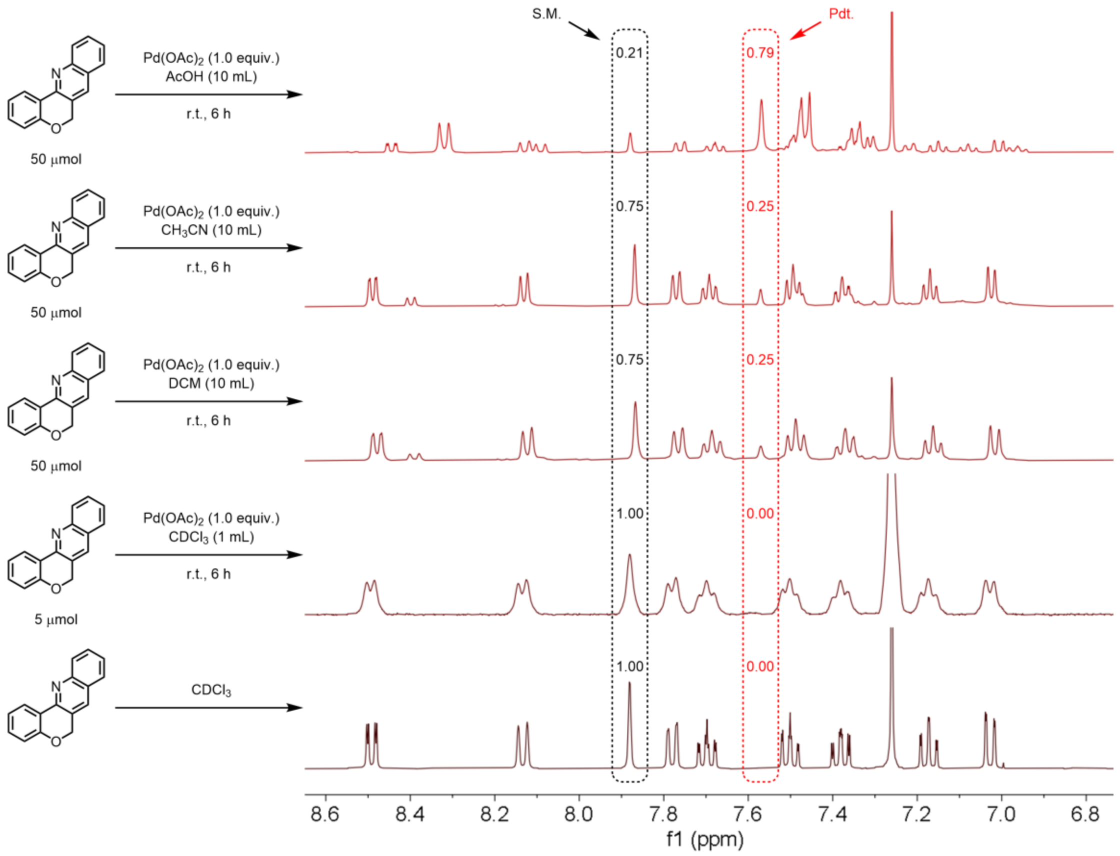

Figure S5. Solvent effect on the cyclopalladation of a quinoline moiety in a designed COF matrix.

3. Preparation of ligands

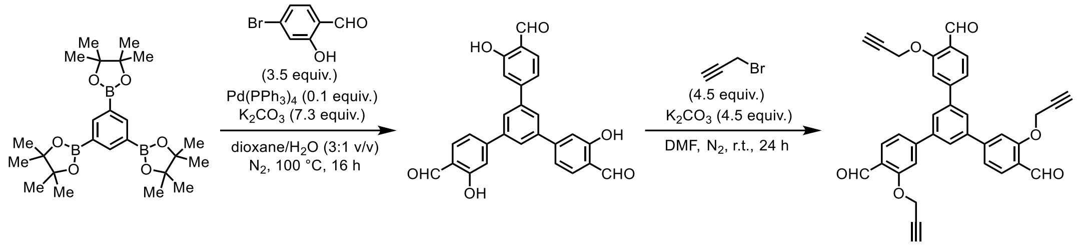

5'-(4-Formyl-3-hydroxyphenyl)-3,3''-dihydroxy-[1,1':3',1''-terphenyl]-4,4''-dicarbaldehyde (S1). To a solution of 1,3,5-tris(4,4,5,5-tetramethyl-1,3,2-dioxaborolan-2-yl)benzene (1.4 g, 3.0 mmol) in 1,4-dioxane (30 mL) and water (10 mL), 4-bromo-2-hydroxybenzaldehyde (2.1 g, 10.5 mmol), $Pd(PPh_3)_4$ (0.347 g, 0.30 mmol), and potassium carbonate (3.0 g, 21.9 mmol) were added. After stirring at 100 °C for 16 h under N_2 atmosphere, an aqueous solution of hydrochloric acid (1.0 M) was added to neutralize the reaction mixture to pH = 7. The formed precipitate was collected by filtration, washed extensively with water, methanol, and dichloromethane (DCM). Finally, the solid was collected and dried at 80 °C for 12 h to give **S1** as a white powder (931 mg, 71% yield).

^{1}H NMR (400 MHz, DMSO-d_6) δ 10.94 (s, 3H), 10.30 (s, 3H), 8.00 (s, 3H), 7.81 (d, J = 8.4 Hz, 3H), 7.51 (d, J = 8.4 Hz, 3H), 7.45 (s, 3H).

5'-(4-Formyl-3-(prop-2-yn-1-yloxy)phenyl)-3,3''-bis(prop-2-yn-1-yloxy)-[1,1':3',1''-terphenyl]-4,4''-dicarbaldehyde (L1). To a solution of 5'-(4-formyl-3-hydroxyphenyl)-3,3''-dihydroxy-[1,1':3',1''-terphenyl]-4,4''-dicarbaldehyde (**S1**) (0.29 g, 0.66 mmol) in dimethylformamide (7 mL), potassium carbonate (0.41 g, 3.0 mmol) and 3-bromoprop-1-yne (0.35 g, 3.0 mmol) was added. After stirring at room temperature under N_2 atmosphere for 24 h, deionized water (30 mL) were added and the reaction mixture was sitred for 30 minutes. The formed precipitate was collected by filtration, washed extensively with water, methanol, and DCM. Finally, the solid was collected and dried at 80 °C for 12 h to give **L1** as a pale brown powder (331 mg, 91% yield).

^{1}H NMR (400 MHz, DMSO-d_6) δ 10.41 (s, 3H), 8.17 (s, 3H), 7.87 (d, J = 6.4 Hz, 3H), 7.76–7.73 (m, 3H), 7.69 (d, J = 6.4 Hz, 3H), 5.19 (d, J = 1.6 Hz, 6H), 3.69 (d, J = 1.6 Hz, 3H).

^{13}C NMR (100 MHz, DMSO-d_6) δ 188.6, 159.8, 147.2, 140.7, 128.5, 126.6, 124.0, 120.7,

113.3, 79.2, 78.7, 56.8.

HRMS *m*/*z* (ESI) calcd. for $C_{36}H_{25}O_6$ $[M+H]^+$: 553.1646; found: 553.1628.

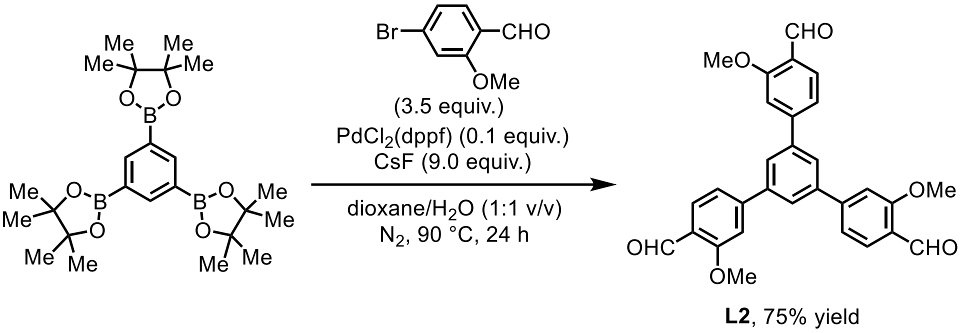

5'-(4-Formyl-3-methoxyphenyl)-3,3''-dimethoxy-[1,1':3',1''-terphenyl]-4,4''-dicarbaldehyde (L2).[1] To a solution of 1,3,5-tris(4,4,5,5-tetramethyl-1,3,2-dioxaborolan-2-yl)benzene (1.4 g, 3.0 mmol) in 1,4-dioxane (10 mL) and water (10 mL), 4-bromo-2-methoxybenzaldehyde (2.3 g, 10.5 mmol), $PdCl_2$(dppf) (73 mg, 0.3 mmol) and CsF (4.1 g, 27.0 mmol) were added. After stirring at 90 °C for 24 h under N_2 atmosphere, methanol (50 mL) was added and the reaction mixture was sitred for 30 minutes. The formed precipitate was collected by filtration, washed extensively with water, methanol, and DCM. Finally, the solid was collected and dried at 80 °C for 12 h to give **L2** as a white powder (1.1 g, 75% yield).

^{1}H NMR (400 MHz, DMSO-*d_6*) δ 10.53 (s, 3H), 7.97 (d, *J* = 8.0 Hz, 3H), 7.82 (s, 3H), 7.35 (d, *J* = 8.0 Hz, 3H), 7.23 (d, *J* = 1.6 Hz, 3H), 4.04 (s, 9H).

4. Synthesis of COF materials

4.1 Synthesis of Im-COF-1

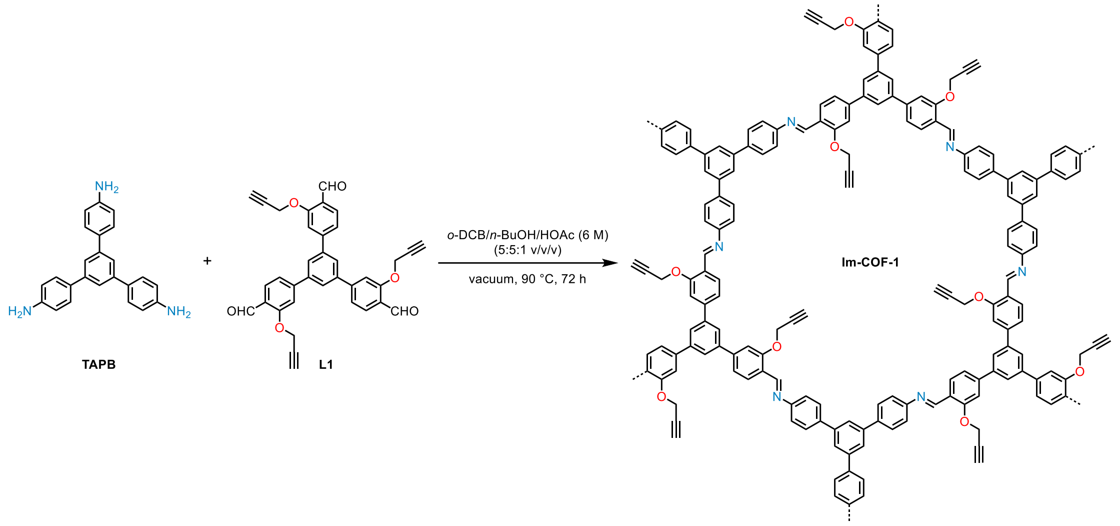

1,3,5-Tris(4-aminophenyl)-benzene (**TAPB**) (10.5 mg, 0.03 mmol) and **L1** (16.5 mg, 0.03 mmol) were weighed into a 10-mL Schlenk tube. Afterwards, *o*-dichlorobenzene (*o*-DCB, 0.5 mL) and *n*-butanol (*n*-BuOH, 0.5 mL) were added, and the mixture was sonicated for 1 h. After addition of 0.1 mL 6 M aqueous acetic acid, the tube was degassed by the three freeze-pump-thaw cycles and was then sealed. Upon warming to room temperature, the sealed tube was heated in an oven at 90 °C for 72 h. After reaction completion, the formed precipitate was collected by filtration, washed with DCM and further purification was carried out by Soxhlet extraction in DCM for 72 h. Finally, the solid was collected and dried at 70 °C for 12 h to give **Im-COF-1** as an orange powder ($C_{60}H_{39}N_3O_3$, 21.1 mg, 83% yield).

4.2 Synthesis of Quin-COF-1

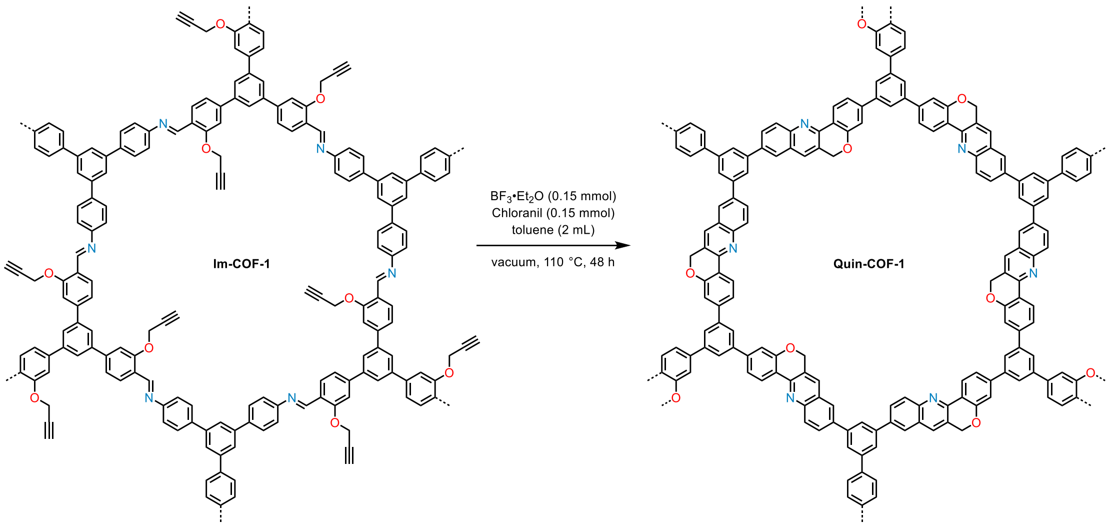

Chloranil (36.9 mg, 0.15 mmol) was first completely dissolved in 2 mL of toluene and then was added into a 10-mL Schlenk tube containing a mixture of parent **Im-COF-1** (25.0 mg) and BF_3•Et_2O (18.8 μL, 0.15 mmol). After degassed by typical three freeze-pump-thaw cycles, the tube was sealed and heated in oven at 110 °C for 48 h. After reaction completion and cooling to room temperature, the resulting dark red powder was collected by filtration and successively washed with EtOH, saturated aqueous sodium bicarbonate and acetonitrile. The yield of **Quin-COF-1** is 95%. Subsequently, the powder was activated by Soxhlet extraction in DCM and acetonitrile for 72 h, respectively, and then dried at 60 °C for 12 h ($C_{60}H_{33}N_3O_3$, 19.8 mg, 80% yield).

4.3 Synthesis of Im-COF-2

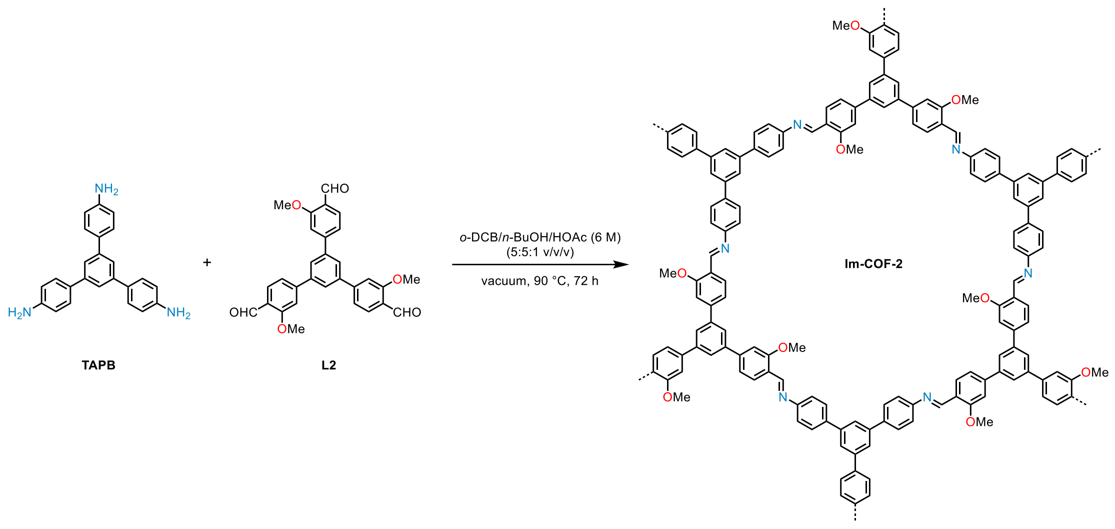

1,3,5-Tris(4-aminophenyl)-benzene (**TAPB**) (10.5 mg, 0.03 mmol) and **L2** (14.4 mg, 0.03 mmol) were weighed into a 10-mL Schlenk tube. Afterwards, *o*-DCB (0.5 mL) and *n*-BuOH (0.5 mL) were added, and the mixture was sonicated for 1 h. After addition of 0.1 mL 6 M aqueous acetic acid, the tube was degassed by the three freeze-pump-thaw cycles and was then sealed. Upon warming to room temperature, the sealed tube was heated in an oven at 90 °C for 72 h. After reaction completion, the formed precipitate was collected by filtration, washed with DCM and further purification was carried out by Soxhlet extraction in DCM for 72 h. Finally, the solid was collected and dried at 70 °C for 12 h to give **Im-COF-2** as a yellow powder ($C_{54}H_{39}N_3O_3$, 18.9 mg, 81% yield).

4.4 Synthesis of other COF supports

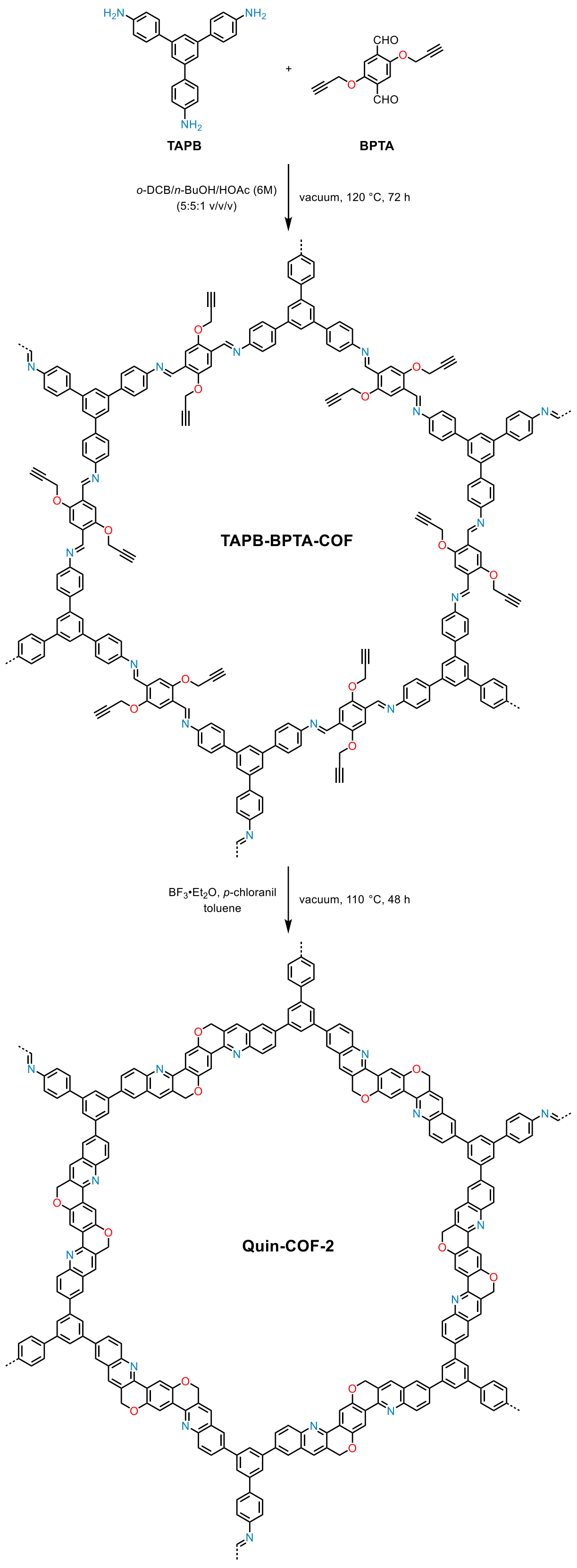

Figure S6. Synthesis of **Quin-COF-2**.[2]

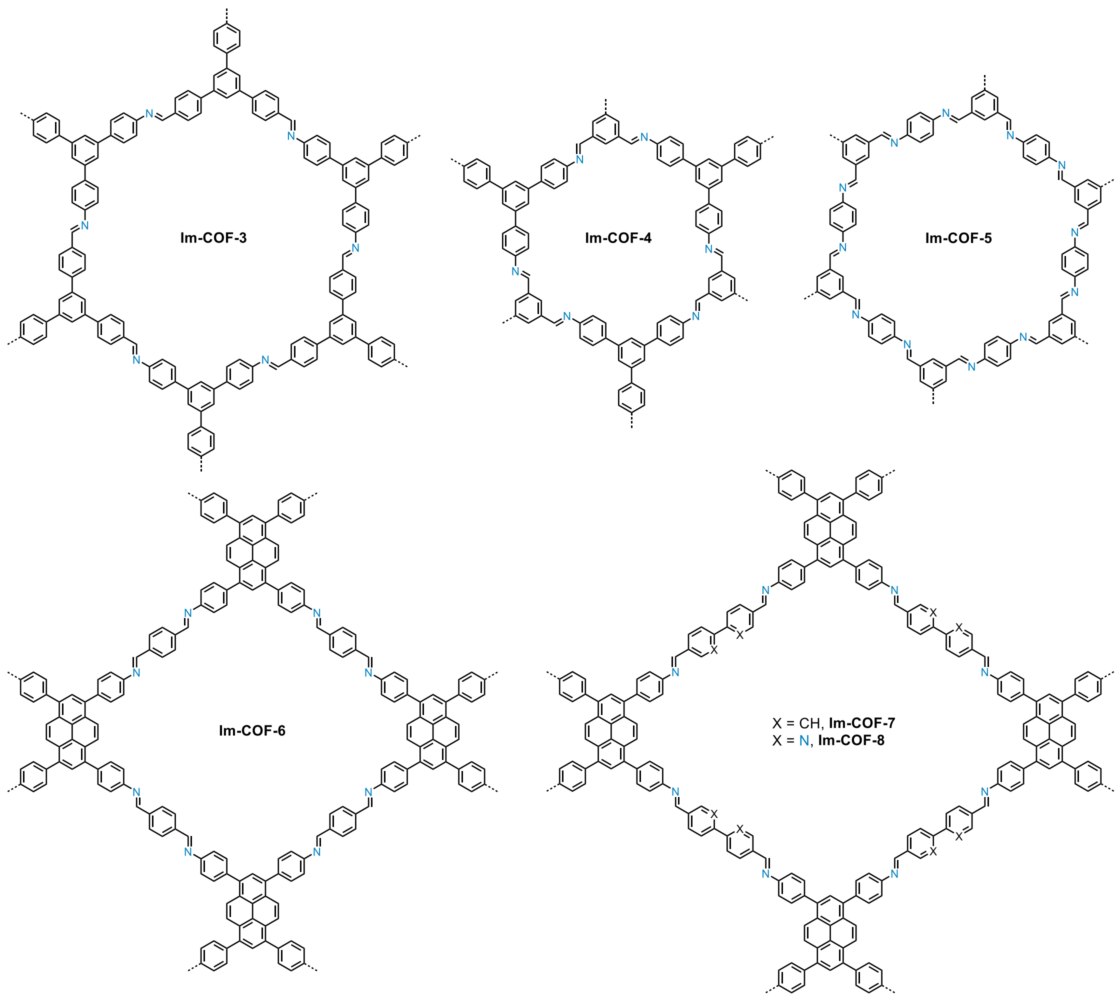

Figure S7. Im-COF-3 to **Im-COF-8** were synthesized according to the previous reported literature.[3]

4.5 Synthesis and characterization of cyclopalladated complexes

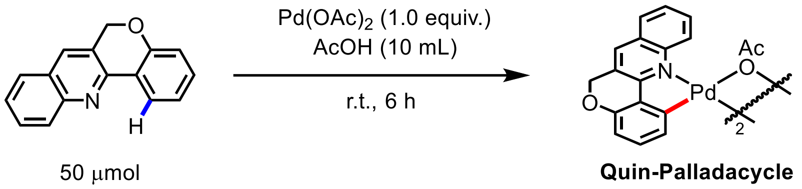

(1)

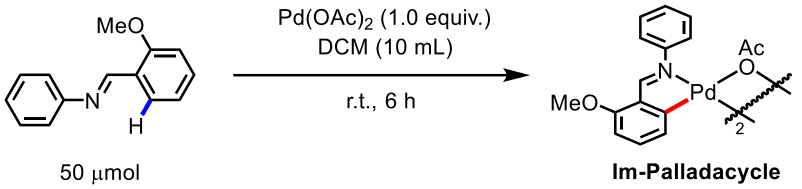

(2)

General procedure: cyclopalladated complexes were prepared using a modified literature procedure.[4] $Pd(OAc)_2$ (11.2 mg, 50 μmol, 1.0 equiv.) was weighed into a 25-mL Schlenk flask. Then acetic acid (10 mL) of DCM (10 mL) and 6*H*-chromeno[4,3-*b*]quinoline[5] or (*E*)-1-(2-methoxyphenyl)-*N*-phenylmethanimine (50 μmol, 1.0 equiv.) were added sequentially. The reaction was stirred at room temperature for 6 h. After cooling the reaction mixture, solvent was removed under vacuum to give a raw product, which was purified by recrystallization in a hexane and diethyl ether mixed solvent twice to yield a yellow solid (**Quin-Palladacycle** or **Im-Palladacycle**).

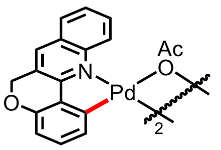

Quin-Palladacycle. Following the general procedure, the title complex was synthesized in 30% yield (6.0 mg).

¹H NMR (400 MHz, $CDCl_3$) δ 8.41 (d, J = 8.4 Hz, 1H), 7.59 (s, 1H), 7.49 (d, J = 7.6 Hz, 2H), 7.37 (d, J = 7.2 Hz, 1H), 5.97–5.92 (m, 2H), 5.83 (dd, J = 6.4, 2.4 Hz, 1H), 5.15 (s, 2H), 2.30 (s, 3H).

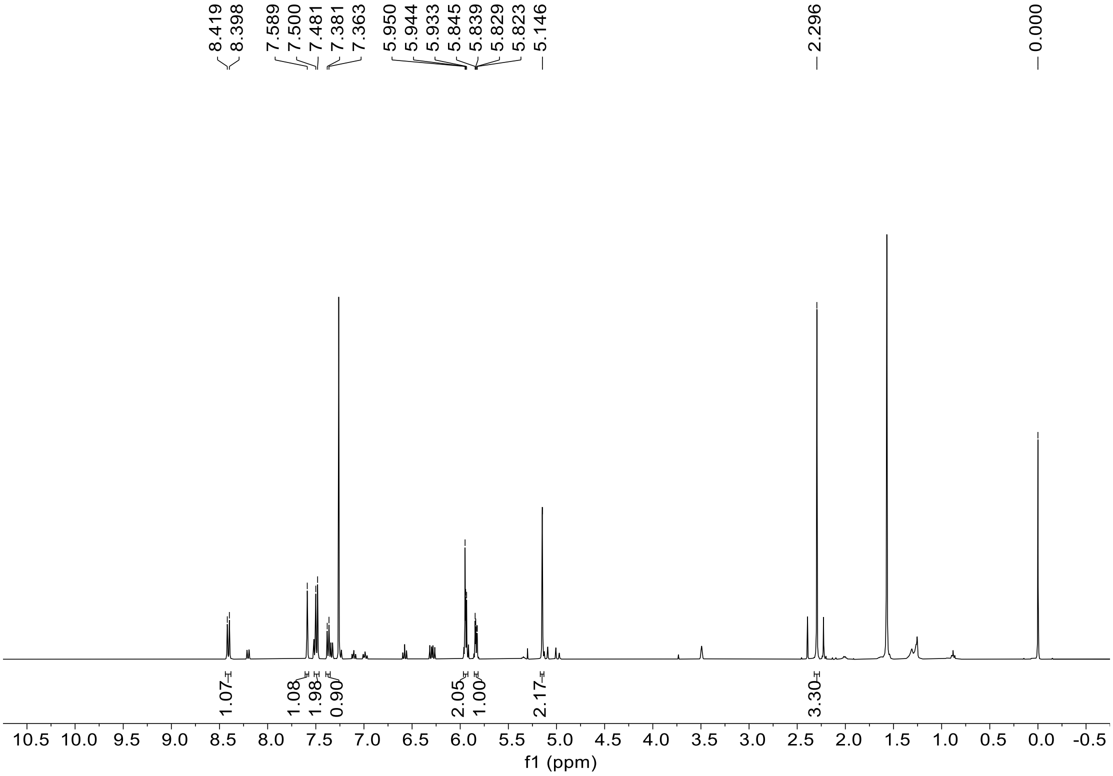

Figure S8. ¹H NMR spectrum of **Quin-Palladacycle**.

^{13}C NMR (100 MHz, $CDCl_3$) δ 181.2, 158.4, 154.5, 149.5, 147.3, 132.6, 130.3, 129.5, 127.9, 127.7, 126.1, 125.9, 124.9, 123.6, 110.9, 67.4, 24.9.

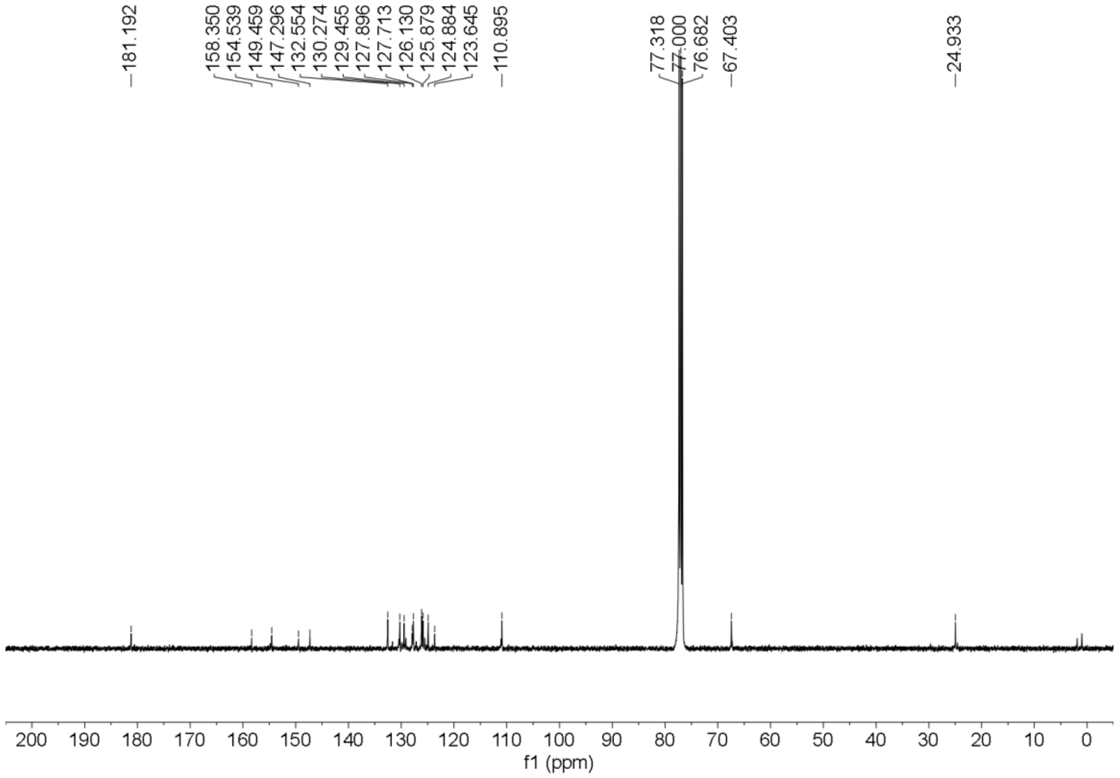

Figure S9. ^{13}C NMR spectrum of **Quin-Palladacycle**.

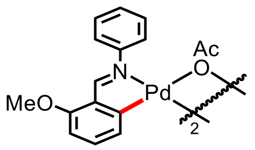

Im-Palladacycle. Following the general procedure, the title complex was synthesized in 35% yield (6.5 mg).

^{1}H NMR (400 MHz, $CDCl_3$) δ 7.99 (s, 1H), 7.23–7.15 (m, 3H), 6.99–6.89 (m, 3H), 6.53 (d, J = 8.4 Hz, 1H), 6.26 (d, J = 7.6 Hz, 1H), 3.80 (s, 3H), 1.78 (s, 3H).

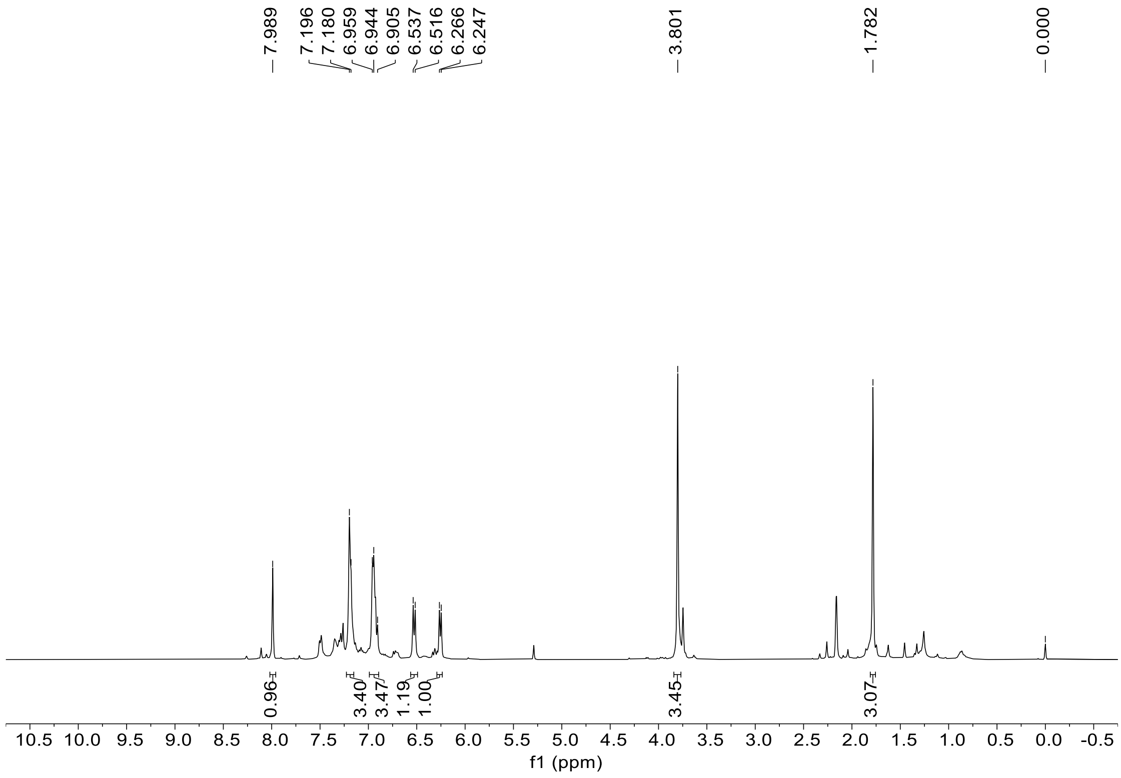

Figure S10. ^{1}H NMR spectrum of **Im-Palladacycle**.

4.6 General procedure for the synthesis of COF-supported palladacycle catalysts

Synthesis of Pd@Im-COF-2 to Pd@Im-COF-8: palladium acetate (45 mg, 0.2 mmol) was dissolved in DCM (10 mL), and then imine-linked COF (100 mg) was added. The mixture was stirred slowly at room temperature for 48 h. After reaction completion, the resulting solid was isolated by filtration and washed with DCM (3 × 30 mL), then dried at 70 °C for 12 h to yield the corresponding COF-supported palladium catalysts.

Synthesis of Pd@Quin-COF-1 and Pd@Quin-COF-2: the preparation procedures of **Pd@Quin-COF-1** and **Pd@Quin-COF-2** were almost the same as those of **Pd@Im-COFs**. Briefly, to the solution of palladium acetate (45 mg, 0.2 mmol) in acetic acid (10 mL), **Quin-COF-1** or **Quin-COF-2** (100 mg) was added. The mixture was stirred slowly at room temperature for 48 h. After reaction completion, the resulting solid was isolated by filtration and washed with a large amount of acetic acid, then dried at 70 °C for 12 h to afford **Pd@Quin-COF-1** or **Pd@Quin-COF-2**.

Table S1. Palladium Loadings in Various COF-Supported Catalysts

Entry	**Pd@COF**	Color	Pd Loading (wt%)[a]	Pd/N Ratio (experimental data)	Pd/N Ratio (theoretical data)
1	**Pd@Quin-COF-1**	dark red	2.6	1:13	1:1
2	**Pd@Quin-COF-2**	dark red	0.7	1:63	1:1
3	**Pd@Im-COF-2**	brown	8.3	1:4.1	1:1
4	**Pd@Im-COF-3**	brown	10.0	1:3.7	1:1
5	**Pd@Im-COF-4**	brown	6.7	1:8.9	1:1
6	**Pd@Im-COF-5**	brown	9.5	1:9.9	1:1
7	**Pd@Im-COF-6**	brown	7.4	1:6.3	1:1
8	**Pd@Im-COF-7**	brown	5.7	1:7.0	1:1
9	**Pd@Im-COF-8**	black	16.0	1:3.7	1:4

[a]Loading was determined by ICP-AES.

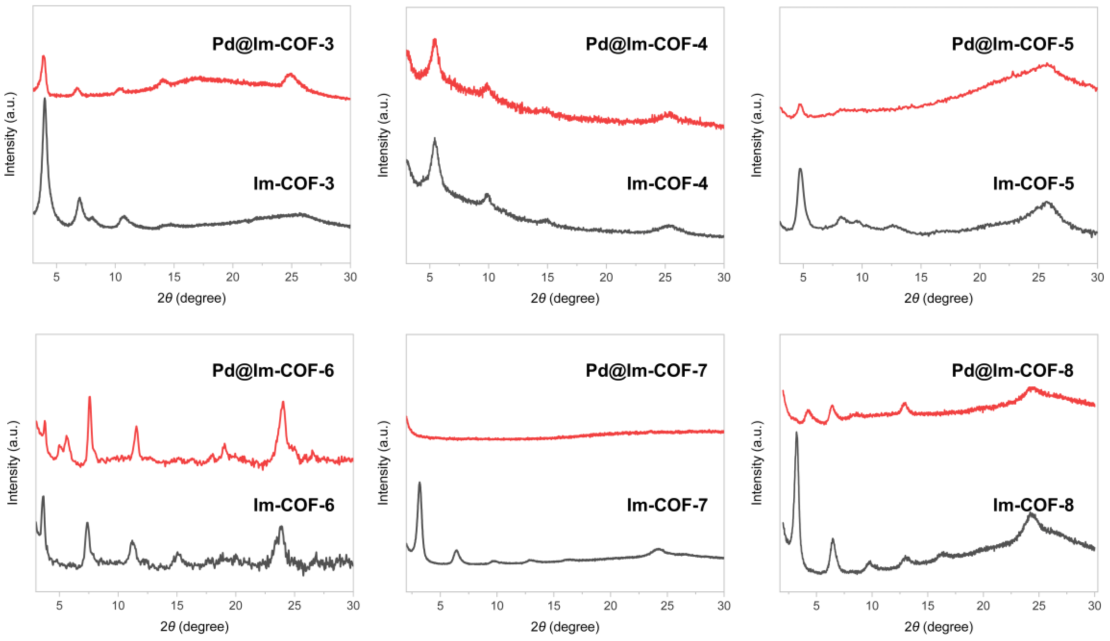

Figure S11. PXRD patterns of **Im-COF-3** to **Im-COF-8** before and after metalation with $Pd(OAc)_2$.

5. **Characterization of framework supports and heterogeneous palladium catalysts**

5.1 Characterizations of COF materials

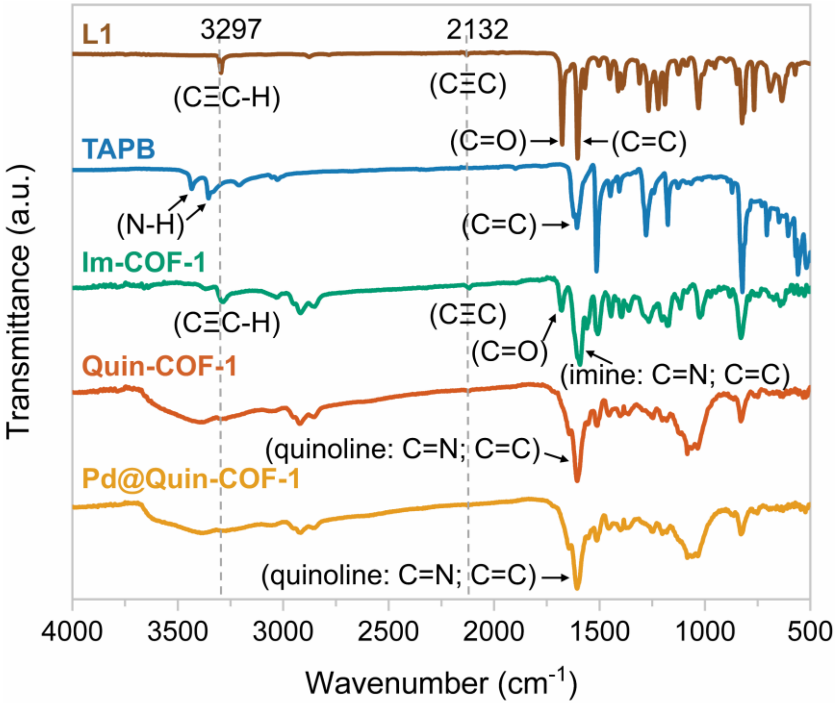

Figure S12. FT-IR spectra of **L1**, **TAPB**, **Im-COF-1**, **Quin-COF-1**, and **Pd@Quin-COF-1**. All the COFs and organic linkers contain arene C=C vibrational peaks around 1600 cm^{-1}.[6]

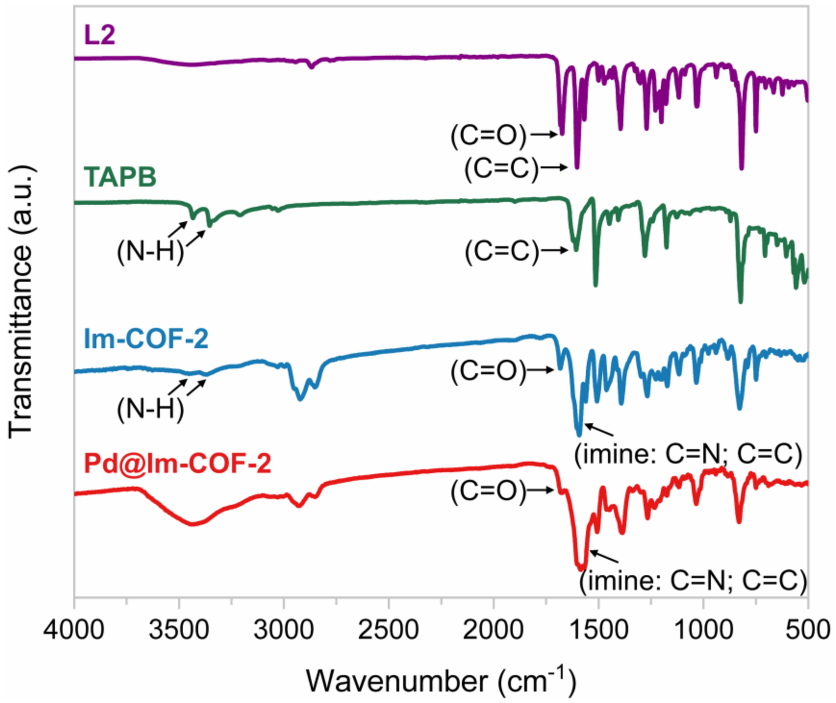

Figure S13. FT-IR spectra of **L2**, **TAPB**, **Im-COF-2**, and **Pd@Im-COF-2**. All the COFs and organic linkers contain arene C=C vibrational peaks around 1600 cm^{-1}.[6]

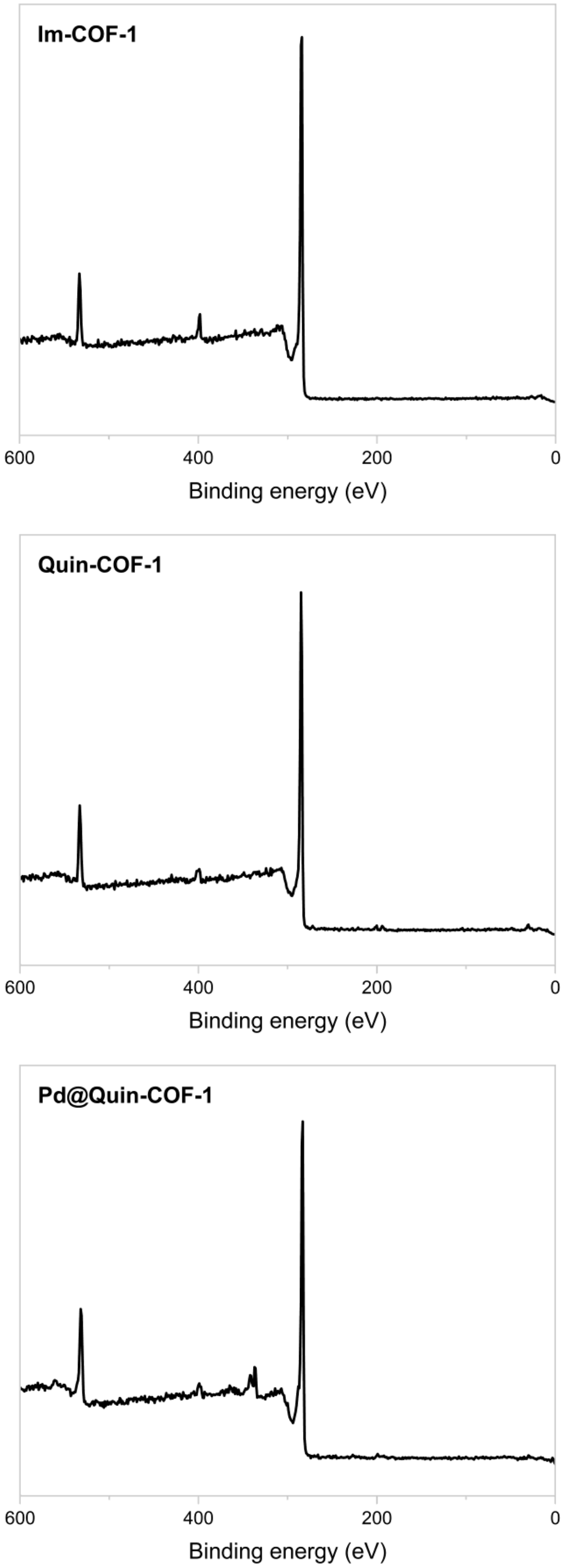

Figure S14. Full-scale XPS spectra of **Im-COF-1**, **Quin-COF-1**, and **Pd@Quin-COF-1**.

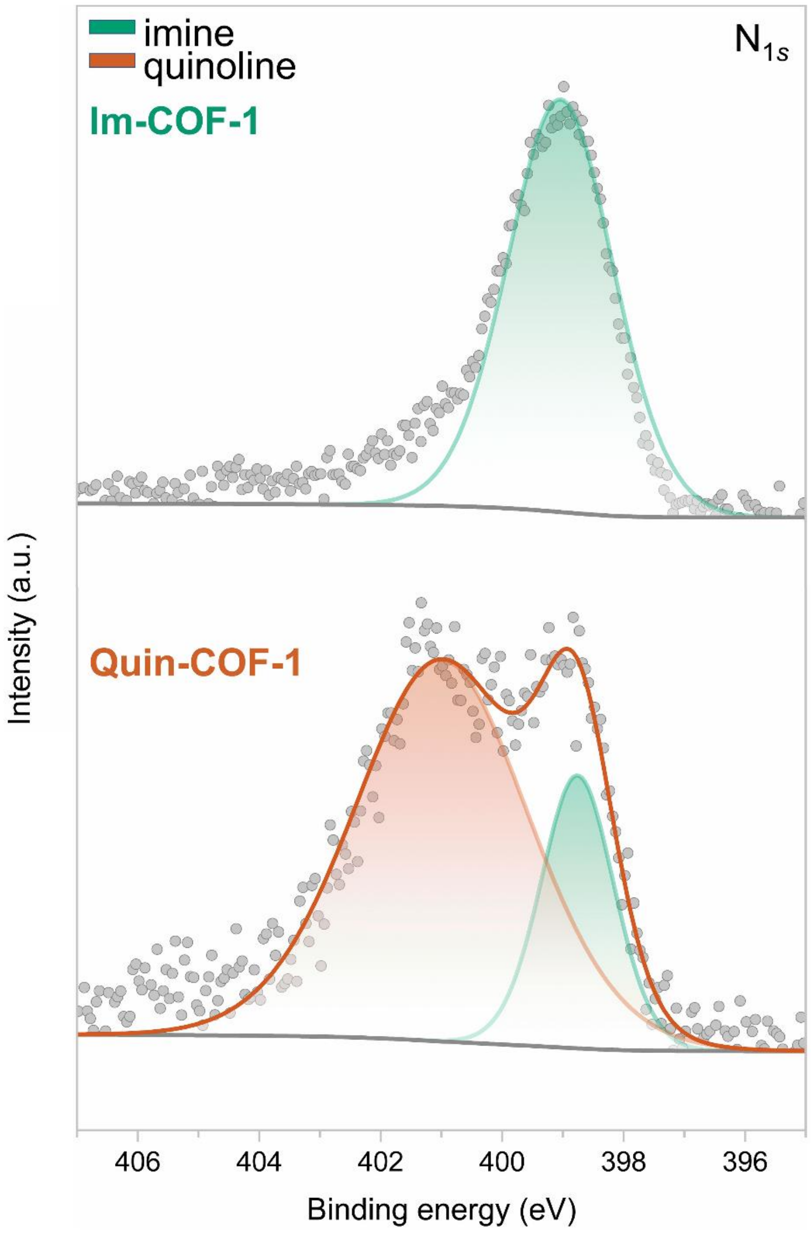

Quin-COF-1	
Imine (398.70 eV)	23%
Quinoline (401.01 eV)	77%

Figure S15. N1*s* XPS spectra of **Im-COF-1** and **Quin-COF-1**. The proportion of various chemical states of **Quin-COF-1** was evaluated based on integration of the peak areas.

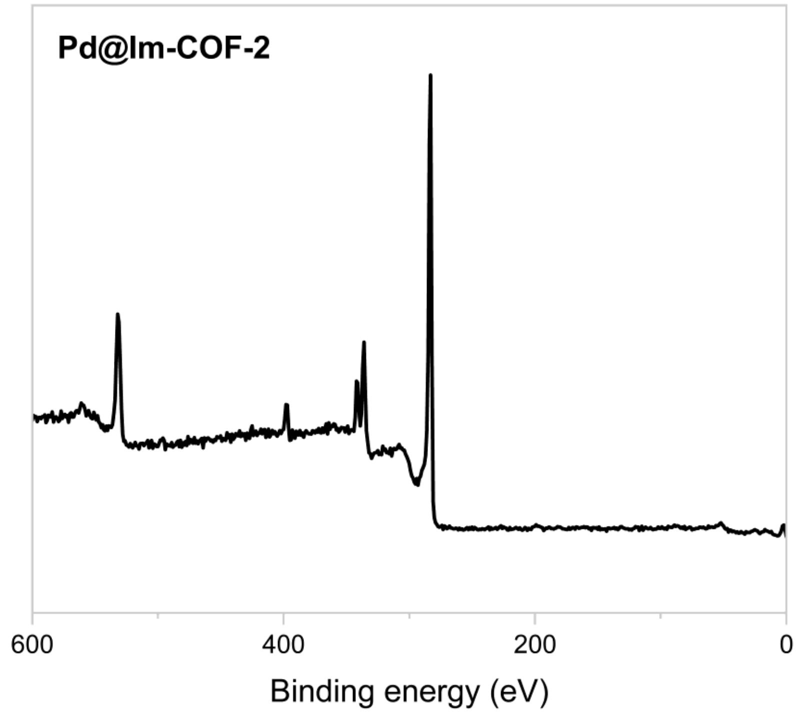

Figure S16. Full-scale XPS spectrum of **Pd@Im-COF-2**.

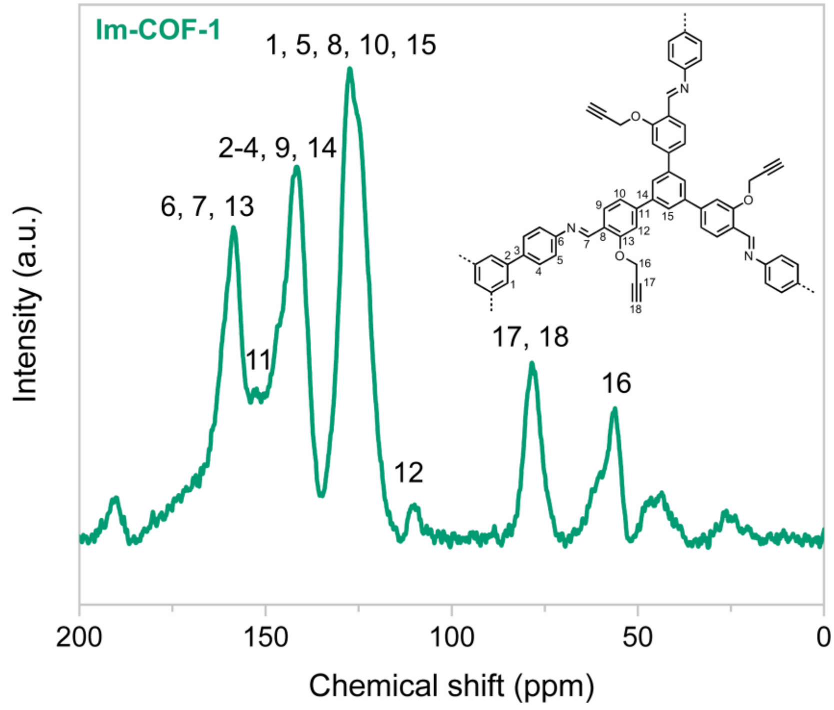

Figure S17. ^{13}C CP-MAS NMR spectrum of **Im-COF-1**. The assignments of the ^{13}C chemical shifts are shown in the chemical structure.

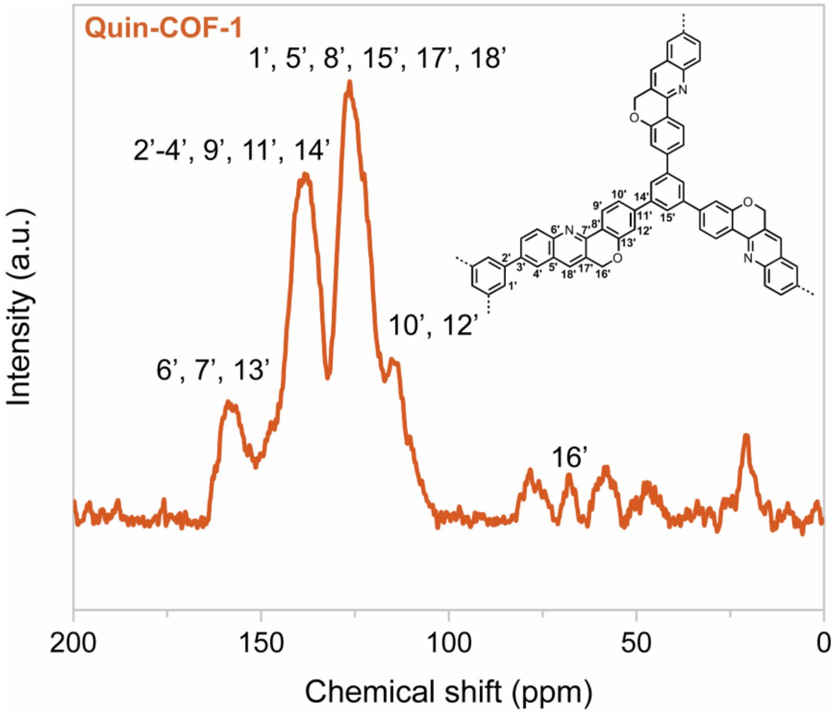

Figure S18. ^{13}C CP-MAS NMR spectra of **Quin-COF-1**. The assignments of the ^{13}C chemical shifts are shown in the chemical structure.

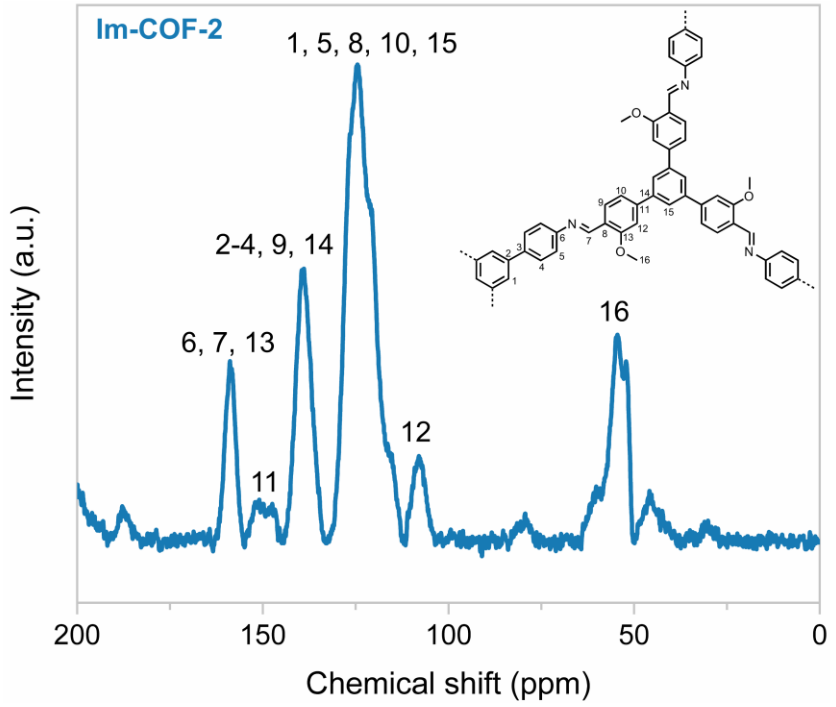

Figure S19. ^{13}C CP-MAS NMR spectrum of **Im-COF-2**. The assignments of the ^{13}C chemical shifts are shown in the chemical structure.

Table S2. Fractional Atomic Coordinates for the Unit Cell of Im-COF-1 with AA Stacking

Crystal system		Hexagonal	
Space group		*P*-6	
Unit cell		$a = b = 25.61$ Å,<br>$c = 3.92$ Å,<br>$\alpha = \beta = 90°$, $\gamma = 120°$	
Rietveld refinement		$R_{wp} = 3.35$ %, $R_p = 2.61$%	
Atom	x	y	z
H1	1.39997	-0.76097	0.72502
N2	1.01903	-0.47856	0.5
C3	1.07844	-0.44804	0.5
C4	1.10716	-0.48081	0.5
C5	1.16876	-0.4529	0.5
C6	1.20597	-0.39061	0.5
C7	1.17482	-0.35908	0.5
C8	1.11325	-0.3865	0.5
C9	1.26933	-0.36211	0.5
C10	1.30017	-0.3936	0.5
C11	1.46041	-0.56041	0.5
C12	1.50019	-0.58169	0.5
C13	1.48031	-0.64256	0.5
C14	1.52143	-0.66226	0.5
C15	1.58354	-0.62433	0.5
C16	1.60111	-0.56357	0.5
C17	1.56128	-0.54282	0.5
C18	1.62487	-0.64543	0.5
C19	1.60738	-0.70598	0.5

O20	1.89796	-0.4212	0.5
C21	1.38907	-0.74361	0.5
C22	1.3305	-0.7626	0.5
C23	1.94166	-0.2792	0.5
H24	1.08323	-0.5257	0.5
H25	1.18474	-0.4809	0.5
H26	1.19555	-0.31458	0.5
H27	1.09435	-0.36078	0.5
H28	1.27606	-0.4374	0.5
H29	1.41643	-0.58962	0.5
H30	1.50367	-0.70632	0.5
H31	1.64388	-0.53143	0.5
H32	1.57791	-0.49839	0.5
H33	1.5643	-0.7346	0.5
H34	1.96852	-0.23961	0.5

Table S3. Fractional Atomic Coordinates for the Unit Cell of Quin-COF-1 with AA Stacking

Crystal system		Triclinic	
Space group		$P1$	
Unit cell		a = 25.35 Å, b = 26.27 Å, c = 3.72 Å, $\alpha = \beta$ = 90°, γ = 120°	
Rietveld refinement		R_{wp} = 1.99%, R_p = 1.52%	
Atom	x	y	z
N1	1.01838	-0.49528	0.60902
C2	1.07878	-0.4618	0.61699
C3	1.11306	-0.48357	0.77197
C4	1.17567	-0.44855	0.77835
C5	1.20598	-0.39166	0.62181
C6	1.17017	-0.37053	0.46907
C7	1.10724	-0.40444	0.46945
C8	1.26947	-0.35752	0.6076
C9	1.30507	-0.38462	0.57769
C10	1.36794	-0.35173	0.53822
C11	1.39458	-0.28937	0.50939
C12	1.362	-0.25953	0.55658
C13	1.29988	-0.29475	0.61591
C14	1.403	-0.37993	0.53513
C15	1.39046	-0.19665	0.5448
C16	1.44843	-0.16042	0.6842
C17	1.47712	-0.09794	0.6696
C18	1.44689	-0.07069	0.52061

C19	1.38936	-0.10566	0.3835
C20	1.36188	-0.16781	0.39267
C21	1.38152	-0.43728	0.39179
C22	1.41472	-0.46679	0.41336
C23	1.47195	-0.43764	0.56791
C24	1.49503	-0.38017	0.70084
C25	1.46063	-0.35217	0.68804
N26	1.504	-0.46576	0.59132
C27	1.48448	-0.5219	0.47686
C28	1.52046	-0.54829	0.51516
C29	1.49911	-0.6063	0.38741
C30	1.53441	-0.63334	0.4059
C31	1.59181	-0.60441	0.56063
C32	1.6116	-0.5473	0.70163
C33	1.57712	-0.51943	0.67562
C34	1.62812	-0.63123	0.56223
C35	1.60288	-0.69338	0.59455
C36	1.63679	-0.72193	0.55137
C37	1.69875	-0.68554	0.49063
C38	1.72781	-0.6228	0.49476
C39	1.69092	-0.59709	0.52326
C40	1.60975	-0.78467	0.56823
C41	1.7912	-0.58736	0.47738
C42	1.82847	-0.60706	0.62531
C43	1.89104	-0.57195	0.6199
C44	1.91926	-0.51458	0.47403
C45	1.88275	-0.4941	0.32843
C46	1.82022	-0.53031	0.32314

C47	1.55188	-0.82238	0.43317
C48	1.52477	-0.88445	0.45373
C49	1.55507	-0.91141	0.60437
C50	1.61329	-0.87416	0.73397
C51	1.63964	-0.8121	0.72048
C52	1.52991	-0.97356	0.62992
N53	1.47395	-0.01071	0.50872
C54	1.98194	-0.47703	0.46991
O55	1.90771	-0.43852	0.19347
O56	1.44403	-0.63682	0.24109
O57	1.64486	-0.89793	0.87409
C58	0.40308	1.38486	1.20116
C59	0.42716	1.44684	1.32168
C60	0.39286	1.47505	1.28902
C61	0.97048	1.6039	1.18673
C62	1.00839	1.58078	1.32636
C63	1.07119	1.61653	1.32535
C64	0.6243	1.03981	1.90392
C65	0.56232	1.00121	1.77797
C66	0.53528	0.93889	1.79836
H67	1.09264	-0.52473	0.88269
H68	1.19877	-0.4655	0.90157
H69	1.18981	-0.33013	0.3493
H70	1.28457	-0.42975	0.58504
H71	1.43908	-0.26494	0.45225
H72	1.27604	-0.27396	0.66679
H73	1.47018	-0.17951	0.80077
H74	1.3675	-0.08613	0.27406

H75	1.32055	-0.19178	0.28294
H76	1.34105	-0.4583	0.26986
H77	1.53642	-0.35888	0.81217
H78	1.47827	-0.31121	0.79912
H79	1.5177	-0.67447	0.29818
H80	1.65168	-0.52496	0.82659
H81	1.59397	-0.47781	0.77673
H82	1.55843	-0.71859	0.65139
H83	1.72348	-0.70544	0.44169
H84	1.71033	-0.55204	0.51493
H85	1.81061	-0.64726	0.74585
H86	1.9156	-0.58865	0.72845
H87	1.79615	-0.51405	0.20406
H88	1.52864	-0.8051	0.31448
H89	1.48275	-0.90963	0.35469
H90	1.68115	-0.78708	0.82627
H91	0.36233	1.35542	1.34219
H92	0.3916	1.38191	0.93084
H93	0.35159	1.45285	1.17615
H94	0.97784	1.64262	1.33429
H95	0.98315	1.61804	0.92289
H96	1.0906	1.65809	1.21969
H97	0.62701	1.03018	2.17243
H98	0.65489	1.03042	1.76046
H99	0.55827	0.9204	1.90651

Table S4. Fractional Atomic Coordinates for the Unit Cell of Im-COF-2 with AA Stacking

Crystal system		Triclinic	
Space group		*P*1	
Unit cell		a = 26.08 Å, b = 25.59 Å, c = 3.81 Å, $\alpha = \beta$ = 90°, γ = 120°	
Rietveld refinement		R_{wp} = 2.44%, R_p = 1.90%	
Atom	x	y	z
C1	0.48822	-1.62136	0.47047
C2	0.52746	-1.64345	0.50667
C3	0.58652	-1.61146	0.37921
C4	0.60341	-1.55659	0.20581
C5	0.56517	-1.53243	0.17797
C6	0.50654	-1.56504	0.31501
C7	0.62682	-1.63303	0.43549
C8	0.60677	-1.69513	0.45636
C9	0.64427	-1.71771	0.55653
C10	0.70485	-1.67581	0.61503
C11	0.72883	-1.61375	0.56021
C12	0.6886	-1.59345	0.48225
C13	0.62214	-1.78003	0.59717
C14	0.79116	-1.57399	0.57605
C15	0.56572	-1.81794	0.74858
C16	0.54266	-1.88021	0.78731
C17	0.57693	-1.90593	0.67254
C18	0.6339	-1.86823	0.52978
C19	0.65573	-1.80664	0.48909

C20	0.83099	-1.59114	0.43658
C21	0.89321	-1.55282	0.44171
C22	0.91716	-1.49425	0.58236
C23	0.87775	-1.47646	0.71849
C24	0.81603	-1.5158	0.71938
C25	0.46562	-1.5441	0.31745
C26	0.97863	-1.45285	0.58894
C27	0.55638	-1.96775	0.68897
N28	0.48078	-1.48678	0.2479
C29	0.4486	-1.4587	0.27522
C30	0.4746	-1.39969	0.14717
C31	0.44524	-1.36654	0.17996
C32	0.38927	-1.39048	0.34946
C33	0.36334	-1.45031	0.47195
C34	0.39195	-1.48395	0.43315
C35	0.36091	-1.35617	0.40842
C36	0.39402	-1.29371	0.47211
C37	0.3669	-1.25941	0.55594
C38	0.30437	-1.29044	0.59366
C39	0.26877	-1.35198	0.51591
C40	0.29815	-1.3833	0.41503
C41	0.40059	-1.19621	0.59476
C42	0.20589	-1.38103	0.53859
C43	0.17441	-1.35224	0.42651
C44	0.11216	-1.38075	0.45059
C45	0.07919	-1.4396	0.5826
C46	0.10985	-1.4689	0.6921
C47	0.17218	-1.4399	0.67359

C48	0.4582	-1.16699	0.7459
C49	0.49212	-1.10418	0.76597
C50	0.46896	-1.06847	0.6407
C51	0.41116	-1.09688	0.50025
C52	0.37795	-1.1595	0.47361
N53	0.49826	-1.00798	0.64259
N54	0.01921	-1.47096	0.60241
O55	0.58275	-1.47872	0.0185
C56	0.64035	-1.4301	-0.07248
O57	0.9309	-1.56987	0.30187
C58	0.9226	-1.62748	0.21119
O59	0.48859	-1.91631	0.93888
C60	0.44449	-1.90269	1.05877
H61	0.44609	-1.64672	0.56852
H62	0.51203	-1.68316	0.63649
H63	0.64436	-1.53377	0.09314
H64	0.56349	-1.72505	0.39672
H65	0.73241	-1.69088	0.69974
H66	0.70503	-1.54847	0.45906
H67	0.54094	-1.7999	0.83592
H68	0.65989	-1.88538	0.44746
H69	0.69653	-1.78156	0.37481
H70	0.81432	-1.632	0.31987
H71	0.89365	-1.43458	0.82233
H72	0.78948	-1.50129	0.82795
H73	0.42258	-1.57344	0.38584
H74	0.99274	-1.40805	0.58842
H75	0.58632	-1.98215	0.72018

H76	0.51538	-1.38029	0.02838
H77	0.46568	-1.32434	0.07681
H78	0.32342	-1.47026	0.60001
H79	0.37124	-1.52656	0.52925
H80	0.43927	-1.27245	0.45763
H81	0.28413	-1.26754	0.68066
H82	0.27293	-1.42724	0.34392
H83	0.19645	-1.31012	0.32171
H84	0.09127	-1.35844	0.36548
H85	0.08694	-1.51163	0.78819
H86	0.19266	-1.46244	0.7626
H87	0.47629	-1.19113	0.84523
H88	0.53355	-1.08497	0.87493
H89	0.39307	-1.07207	0.41047
H90	0.33701	-1.17778	0.3582
H91	0.64439	-1.42648	-0.35521
H92	0.67674	-1.43485	0.03263
H93	0.64435	-1.38881	0.02991
H94	0.95941	-1.63122	0.31959
H95	0.9234	-1.63109	-0.07145
H96	0.88123	-1.66446	0.31146
H97	0.40851	-1.94249	1.18145
H98	0.42643	-1.88958	0.8406
H99	0.46335	-1.86657	1.2502

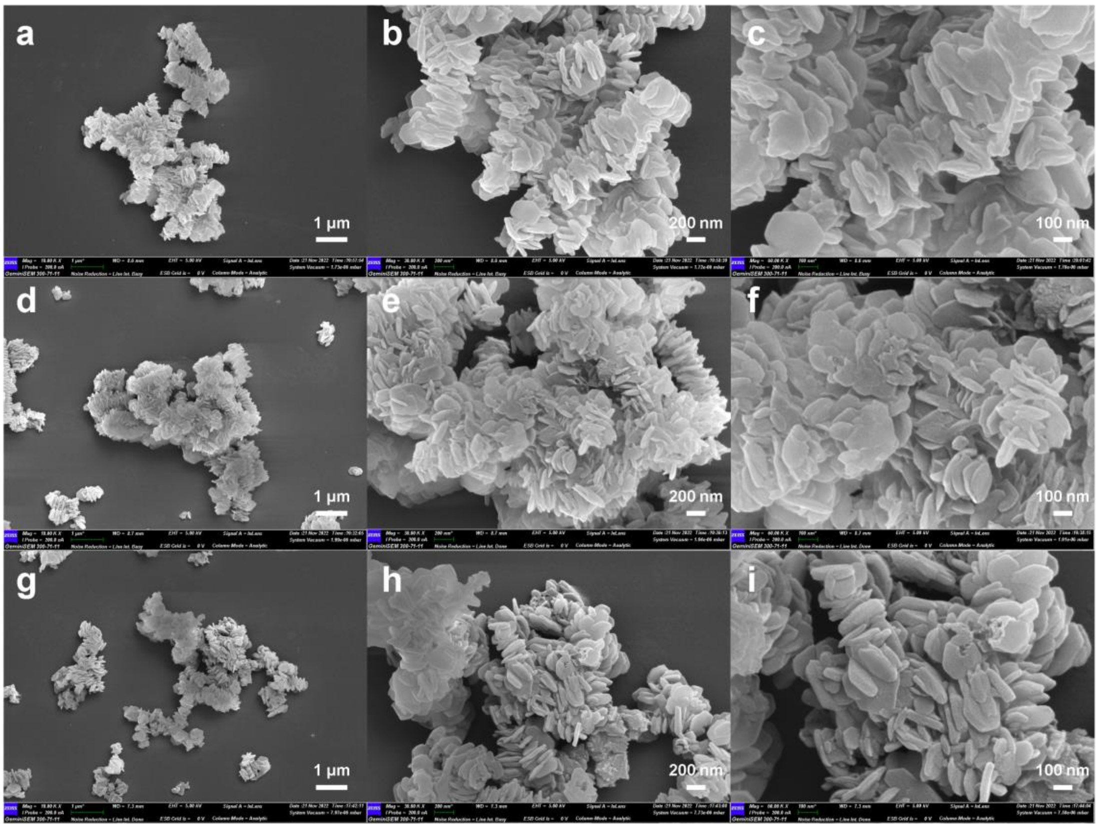

Figure S20. SEM images of **Im-COF-1** (a–c), **Quin-COF-1** (d–f), and **Pd@Quin-COF-1** (g–i) in 10×, 30×, and 60× magnification.

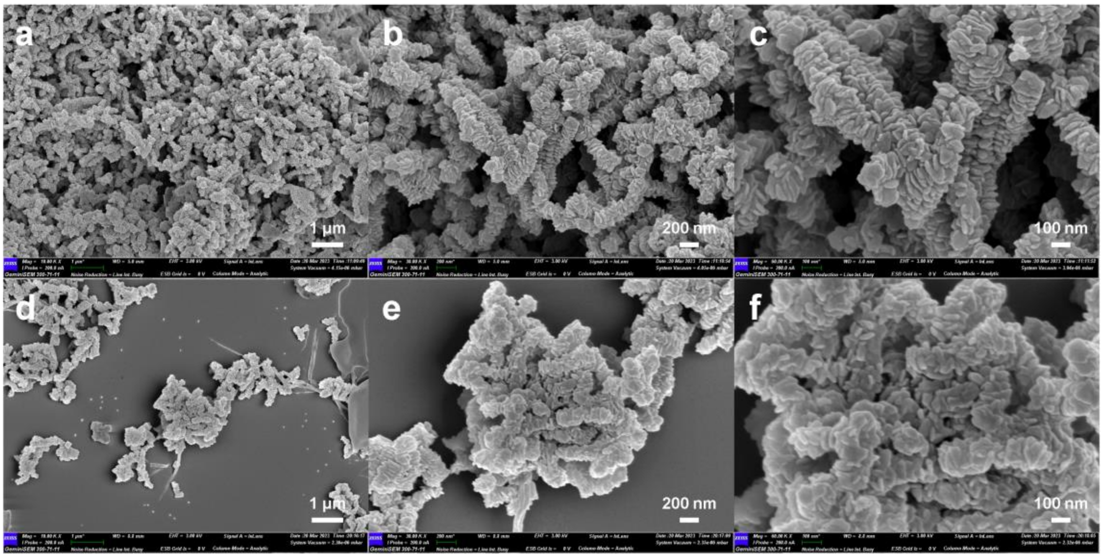

Figure S21. SEM images of **Im-COF-2** (a–c), and **Pd@Im-COF-2** (d–f) in 10×, 30×, and 60× magnification.

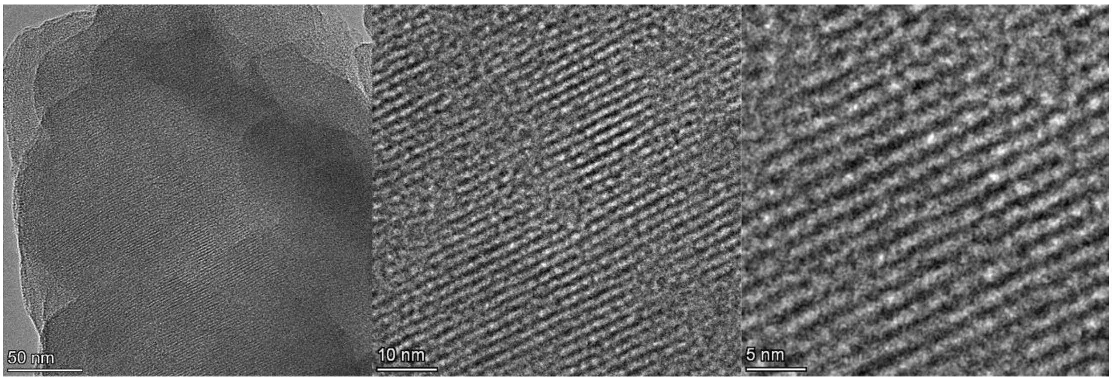

Figure S22. HR-TEM image of **Im-COF-1**.

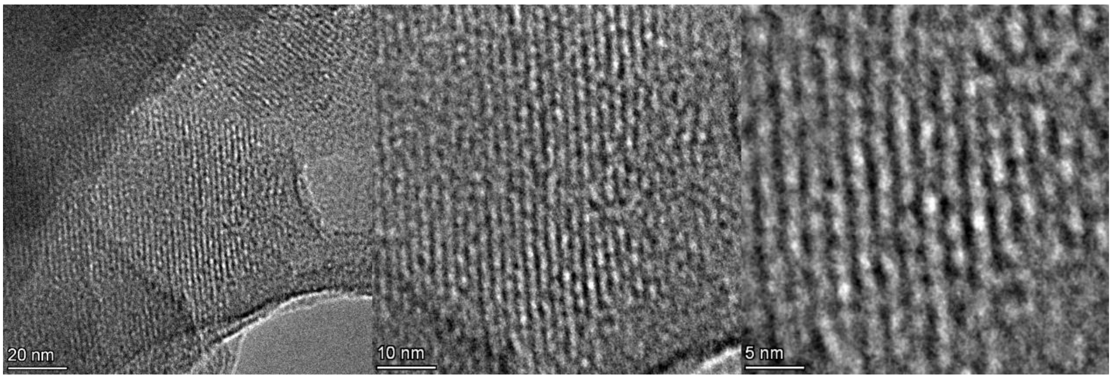

Figure S23. HR-TEM image of **Quin-COF-1**.

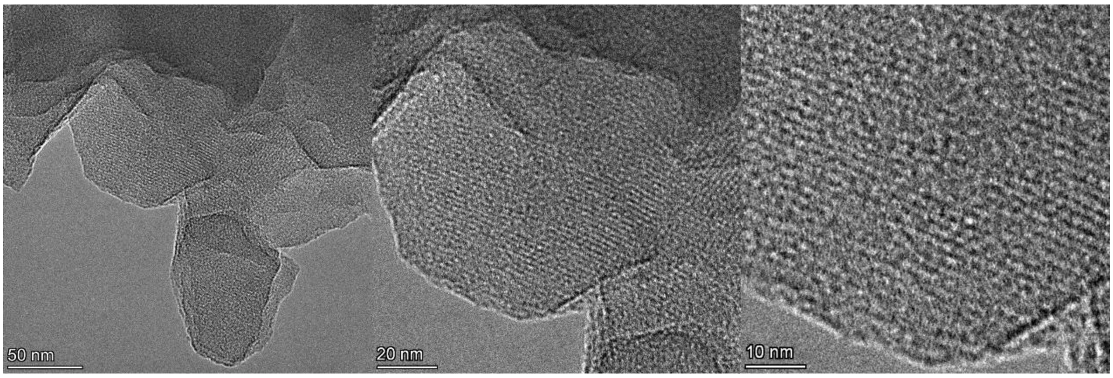

Figure S24. HR-TEM image of **Pd@Quin-COF-1**.

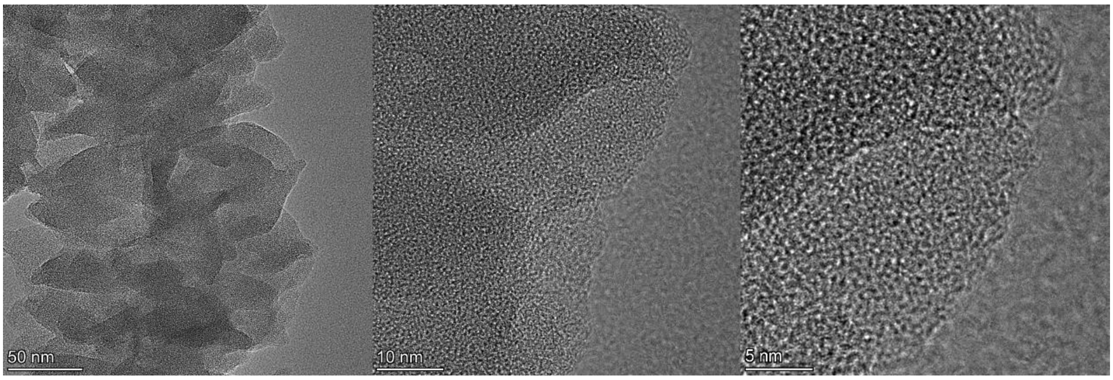

Figure S25. HR-TEM image of **Im-COF-2**.

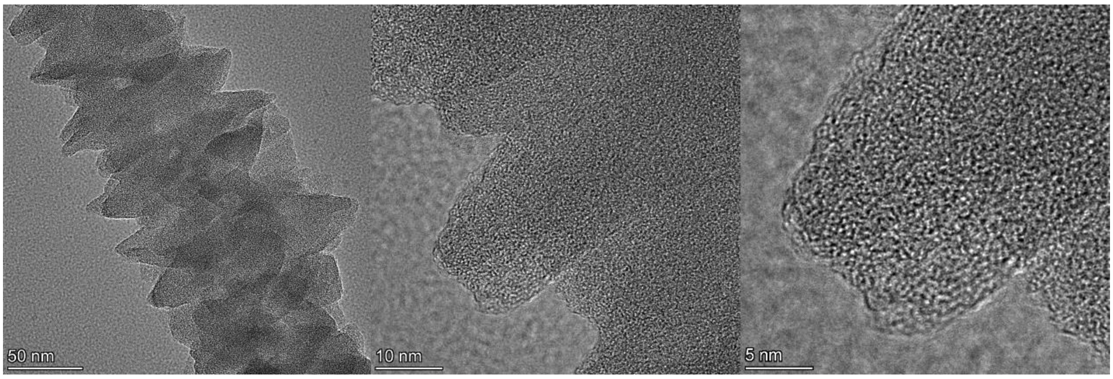

Figure S26. HR-TEM image of **Pd@Im-COF-2**.

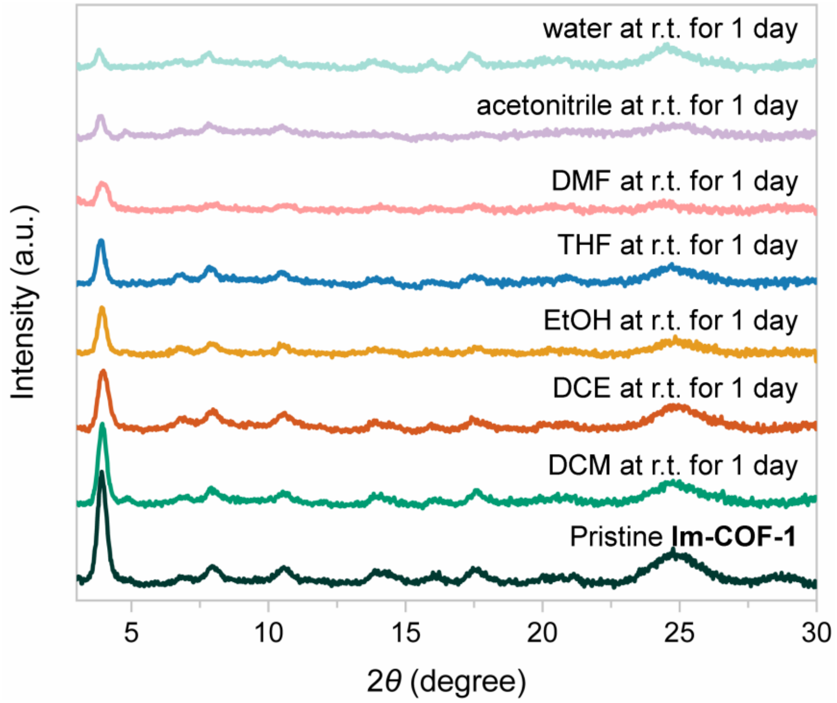

Figure S27. PXRD patterns of **Im-COF-1** after treatment with different solvents for 1 day.

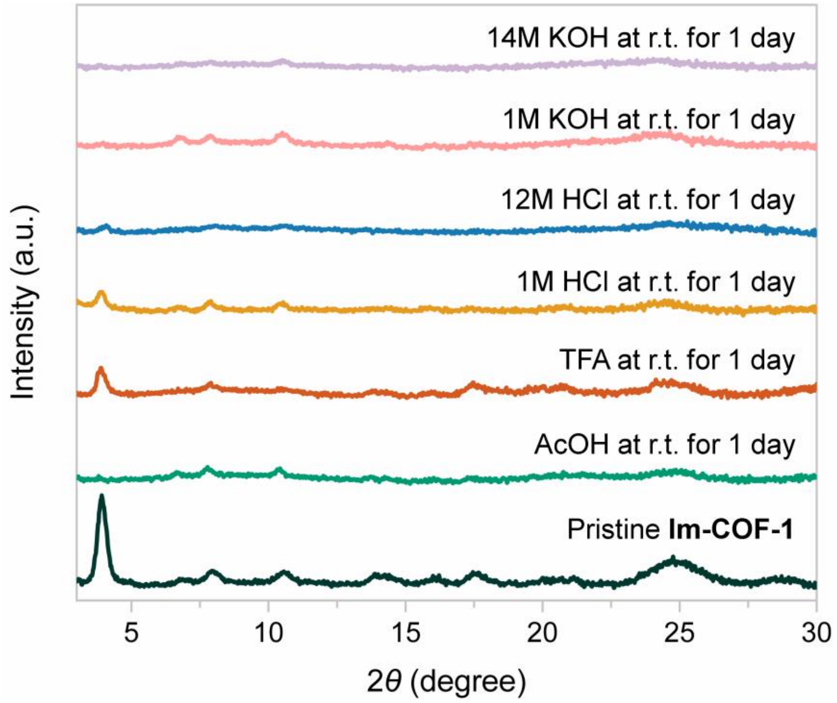

Figure S28. PXRD patterns of **Im-COF-1** after treatment harsh chemical conditions for 1 day.

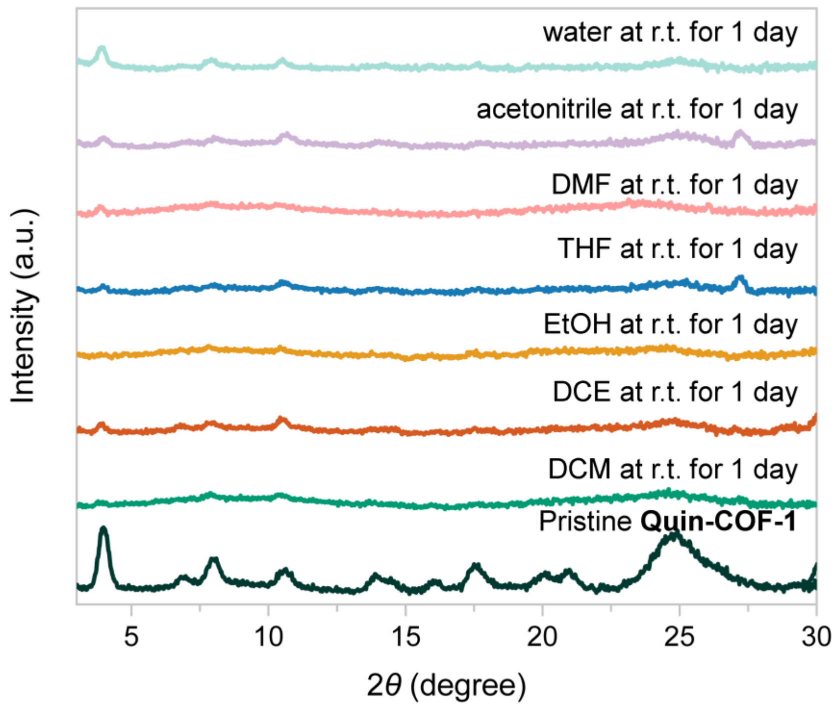

Figure S29. PXRD patterns of **Quin-COF-1** after treatment with different solvents for 1 day.

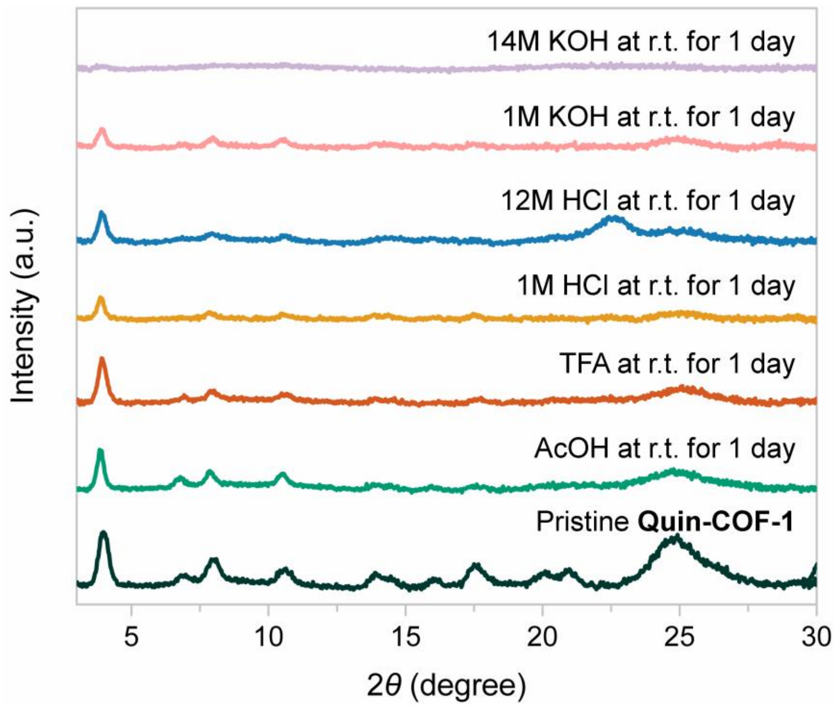

Figure S30. PXRD patterns of **Quin-COF-1** after treatment under harsh chemical conditions for 1 day.

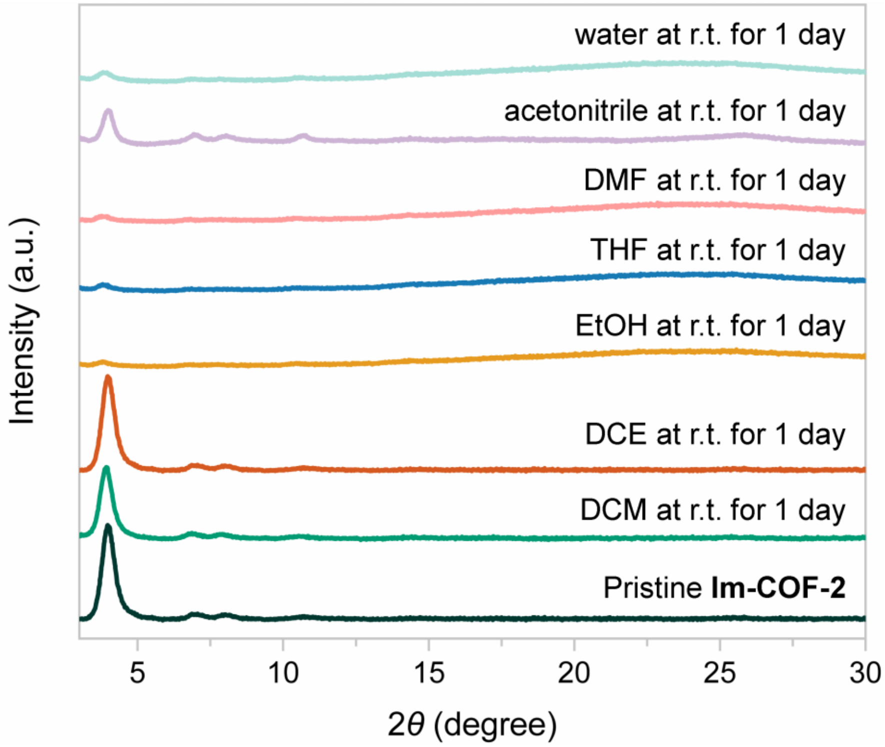

Figure S31. PXRD patterns of **Im-COF-2** after treatment with different solvents for 1 day.

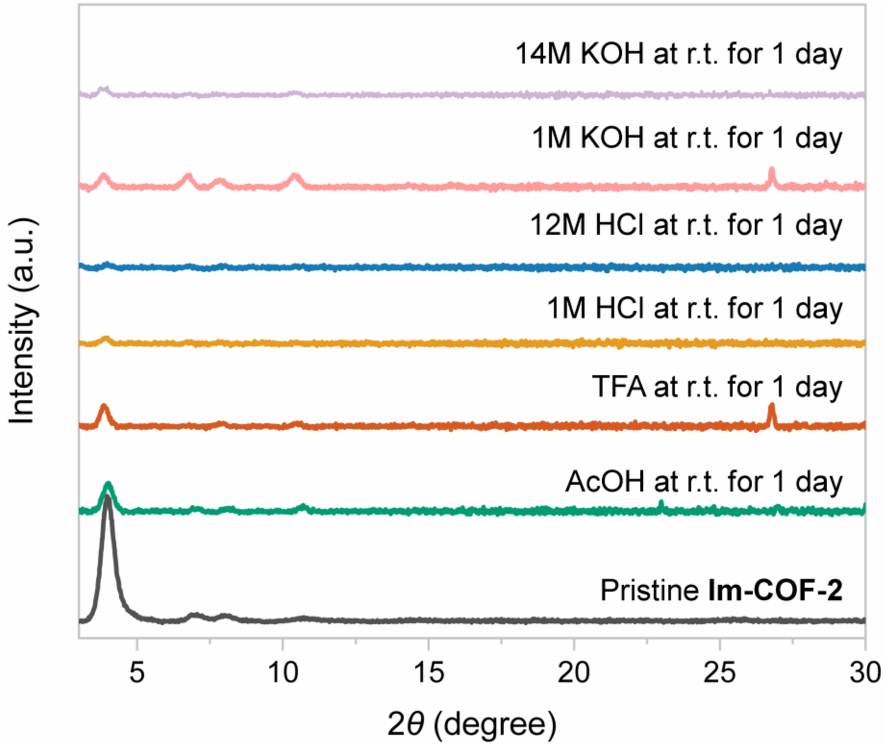

Figure S32. PXRD patterns of **Imine-COF-2** after treatment under harsh chemical conditions for 1 day.

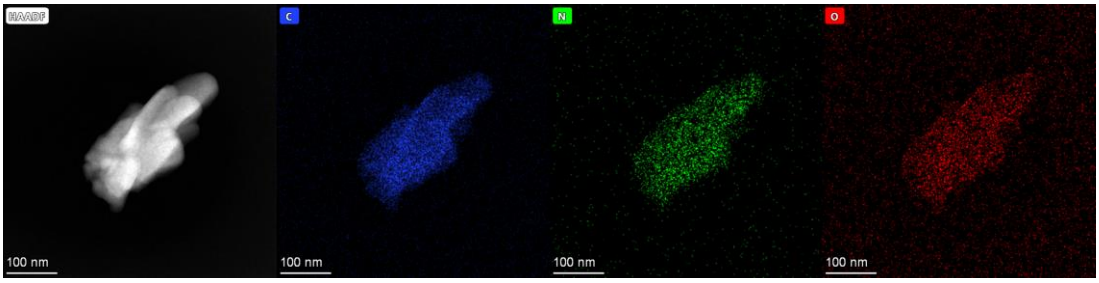

Figure S33. HADDF image and EDS mapping image of **Im-COF-1**. EDS mapping images of C, N, and O are represented in blue, green, and red.

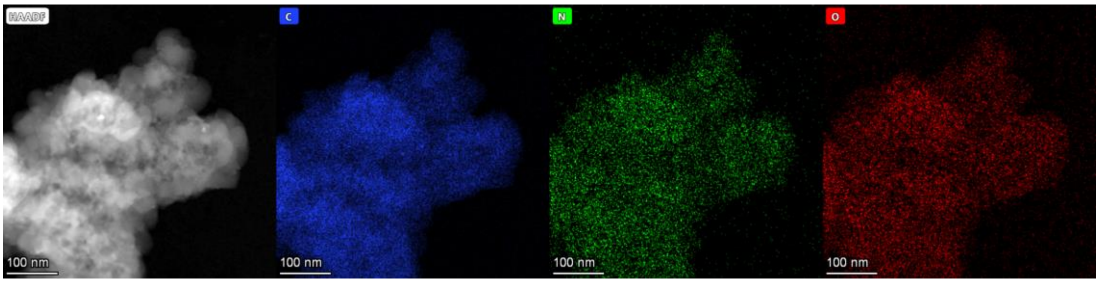

Figure S34. HADDF image and EDS mapping image of **Quin-COF-1**. EDS mapping images of C, N, and O are represented in blue, green, and red.

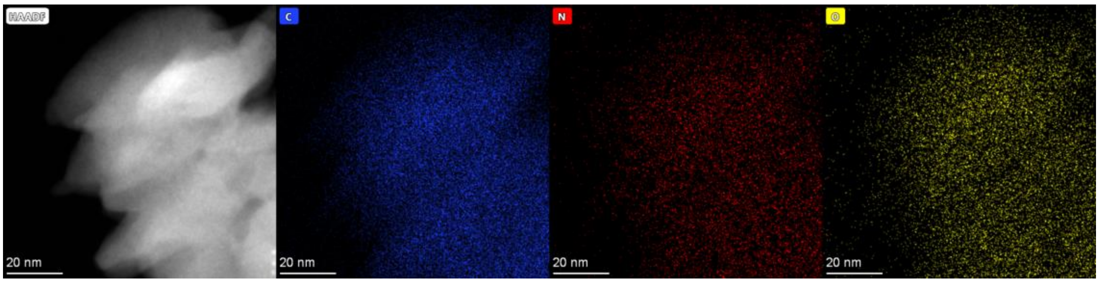

Figure S35. HADDF image and EDS mapping image of **Im-COF-2**. EDS mapping images of C, N, and O are represented in blue, red, and yellow.

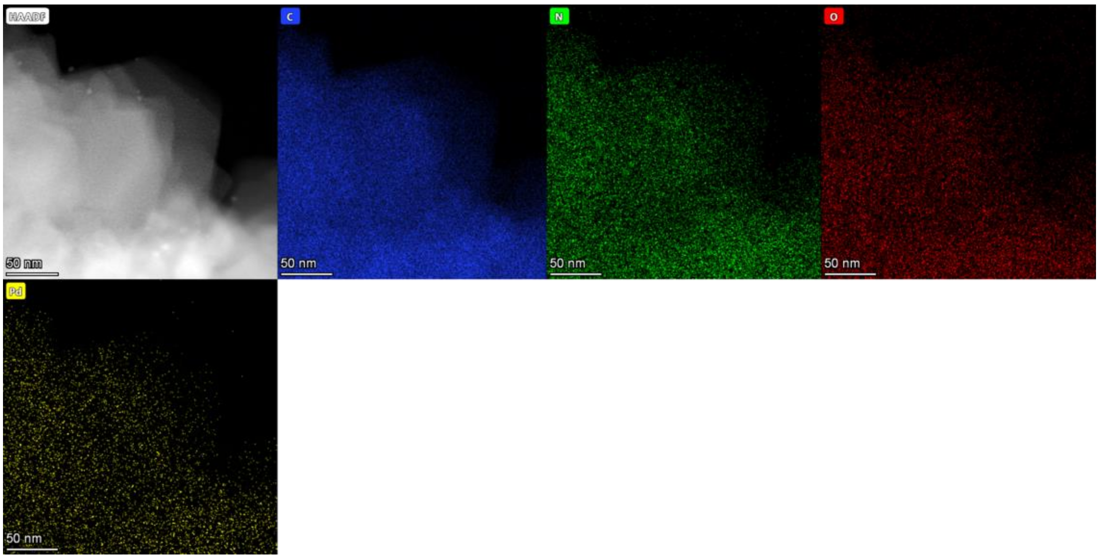

Figure S36. HADDF image and EDS mapping image of **Pd@Im-COF-2**. EDS mapping images of C, N, O, and Pd are represented in blue, green, red, and yellow.

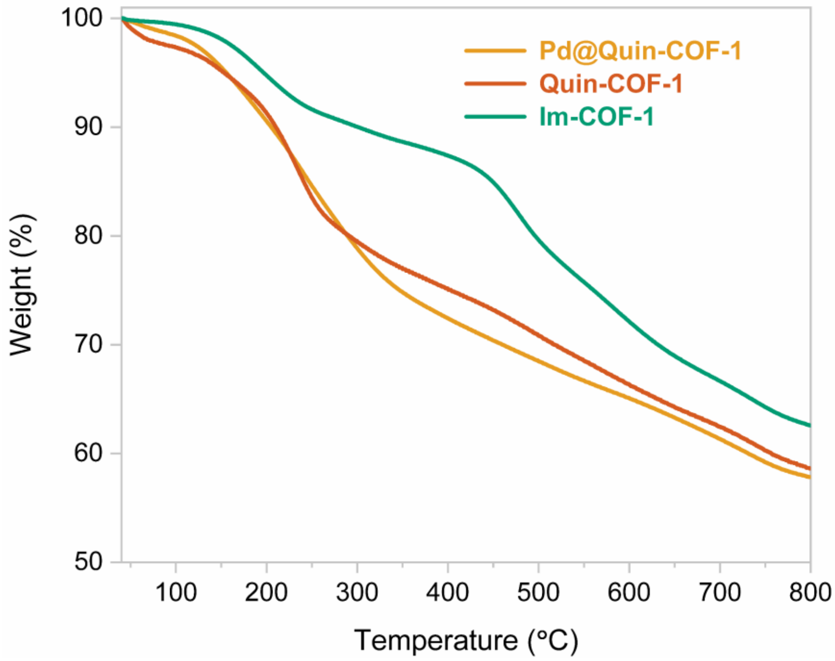

Figure S37. TGA plots of **Im-COF-1**, **Quin-COF-1**, and **Pd@Quin-COF-1** under N_2 atmosphere.

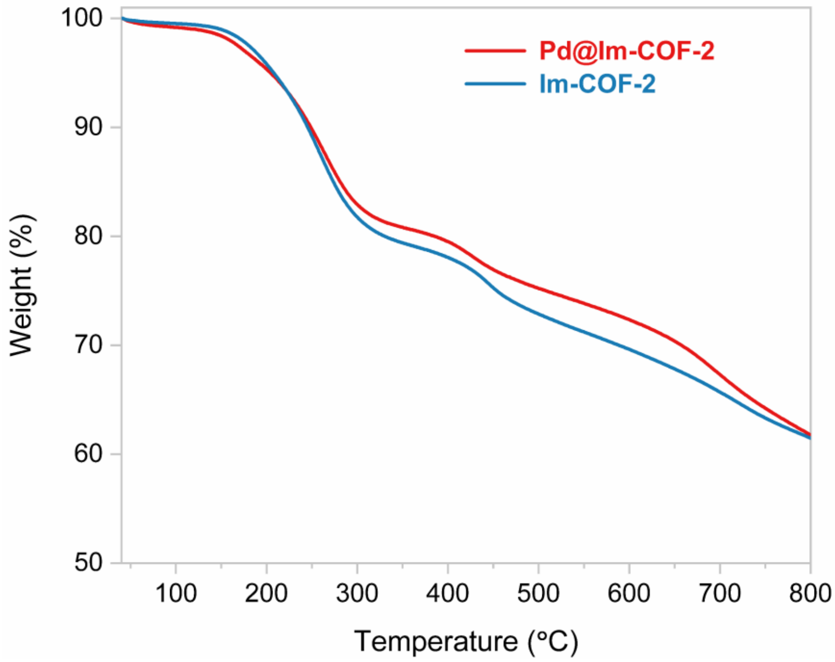

Figure S38. TGA plots of **Im-COF-2** and **Pd@Im-COF-2** under N_2 atmosphere.

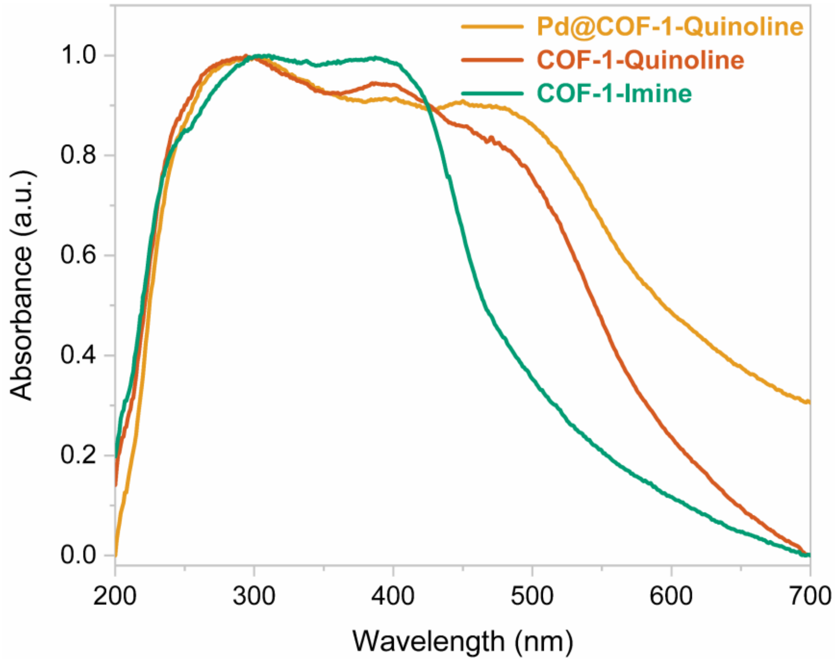

Figure S39. Solid-state absorption spectra for **Im-COF-1**, **Quin-COF-1**, and **Pd@Quin-COF-1**.

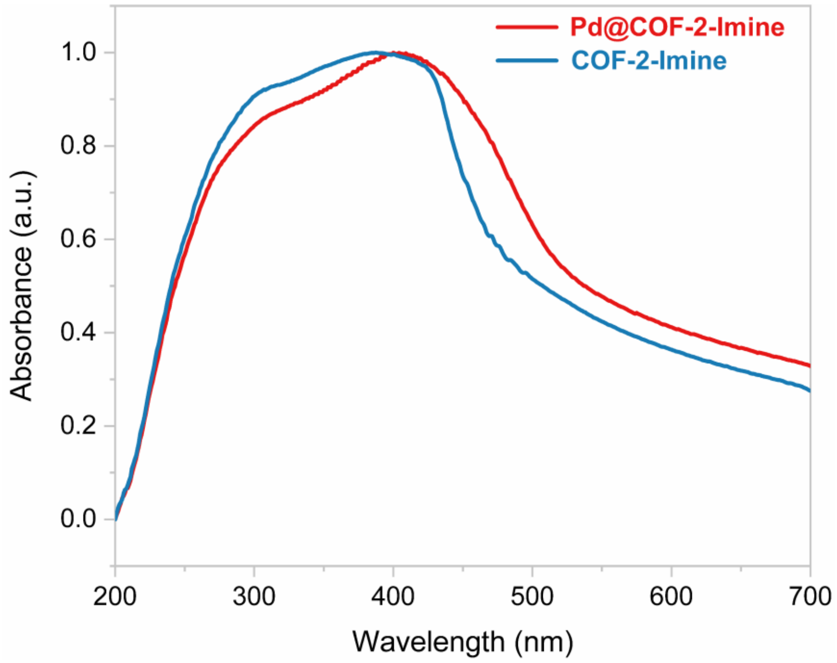

Figure S40. Solid-state absorption spectra for **Im-COF-2** and **Pd@Im-COF-2**.

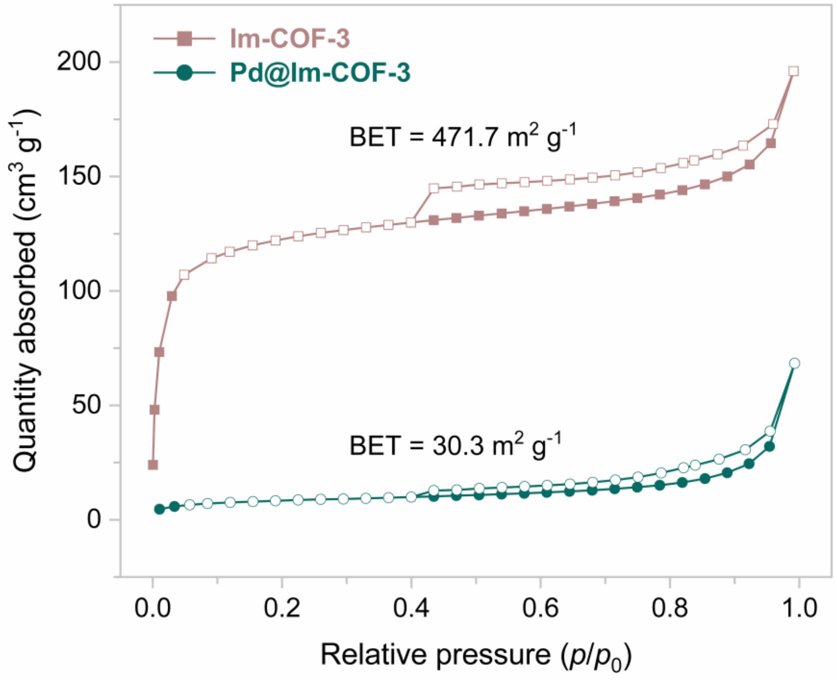

Figure S41. N_2 adsorption/desorption isotherms of **Im-COF-3**, and **Pd@Im-COF-3** recorded at 77 K.

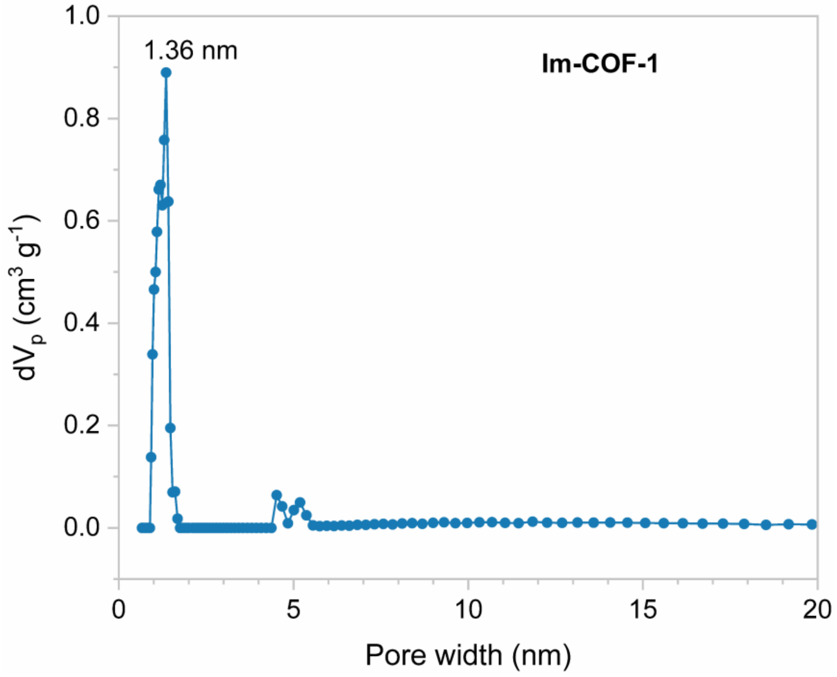

Figure S42. Pore size distribution profiles of **Im-COF-1**. The pores at around 5 nm could be generated by **Im-COF-1**'s partial structural collapse during N_2 adsorption/desorption isotherm measurements.

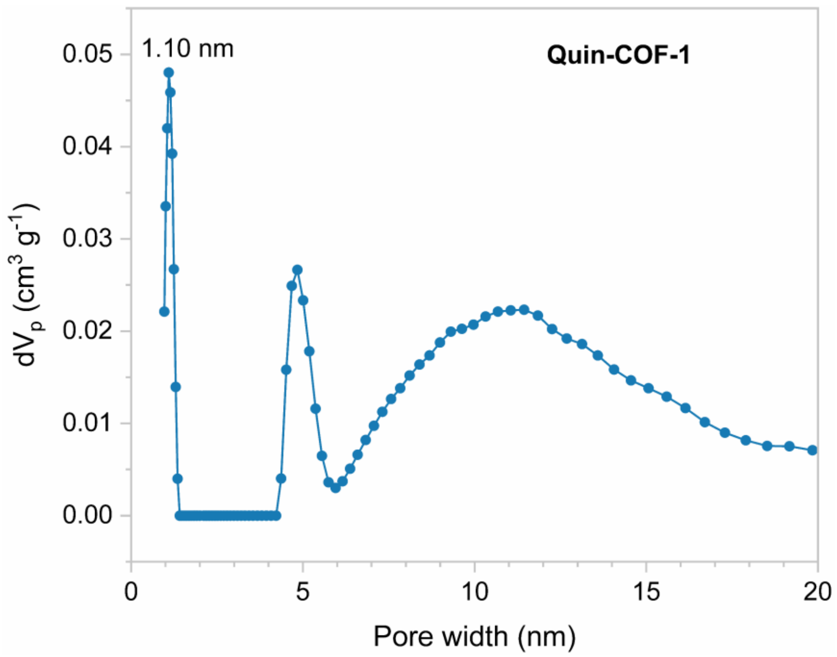

Figure S43. Pore size distribution profiles of **Quin-COF-1**. The pore at around 5 nm could be generated by **Quin-COF-1**'s partial structural collapse during N_2 adsorption/desorption isotherm measurements.

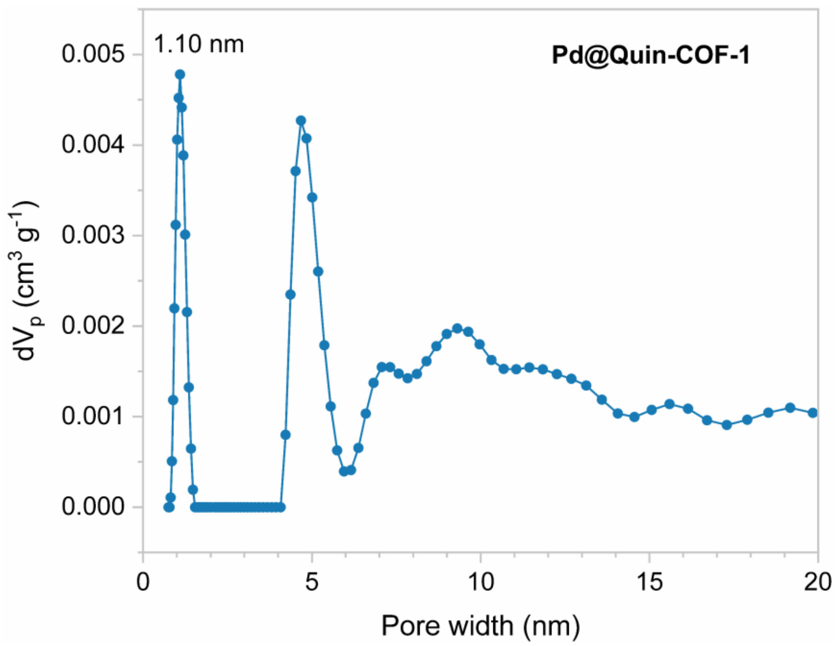

Figure S44. Pore size distribution profiles of **Pd@Quin-COF-1**. The pore at around 5 nm could be generated by **Pd@Quin-COF-1**'s partial structural collapse during N_2 adsorption/desorption isotherm measurements.

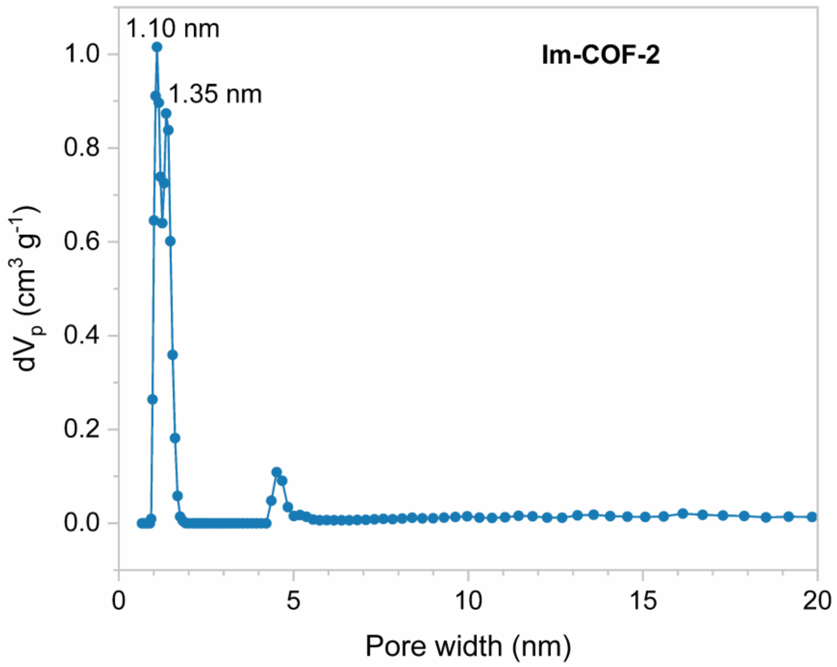

Figure S45. Pore size distribution profiles of **Im-COF-2**. The pores at around 5 nm could be generated by **Im-COF-2**'s partial structural collapse during N_2 adsorption/desorption isotherm measurements.

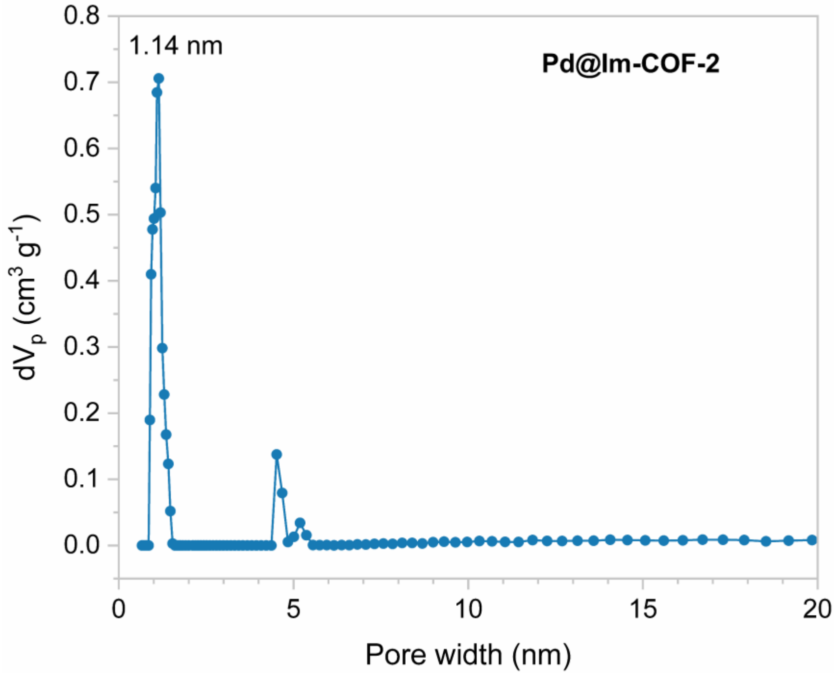

Figure S46. Pore size distribution profiles of **Pd@Im-COF-2**. The pores at around 5 nm could be generated by **Pd@Im-COF-2**'s partial structural collapse during N_2 adsorption/desorption isotherm measurements.

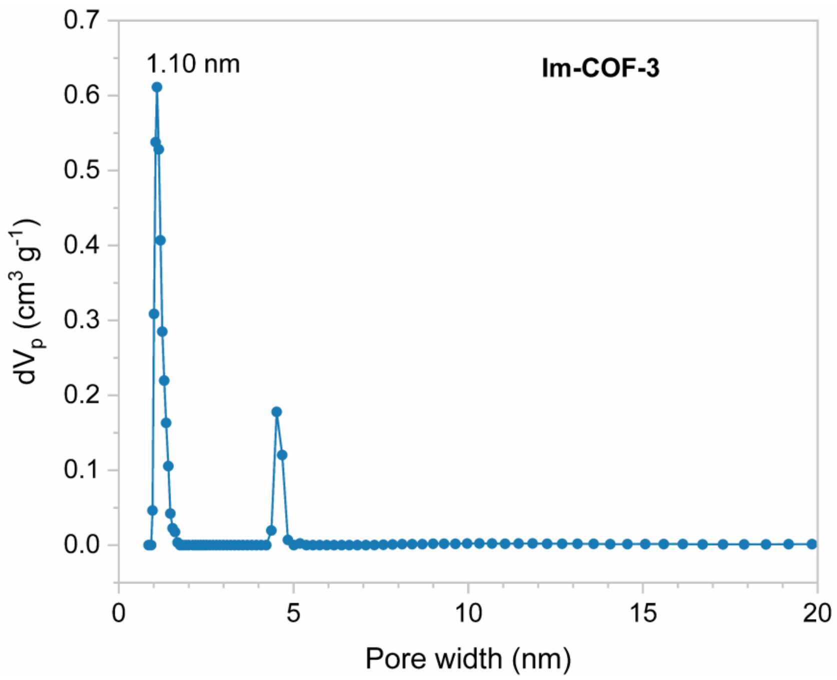

Figure S47. Pore size distribution profiles of **Im-COF-3**. The pore at around 5 nm could be generated by **Im-COF-3**'s partial structural collapse during N_2 adsorption/desorption isotherm measurements.

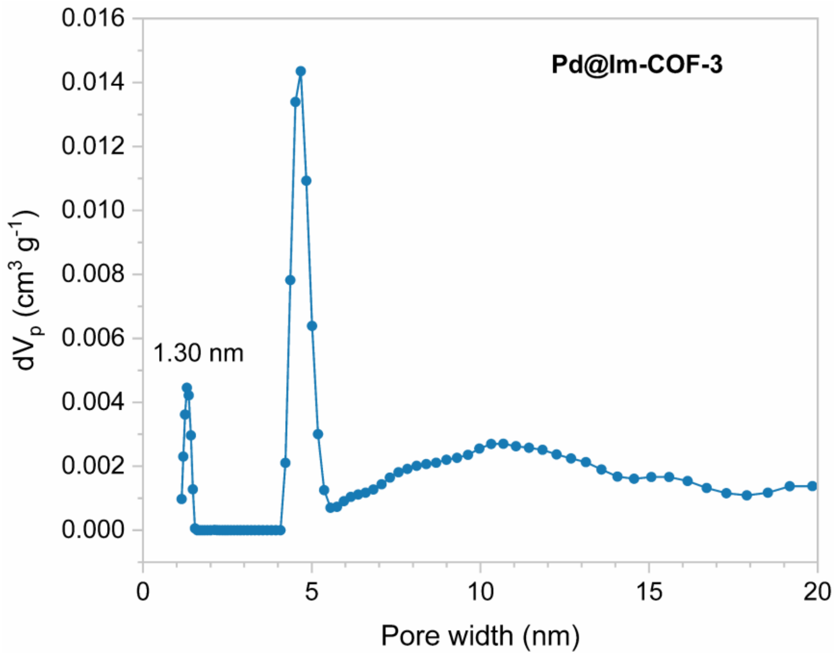

Figure S48. Pore size distribution profiles of **Pd@Im-COF-3**. The pore at around 5 nm could be generated by **Pd@Im-COF-3**'s partial structural collapse during N_2 adsorption/desorption isotherm measurements.

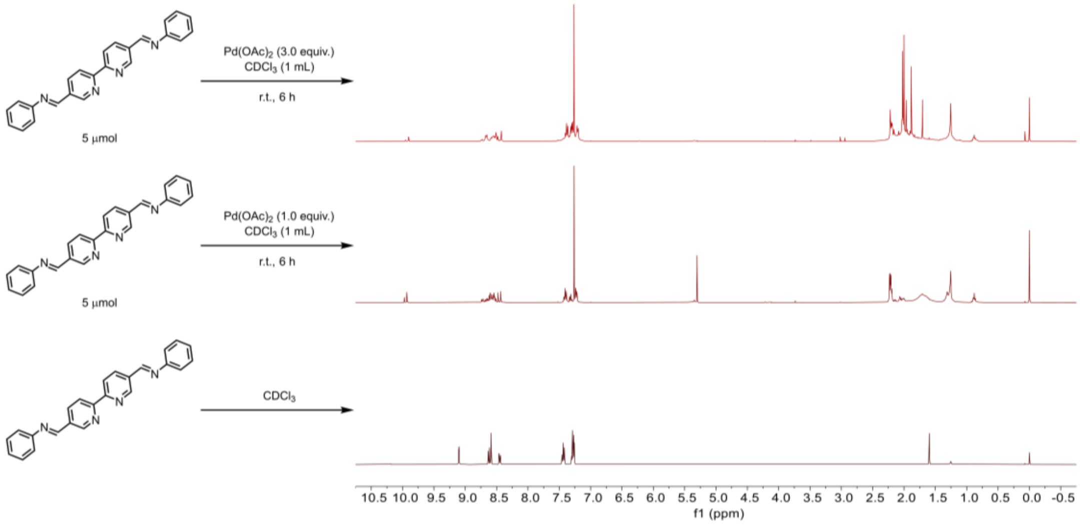

Figure S49. ^{1}H NMR spectra for the coordination of (1*E*,1'*E*)-1,1'-([2,2'-bipyridine]-5,5'-diyl)bis(*N*-phenylmethanimine) with $Pd(OAc)_2$ at different ratios.

5.2 DFT calculation

All DFT calculations were carried out using the CP2K code.[7] All calculations employed a mixed Gaussian and planewave basis sets. Core electrons were represented with norm-conserving Goedecker-Teter-Hutter pseudopotentials,[8-10] and the valence electron wavefunction was expanded in a double-zeta basis set with polarization functions[11] along with an auxiliary plane wave basis set with an energy cutoff of 360 eV. The generalized gradient approximation exchange-correlation functional of Perdew, Burke, and Enzerhof (PBE)[12] was used. Each configuration was optimized with the Broyden-Fletcher-Goldfarb-Shanno (BGFS) algorithm with SCF convergence criteria of 1.0×10^{-6} au. To compensate the long-range van der Waals dispersion interaction, the DFT-D3 scheme[13] with an empirical damped potential term was added into the energies obtained from exchange-correlation functional in all calculations.

The energy difference of the C–H cleavage process can be calculated using the following equation:

$$\Delta E_{CH} = E_{\mathrm{Pd(OAc)@COF}} + E_{\mathrm{HOAc}} - E_{\mathrm{COF}} - E_{\mathrm{Pd(OAc)2}} \qquad \text{(S1)}$$

In Eq. S1, $E_{\mathrm{Pd(OAc)@COF}}$ and E_{HOAc} represent the total energies of the COF material after cyclopalladation and acetic acid molecule, respectively.

The adsorption energy between $Pd(OAc)_2$ and the COF can be calculated using the following equation:

$$\Delta E_{ads} = E_{\mathrm{Pd(OAc)2@COF}} - E_{\mathrm{COF}} - E_{\mathrm{Pd(OAc)2}} \qquad \text{(S2)}$$

In Eq. S2, $E_{\mathrm{Pd(OAc)2@COF}}$ and E_{COF} represent the total energies of the COF material with and without the adsorption of adsorbate $Pd(OAc)_2$, respectively. $E_{\mathrm{Pd(OAc)2}}$ is the total energy of adsorbate $Pd(OAc)_2$. According to this equation, a negative adsorption energy corresponds to a stable adsorption structure.

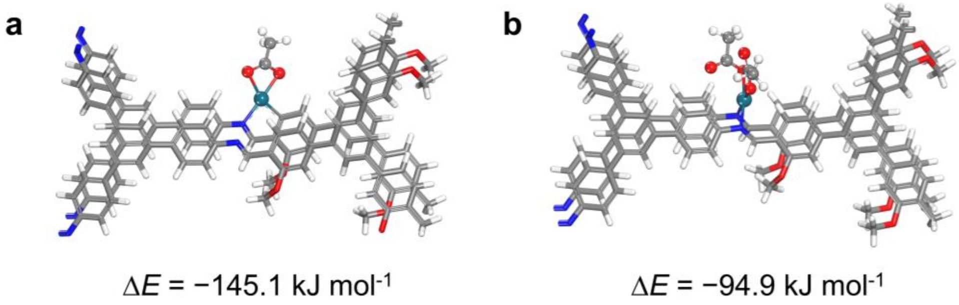

Figure S50. (a) Adsorption energy of cyclopalladation on **Im-COF-2**. (b) Adsorption energy of $Pd(OAc)_2$ in the interlayers of **Im-COF-2**.

5.3 Preparation of MOF-supported palladium catalysts

Synthesis of UiO-67-bpy:[14] $ZrCl_4$ (120 mg, 0.51 mmol) and 2,2'-bipyridine-5,5'-dicarboxylic acid (125 mg, 0.51 mmol) were dissolved in 30 mL of *N*,*N*-dimethylformamide (DMF) by sonication in a 100-mL Schlenk tube. Glacial acetic acid (0.56 mL) was added as a modulator. The tube was sealed and placed in an oven at 120 °C for 3 d. After cooling to ambient temperature, the solid MOF was collected via filtration and washed with DMF and ethanol. Finally, **UiO-67-bpy** was activated at 120 °C for 12 h prior to experimental use (144 mg, 80% yield).

Synthesis of MOF-253-bpy:[15] $AlCl_3{\cdot}6H_2O$ (151 mg, 0.625 mmol) and 2,2'-bipyridine-5,5'-dicarboxylic acid (153 mg, 0.625 mmol) were dissolved in 10 mL of DMF in a 20-mL vial. The vial was placed in an oven at 120 °C for 3 d. After cooling to ambient temperature, **MOF-253-bpy** was collected as a white solid via filtration and thoroughly washed with DMF and methanol. Finally, **MOF-253-bpy** was activated at 120 °C for 12 h prior to experimental use (127 mg, 72% yield).

Synthesis of Pd@UiO-67-bpy and Pd@MOF-253-bpy: The preparation procedures of **Pd@UiO-67-bpy** and **Pd@MOF-253-bpy** were almost the same as those of **Pd@Im-COFs**. Briefly, to the solution of palladium acetate (45 mg, 0.2 mmol) in DCM (10 mL), **UiO-67-bpy** or **MOF-253-bpy** (100 mg) was added. The mixture was stirred slowly at room temperature for 48 h. After reaction completion, the resulting solid was isolated by filtration and washed with a large amount of DCM, then dried at 70 °C for 12 h to afford **Pd@UiO-67-bpy** or **Pd@MOF-253-bpy**. The palladium loading was confirmed by ICP-AES to be 0.7 wt% for **Pd@UiO-67-bpy** and 2.6 wt% for **Pd@MOF-253-bpy**, respectively.

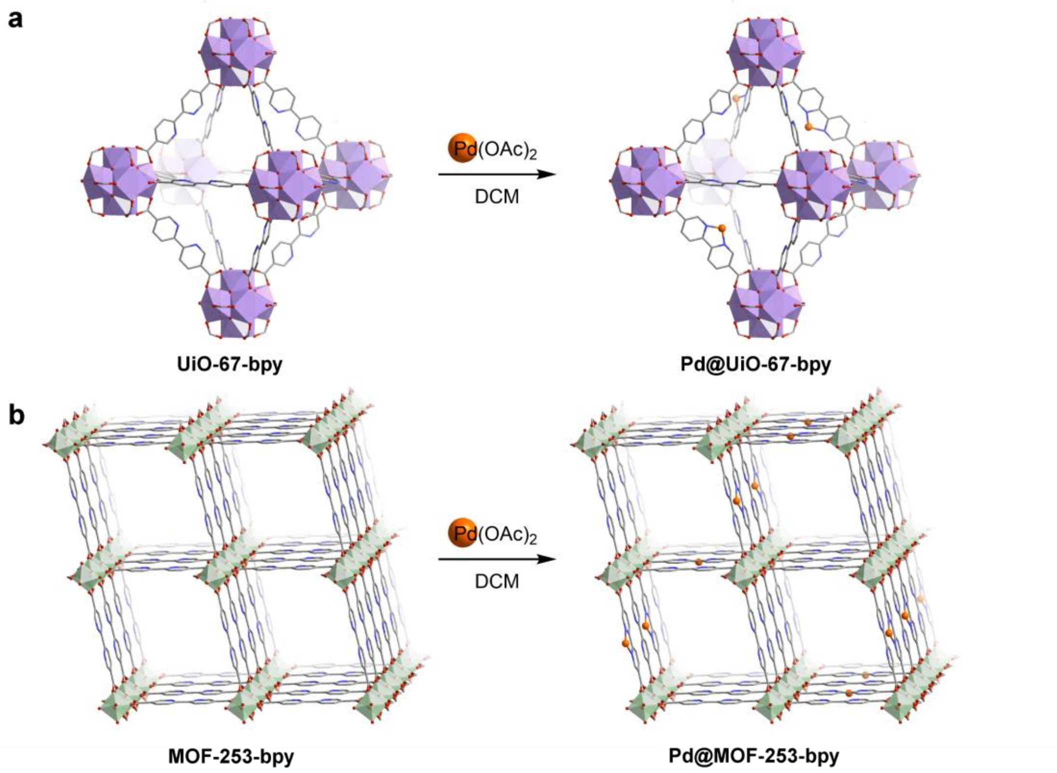

Figure S51. Post-synthetic metalation for the synthesis of **Pd@UiO-67-bpy** (a) and **Pd@MOF-253-bpy** (b).

6 Catalytic activity test of heterogeneous palladium catalysts

6.1 Synthesis of diaryliodonium salts

The diaryliodonium salts diphenyliodonium tetrafluoroborate (**2a**), di-*o*-tolyliodonium tetrafluoroborate (**2b**), bis(4-(methoxycarbonyl)phenyl)iodonium tetrafluoroborate (**2e**), bis(4-fluorophenyl)iodonium tetrafluoroborate (**2f**), bis(4-chlorophenyl)iodonium tetrafluoroborate (**2g**), bis(2-chlorophenyl)iodonium tetrafluoroborate (**2h**), and bis(4-bromophenyl)iodonium tetrafluoroborate (**2i**) were synthesized by following a modified literature procedure (General Procedure A).[16] The diaryliodonium salts di(naphthalen-1-yl)iodonium tetrafluoroborate (**2c**) was synthesized according to a literature procedure.[17] The diaryliodonium salts bis(4-methoxyphenyl)iodonium tetrafluoroborate (**2d**) was synthesized according to a literature procedure.[18]

General Procedure A:

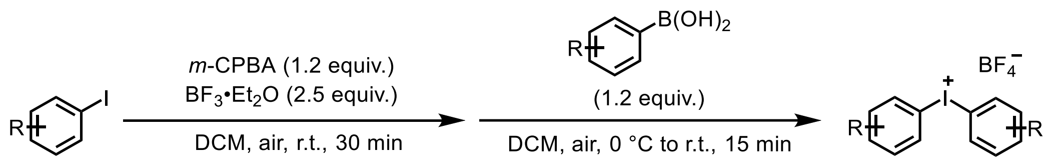

To a solution of *m*-chloroperoxybenzoic acid (85% active oxidant, 1.2 g, 6.0 mmol) and DCM (20 mL), iodoarene derivative (5.0 mmol) and BF_3·Et_2O (1.54 mL, 12.5 mmol) were added slowly. After stirring at room temperature for 30 min, the reaction mixture was cooled to 0 °C and arylboronic acid (6.0 mmol) was added in small portions followed by stirring at room temperature for an additional 15 min. Upon completion of reaction, the reaction mixture was applied on a silica plug (20 g) and eluted with DCM (150 mL) followed by 5% methanol in DCM (300 mL). The latter solution was concentrated followed by addition of diethyl ether (30 mL) to indue precipitation of the diaryliodonium salt. The resulting solution was stirred at room temperature for 15 min and the ether phase was decanted. The solid product was washed twice with diethyl ether (2 × 25 mL). Finally, the diethyl ether layer was decanted, and the solid was dried under vacuum to afford the target diaryliodonium salt product **2a**, **2b**, and **2e**–**2i**.

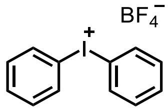

Diphenyliodonium tetrafluoroborate (2a). Following the General Procedure A, **2a** was synthesized as a white solid (1.1 g, 60% yield).

^{1}H NMR (400 MHz, DMSO-*d*$_6$) δ 8.28–8.21 (m, 4H), 7.67 (t, *J* = 7.2 Hz, 2H), 7.53 (t, *J* = 7.6 Hz, 4H).

^{13}C NMR (100 MHz, DMSO-*d*$_6$) δ 135.2, 132.1, 131.8, 116.5.

^{19}F NMR (376 MHz, DMSO-*d*$_6$) δ -148.15, -148.21.

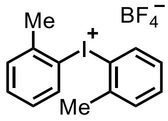

Di-*o*-tolyliodonium tetrafluoroborate (2b). Following the General Procedure A, **2b** was synthesized as a white solid (1.4 g, 72% yield).

^{1}H NMR (400 MHz, DMSO-*d*$_6$) δ 8.36–8.26 (m, 2H), 7.62–7.52 (m, 4H), 7.34–7.26 (m, 2H), 2.61 (s, 6H).

^{13}C NMR (100 MHz, DMSO-*d*$_6$) δ 140.6, 137.2, 132.8, 131.6, 129.3, 120.6, 25.0

^{19}F NMR (376 MHz, DMSO-*d*$_6$) δ -148.16, -148.22.

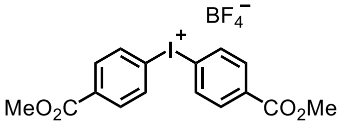

Bis(4-(methoxycarbonyl)phenyl)iodonium tetrafluoroborate (2e). Following the General Procedure A, **2e** was synthesized as a pale brown solid (1.6 g, 66% yield).

^{1}H NMR (400 MHz, DMSO-*d*$_6$) δ 8.40 (d, *J* = 8.4 Hz, 4H), 8.04 (d, *J* = 8.0 Hz, 4H), 3.86 (s, 6H).

^{13}C NMR (100 MHz, DMSO-*d*$_6$) δ 165.1, 135.7, 132.8, 132.1, 121.4, 52.8.

^{19}F NMR (376 MHz, DMSO-*d*$_6$) δ -148.15, -148.20.

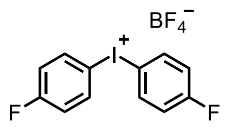

Bis(4-fluorophenyl)iodonium tetrafluoroborate (2f). Following the General Procedure A, **2f** was synthesized as a white solid (0.97 g, 48% yield).

^{1}H NMR (400 MHz, DMSO-*d*$_6$) δ 8.36–8.27 (m, 4H), 7.54–7.26 (m, 4H).

^{13}C NMR (100 MHz, DMSO-*d*$_6$) δ 164.0 (d, *J* = 251.4 Hz), 138.0 (d, *J* = 9.0 Hz), 119.3 (d, *J* = 22.8 Hz), 111.2 (d, *J* = 3.2 Hz).

^{19}F NMR (376 MHz, DMSO-*d*$_6$) δ -106.6, -148.15, -148.21.

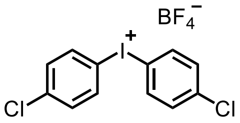

Bis(4-chlorophenyl)iodonium tetrafluoroborate (2g). Following the General Procedure A, **2g** was synthesized as a white solid (1.4 g, 65% yield).

^{1}H NMR (400 MHz, DMSO-*d*$_6$) δ 8.26 (d, *J* = 8.8 Hz, 4H), 7.63 (d, *J* = 8.8 Hz, 4H).

^{13}C NMR (100 MHz, DMSO-*d*$_6$) δ 137.5, 137.0, 131.9, 114.8.

^{19}F NMR (376 MHz, DMSO-*d*$_6$) δ -148.08, -148.13.

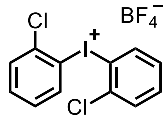

Bis(2-chlorophenyl)iodonium tetrafluoroborate (2h). Following the General Procedure A, **2h** was synthesized as a white solid (1.5 g, 68% yield).

^{1}H NMR (400 MHz, DMSO-*d*$_6$) δ 8.53 (dd, *J* = 8.0, 1.4 Hz, 2H), 7.84 (dd, *J* = 8.0, 1.4 Hz, 2H), 7.70 (m, 2H), 7.50 (m, 2H).

^{13}C NMR (100 MHz, DMSO-*d*$_6$) δ 139.0, 136.0, 134.8, 130.5, 130.2, 119.6.

^{19}F NMR (376 MHz, DMSO-*d*$_6$) δ -148.15, -148.20.

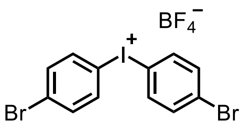

Bis(4-bromophenyl)iodonium tetrafluoroborate (2i). Following the General Procedure A, **2i** was synthesized as a white solid (1.7 g, 63% yield).

^{1}H NMR (400 MHz, DMSO-d_6) δ 8.17 (d, J = 8.8 Hz, 4H), 7.76 (d, J = 8.4 Hz, 4H).

^{13}C NMR (100 MHz, DMSO-d_6) δ 137.1, 134.7, 126.4, 115.4.

^{19}F NMR (376 MHz, DMSO-d_6) δ -148.16, -148.21.

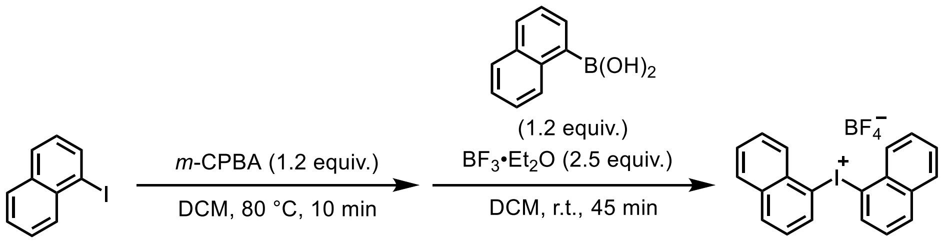

To a solution of *m*-chloroperoxybenzoic acid (85% active oxidant, 1.2 g, 6.0 mmol) and DCM (20 mL), 1-iodonaphthalene (1.3 g, 5.0 mmol) were added slowly. After stirring at 80 °C temperature for 10 min, the reaction mixture was returned to room temperature. BF_3•Et_2O (1.54 mL, 12.5 mmol) and naphthalen-1-ylboronic acid (1.0 g, 6.0 mmol) was added in small portions followed by stirring at room temperature for an additional 15 min. Upon completion of reaction, the reaction mixture was applied on a silica plug (20 g) and eluted with DCM (150 mL) followed by 5% methanol in DCM (300 mL). The latter solution was concentrated followed by addition of diethyl ether (30 mL) to indue precipitation of the diaryliodonium salt. The resulting solution was stirred at room temperature for 15 min and the ether phase was decanted. The solid product was washed twice with diethyl ether (2 × 25 mL). Finally, the diethyl ether layer was decanted, and the solid was dried under vacuum to afford the target diaryliodonium salt product **2c**.

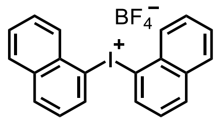

Di(naphthalen-1-yl)iodonium tetrafluoroborate (2c). Following the above procedure, **2c** was synthesized as a grey solid (0.84 g, 36% yield).

^{1}H NMR (400 MHz, DMSO-d_6) δ 8.88 (dd, J = 7.6, 1.0 Hz, 2H), 8.50 (d, J = 8.4 Hz, 2H), 8.21 (d, J = 8.0 Hz, 2H), 8.00 (d, J = 8.0 Hz, 2H), 7.81 (m, 2H), 7.68 (m, 2H), 7.57 (t, J = 7.6 Hz, 2H).

^{13}C NMR (100 MHz, DMSO-d_6) δ 137.4, 134.2, 133.4, 131.0, 129.8, 129.5, 128.9, 128.0, 127.5, 119.2.

^{19}F NMR (376 MHz, DMSO-d_6) δ -148.15, -148.21.

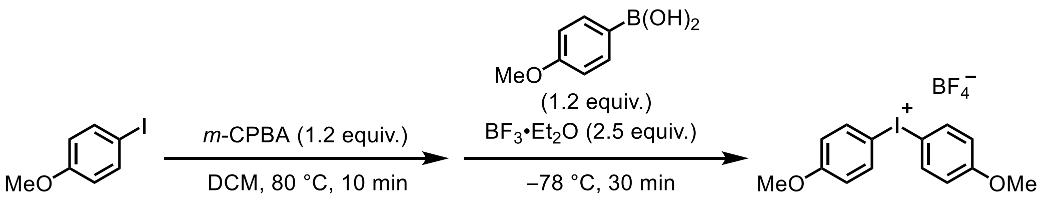

To a solution of *m*-chloroperoxybenzoic acid (85% active oxidant, 1.2 g, 6.0 mmol) and DCM (20 mL), 1-iodo-4-methoxybenzene (1.2 g, 5.0 mmol) were added slowly. After stirring at 80 °C temperature for 10 min, the reaction mixture was cooled to -78 °C in which BF_3•Et_2O (1.54 mL, 12.5 mmol) and (4-methoxyphenyl)boronic acid (0.91g, 6.0 mmol) were added in small portions. The reaction mixture was stirred at -78 °C for an additional 30 min. Upon completion of reaction, the reaction mixture was applied on a silica plug (20 g) and eluted with DCM (150 mL) followed by 5% methanol in DCM (300 mL). The latter solution was concentrated followed by addition of diethyl ether (30 mL) to indue precipitation of the diaryliodonium salt. The resulting solution was stirred at room temperature for 15 min and the ether phase was decanted. The solid product was washed twice with diethyl ether (2 × 25 mL). Finally, the diethyl ether layer was decanted, and the solid was dried under vacuum to afford the target diaryliodonium salt product **2d**.

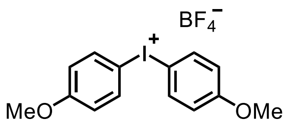

Bis(4-methoxyphenyl)iodonium tetrafluoroborate (2d). Following the above procedure, **2d** was synthesized as a grey solid (0.86 g, 40% yield).

^{1}H NMR (400 MHz, DMSO-*d*$_6$) δ 8.12 (d, *J* = 8.8 Hz, 4H), 7.05 (d, *J* = 9.2 Hz, 4H), 3.78 (s, 6H).

^{13}C NMR (100 MHz, DMSO-*d*$_6$) δ 161.8, 136.9, 117.3, 106.2, 55.7.

^{19}F NMR (376 MHz, DMSO-*d*$_6$) δ -148.16, -148.22.

6.2 Synthesis of indole substrates

Indole substrates 1,6-dimethyl-1*H*-indole (**1c**), 5-fluoro-1-methyl-1*H*-indole (**1h**), and 5-bromo-1-methyl-1*H*-indole (**1j**) were synthesized by following a modified literature procedure (General Procedure B).[19]

General Procedure B:

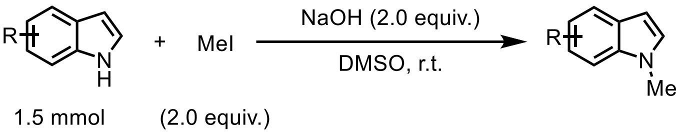

To a DMSO (10 mL) solution of the free indole (1.5 mmol, 1.0 equiv.) was added NaOH (2.0 equiv.) and methyl iodide (2.0 equiv.). The reaction was stirred at room temperature until completion as determined by TLC (typically less than 2 h). After reaction completion, water was then added (100 mL) followed by ethyl acetate (50 mL), and the organic phase was washed twice with water (100 mL). The organic phase was then dried with anhydrous Na_2SO_4, filtered on a short silica pad and the volatiles evaporated under reduced pressure. The products were usually used without further purification, but where the protecting indole were not pure, they were further purified using silica gel chromatography with hexanes/ethyl acetate (80:1, v/v) as eluent.

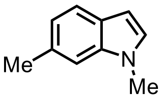

1,6-Dimethyl-1*H*-indole (1c). Following the General Procedure B, **1c** was synthesized as a white solid (0.20 g, 92% yield).

^{1}H NMR (400 MHz, $CDCl_3$) δ 7.49 (d, *J* = 8.0 Hz, 1H), 7.10 (s, 1H), 6.99–6.88 (m, 2H), 6.41 (d, *J* = 3.2 Hz, 1H), 3.70 (s, 3H), 2.49 (s, 3H).

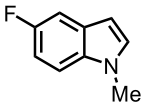

5-Fluoro-1-methyl-1*H*-indole (1h). Following the General Procedure B, **1h** was

synthesized as a white solid (0.21 g, 93% yield).

^{1}H NMR (400 MHz, $CDCl_3$) δ 7.29–7.24 (m, 1H), 7.22 (dd, *J* = 8.8, 4.4 Hz, 1H), 7.08 (d, *J* = 3.2 Hz, 1H), 6.96 (td, *J* = 9.0, 2.6 Hz, 1H), 6.43 (d, *J* = 3.2 Hz, 1H), 3.78 (s, 3H).

^{13}C NMR (100 MHz, $CDCl_3$) δ 157.8 (d, *J* = 233.8 Hz), 133.3, 130.3, 128.6 (d, *J* = 10.2 Hz), 109.9 (d, *J* = 19.6 Hz), 109.7 (d, *J* = 3.2 Hz), 105.5 (d, *J* = 23.4 Hz), 100.8 (d, *J* = 4.8 Hz), 33.1.

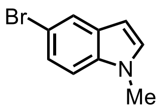

5-Bromo-1-methyl-1*H*-indole (1j). Following the General Procedure B, **1j** was synthesized as a white solid (0.28 g, 90% yield).

^{1}H NMR (400 MHz, $CDCl_3$) δ 7.26 (d, *J* = 7.6 Hz, 1H), 7.21 (d, *J* = 8.2 Hz, 1H), 7.07–7.00 (m, 2H), 6.50 (d, *J* = 3.2 Hz, 1H), 3.70 (s, 3H).

^{13}C NMR (100 MHz, $CDCl_3$) δ136.9, 129.3, 129.0, 122.3, 122.1, 114.7, 108.4, 101.2, 33.1.

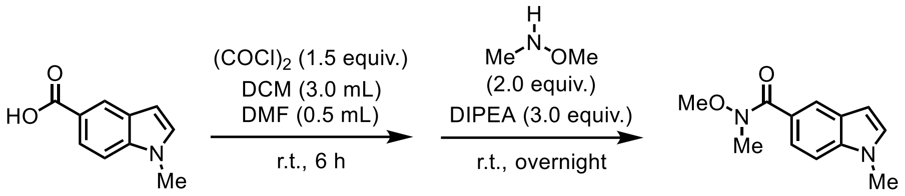

To a DCM solution of the 1-methyl-1*H*-indole-5-carboxylic acid (0.5 M) was added $(COCl)_2$ (2.3 mmol, 1.5 equiv.) and DMF (0.5 mL) under 0 °C. The reaction was stirred at room temperature for 6 h. *N*,*O*-dimethylhydroxylamine (3.0 mmol, 2.0 equiv.) and DIPEA (4.5 mmol, 3.0 equiv.) were then added under 0 °C, and the reaction was stirred at room temperature overnight. After reaction completion, the organic phase was washed twice with saturated aqueous sodium bicarbonate (100 mL). The organic phase was then dried with anhydrous Na_2SO_4, filtered on a short silica pad and the volatiles evaporated under reduced pressure. The products were usually used without further purification.

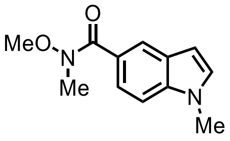

***N*-methoxy-*N*,1-dimethyl-1*H*-indole-5-carboxamide (1g).** Following the above procedure, **1g** was synthesized as a white solid (0.07 g, 20% yield).

^{1}H NMR (400 MHz, $CDCl_3$) δ 8.05 (d, *J* = 1.0 Hz, 1H), 7.61 (dd, *J* = 8.6, 1.6 Hz, 1H), 7.31 (dd, *J* = 8.6, 0.8 Hz, 1H), 7.10 (d, *J* = 3.2 Hz, 1H), 6.55 (dd, *J* = 3.2, 0.8 Hz, 1H), 3.81 (s, 3H), 3.58 (s, 3H), 3.39 (s, 3H).

^{13}C NMR (100 MHz, $CDCl_3$) δ 171.1, 137.8, 129.8, 127.6, 124.9, 122.2, 122.1, 108.6, 102.1, 60.8, 34.4, 32.9.

HRMS *m*/*z* (ESI) calcd. for $C_{12}H_{15}N_2O_2$ $[M+H]^+$: 219.1128; found: 219.1132.

6.3 Heterogeneous palladium-catalyzed C–H arylation

Table S5. Effect of Solvent in the C2-Selective C–H Arylation of *N*-methyl Indole 1a with Diphenyliodonium Tetrafluoroborate 2a

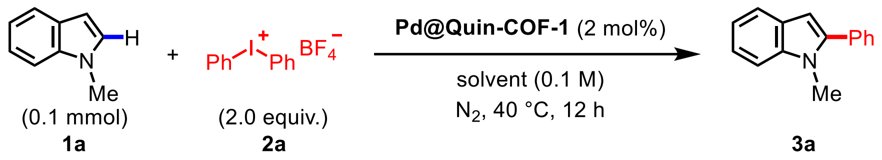

Entry	Solvent (1 mL)	Yield of **3a** (%)[a]
1	DCM	48
2	AcOH	19
3	toluene	11
4	acetonitrile	7
5	hexanes	<5
6	anhydrous THF	<5
7	DMF	<5
8	DMSO	<5
9	dioxane	<5
10	anhydrous DCE	66
11[b]	anhydrous DCE	54

[a]Yield was determined by ^{1}H NMR of the crude product using CH_2Br_2 as an internal standard.
[b]In air.

Table S6. Effect of Water Amount in the C2-Selective C–H Arylation of *N*-methyl Indole 1a with Diphenyliodonium Tetrafluoroborate 2a

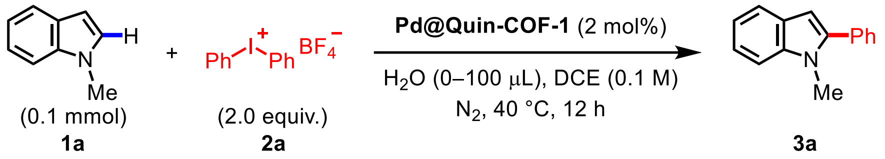

Entry	Solvent	Yield of **3a** (%)[a]
1	anhydrous DCE (1 mL) + H_2O (0 μL)	66
2	anhydrous DCE (1 mL) + H_2O (10 μL)	78
3	anhydrous DCE (1 mL) + H_2O (30 μL)	82
4	anhydrous DCE (1 mL) + H_2O (50 μL)	97
5	anhydrous DCE (1 mL) + H_2O (100 μL)	95
6[b]	anhydrous DCE (1 mL) + H_2O (50 μL)	<5

[a]Yield was determined by ^{1}H NMR of the crude product using CH_2Br_2 as an internal standard.

[b]Without **Pd@Quin-COF-1** catalyst.

Table S7. Optimization of the C2-Selective C–H Arylation using Pd@Quin-COF-1 as the Heterogeneous Catalyst

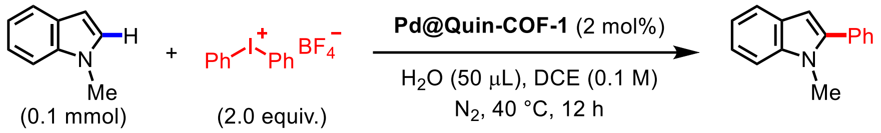

1a 2a 3a

Entry	Change from "Standard Conditions"	Yield of **3a** (%)[a]
1	none	97
2	r.t., instead of 40 °C	74
3	6 h	79
4	2 h	43
5	1 h	33
6	**Pd@Quin-COF-1** (1 mol%)	59
7	**2a** (1.5 equiv.)	69
8	**2a** (1.0 equiv.)	55
9	AcOH (50 μL), instead of H_2O	66
10	under an atmosphere of air, instead of N_2	76

[a]Yield was determined by ^1H NMR of the crude product using CH_2Br_2 as an internal standard.

Table S8. Homogenous Palladium Catalysis in Acetic Acid

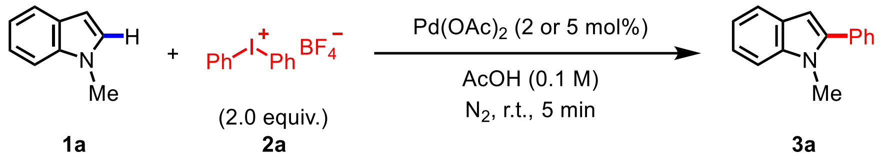

Entry	Loading of $Pd(OAc)_2$ (mol%)	Yield of **3a** (%)[a]
1	2	49
2	5	59

[a]Yield was determined by ^{1}H NMR of the crude product using CH_2Br_2 as an internal standard.

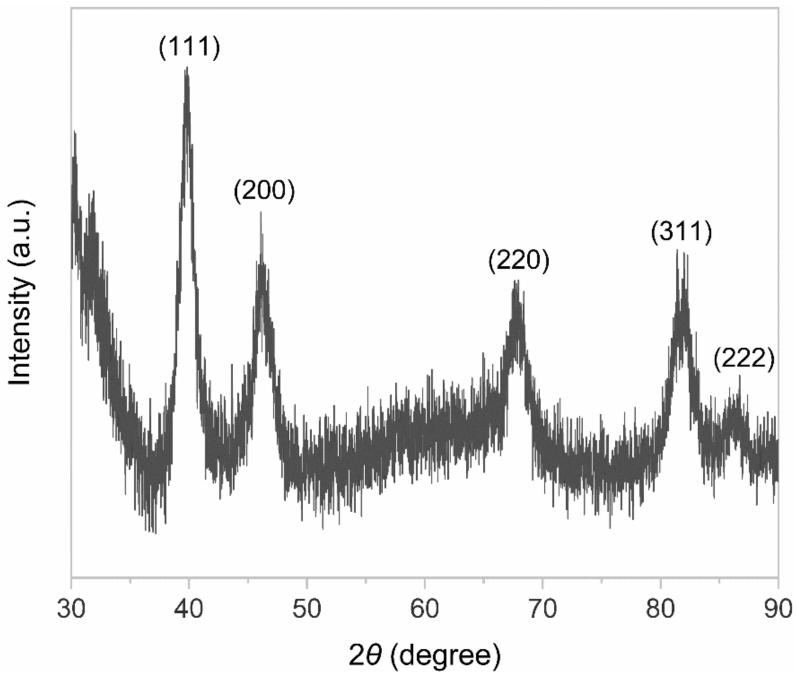

Figure S52. PXRD patterns of recycled palladium species in homogenous catalysis with $Pd(OAc)_2$ in acetic acid.

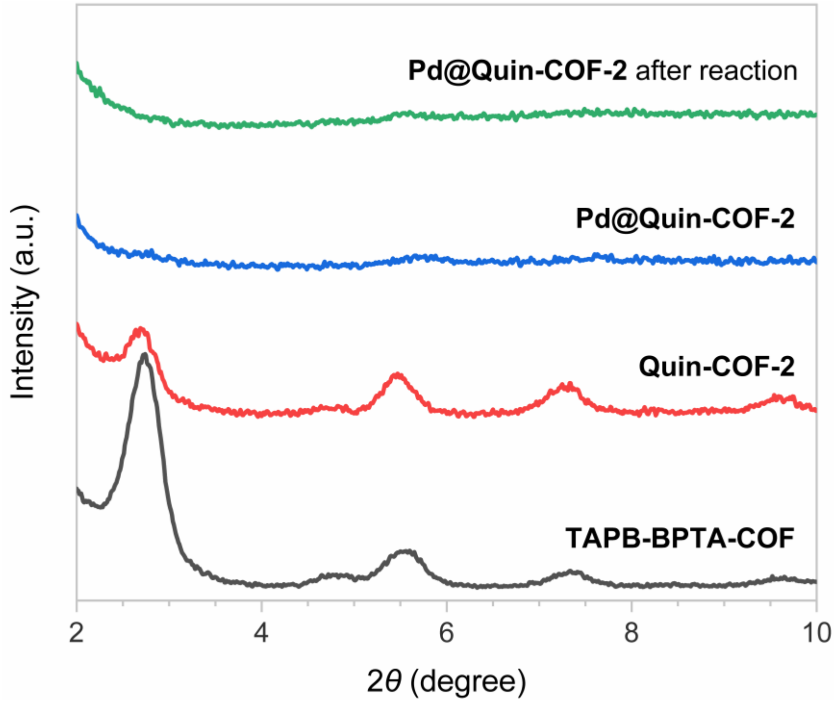

Figure S53. PXRD patterns of COF materials related to **Pd@Quin-COF-2**.

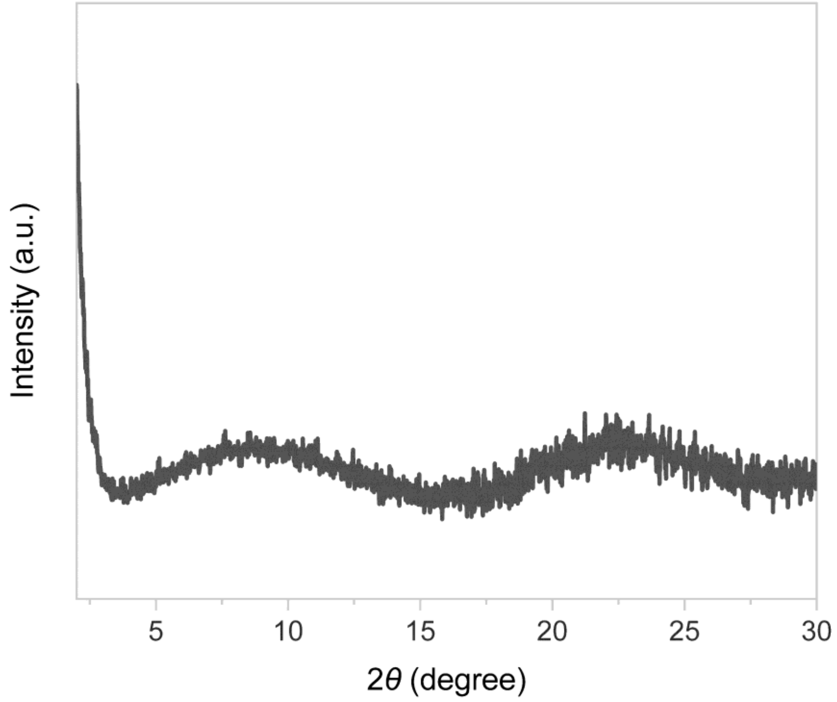

Figure S54. PXRD patterns of **Pd@Im-COF-8** after the catalytic reaction.

Table S9. Effects of Different Catalytic Systems on the C–H Arylation of 5a

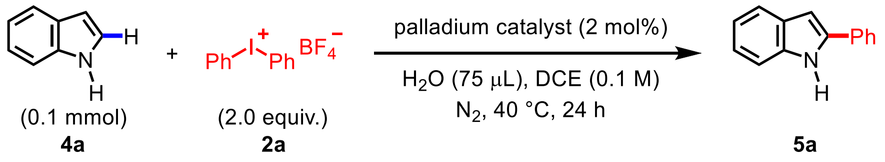

Entry	Catalyst (mol%)	Yield of **5a** (%)[a]
1	no catalyst	<5
2	**Pd@Quin-COF-1** (2)	55
3	**Pd@Im-COF-2** (2)	72
4	**Pd@Im-COF-3** (2)	51
5	**Pd@Im-COF-4** (2)	52
6	**Pd@Im-COF-5** (2)	<5
7	**Pd@Im-COF-6** (2)	<5
8	**Pd@Im-COF-7** (2)	<5
9[b]	**Pd@Im-COF-2** (2)	77

[a]Yield was determined by ^{1}H NMR of the crude product using CH_2Br_2 as an internal standard.

[b]48 h.

Table S10. Effect of Water Loadings in the C2-Selective C–H Arylation of Indole 4a with Diphenyliodonium Tetrafluoroborate 2a

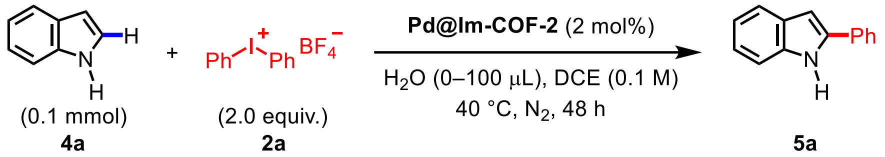

Entry	Solvent	Yield of **5a** (%)[a]
1	anhydrous DCE (1 mL)	35
2	anhydrous DCE (1 mL) + H_2O (25 μL)	59
3	anhydrous DCE (1 mL) + H_2O (50 μL)	62
4	anhydrous DCE (1 mL) + H_2O (75 μL)	77
5	anhydrous DCE (1 mL) + H_2O (100 μL)	67

[a]Yield was determined by ^1H NMR of the crude product using CH_2Br_2 as an internal standard.

Table S11. Investigation of Size Selectivity in COF-Based Palladium Catalysis

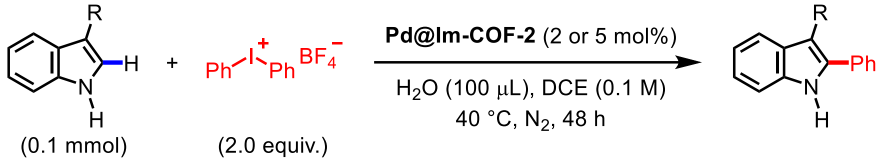

Entry	Substrate	Catalyst (mol%)	Product	Yield (%)[a]
1	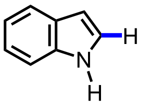	2	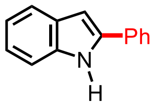	67
2	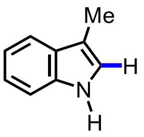	5	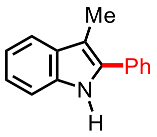	49
3	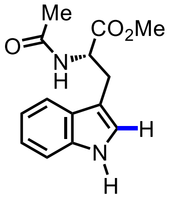	5	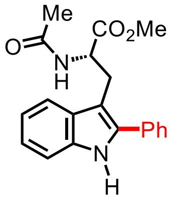	29

[a]Yield was determined by ^1H NMR of the crude product using CH_2Br_2 as an internal standard.

General procedure for the synthesis of COF-supported catalysts using different palladium salts: The palladium salt was dissolved in DCM (5 mL) to prepare a saturated solution. Upon removal of the undissolved solid, **Quin-COF-1** (25 mg) was added. The mixture was stirred slowly at 60 °C for 48 h. After reaction completion, the resulting solid was isolated by filtration and washed with DCM (3 × 50 mL), then dried at 70 °C for 12 h to yield the corresponding COF-supported palladium catalyst.

Table S12. Post-Synthetic Metalation of Quin-COF-1 with Different Palladium Salts

Entry	**PdX_n@Quin-COF-1**	Color	Pd Loading (wt%)[a]	Pd/N Ratio (experimental data)	Pd/N Ratio (theoretical data)
1	**$Pd(TFA)_2$@Quin-COF-1**	brown	7.2	1:4.3	1:1
2	**$PdCl_2$@Quin-COF-1**	dark red	1.3	1:30	1:1
3	**$PdBr_2$@Quin-COF-1**	dark red	0.1	1:290	1:1
4	**K_2PdCl_4@Quin-COF-1**	dark red	0.2	1:220	1:1

[a]Loading was determined by ICP-AES.

Table S13. Effect of Palladium Salts in Heterogeneous Catalysis for C–H Arylation

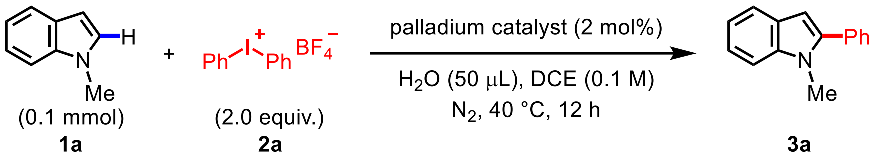

Entry	Catalyst	1st run yield (%)[a]	2nd run yield (%)[a]
1	**Pd@Quin-COF-1**	97	97
2	**Pd(TFA)$_2$@Quin-COF-1**	54	57
3	**PdCl$_2$@Quin-COF-1**	85	69

[a]Yield was determined by ^{1}H NMR of the crude product using CH_2Br_2 as an internal standard.

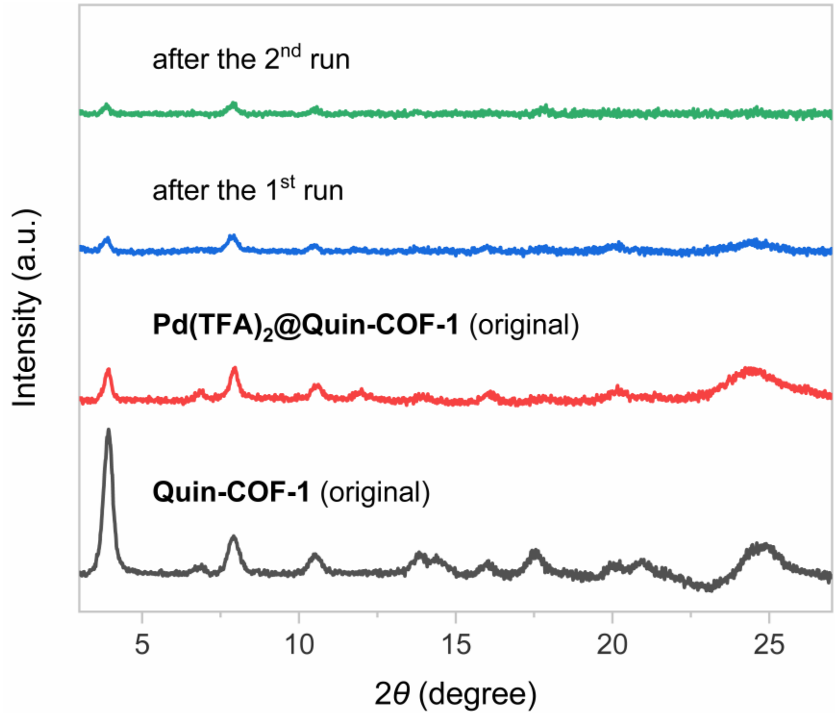

Figure S55. PXRD patterns of **Pd(TFA)$_2$@Quin-COF-1** before and after reaction.

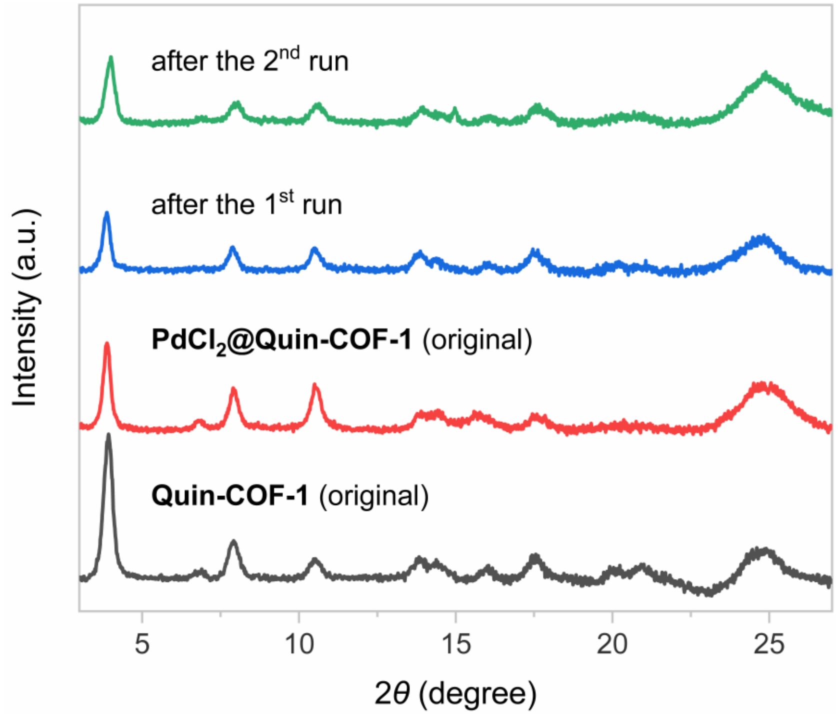

Figure S56. PXRD patterns of **PdCl$_2$@Quin-COF-1** before and after reaction.

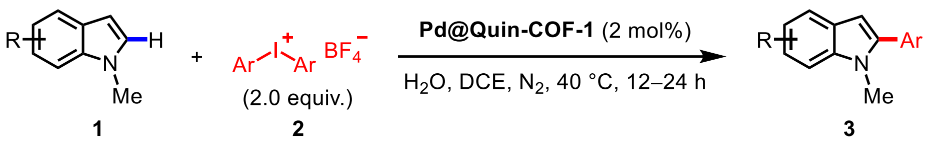

General Procedure C: A 10-mL Schlenk tube equipped with a magnetic stirrer was loaded with indole **1** (0.1 mmol, 1 equiv.), diaryliodonium salt **2** (0.2 mmol, 2 equiv.), **Pd@Quin-COF-1** (8.0 mg, 2 mol%), anhydrous DCE (1.0 mL), and H_2O (50 μL). The resulting mixture was degassed via three freeze-pump-thaw cycles and backfilled with N_2. Next, the reaction vessel was immersed in a water bath and stirred at 40 °C for 12–24 h. After reaction completion, the crude reaction mixture was concentrated under vacuum and purified by column chromatography to give the desired product **3**.

The amount of the COF-supported catalysts used in each reaction was determined based on ICP-AES. For example, the corresponding Pd content of **Pd@Quin-COF-1** was 2.6 wt%, which means that 10 mg of **Pd@Quin-COF-1** contains 0.26 mg (2.5 mol) of Pd catalyst. To run a catalytic reaction (on a 0.1-mmol scale) with 2 mol% of the Pd catalyst: the amount of **Pd@Quin-COF-1** = (0.1 mmol × 0.02) / 2.5 μmol × 10 mg = 8.0 mg.

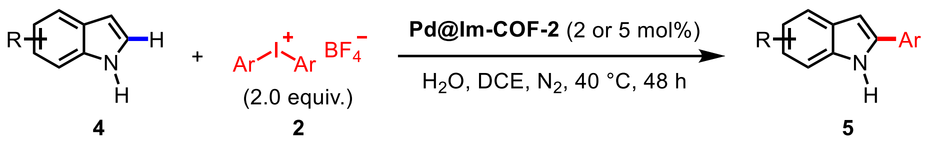

General Procedure D: A 10-mL Schlenk tube equipped with a magnetic stirrer was loaded with indole **4** (0.1 mmol, 1 equiv.), diaryliodonium salt **2** (0.2 mmol, 2 equiv.), **Pd@Im-COF-2** (2 or 5 mol%), anhydrous DCE (1.0 mL), and H_2O (75–100 μL). The resulting mixture was degassed via three freeze-pump-thaw cycles and backfilled with N_2. Next, the reaction vessel was immersed in a water bath and stirred at 40 °C for 48 h. After reaction completion, the crude reaction mixture was concentrated under vacuum and purified by column chromatography to give the desired product **5**.

The Pd content of **Pd@Im-COF-2** was 8.3 wt% as determined by ICP-AES. Therefore, 0.83 mg (7.8 μmol) of the Pd catalyst can be obtained in 10 mg of **Pd@Quin-COF-1**. To run a

catalytic reaction (on a 0.1-mmol scale) with 2 mol% of the Pd catalyst: the amount of **Pd@Im-COF-2** = (0.1 mmol × 0.02) / 10.2 µmol × 10 mg = 2.6 mg.

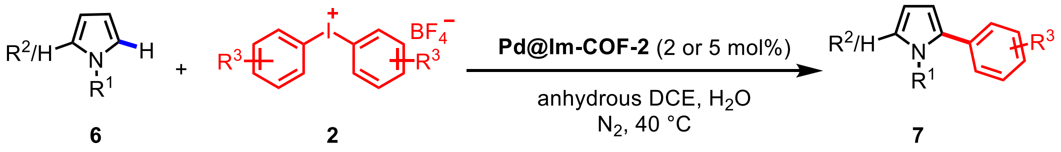

General Procedure E: A 10-mL Schlenk tube equipped with a magnetic stirrer was loaded with pyrrole **6** (1 mmol, 10 equiv.), diaryliodonium salt **2** (0.1 mmol, 1 equiv.), **Pd@Im-COF-2** (2 or 5 mol%), anhydrous DCE (1.0 mL), and H_2O (75 µL). The resulting mixture was degassed via three freeze-pump-thaw cycles and backfilled with N_2. Next, the reaction vessel was immersed in a water bath and stirred at 40 °C for 48 h. After reaction completion, the crude reaction mixture was concentrated under vacuum and purified by column chromatography to give the desired product **7**.

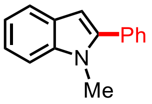

1-Methyl-2-phenyl-1*H*-indole (3a). The title compound was synthesized according to the General Procedure C using *N*-methyl indole (13.1 mg, 0.1 mmol), diphenyliodonium tetrafluoroborate (73.6 mg, 0.2 mmol), **Pd@Quin-COF-1** (8.0 mg, 2 mol%), anhydrous DCE (1.0 mL), and H_2O (50 µL). The resulting mixture was degassed via three freeze-pump-thaw cycles and backfilled with N_2. Next, the reaction vessel was immersed in a water bath and stirred at 40 °C for 12 h. After reaction completion, the crude reaction mixture was purified by flash column chromatography using hexanes/ethyl acetate (10:1 v/v) as the eluent to give **3a** as a white solid (20.1 mg, 97% yield).

^{1}H NMR (400 MHz, $CDCl_3$) δ 7.64 (d, J = 8.0 Hz, 1H), 7.51 (d, J = 6.8 Hz, 2H), 7.47 (t, J = 7.6 Hz, 2H), 7.41 (d, J = 7.0 Hz, 1H), 7.36 (d, J = 8.0 Hz, 1H), 7.24 (d, J = 8.0 Hz, 1H), 7.17–7.12 (m, 1H), 6.57 (s, 1H), 3.74 (s, 3H).

^{13}C NMR (100 MHz, $CDCl_3$) δ 141.5, 138.3, 132.8, 129.4, 128.5, 127.9, 127.8, 121.6, 120.4, 119.8, 109.6, 101.6, 31.2.

HRMS *m/z* (ESI) calcd. for $C_{15}H_{14}N$ $[M+H]^+$: 208.1121; found: 208.1101.

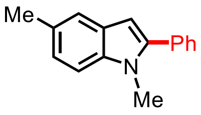

1,5-Dimethyl-2-phenyl-1*H*-indole (3b). The title compound was synthesized according to the General Procedure C using 1,5-dimethyl-1*H*-indole (14.5 mg, 0.1 mmol), diphenyliodonium tetrafluoroborate (73.6 mg, 0.2 mmol), **Pd@Quin-COF-1** (8.0 mg, 2 mol%), anhydrous DCE (1.0 mL), and H_2O (50 μL). The resulting mixture was degassed via three freeze-pump-thaw cycles and backfilled with N_2. Next, the reaction vessel was immersed in a water bath and stirred at 40 °C for 12 h. After reaction completion, the crude reaction mixture was purified by flash column chromatography using hexanes/ethyl acetate (80:1 v/v) as the eluent to give **3b** as a white solid (15.5 mg, 70% yield).

^{1}H NMR (400 MHz, $CDCl_3$) δ 7.53–7.38 (m, 6H), 7.25 (d, *J* = 4.2 Hz, 1H), 7.07 (d, *J* = 8.4 Hz, 1H), 6.48 (s, 1H), 3.73 (s, 3H), 2.47 (s, 3H).

^{13}C NMR (100 MHz, $CDCl_3$) δ 141.6, 136.8, 133.0, 129.3, 129.0, 128.4, 128.2, 127.7, 123.2, 120.1, 109.3, 101.1, 31.2, 21.4.

HRMS *m/z* (ESI) calcd. for $C_{16}H_{16}N$ $[M+H]^+$: 222.1277; found: 222.1261.

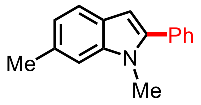

1,6-Dimethyl-2-phenyl-1*H*-indole (3c). The title compound was synthesized according to the General Procedure C using 1,6-dimethyl-1*H*-indole (14.5 mg, 0.1 mmol), diphenyliodonium tetrafluoroborate (73.6 mg, 0.2 mmol), **Pd@Quin-COF-1** (8.0 mg, 2 mol%), anhydrous DCE (1.0 mL), and H_2O (50 μL). The resulting mixture was degassed via three freeze-pump-thaw cycles and backfilled with N_2. Next, the reaction vessel was immersed in a water bath and stirred at 40 °C for 12 h. After reaction completion, the crude reaction mixture was purified by flash column chromatography using hexanes/ethyl acetate (80:1 v/v) as the

eluent to give **3c** as a white solid (19.2 mg, 87% yield).

^{1}H NMR (400 MHz, $CDCl_3$) δ 7.53–7.40 (m, 5H), 7.36 (t, *J* = 7.2 Hz, 1H), 7.14 (s, 1H), 6.97 (d, *J* = 8.0 Hz, 1H), 6.50 (s, 1H), 3.69 (s, 3H), 2.51 (s, 3H).

^{13}C NMR (100 MHz, $CDCl_3$) δ 141.0, 138.8, 133.0, 131.5, 129.3, 128.4, 127.6, 125.7, 121.6, 120.1, 109.6, 101.4, 31.1, 22.0.

HRMS *m/z* (ESI) calcd. for $C_{16}H_{16}N$ $[M+H]^+$: 222.1277; found: 222.1276.

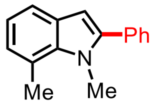

1,7-Dimethyl-2-phenyl-1*H*-indole (3d). The title compound was synthesized according to the General Procedure C using 1,7-dimethyl-1*H*-indole (14.5 mg, 0.1 mmol), diphenyliodonium tetrafluoroborate (73.6 mg, 0.2 mmol), **Pd@Quin-COF-1** (8.0 mg, 2 mol%), anhydrous DCE (1.0 mL), and H_2O (50 μL). The resulting mixture was degassed via three freeze-pump-thaw cycles and backfilled with N_2. Next, the reaction vessel was immersed in a water bath and stirred at 40 °C for 12 h. After reaction completion, the crude reaction mixture was purified by flash column chromatography using hexanes/ethyl acetate (80:1 v/v) as the eluent to give **3d** as a white solid (17.9 mg, 81% yield).

^{1}H NMR (400 MHz, $CDCl_3$) δ 7.52–7.43 (m, 5H), 7.43–7.36 (m, 1H), 7.00 (t, *J* = 7.4 Hz, 1H), 6.94 (d, *J* = 7.0 Hz, 1H), 6.52 (s, 1H), 3.94 (s, 3H), 2.82 (s, 3H).

^{13}C NMR (100 MHz, $CDCl_3$) δ 142.7, 137.6, 133.0, 129.6, 128.9, 128.4, 127.8, 124.7, 121.5, 120.0, 118.6, 102.4, 34.5, 20.2.

HRMS *m/z* (ESI) calcd. for $C_{16}H_{16}N$ $[M+H]^+$: 222.1277; found: 222.1263.

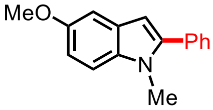

5-Methoxy-1-methyl-2-phenyl-1*H*-indole (3e). The title compound was synthesized according to the General Procedure C using 5-methoxy-1-methyl-1*H*-indole (16.4 mg, 0.1 mmol), diphenyliodonium tetrafluoroborate (73.6 mg, 0.2 mmol), **Pd@Quin-COF-1** (8.0 mg, 2 mol%), anhydrous DCE (1.0 mL), and H_2O (50 μL). The resulting mixture was degassed via

three freeze-pump-thaw cycles and backfilled with N_2. Next, the reaction vessel was immersed in a water bath and stirred at 40 °C for 12 h. After reaction completion, the crude reaction mixture was purified by flash column chromatography using hexanes/ethyl acetate (80:1 v/v) as the eluent to give **3e** as a white solid (20.4 mg, 86% yield).

^{1}H NMR (400 MHz, $CDCl_3$) δ 7.54–7.43 (m, 4H), 7.39 (t, *J* = 7.0 Hz, 1H), 7.25 (d, *J* = 2.0 Hz, 1H), 7.11 (d, *J* = 2.4 Hz, 1H), 6.91 (dd, *J* = 8.8, 2.4 Hz, 1H), 6.49 (s, 1H), 3.87 (s, 3H), 3.72 (s, 3H).

^{13}C NMR (100 MHz, $CDCl_3$) δ 154.3, 142.1, 133.8, 132.9, 129.3, 128.5, 128.2, 127.8, 111.9, 110.3, 102.1, 101.3, 55.9, 31.3.

HRMS *m/z* (ESI) calcd. for $C_{16}H_{16}NO$ $[M+H]^+$: 238.1226; found: 238.1206.

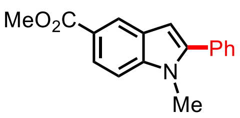

Methyl 1-methyl-2-phenyl-1*H*-indole-5-carboxylate (3f). The title compound was synthesized according to the General Procedure C using methyl 1-methyl-1*H*-indole-5-carboxylate (18.9 mg, 0.1 mmol), diphenyliodonium tetrafluoroborate (73.6 mg, 0.2 mmol), **Pd@Quin-COF-1** (8.0 mg, 2 mol%), anhydrous DCE (1.0 mL), and H_2O (50 μL). The resulting mixture was degassed via three freeze-pump-thaw cycles and backfilled with N_2. Next, the reaction vessel was immersed in a water bath and stirred at 40 °C for 24 h. After reaction completion, the crude reaction mixture was purified by flash column chromatography using hexanes/ethyl acetate (80:1 v/v) as the eluent to give **3f** as a gray solid powder (16.7 mg, 63% yield).

^{1}H NMR (400 MHz, $CDCl_3$) δ 8.40 (d, *J* = 1.6 Hz, 1H), 7.96 (dd, *J* = 8.8, 1.6 Hz, 1H), 7.54–7.40 (m, 5H), 7.36 (d, *J* = 8.8 Hz, 1H), 6.64 (s, 1H), 3.94 (s, 3H), 3.77 (s, 3H).

^{13}C NMR (100 MHz, $CDCl_3$) δ 168.2, 143.0, 140.7, 132.2, 129.4, 128.6, 128.3, 127.4, 123.4, 123.0, 121.8, 109.2, 102.9, 51.8, 31.4.

HRMS *m/z* (ESI) calcd. for $C_{17}H_{16}NO_2$ $[M+H]^+$: 266.1176; found: 266.1172.

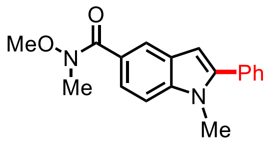

***N*-methoxy-N,1-dimethyl-2-phenyl-1*H*-indole-5-carboxamide (3g).** The title compound was synthesized according to the General Procedure C using *N*-methoxy-*N*,1-dimethyl-1*H*-indole-5-carboxamide (21.8 mg, 0.1 mmol), diphenyliodonium tetrafluoroborate (73.6 mg, 0.2 mmol), **Pd@Quin-COF-1** (8.0 mg, 2 mol%), anhydrous DCE (1.0 mL), and H_2O (50 µL). The resulting mixture was degassed via three freeze-pump-thaw cycles and backfilled with N_2. Next, the reaction vessel was immersed in a water bath and stirred at 40 °C for 12 h. After reaction completion, the crude reaction mixture was purified by flash column chromatography using hexanes/ethyl acetate (80:1 v/v) as the eluent to give **3g** as a white solid powder (25.0 mg, 85% yield).

^{1}H NMR (400 MHz, $CDCl_3$) δ 8.05 (s, 1H), 7.63 (d, *J* = 8.4 Hz, 1H), 7.44 (q, *J* = 7.6 Hz, 4H), 7.38 (d, *J* = 7.2 Hz, 1H), 7.31 (d, *J* = 8.6 Hz, 1H), 6.59 (s, 1H), 3.69 (s, 3H), 3.56 (s, 3H), 3.37 (s, 3H).

^{13}C NMR (100 MHz, $CDCl_3$) δ 170.8, 142.4, 139.2, 132.0, 129.0, 128.3, 128.0, 126.9, 125.2, 122.1, 121.4, 108.8, 102.3, 60.6, 34.2, 31.1.

HRMS *m/z* (ESI) calcd. for $C_{18}H_{19}N_2O_2$ $[M+H]^+$: 295.1441; found: 295.1461.

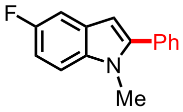

5-Fluoro-1-methyl-2-phenyl-1*H*-indole (3h). The title compound was synthesized according to the General Procedure C using 5-fluoro-1-methyl-1*H*-indole (14.9 mg, 0.1 mmol), diphenyliodonium tetrafluoroborate (73.6 mg, 0.2 mmol), **Pd@Quin-COF-1** (8.0 mg, 2 mol%), anhydrous DCE (1.0 mL), and H_2O (50 µL). The resulting mixture was degassed via three freeze-pump-thaw cycles and backfilled with N_2. Next, the reaction vessel was immersed in a water bath and stirred at 40 °C for 12 h. After reaction completion, the crude reaction mixture was purified by flash column chromatography using hexanes/ethyl acetate (80:1 v/v) as the eluent to give **3h** as a yellow solid (16.9 mg, 75% yield).

^{1}H NMR (400 MHz, $CDCl_3$) δ 7.48 (q, J = 7.8 Hz, 4H), 7.44–7.39 (m, 1H), 7.30–7.23 (m, 2H), 6.99 (td, J = 9.0, 2.4 Hz, 1H), 6.52 (s, 1H), 3.73 (s, 3H).

^{13}C NMR (100 MHz, $CDCl_3$) δ 159.2 (d, J = 234.3 Hz), 143.1, 135.0, 132.5, 129.3, 128.5, 128.1, 128.0 (d, J = 10.2 Hz), 110.2 (d, J = 9.7 Hz), 109.9 (d, J = 26.4 Hz), 105.1 (d, J = 23.5 Hz), 101.5 (d, J = 4.7 Hz), 31.3.

^{19}F NMR (376 MHz, $CDCl_3$) δ -124.8.

HRMS *m*/*z* (ESI) calcd. for $C_{15}H_{13}FN$ $[M+H]^+$: 226.1027; found: 226.1011.

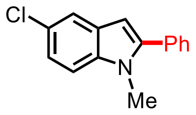

5-Chloro-1-methyl-2-phenyl-1*H*-indole (3i). The title compound was synthesized according to the General Procedure C using 5-chloro-1-methyl-1*H*-indole (16.5 mg, 0.1 mmol), diphenyliodonium tetrafluoroborate (73.6 mg, 0.2 mmol), **Pd@Im-COF-2** (6.4 mg, 5 mol%), anhydrous DCE (1.0 mL), and H_2O (50 μL). The resulting mixture was degassed via three freeze-pump-thaw cycles and backfilled with N_2. Next, the reaction vessel was immersed in a water bath and stirred at 40 °C for 24 h. After reaction completion, the crude reaction mixture was purified by flash column chromatography using hexanes/ethyl acetate (80:1 v/v) as the eluent to give **3i** as a white solid (19.0 mg, 79% yield).

^{1}H NMR (400 MHz, $CDCl_3$) δ 7.59 (s, 1H), 7.53–7.37 (m, 5H), 7.27–7.23 (m, 1H), 7.21–7.15 (m, 1H), 6.49 (s, 1H), 3.73 (s, 3H).

^{13}C NMR (100 MHz, $CDCl_3$) δ 142.8, 136.7, 132.3, 129.3, 128.9, 128.6, 128.2, 125.5, 121.8, 119.8, 110.6, 101.2, 31.3.

HRMS *m*/*z* (ESI) calcd. for $C_{15}H_{13}ClN$ $[M+H]^+$: 242.0731; found: 242.0743.

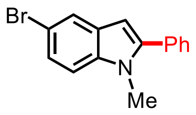

5-Bromo-1-methyl-2-phenyl-1*H*-indole (3j). The title compound was synthesized according to the General Procedure C using 5-bromo-1-methyl-1*H*-indole (20.9 mg, 0.1 mmol), diphenyliodonium tetrafluoroborate (73.6 mg, 0.2 mmol), **Pd@Quin-COF-1** (8.0 mg, 2 mol%),

anhydrous DCE (1.0 mL), and H_2O (50 μL). The resulting mixture was degassed via three freeze-pump-thaw cycles and backfilled with N_2. Next, the reaction vessel was immersed in a water bath and stirred at 40 °C for 12 h. After reaction completion, the crude reaction mixture was purified by flash column chromatography using hexanes/ethyl acetate (80:1 v/v) as the eluent to give **3j** as an orange solid (22.8 mg, 80% yield).

^{1}H NMR (400 MHz, $CDCl_3$) δ 7.74 (d, *J* = 1.6 Hz, 1H), 7.50–7.44 (m, 4H), 7.41 (m, 1H), 7.31 (dd, *J* = 8.8, 2.0 Hz, 1H), 7.21 (d, *J* = 8.4 Hz, 1H), 6.48 (s, 1H), 3.71 (s, 3H).

^{13}C NMR (100 MHz, $CDCl_3$) δ 142.7, 137.0, 132.2, 129.5, 129.3, 128.6, 128.2, 124.4, 122.8, 113.0, 111.0, 101.1, 31.3.

HRMS *m/z* (ESI) calcd. for $C_{15}H_{13}BrN$ $[M+H]^+$: 286.0226; found: 286.0211.

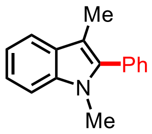

1,3-Dimethyl-2-phenyl-1*H*-indole (3k). The title compound was synthesized according to the General Procedure C using 1,3-dimethyl-1*H*-indole (14.5 mg, 0.1 mmol), diphenyliodonium tetrafluoroborate (73.6 mg, 0.2 mmol), **Pd@Quin-COF-1** (8.0 mg, 2 mol%), anhydrous DCE (1.0 mL), and H_2O (50 μL). The resulting mixture was degassed via three freeze-pump-thaw cycles and backfilled with N_2. Next, the reaction vessel was immersed in a water bath and stirred at 40 °C for 12 h. After reaction completion, the crude reaction mixture was purified by flash column chromatography using hexanes/ethyl acetate (80:1 v/v) as the eluent to give **3k** as a white solid (12.6 mg, 57% yield).

^{1}H NMR (400 MHz, $CDCl_3$) δ 7.60 (d, *J* = 7.8 Hz, 1H), 7.49 (t, *J* = 7.6 Hz, 2H), 7.45–7.37 (m, 3H), 7.33 (d, *J* = 8.0 Hz, 1H), 7.27–7.23 (m, 1H), 7.15 (t, *J* = 7.4 Hz, 1H), 3.61 (s, 3H), 2.29 (s, 3H).

^{13}C NMR (100 MHz, $CDCl_3$) δ 137.6, 137.2, 132.1, 130.6, 128.4, 128.3, 127.7, 121.7, 119.1, 118.8, 109.2, 108.5, 30.9, 9.3.

HRMS *m/z* (ESI) calcd. for $C_{16}H_{16}N$ $[M+H]^+$: 222.1277; found: 222.1249.

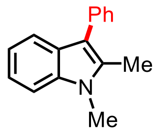

1,2-Dimethyl-3-phenyl-1*H*-indole (3l). The title compound was synthesized according to the General Procedure C using 1,2-dimethyl-1*H*-indole (14.5 mg, 0.1 mmol), diphenyliodonium tetrafluoroborate (73.6 mg, 0.2 mmol), **Pd@Quin-COF-1** (8.0 mg, 2 mol%), anhydrous DCE (1.0 mL), and H_2O (50 μL). The resulting mixture was degassed via three freeze-pump-thaw cycles and backfilled with N_2. Next, the reaction vessel was immersed in a water bath and stirred at 40 °C for 24 h. After reaction completion, the crude reaction mixture was purified by flash column chromatography using hexanes/ethyl acetate (80:1 v/v) as the eluent to give **3l** as a yellow solid (11.5 mg, 52% yield).

^{1}H NMR (400 MHz, $CDCl_3$) δ 7.66 (d, *J* = 7.6 Hz, 1H), 7.51–7.42 (m, 4H), 7.34–7.27 (m, 2H), 7.23–7.18 (m, 1H), 7.13–7.08 (m, 1H), 3.74 (s, 3H), 2.49 (s, 3H).

^{13}C NMR (100 MHz, $CDCl_3$) δ 136.6, 135.8, 133.3, 129.7, 128.4, 126.9, 125.7, 121.1, 119.6, 118.7, 114.0, 108.7, 29.6, 11.1.

HRMS *m/z* (ESI) calcd. for $C_{16}H_{16}N$ $[M+H]^+$: 222.1277; found: 222.1269.

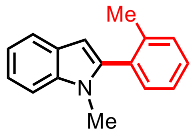

1-Methyl-2-(*o*-tolyl)-1*H*-indole (3m). The title compound was synthesized according to the General Procedure C using *N*-methyl indole (13.1 mg, 0.1 mmol), di-*o*-tolyliodonium tetrafluoroborate (79.2 mg, 0.2 mmol), **Pd@Quin-COF-1** (8.0 mg, 2 mol%), anhydrous DCE (1.0 mL), and H_2O (50 μL). The resulting mixture was degassed via three freeze-pump-thaw cycles and backfilled with N_2. Next, the reaction vessel was immersed in a water bath and stirred at 40 °C for 24 h. After reaction completion, the crude reaction mixture was purified by flash column chromatography using hexanes/ethyl acetate (80:1 v/v) as the eluent to give **3m** as a white solid (16.4 mg, 74% yield).

^{1}H NMR (400 MHz, $CDCl_3$) δ 7.64 (d, *J* = 7.6 Hz, 1H), 7.38–7.22 (m, 6H), 7.18–7.12 (m, 1H), 6.43 (s, 1H), 3.51 (s, 3H), 2.20 (s, 3H).

^{13}C NMR (100 MHz, $CDCl_3$) δ 140.5, 138.0, 137.3, 132.5, 131.1, 130.0, 128.6, 128.0, 125.5, 121.2, 120.4, 119.6, 109.4, 101.5, 30.3, 20.0.

HRMS *m/z* (ESI) calcd. for $C_{16}H_{16}N$ $[M+H]^+$: 222.1277; found: 222.1280.

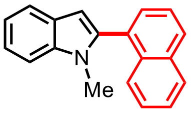

1-Methyl-2-(naphthalen-1-yl)-1*H*-indole (3n). The title compound was synthesized according to the General Procedure C using *N*-methyl indole (13.1 mg, 0.1 mmol), di(naphthalen-1-yl)iodonium tetrafluoroborate (93.6 mg, 0.2 mmol), **Pd@Quin-COF-1** (8.0 mg, 2 mol%), anhydrous DCE (1.0 mL), and H_2O (50 µL). The resulting mixture was degassed via three freeze-pump-thaw cycles and backfilled with N_2. Next, the reaction vessel was immersed in a water bath and stirred at 40 °C for 24 h. After reaction completion, the crude reaction mixture was purified by flash column chromatography using hexanes/ethyl acetate (80:1 v/v) as the eluent to give **3n** as a pale-yellow solid (17.0 mg, 66% yield).

^{1}H NMR (400 MHz, $CDCl_3$) δ 7.98–7.90 (m, 2H), 7.70 (d, J = 7.8 Hz, 2H), 7.59–7.47 (m, 3H), 7.47–7.38 (m, 2H), 7.33–7.26 (m, 1H), 7.23–7.16 (m, 1H), 6.63 (s, 1H), 3.49 (s, 3H).

^{13}C NMR (100 MHz, $CDCl_3$) δ 139.4, 137.6, 133.5, 132.9, 130.5, 129.0, 128.9, 128.3, 128.0, 126.6, 126.1, 125.2, 121.5, 120.5, 119.8, 109.5, 103.0, 30.8.

HRMS *m/z* (ESI) calcd. for $C_{19}H_{16}N$ $[M+H]^+$: 258.1277; found: 258.1279.

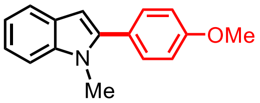

2-(4-Methoxyphenyl)-1-methyl-1*H*-indole (3o). The title compound was synthesized according to the General Procedure C using *N*-methyl indole (13.1 mg, 0.1 mmol), bis(4-methoxyphenyl)iodonium tetrafluoroborate (85.6 mg, 0.2 mmol), **Pd@Quin-COF-1** (8.0 mg, 2 mol%), anhydrous DCE (1.0 mL), and H_2O (50 µL). The resulting mixture was degassed via three freeze-pump-thaw cycles and backfilled with N_2. Next, the reaction vessel was immersed in a water bath and stirred at 40 °C for 24 h. After reaction completion, the crude reaction mixture was purified by flash column chromatography using hexanes/ethyl acetate (80:1 v/v)

as the eluent to give **3o** as a brown solid (19.2 mg, 81% yield).

^{1}H NMR (400 MHz, $CDCl_3$) δ 7.62 (d, *J* = 7.6 Hz, 1H), 7.44 (d, *J* = 8.8 Hz, 2H), 7.35 (d, *J* = 8.2 Hz, 1H), 7.22 (d, *J* = 8.2 Hz, 1H), 7.13 (t, *J* = 7.2 Hz, 1H), 7.01 (d, *J* = 8.8 Hz, 2H), 6.50 (s, 1H), 3.87 (s, 3H), 3.73 (s, 3H).

^{13}C NMR (100 MHz, $CDCl_3$) δ 159.4, 141.4, 138.1, 130.6, 128.0, 125.3, 121.4, 120.2, 119.7, 114.0, 109.5, 101.0, 55.4, 31.1.

HRMS *m/z* (ESI) calcd. for $C_{16}H_{16}NO$ $[M+H]^+$: 238.1226; found: 238.1222.

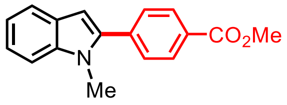

Methyl 4-(1-methyl-1*H*-indol-2-yl)benzoate (3p). The title compound was synthesized according to the General Procedure C using *N*-methyl indole (13.1 mg, 0.1 mmol), bis(4-(methoxycarbonyl)phenyl)iodonium tetrafluoroborate (96.8 mg, 0.2 mmol), **Pd@Quin-COF-1** (8.0 mg, 2 mol%), anhydrous DCE (1.0 mL), and H_2O (50 μL). The resulting mixture was degassed via three freeze-pump-thaw cycles and backfilled with N_2. Next, the reaction vessel was immersed in a water bath and stirred at 40 °C for 24 h. After reaction completion, the crude reaction mixture was purified by flash column chromatography using hexanes/ethyl acetate (80:1 v/v) as the eluent to give **3p** as a yellow solid (24.4 mg, 92% yield).

^{1}H NMR (400 MHz, $CDCl_3$) δ 8.13 (d, *J* = 8.4 Hz, 2H), 7.65 (d, *J* = 8.0 Hz, 1H), 7.58 (d, *J* = 8.4 Hz, 2H), 7.37 (d, *J* = 8.2 Hz, 1H), 7.31–7.23 (m, 1H), 7.19–7.12 (m, 1H), 6.64 (s, 1H), 3.95 (s, 3H), 3.76 (s, 3H).

^{13}C NMR (100 MHz, $CDCl_3$) δ 166.8, 140.3, 138.8, 137.2, 129.8, 129.2, 129.0, 127.8, 122.3, 120.7, 120.1, 109.7, 102.8, 52.2, 31.4.

HRMS *m/z* (ESI) calcd. for $C_{17}H_{16}NO_2$ $[M+H]^+$: 266.1176; found: 266.1182.

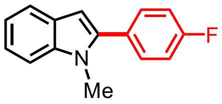

2-(4-Fluorophenyl)-1-methyl-1*H*-indole (3q). The title compound was synthesized according to the General Procedure C using *N*-methyl indole (13.1 mg, 0.1 mmol), bis(4-

fluorophenyl)iodonium tetrafluoroborate (80.8 mg, 0.2 mmol), **Pd@Quin-COF-1** (8.0 mg, 2 mol%), anhydrous DCE (1.0 mL), and H_2O (50 μL). The resulting mixture was degassed via three freeze-pump-thaw cycles and backfilled with N_2. Next, the reaction vessel was immersed in a water bath and stirred at 40 °C for 12 h. After reaction completion, the crude reaction mixture was purified by flash column chromatography using hexanes/ethyl acetate (80:1 v/v) as the eluent to give **3q** as a brown solid (20.0 mg, 89% yield).

^{1}H NMR (500 MHz, $CDCl_3$) δ 7.62 (d, *J* = 7.5 Hz, 1H), 7.46 (dd, *J* = 8.5, 5.5 Hz, 2H), 7.35 (d, *J* = 8.5 Hz, 1H), 7.25 (t, *J* = 7.5 Hz, 1H), 7.15 (td, *J* = 8.0, 7.5, 4.0 Hz, 3H), 6.52 (s, 1H), 3.70 (s, 3H).

^{13}C NMR (125 MHz, $CDCl_3$) δ 162.6 (d, *J* = 247.5 Hz), 140.4, 138.2, 131.0 (d, *J* = 8.0 Hz), 128.9 (d, *J* = 3.5 Hz), 127.8, 121.8, 120.4, 119.9, 115.5 (d, *J* = 21.5 Hz), 109.6, 101.7, 31.0.

^{19}F NMR (470 MHz, $CDCl_3$) δ -113.84.

HRMS *m/z* (ESI) calcd. for $C_{15}H_{13}FN$ $[M+H]^+$: 226.1027; found: 226.1023.

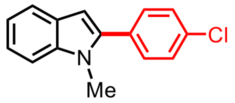

2-(4-Chlorophenyl)-1-methyl-1*H*-indole (3r). The title compound was synthesized according to the General Procedure C using *N*-methyl indole (13.1 mg, 0.1 mmol), bis(4-chlorophenyl)iodonium tetrafluoroborate (87.2 mg, 0.2 mmol), **Pd@Quin-COF-1** (8.0 mg, 2 mol%), anhydrous DCE (1.0 mL), and H_2O (50 μL). The resulting mixture was degassed via three freeze-pump-thaw cycles and backfilled with N_2. Next, the reaction vessel was immersed in a water bath and stirred at 40 °C for 12 h. After reaction completion, the crude reaction mixture was purified by flash column chromatography using hexanes/ethyl acetate (80:1 v/v) as the eluent to give **3r** as a white solid (23.1 mg, 96% yield).

^{1}H NMR (400 MHz, $CDCl_3$) δ 7.63 (d, *J* = 8.0 Hz, 1H), 7.43 (s, 4H), 7.35 (d, *J* = 8.2 Hz, 1H), 7.27–7.22 (m, 1H), 7.14 (t, *J* = 7.0 Hz, 1H), 6.55 (s, 1H), 3.72 (s, 3H).

^{13}C NMR (100 MHz, $CDCl_3$) δ 140.2, 138.4, 134.0, 131.3, 130.5, 128.7, 127.8, 121.9, 120.5, 120.0, 109.6, 102.0, 31.2.

HRMS *m/z* (ESI) calcd. for $C_{15}H_{13}ClN$ $[M+H]^+$: 242.0731; found: 242.0718.

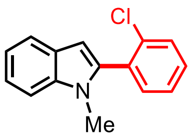

2-(2-Chlorophenyl)-1-methyl-1*H*-indole (3s). The title compound was synthesized according to the General Procedure C using *N*-methyl indole (13.1 mg, 0.1 mmol), bis(2-chlorophenyl)iodonium tetrafluoroborate (87.2 mg, 0.2 mmol), **Pd@Quin-COF-1** (8.0 mg, 2 mol%), anhydrous DCE (1.0 mL), and H_2O (50 µL). The resulting mixture was degassed via three freeze-pump-thaw cycles and backfilled with N_2. Next, the reaction vessel was immersed in a water bath and stirred at 40 °C for 12 h. After reaction completion, the crude reaction mixture was purified by flash column chromatography using hexanes/ethyl acetate (80:1 v/v) as the eluent to give **3s** as a white solid (22.7 mg, 94% yield).

^{1}H NMR (400 MHz, $CDCl_3$) δ 7.65 (d, J = 7.6 Hz, 1H), 7.50 (d, J = 7.4 Hz, 1H), 7.40 (td, J = 6.8, 2.2 Hz, 1H), 7.38–7.31 (m, 3H), 7.26 (t, J = 7.4 Hz, 1H), 7.15 (t, J = 7.4 Hz, 1H), 6.52 (s, 1H), 3.57 (s, 3H).

^{13}C NMR (100 MHz, $CDCl_3$) δ 138.2, 137.4, 134.8, 132.8, 132.1, 129.9, 129.6, 127.6, 126.6, 121.7, 120.7, 119.7, 109.5, 102.3, 30.6.

HRMS *m/z* (ESI) calcd. for $C_{15}H_{13}ClN$ $[M+H]^+$: 242.0731; found: 242.0740.

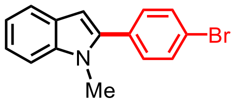

2-(4-Bromophenyl)-1-methyl-1*H*-indole (3t). The title compound was synthesized according to the General Procedure C using *N*-methyl indole (13.1 mg, 0.1 mmol), bis(4-bromophenyl)iodonium tetrafluoroborate (104.8 mg, 0.2 mmol), **Pd@Quin-COF-1** (8.0 mg, 2 mol%), anhydrous DCE (1.0 mL), and H_2O (50 µL). The resulting mixture was degassed via three freeze-pump-thaw cycles and backfilled with N_2. Next, the reaction vessel was immersed in a water bath and stirred at 40 °C for 12 h. After reaction completion, the crude reaction mixture was purified by flash column chromatography using hexanes/ethyl acetate (80:1 v/v) as the eluent to give **3t** as a yellow solid (26.2 mg, 92% yield).

^{1}H NMR (400 MHz, $CDCl_3$) δ 7.62 (d, J = 7.8 Hz, 1H), 7.58 (d, J = 8.4 Hz, 2H), 7.38–

7.32 (m, 3H), 7.25 (t, J = 7.6 Hz, 1H), 7.14 (t, J = 7.4 Hz, 1H), 6.55 (s, 1H), 3.70 (s, 3H).

^{13}C NMR (100 MHz, $CDCl_3$) δ 140.2, 138.4, 131.70, 131.68, 130.8, 127.8, 122.1, 122.0, 120.5, 120.0, 109.6, 102.0, 31.1.

HRMS *m/z* (ESI) calcd. for $C_{15}H_{12}BrN$ $[M+H]^+$: 286.0226; found: 286.0214.

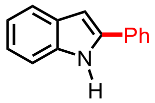

2-Phenyl-1*H*-indole (5a). The title compound was synthesized according to the General Procedure D using 1*H*-indole (11.7 mg, 0.1 mmol), diphenyliodonium tetrafluoroborate (73.6 mg, 0.2 mmol), **Pd@Im-COF-2** (2.6 mg, 2 mol%), anhydrous DCE (1.0 mL), and H_2O (75 µL). The resulting mixture was degassed via three freeze-pump-thaw cycles and backfilled with N_2. Next, the reaction vessel was immersed in a water bath and stirred at 40 °C for 48 h. After reaction completion, the crude reaction mixture was purified by flash column chromatography using hexanes/ethyl acetate (20:1 v/v) as the eluent to give **5a** as a pale brown solid (14.3 mg, 74% yield).

^{1}H NMR (400 MHz, $CDCl_3$) δ 8.29 (br, 1H), 7.63 (t, J = 6.4 Hz, 3H), 7.46–7.35 (m, 3H), 7.34–7.27 (m, 1H), 7.19 (t, J = 7.6 Hz, 1H), 7.12 (t, J = 7.4 Hz, 1H), 6.82 (s, 1H).

^{13}C NMR (100 MHz, $CDCl_3$) δ 137.8, 136.8, 132.3, 129.2, 129.0, 127.7, 125.1, 122.3, 120.6, 120.2, 110.9, 99.9.

HRMS *m/z* (ESI) calcd. for $C_{14}H_{12}N$ $[M+H]^+$: 194.0964; found: 194.0954.

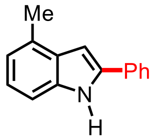

4-Methyl-2-phenyl-1*H*-indole (5b). The title compound was synthesized according to the General Procedure D using 4-methyl-1*H*-indole (13.1 mg, 0.1 mmol), diphenyliodonium tetrafluoroborate (73.6 mg, 0.2 mmol), and **Pd@Im-COF-2** (6.4 mg, 5 mol%), anhydrous DCE (1.0 mL), and H_2O (75 µL). The resulting mixture was degassed via three freeze-pump-thaw cycles and backfilled with N_2. Next, the reaction vessel was immersed in a water bath and stirred at 40 °C for 48 h. After reaction completion, the crude reaction mixture was purified by

flash column chromatography using hexanes/ethyl acetate (20:1 v/v) as the eluent to give **5b** as a brown solid (9.8 mg, 48% yield).

^{1}H NMR (400 MHz, $CDCl_3$) δ 8.30 (br, 1H), 7.65 (d, *J* = 7.6 Hz, 2H), 7.42 (t, *J* = 7.8 Hz, 2H), 7.30 (t, *J* = 7.4 Hz, 1H), 7.22 (d, *J* = 8.4 Hz, 1H), 7.10 (t, *J* = 7.6 Hz, 1H), 6.92 (d, *J* = 7.2 Hz, 1H), 6.84 (d, *J* = 1.6 Hz, 1H), 2.58 (s, 3H).

^{13}C NMR (100 MHz, $CDCl_3$) δ 137.2, 136.5, 132.4, 130.2, 129.1, 129.0, 127.6, 125.0, 122.5, 120.3, 108.5, 98.5, 18.8.

HRMS *m*/*z* (ESI) calcd. for $C_{15}H_{14}N$ $[M+H]^+$: 208.1121; found: 208.1106.

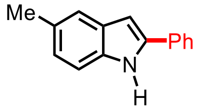

5-Methyl-2-phenyl-1*H*-indole (5c). The title compound was synthesized according to the General Procedure D using 5-methyl-1*H*-indole (13.1 mg, 0.1 mmol), diphenyliodonium tetrafluoroborate (73.6 mg, 0.2 mmol), **Pd@Im-COF-2** (2.6 mg, 2 mol%), anhydrous DCE (1.0 mL), and H_2O (75 μL). The resulting mixture was degassed via three freeze-pump-thaw cycles and backfilled with N_2. Next, the reaction vessel was immersed in a water bath and stirred at 40 °C for 48 h. After reaction completion, the crude reaction mixture was purified by flash column chromatography using hexanes/ethyl acetate (20:1 v/v) as the eluent to give **5c** as a white solid (15.4 mg, 74% yield).

^{1}H NMR (400 MHz, $CDCl_3$) δ 8.22 (br, 1H), 7.63 (d, *J* = 7.6 Hz, 2H), 7.46–7.39 (m, 3H), 7.34–7.23 (m, 2H), 7.01 (d, *J* = 8.2 Hz, 1H), 6.74 (d, *J* = 1.8 Hz, 1H), 2.44 (s, 3H).

^{13}C NMR (100 MHz, $CDCl_3$) δ 137.9, 135.1, 132.5, 129.5, 129.4, 129.0, 127.5, 125.0, 124.0, 120.3, 110.5, 99.5, 21.4.

HRMS *m*/*z* (ESI) calcd. for $C_{15}H_{14}N$ $[M+H]^+$: 208.1121; found: 208.1132.

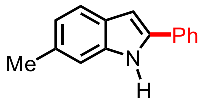

6-Methyl-2-phenyl-1*H*-indole (5d). The title compound was synthesized according to the General Procedure D using 6-methyl-1*H*-indole (13.1 mg, 0.1 mmol), diphenyliodonium

tetrafluoroborate (73.6 mg, 0.2 mmol), **Pd@Im-COF-2** (6.4 mg, 5 mol%), anhydrous DCE (1.0 mL), and H_2O (0.10 mL). The resulting mixture was degassed via three freeze-pump-thaw cycles and backfilled with N_2. Next, the reaction vessel was immersed in a water bath and stirred at 40 °C for 48 h. After reaction completion, the crude reaction mixture was purified by flash column chromatography using hexanes/ethyl acetate (20:1 v/v) as the eluent to give **5d** as a brown solid (11.2 mg, 54% yield).

^{1}H NMR (400 MHz, $CDCl_3$) δ 8.21 (br, 1H), 7.64 (dd, *J* = 8.2, 1.4 Hz, 2H), 7.51 (d, *J* = 8.0 Hz, 1H), 7.43 (t, *J* = 7.6 Hz, 2H), 7.30 (t, *J* = 7.4 Hz, 1H), 7.19 (s, 1H), 6.96 (dd, *J* = 8.0, 1.4 Hz, 1H), 6.83–6.74 (m, 1H), 2.47 (s, 3H).

^{13}C NMR (100 MHz, $CDCl_3$) δ 137.3, 137.2, 132.5, 132.2, 129.0, 127.4, 127.0, 124.9, 122.0, 120.3, 110.8, 99.8, 21.8.

HRMS *m/z* (ESI) calcd. for $C_{15}H_{14}N$ $[M+H]^+$: 208.1121; found: 208.1115.

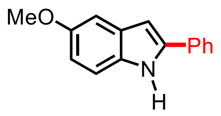

5-Methoxy-2-phenyl-1*H*-indole (5e). The title compound was synthesized according to the General Procedure D using 5-methoxy-1*H*-indole (14.7 mg, 0.1 mmol), diphenyliodonium tetrafluoroborate (73.6 mg, 0.2 mmol), **Pd@Im-COF-2** (6.4 mg, 5 mol%), anhydrous DCE (1.0 mL), and H_2O (75 μL). The resulting mixture was degassed via three freeze-pump-thaw cycles and backfilled with N_2. Next, the reaction vessel was immersed in a water bath and stirred at 40 °C for 48 h. After reaction completion, the crude reaction mixture was purified by flash column chromatography using hexanes/ethyl acetate (20:1 v/v) as the eluent to give **5e** as a white solid (15.8 mg, 71% yield).

^{1}H NMR (500 MHz, $CDCl_3$) δ 8.24 (br, 1H), 7.64 (d, *J* = 7.5 Hz, 2H), 7.43 (t, *J* = 7.5 Hz, 2H), 7.35–7.26 (m, 2H), 7.09 (s, 1H), 6.85 (dd, *J* = 8.5, 2.6 Hz, 1H), 6.75 (s, 1H), 3.86 (s, 3H).

^{13}C NMR (125 MHz, $CDCl_3$) δ 154.5, 138.6, 132.4, 132.0, 129.7, 129.0, 127.6, 125.0, 112.6, 111.6, 102.2, 99.8, 55.8.

HRMS *m/z* (ESI) calcd. for $C_{15}H_{13}NO$ $[M+H]^+$: 224.1070; found: 224.1078.

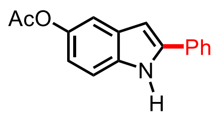

2-Phenyl-1*H*-indol-5-yl acetate (5f). The title compound was synthesized according to the General Procedure D using 1*H*-indol-5-yl acetate (17.5 mg, 0.1 mmol), diphenyliodonium tetrafluoroborate (73.6 mg, 0.2 mmol), **Pd@Im-COF-2** (6.4 mg, 5 mol%), anhydrous DCE (1.0 mL), and H_2O (75 µL). The resulting mixture was degassed via three freeze-pump-thaw cycles and backfilled with N_2. Next, the reaction vessel was immersed in a water bath and stirred at 40 °C for 48 h. After reaction completion, the crude reaction mixture was purified by flash column chromatography using hexanes/ethyl acetate (20:1 v/v) as the eluent to give **5f** as a white solid (19.3 mg, 77% yield).

^{1}H NMR (400 MHz, $CDCl_3$) δ 8.47 (br, 1H), 7.62 (d, *J* = 7.2 Hz, 2H), 7.42 (t, *J* = 7.6 Hz, 2H), 7.31 (t, *J* = 7.6 Hz, 1H), 7.28–7.23 (m, 2H), 6.84 (dd, *J* = 8.8, 2.4 Hz, 1H), 6.75 (s, 1H), 2.32 (s, 3H).

^{13}C NMR (100 MHz, $CDCl_3$) δ 170.6, 144.6, 139.2, 134.7, 132.1, 129.4, 129.0, 127.8, 125.2, 116.2, 112.5, 111.4, 100.0, 21.2.

HRMS *m/z* (ESI) calcd. for $C_{16}H_{14}NO_2$ $[M+H]^+$: 252.1019; found: 252.1010.

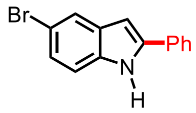

5-Bromo-2-phenyl-1*H*-indole (5g). The title compound was synthesized according to the General Procedure D using 5-bromo-1*H*-indole (19.5 mg, 0.1 mmol), diphenyliodonium tetrafluoroborate (73.6 mg, 0.2 mmol), **Pd@Im-COF-2** (2.6 mg, 2 mol%), anhydrous DCE (1.0 mL), and H_2O (75 µL). The resulting mixture was degassed via three freeze-pump-thaw cycles and backfilled with N_2. Next, the reaction vessel was immersed in a water bath and stirred at 40 °C for 48 h. After reaction completion, the crude reaction mixture was purified by flash column chromatography using hexanes/ethyl acetate (20:1 v/v) as the eluent to give **5g** as a white solid (20.1 mg, 74% yield).

^{1}H NMR (400 MHz, $CDCl_3$) δ 8.37 (br, 1H), 7.75 (s, 1H), 7.65 (d, *J* = 7.6 Hz, 2H), 7.45 (t, *J* = 7.6 Hz, 2H), 7.37–7.32 (m, 1H), 7.27 (s, 2H), 6.75 (d, *J* = 2.2 Hz, 1H).

^{13}C NMR (100 MHz, $CDCl_3$) δ 139.1, 135.4, 131.8, 131.0, 129.1, 128.1, 125.2, 125.1, 123.1, 113.4, 112.3, 99.4.

HRMS *m/z* (ESI) calcd. for $C_{14}H_{11}BrN$ $[M+H]^+$: 272.0069; found: 272.0067.

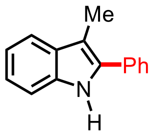

3-Methyl-2-phenyl-1*H*-indole (5h). The title compound was synthesized according to the General Procedure D using 3-methyl-1*H*-indole (13.1 mg, 0.1 mmol), diphenyliodonium tetrafluoroborate (73.6 mg, 0.2 mmol), **Pd@Im-COF-2** (6.4 mg, 5 mol%), anhydrous DCE (1.0 mL), and H_2O (0.10 mL). The resulting mixture was degassed via three freeze-pump-thaw cycles and backfilled with N_2. Next, the reaction vessel was immersed in a water bath and stirred at 40 °C for 48 h. After reaction completion, the crude reaction mixture was purified by flash column chromatography using hexanes/ethyl acetate (20:1 v/v) as the eluent to give **5h** as a brown solid (9.5 mg, 46% yield).

^{1}H NMR (400 MHz, $CDCl_3$) δ 7.99 (s, 1H), 7.58 (dd, *J* = 13.2, 7.8 Hz, 3H), 7.47 (t, *J* = 7.6 Hz, 2H), 7.35 (d, *J* = 7.6 Hz, 2H), 7.20 (t, *J* = 7.6 Hz, 1H), 7.14 (t, *J* = 7.4 Hz, 1H), 2.46 (s, 3H).

^{13}C NMR (100 MHz, $CDCl_3$) δ 135.8, 134.0, 133.3, 130.0, 128.8, 127.7, 127.3, 122.3, 119.5, 119.0, 110.6, 108.7, 9.6.

HRMS *m/z* (ESI) calcd. for $C_{15}H_{14}N$ $[M+H]^+$: 208.1121; found: 208.1117.

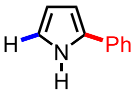

2-Phenyl-1*H*-pyrrole (7a). The title compound was synthesized according to the General Procedure E using 1*H*-pyrrole (67.0 mg, 1.0 mmol), diphenyliodonium tetrafluoroborate (36.8 mg, 0.1 mmol), **Pd@Im-COF-2** (6.4 mg, 5 mol%), anhydrous DCE (1.0 mL), and H_2O (75 μL). The resulting mixture was degassed via three freeze-pump-thaw cycles and backfilled with N_2. Next, the reaction vessel was immersed in a water bath and stirred at 40 °C for 48 h. After reaction completion, the ratio of the mono- and di-arylated products (mono:di > 10:1) was determined by ^{1}H NMR of the crude reaction mixture, which was then purified by flash column

chromatography using hexanes/ethyl acetate (10:1 v/v) as the eluent to give **7a** as a brown solid (10.3 mg, 72% yield).

^{1}**H NMR** (400 MHz, $CDCl_3$) δ 8.44 (br, 1H), 7.47 (dd, *J* = 8.2 Hz, 1.4 Hz, 2H), 7.36 (t, *J* = 7.8 Hz, 2H), 7.20 (t, *J* = 7.4 Hz, 1H), 6.86 (td, *J* = 2.8, 1.4 Hz, 1H), 6.53 (ddd, *J* = 4.0, 2.6, 1.4 Hz, 1H), 6.30 (q, *J* = 3.0 Hz, 1H).

^{13}**C NMR** (100 MHz, $CDCl_3$) δ 132.7, 132.1, 128.9, 126.2, 123.8, 118.8, 110.1, 105.9.

HRMS *m*/*z* (ESI) calcd. for $C_{10}H_{10}N$ $[M+H]^+$: 144.0808; found: 144.0798.

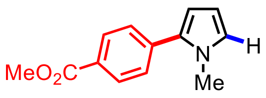

Methyl 4-(1-methyl-1*H*-pyrrol-2-yl)benzoate (7b). The title compound was synthesized according to the General Procedure E using 1-methyl-1*H*-pyrrole (81.1 mg, 1.0 mmol), bis(4-(methoxycarbonyl)phenyl)iodonium tetrafluoroborate (48.4 mg, 0.1 mmol), **Pd@Im-COF-2** (2.6 mg, 2 mol%), anhydrous DCE (1.0 mL), and H_2O (75 μL). The resulting mixture was degassed via three freeze-pump-thaw cycles and backfilled with N_2. Next, the reaction vessel was immersed in a water bath and stirred at 40 °C for 48 h. After reaction completion, the ratio of the mono- and di-arylated products (mono:di > 10:1) was determined by ^{1}H NMR of the crude reaction mixture, which was then purified by flash column chromatography using hexanes/ethyl acetate (10:1 v/v) as the eluent to give **7b** as a pale brown solid (18.7 mg, 87% yield).

^{1}**H NMR** (400 MHz, $CDCl_3$) δ 8.11–8.02 (m, 2H), 7.52–7.43 (m, 2H), 6.82–6.71 (m, 1H), 6.34 (dd, *J* = 3.8, 1.8 Hz, 1H), 6.22 (dd, *J* = 3.8, 2.8 Hz, 1H), 3.93 (s, 3H), 3.71 (s, 3H).

^{13}**C NMR** (100 MHz, $CDCl_3$) δ 167.0, 137.7, 133.5, 129.7, 127.90, 127.85, 125.1, 110.0, 108.3, 52.1, 35.4.

HRMS *m*/*z* (ESI) calcd. for $C_{13}H_{14}NO_2$ $[M+H]^+$: 216.1019; found: 216.1021.

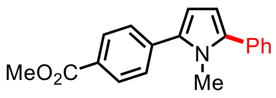

Methyl 4-(1-methyl-5-phenyl-1*H*-pyrrol-2-yl)benzoate (7c). The title compound was

synthesized according to the General Procedure E using methyl 4-(1-methyl-1*H*-pyrrol-2-yl)benzoate (21.5 mg, 0.1 mmol), diphenyliodonium tetrafluoroborate (73.6 mg, 0.2 mmol), **Pd@Im-COF-2** (2.6 mg, 2 mol%), anhydrous DCE (1.0 mL), and H_2O (75 µL). The resulting mixture was degassed via three freeze-pump-thaw cycles and backfilled with N_2. Next, the reaction vessel was immersed in a water bath and stirred at 40 °C for 48 h. After reaction completion, the crude reaction mixture was purified by flash column chromatography using hexanes/ethyl acetate (10:1 v/v) as the eluent to give **7c** as a pale-yellow solid (22.7 mg, 78% yield).

^{1}H NMR (400 MHz, $CDCl_3$) δ 8.08 (d, J = 8.2 Hz, 2H), 7.55 (d, J = 8.2 Hz, 2H), 7.45 (dt, J = 15.0, 7.2 Hz, 4H), 7.33 (t, J = 7.2 Hz, 1H), 6.43 (d, J = 3.8 Hz, 1H), 6.34 (d, J = 3.6 Hz, 1H), 3.94 (s, 3H), 3.64 (s, 3H).

^{13}C NMR (100 MHz, $CDCl_3$) δ 167.0, 138.4, 137.8, 135.8, 133.1, 129.8, 128.8, 128.5, 127.9, 127.1, 110.1, 109.2, 52.1, 34.6.

HRMS *m*/*z* (ESI) calcd. for $C_{19}H_{18}NO_2$ $[M+H]^+$: 292.1332; found: 2992.1331.

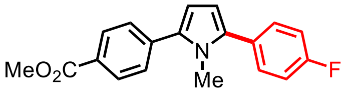

Methyl 4-(5-(4-fluorophenyl)-1-methyl-1*H*-pyrrol-2-yl)benzoate (7d). The title compound was synthesized according to the General Procedure E using methyl 4-(1-methyl-1*H*-pyrrol-2-yl)benzoate (21.5 mg, 0.1 mmol), bis(4-fluorophenyl)iodonium tetrafluoroborate (80.8 mg, 0.2 mmol), **Pd@Im-COF-2** (2.6 mg, 2 mol%), anhydrous DCE (1.0 mL), and H_2O (75 µL). The resulting mixture was degassed via three freeze-pump-thaw cycles and backfilled with N_2. Next, the reaction vessel was immersed in a water bath and stirred at 40 °C for 48 h. After reaction completion, the crude reaction mixture was purified by flash column chromatography using hexanes/ethyl acetate (20:1 v/v) as the eluent to give **7d** as a white solid (26.6 mg, 86% yield).

^{1}H NMR (400 MHz, $CDCl_3$) δ 8.09 (d, J = 8.4 Hz, 2H), 7.53 (d, J = 8.4 Hz, 2H), 7.43 (dd, J = 8.6, 5.4 Hz, 2H), 7.13 (t, J = 8.6 Hz, 2H), 6.42 (d, J = 3.8 Hz, 1H), 6.30 (d, J = 3.8 Hz, 1H), 3.94 (s, 3H), 3.61 (s, 3H).

^{13}C NMR (100 MHz, $CDCl_3$) δ 166.9, 162.1 (d, J = 247.1 Hz), 137.7, 137.2, 135.8, 130.5 (d, J = 8.0 Hz), 129.8, 129.3 (d, J = 3.2 Hz), 128.0, 127.9, 115.5 (d, J = 21.7 Hz), 110.1, 109.1, 52.1, 34.5.

^{19}F NMR (470 MHz, $CDCl_3$) δ -114.81.

HRMS *m/z* (ESI) calcd. for $C_{19}H_{17}FNO_2$ $[M+H]^+$: 310.1238; found: 310.1225.

6.4 Mechanistic studies

Synthesis of deuterated starting materials:

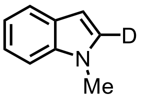

2-*Deutero*-1-methylindole (1a-*d*). The deuterated compound was prepared using a previously published method.[20] 1-Methylindole (0.70 g, 5.1 mmol) was placed under nitrogen atmosphere in a screwcap Schlenk tube with a magnetic stir bar. Anhydrous THF (20 mL) was added, and the reaction was allowed to stir until all the 1-methylindole is dissolved. The solution was cooled to 0 °C, and *n*-BuLi (1.6 M, 5.8 mL, 9.2 mmol) was added drop wise. The mixture was allowed to warm to room temperature and allowed to stir for an additional 60 minutes. Then 1.0 mL of D_2O was slowly added drop wise. After the solution was fully quenched with D_2O, it was extracted with ethyl acetate (2 × 20 mL). The resulting organic solution was dried over Na_2SO_4, and the solvent was removed. The final product was filtered over a silica gel pad to afford **1a**-*d* as a yellowish oil in 98% yield. According to the ^{1}H NMR the compound contains 100% deuterium at position 2.

^{1}H NMR (400 MHz, $CDCl_3$) δ 7.55 (d, J = 8.0 Hz, 1H), 7.25 (d, J = 8.2 Hz, 1H), 7.18–7.13 (m, 1H), 7.03 (t, J = 7.4 Hz, 1H), 6.40 (s, 1H), 3.70 (s, 3H).

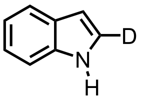

2-*Deutero*-1*H*-indole (4a-*d*). The deuterated compound was prepared using a previously published method.[20] 1*H*-indole (0.6g, 5.1 mmol) was placed under nitrogen atmosphere in a screwcap Schlenk tube with a magnetic stir bar. Anhydrous THF (20 mL) was added, and the reaction was allowed to stir until all the 1*H*-indole is dissolved. The solution was cooled to 0 °C, and *n*-BuLi (1.6 M, 5.8 mL, 9.2 mmol) was added drop wise. The mixture was allowed to warm to room temperature and allowed to stir for an additional 60 minutes. Then 1.0 mL of D_2O was slowly added drop wise. After the solution was fully quenched with D_2O, it was extracted with ethyl acetate (2 × 20 mL). The resulting organic solution was dried over Na_2SO_4, and the solvent

was removed. The final product was filtered over a silica gel pad to afford **4a**-*d* as a yellowish oil in 90% yield. According to the ^{1}H NMR the compound contains 100% deuterium at position 2.

^{1}H NMR (400 MHz, $CDCl_3$) δ 8.11 (s, 1H), 7.69 (d, *J* = 8.0 Hz, 1H), 7.41 (d, *J* = 8.0 Hz, 1H), 7.26–7.20 (m, 1H), 7.19–7.11 (m, 1H), 6.59 (s, 1H).

Determination of kinetic isotope effect (KIE):

The KIE in the C2-selective C–H arylation was investigated by the following pseudo-first-order rate experiments. The results are shown in Figure S57 and S58. The yield for each time point was determined by a separate reaction. Experiments for both the deuterated and the non-deuterated substrates were conducted in parallel.

Experimental procedure:

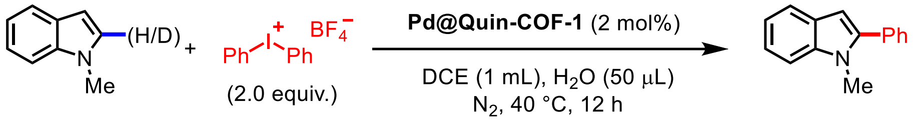

Six 10-mL Schlenk tubes equipped with a magnetic stirrer were fully dried (no longer the appearance of moisture inside the tubes), the tubes were then cooled to room temperature. In a separate dried flasks were made six solutions, three for the non-deuterated and three for the deuterated substrate. Each solution was prepared in the following manner: a 10-mL Schlenk tube equipped with a magnetic stirrer was loaded with **1a** or **1a**-*d* (0.1 mmol, 1 equiv.), **2a** (0.2 mmol, 2 equiv.), **Pd@Quin-COF-1** (2 mol%), anhydrous DCE (1.0 mL), and H_2O (50 µL). The resulting mixture was degassed via three freeze-pump-thaw cycles and backfilled with N_2. Next, the reaction vessel was immersed in a water bath and stirred at 40 °C for reaction. One set of reactions (one reaction containing deuterated and one containing non-deuterated substrate) was taken off the water bath at the desired time points. Subsequently, the stopped reactions were filtered and dried over Na_2SO_4, then the solvent was removed. The crude product was analyzed by ^{1}H NMR versus the internal standard (CH_2Br_2) (7 µL, 0.1 mmol).

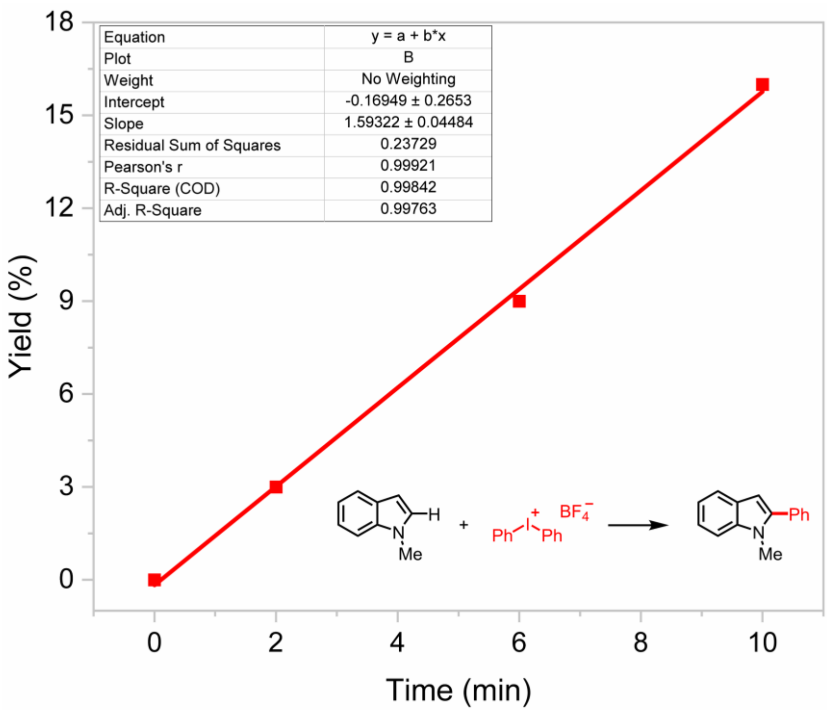

Figure S57. Initial rate studies for the C2-selective C–H arylation of **1a** with **2a**.

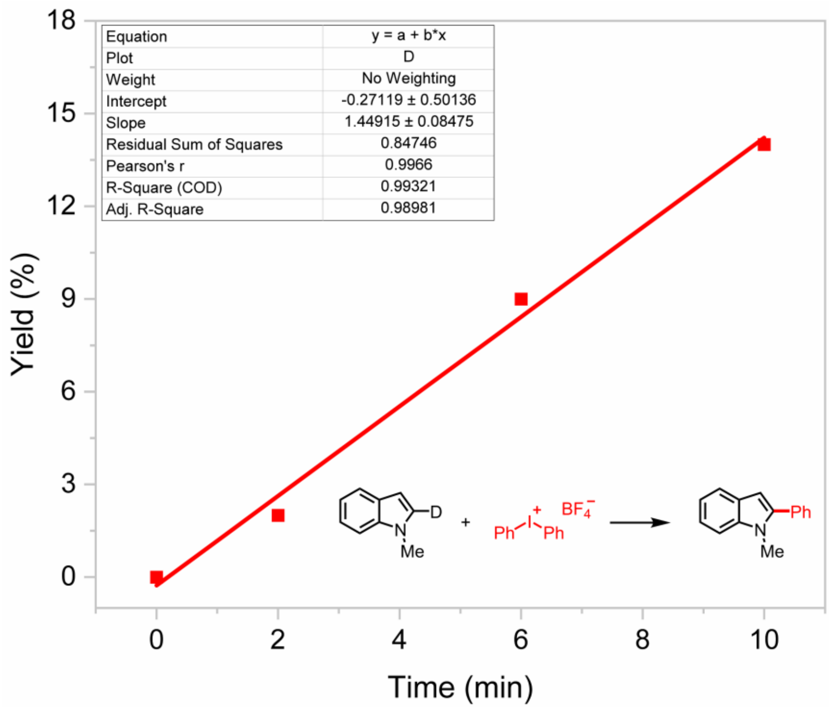

Figure S58. Initial rate studies for the C2-selective C–H arylation of **1a**-*d* with **2a**.

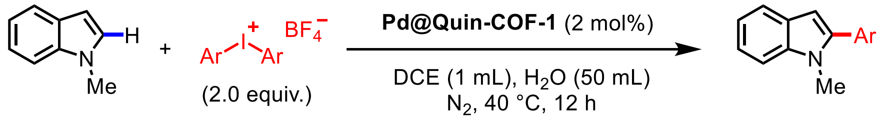

Eight 10-mL Schlenk tubes equipped with a magnetic stirrer were fully dried (no longer the appearance of moisture inside the tubes), the tubes were then cooled to room temperature. In a separate dried flasks were made six solutions, four for the di-*o*-tolyliodonium tetrafluoroborate and four for the bis(2-chlorophenyl)iodonium tetrafluoroborate substrate. Each solution was prepared in the following manner: a 10-mL Schlenk tube equipped with a magnetic stirrer was loaded with indole **1a** (0.1 mmol, 1 equiv.), diaryliodonium salt (0.2 mmol, 2 equiv.), **Pd@Quin-COF-1** (2 mol%), anhydrous DCE (1.0 mL), and H_2O (50 μL). The resulting mixture was degassed via three freeze-pump-thaw cycles and backfilled with N_2. Next, the reaction vessel was immersed in a water bath and stirred at 40 °C for reaction. One set of reactions (one reaction containing di-*o*-tolyliodonium tetrafluoroborate and one containing bis(2-chlorophenyl)iodonium tetrafluoroborate substrate) was taken off the water bath at the desired time points. Subsequently, the stopped reactions were filtered and dried over Na_2SO_4, then the solvent was removed. The crude product was analyzed by ^1H NMR versus the internal standard (CH_2Br_2) (7 μL, 0.1 mmol).

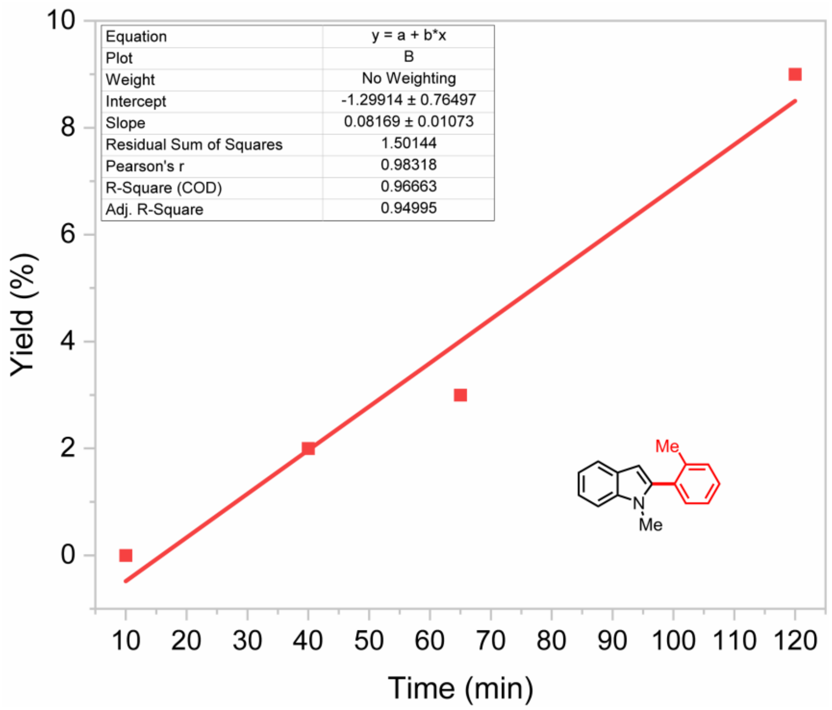

Figure S59. Initial rate studies for the C2-selective C–H arylation of **1a** with di-*o*-tolyliodonium tetrafluoroborate.

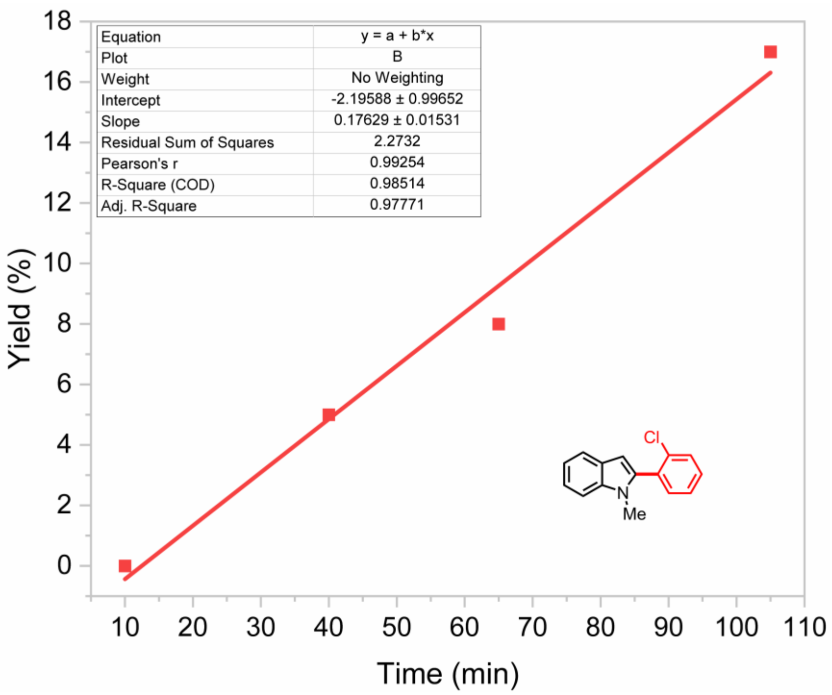

Figure S60. Initial rate studies for the C2-selective C–H arylation of **1a** with bis(2-chlorophenyl)iodonium tetrafluoroborate.

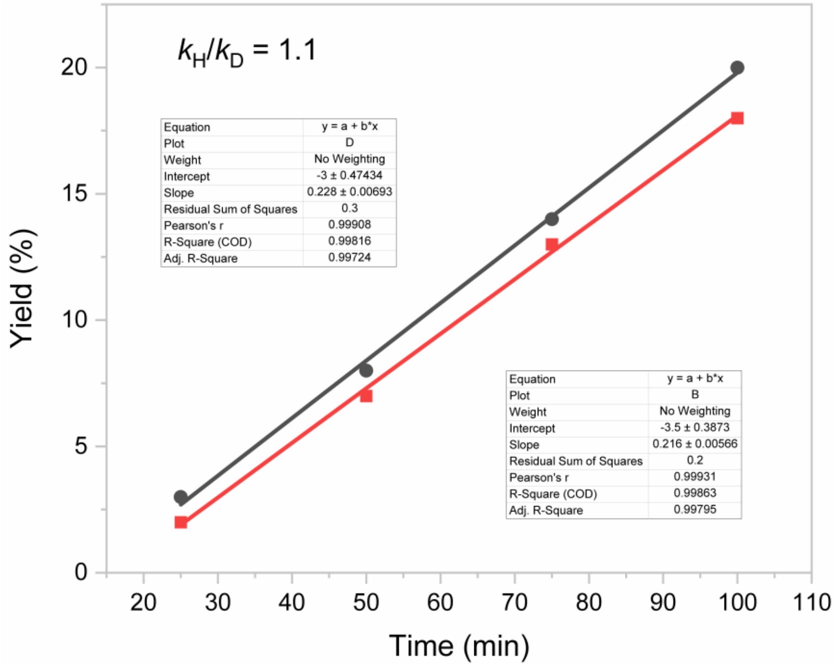

Figure S61. KIE of the heterogeneous palladium-catalyzed C–H arylation of free indoles with **Pd@Im-COF-2**.

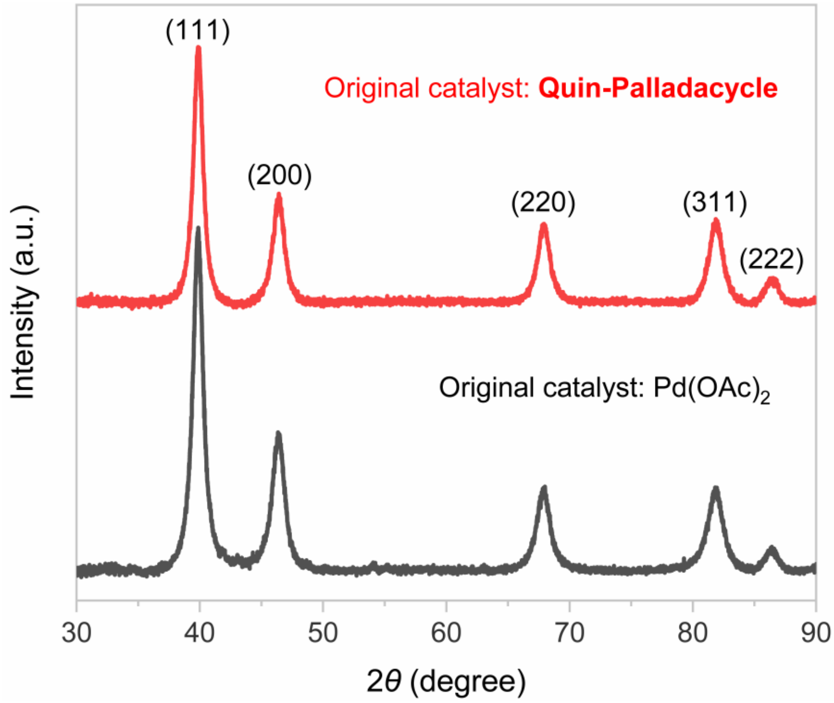

Figure S62. PXRD patterns of recycled palladium catalysts after the first run of homogeneous catalytic reactions.[21]

6.5 Recycling experiments of Pd@Quin-COF-1 and Pd@Im-COF-2

In the recycling experiments for the synthesis of **3a**, *N*-methyl indole (13.2 mg, 0.1 mmol), diphenyliodonium tetrafluoroborate (73.6 mg, 0.2 mmol), **Pd@Quin-COF-1** (8.0 mg, 2 mol%), anhydrous DCE (1.0 mL), and H_2O (50 μL) were used. The reaction mixture stirred at 40 °C under N_2 atmosphere for 12 h. After each cycle, the catalyst was dried and then used directly without further treatment. For the BET and TEM measurements, the recycled catalyst was washed with DCE and DCM, and dried at 70 °C overnight.

In the recycling experiments for the synthesis of **5a**, 1*H*-indole (11.7 mg, 0.1 mmol), diphenyliodonium tetrafluoroborate (73.6 mg, 0.2 mmol), **Pd@Im-COF-2** (6.4 mg, 5 mol%), anhydrous DCE (1.0 mL), and H_2O (75 μL) were used. The reaction mixture stirred at 40 °C under N_2 atmosphere for 24 h. After each cycle, the catalyst was dried and then used directly without further treatment.

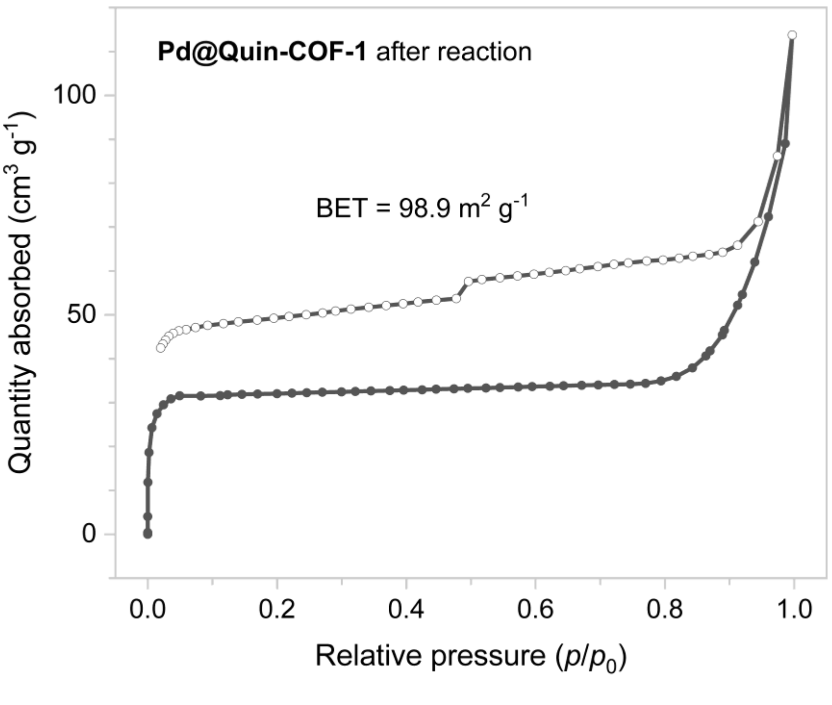

Figure S63. N_2 adsorption/desorption isotherms of **Pd@Quin-COF-1** after reaction recorded at 77 K.

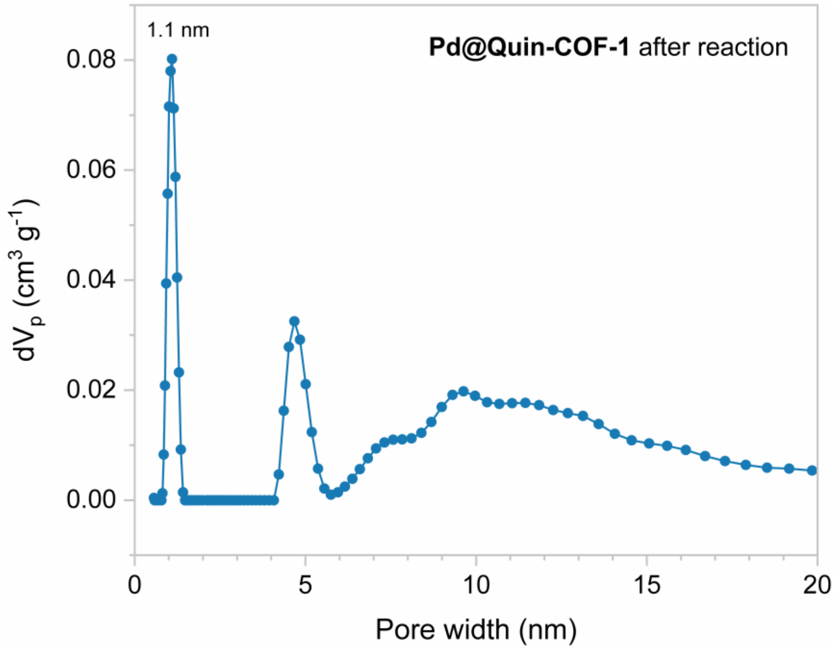

Figure S64. Pore size distribution profiles of **Pd@Quin-COF-1** after reaction.

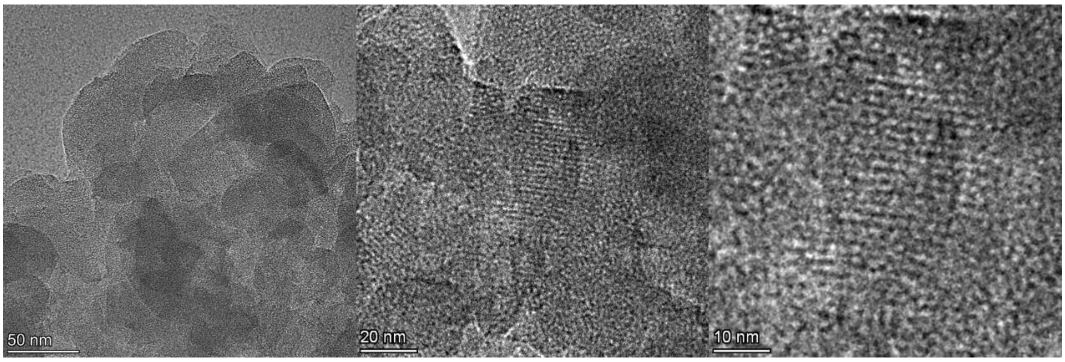

Figure S65. HR-TEM images of recycled **Pd@Quin-COF-1** after 5 runs.

Table S14. Palladium Loading Determination of Pd@Quin-COF-1 before and after Catalysis

Entry	Samples	Color	Loading of Pd (wt%)	Pd/N Ratio
1	freshly prepared	dark red	2.6	1:13
2	recycled after 5 runs	dark red	2.6	1:13

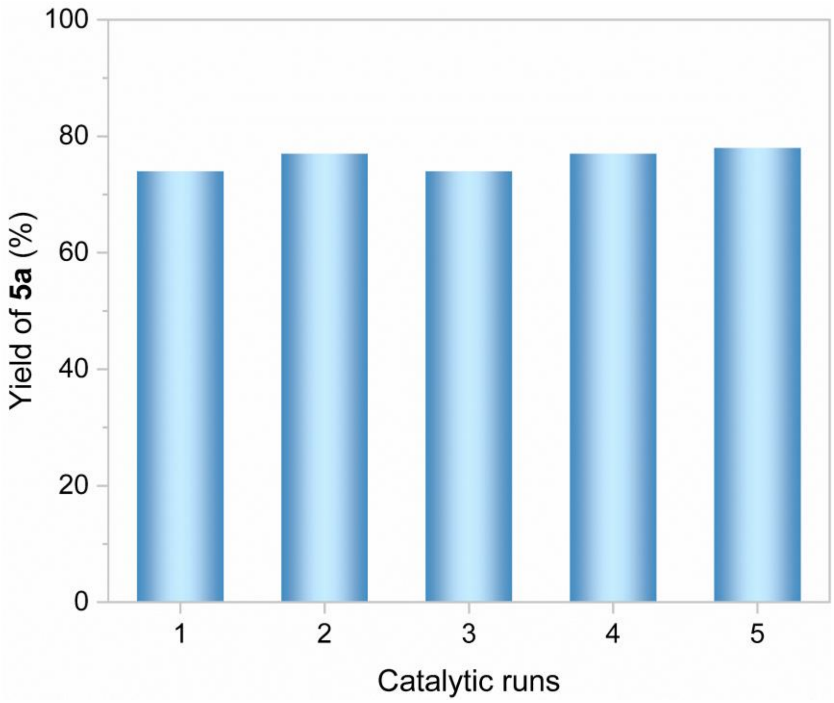

Figure S66. Recycling experiments for the synthesis of **5a**.

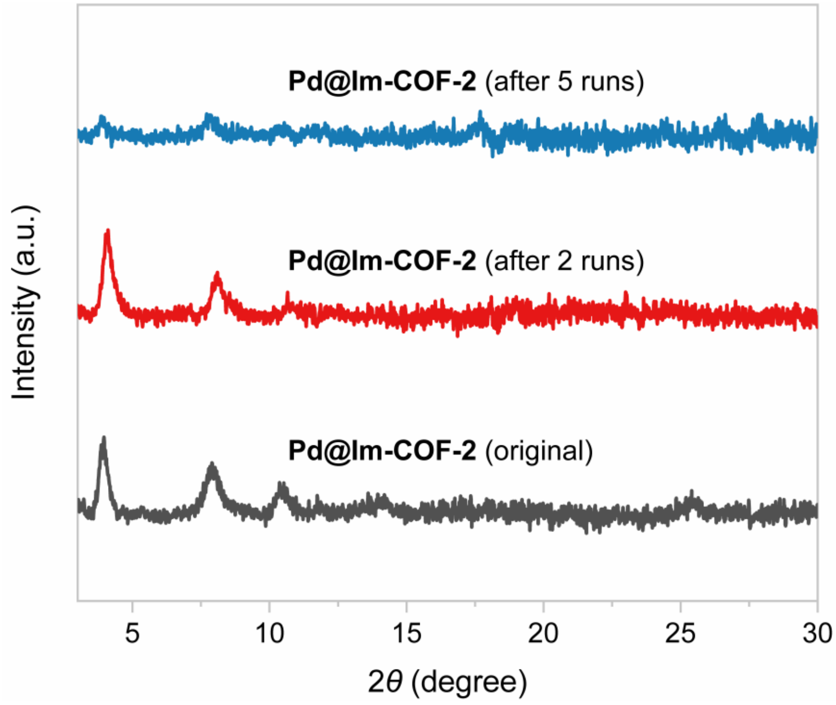

Figure S67. PXRD patterns of **Pd@Im-COF-2** before and after catalysis.

Table S15. Representative C–H Arylation of Indoles with Homogeneous and Heterogeneous Palladium Catalysts

Entry	Catalyst & Additive	Indole Substrate (R = Me or H)	Counter partner (equiv.)	Solvent	Temp. (°C)	TON (1st Run)	TON (2nd Run)	TON (3rd Run)	Ref.
1	2 mol% **Pd@Quin-COF-1**	Me	Ph_2IBF_4 (2.0)	DCE/ H_2O	40	48.5	48.5	46.5	This work
2	2 mol% **Pd@Im-COF-2**	H	Ph_2IBF_4 (2.0)	DCE/ H_2O	40	37	38.5	37	This work
3	5 mol% IMesPd(OAc)$_2$	Me	Ph_2IBF_4 (2.0)	AcOH	25	17.2	N.A.	N.A.	22
4	5 mol% IMesPd(OAc)$_2$	H	Ph_2IBF_4 (2.0)	AcOH	25	16.2	N.A.	N.A.	22
5	2.5 mol% Pd^0-AmP-MCF	Me	Ph_2IBF_4 (2.0)	H_2O	25	32			23
6	2.5 mol% Pd^0-AmP-MCF	H	Ph_2IBF_4 (2.0)	H_2O	25	36.4	32[a]	26.8[a]	23
7	5 mol% Pd/C	H	Ph_2IBF_4 (1.4)	EtOH	60	8			24
8	10 mol% Pd/C, Polarclean	Me	Ph_2IBF_4 (1.25)	H_2O	70	9.2			25
9	10 mol% Pd/C, Polarclean	H	Ph_2IBF_4 (1.25)	H_2O	70	9.3	9.3	9.3	25
10	5 mol% Pd(OAc)$_2$, Ag_2O (0.75 equiv.) *o*-$NO_2C_6H_4CO_2H$ (1.5 equiv.)	Me	PhI (2.0)	DMF	25	18.4	N.A.	N.A.	26
11	5 mol% Pd(OAc)$_2$, Ag_2O (0.75 equiv.) *o*-$NO_2C_6H_4CO_2H$ (1.5 equiv.)	H	PhI (2.0)	DMF	50	12.2	N.A.	N.A.	26

[a]TON values was calculated based on ^1H NMR yields rather than isolated yields.

6.6 Large-scale synthetic application

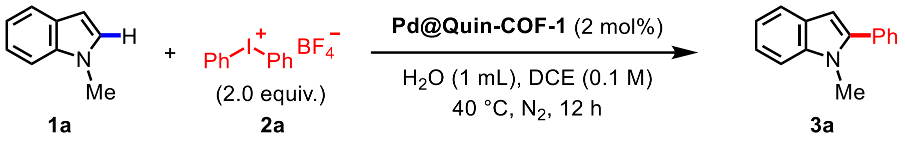

Experiment procedure: A 100-mL Schlenk tube equipped with a magnetic stirrer was loaded with *N*-methyl indole **1a** (2.0 mmol, 1 equiv.), diphenyliodonium tetrafluoroborate **2a** (4.0 mmol, 2 equiv.), **Pd@Quin-COF-1** (160 mg, 2 mol%), anhydrous DCE (0.1 M, 20 mL), and H_2O (1 mL). The resulting mixture was degassed via three freeze-pump-thaw cycles and backfilled with N_2. Next, the reaction vessel was immersed in a water bath and stirred at 40 °C for 12 h. After reaction completion, the crude reaction mixture was concentrated under vacuum and purified by column chromatography to give **3a** with an isolated yield of 73% (302.4 mg).

6.7 Investigation of heterogeneous catalysis with low palladium loadings

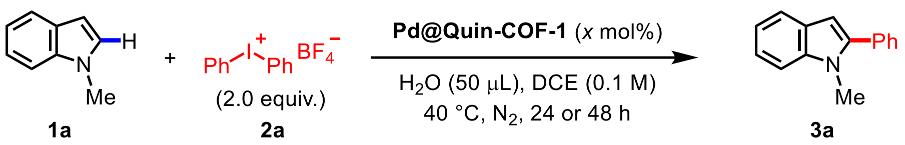

Experiment procedures: A 10-mL Schlenk tube equipped with a magnetic stirrer was loaded with *N*-methyl indole **1a** (0.1 mmol, 1 equiv.), diphenyliodonium tetrafluoroborate **2a** (0.2 mmol, 2 equiv.), **Pd@Quin-COF-1** (4 mg, 1 mol%), anhydrous DCE (1 mL), and H_2O (50 μL). The resulting mixture was degassed via three freeze-pump-thaw cycles and backfilled with N_2. Next, the reaction vessel was immersed in a water bath and stirred at 40 °C for 24 h. After reaction completion, the crude reaction mixture was concentrated under vacuum and purified by column chromatography to give **3a** with isolated yield of 94% (19.5 mg).

A 10-mL Schlenk tube equipped with a magnetic stirrer was loaded with *N*-methyl indole **1a** (0.1 mmol, 1 equiv.), diphenyliodonium tetrafluoroborate **2a** (0.2 mmol, 2 equiv.), **Pd@Quin-COF-1** (2 mg, 0.5 mol%), anhydrous DCE (1 mL), and H_2O (50 μL). The resulting mixture was degassed via three freeze-pump-thaw cycles and backfilled with N_2. Next, the reaction vessel was immersed in a water bath and stirred at 40 °C for 48 h. After reaction completion, the crude reaction mixture was concentrated under vacuum and purified by column chromatography to give **3a** with isolated yield of 79% (16.4 mg).

6.8 Investigation of C–H bromination and acetoxylation with Pd@Quin-COF-1

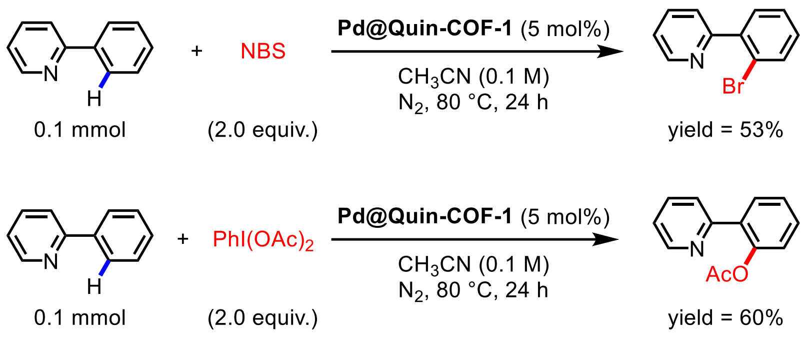

Experiment procedure: A 10-mL Schlenk tube equipped with a magnetic stirrer was loaded with 2-phenylpyridine (0.1 mmol, 15.5 mg), *N*-bromosuccinimide or (diacetoxyiodo)benzene (0.2 mmol, 2 equiv.), **Pd@Quin-COF-1** (20 mg, 5 mol%), and anhydrous acetonitrile (1.0 mL). The resulting mixture was degassed via three freeze-pump-thaw cycles and backfilled with N_2. Next, the reaction vessel was immersed in an oil bath and stirred at 80 °C for 24 h. After reaction completion, the crude reaction mixture was concentrated under vacuum and purified by column chromatography to give 2-(2-bromophenyl)pyridine or 2-(pyridin-2-yl)phenyl acetate in an isolated yield of 53% (12.3 mg) or 60% (12.8 mg).

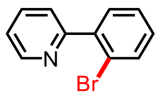

2-(2-Bromophenyl)pyridine. **^{1}H NMR** (500 MHz, $CDCl_3$) δ 8.71 (d, J = 4.5 Hz, 1H), 7.76 (t, J = 7.5 Hz, 1H), 7.67 (d, J = 7.5 Hz, 1H), 7.60 (d, J = 8.0 Hz, 1H), 7.53 (d, J = 7.5 Hz, 1H), 7.40 (t, J = 7.5 Hz, 1H), 7.33 – 7.22 (m, 2H).

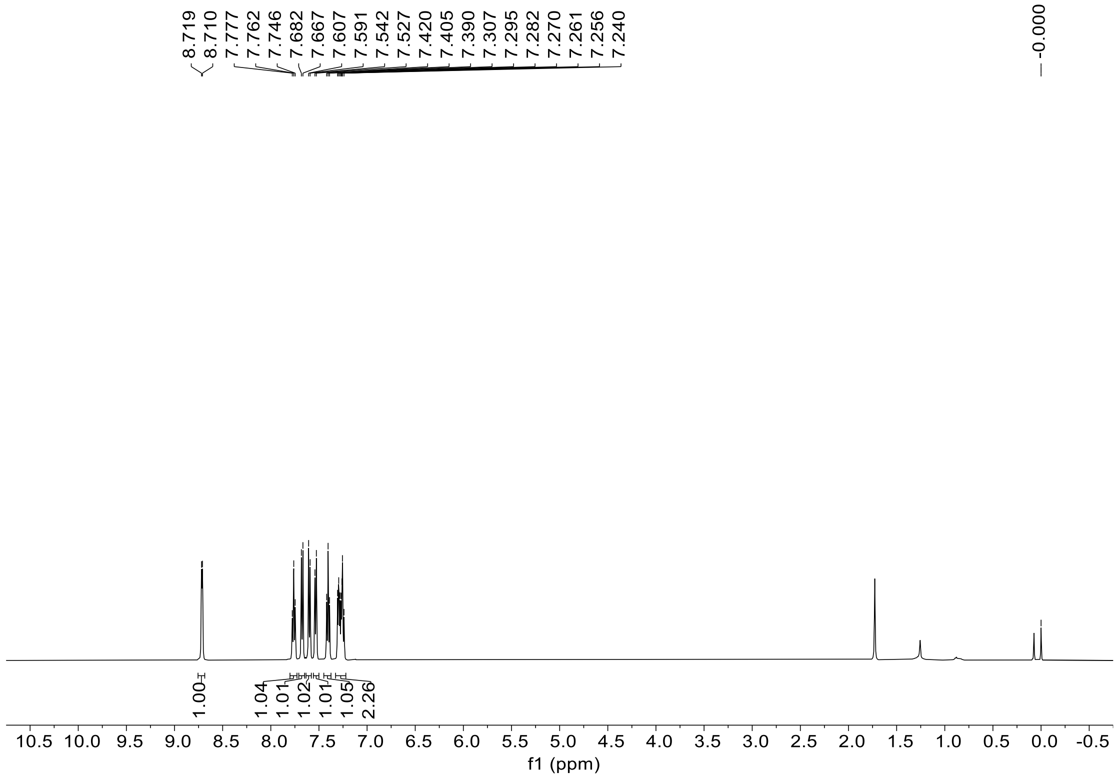

Figure S68. ^{1}H NMR spectrum of 2-(2-bromophenyl)pyridine.

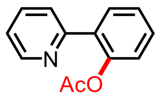

2-(Pyridin-2-yl)phenyl acetate. **^{1}H NMR** (400 MHz, $CDCl_3$) δ 8.69 (d, *J* = 3.2 Hz, 1H), 7.78 – 7.66 (m, 2H), 7.54 (d, *J* = 7.6 Hz, 1H), 7.43 (t, *J* = 6.4 Hz, 1H), 7.35 (t, *J* = 7.2 Hz, 1H), 7.28 – 7.22 (m, 1H), 7.16 (d, *J* = 8.0 Hz, 1H), 2.17 (s, 3H).

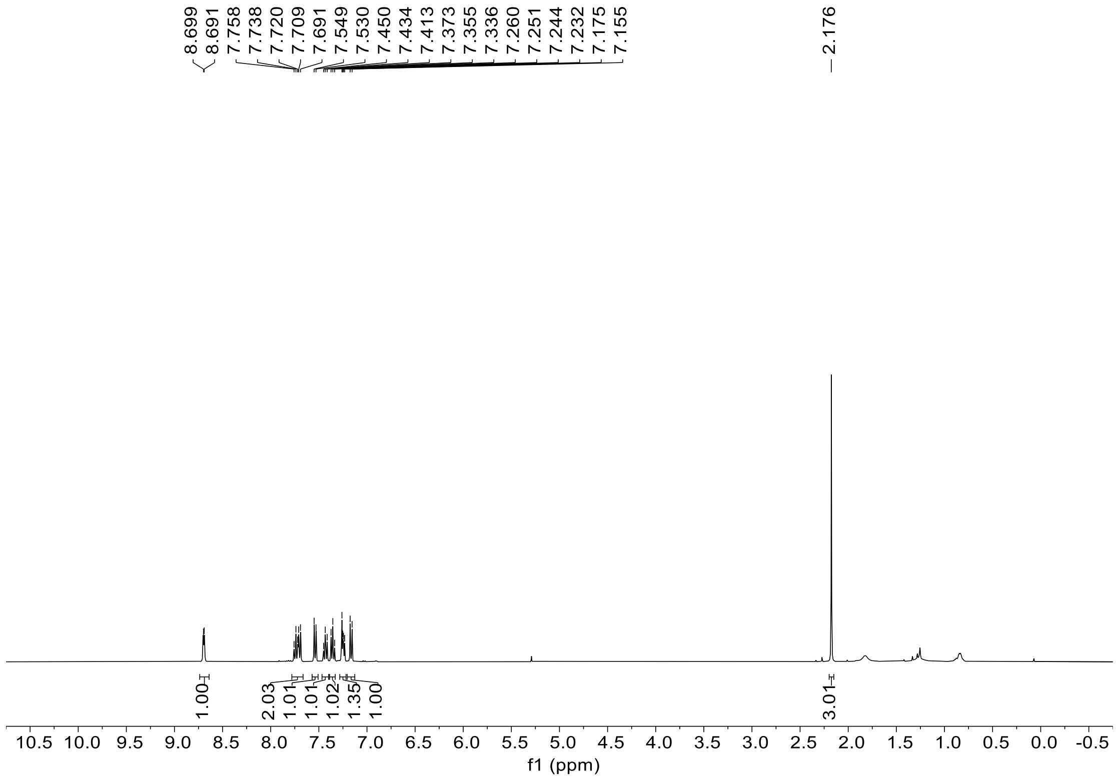

Figure S69. ^{1}H NMR spectrum of 2-(pyridin-2-yl)phenyl acetate.

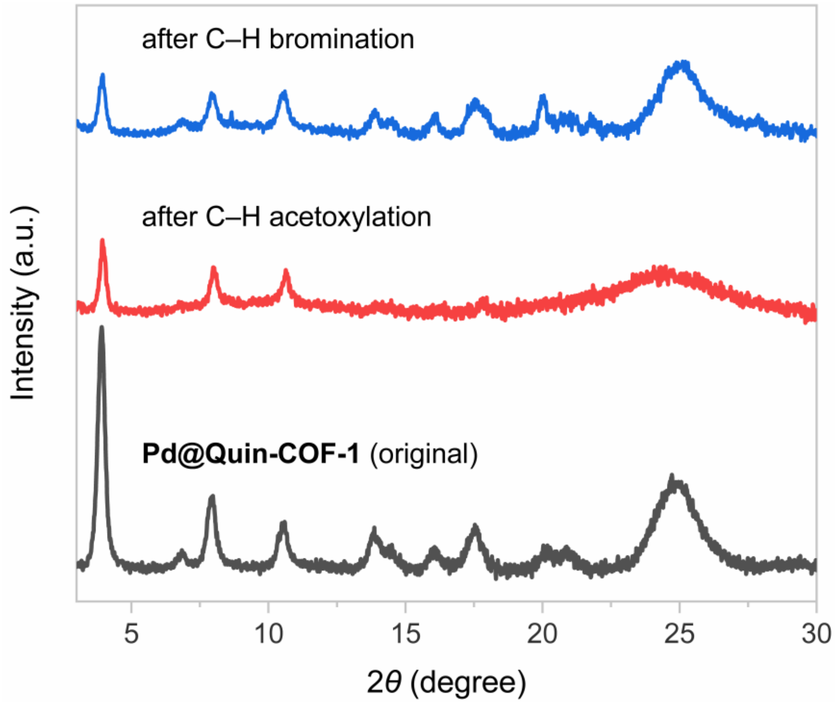

Figure S70. PXRD patterns of **Pd@Quin-COF-1** recycled from C–H bromination and acetoxylation.

7. References

1. Braunecker, W. A.; Hurst, K. E.; Ray, K. G.; Owczarczyk, Z. R.; Martinez, M. B.; Leick, N.; Keuhlen, A.; Sellinger, A.; Johnson, J. C. Phenyl/Perfluorophenyl Stacking Interactions Enhance Structural Order in Two-Dimensional Covalent Organic Frameworks. *Cryst. Growth Des.* **2018**, *18,* 4160−4166.
2. Ren, X.-R.; Bai, B.; Zhang, Q.; Hao, Q.; Guo, Y.; Wan, L.-J.; Wang, D. Constructing Stable Chromenoquinoline-Based Covalent Organic Frameworks via Intramolecular Povarov Reaction. *J. Am. Chem. Soc.* **2022**, *144*, 2488−2494.
3. (a) Zhai, L.; Huang, N.; Xu, H.; Chen, Q.; Jiang, D. A Backbone Design Principle for Covalent Organic Frameworks: the Impact of Weakly Interacting Units on CO_2 Adsorption. *Chem. Commum*. **2017**, *53*, 4242–4245. (b) Ding, S.-Y.; Gao, J.; Wang, Q.; Zhang, Y.; Song, W.-G.; Su, C.-Y.; Wang, W. Construction of Covalent Organic Framework for Catalysis: Pd/COF-LZU1 in Suzuki-Miyaura Coupling Reaction. *J. Am. Chem. Soc.* **2011**, *133*, 19816–19822. (c) De la Peña Ruigómez, A.; Rodríguez-San-Miguel, D.; Stylianou, K. C.; Cavallini, M.; Gentili, D.; Liscio, F.; Milita, S.; Roscioni, O. M.; Ruiz-González, M. L.; Carbonell, C.; Maspoch, D.; Mas-Ballesté, R.; Segura, J. L.; Zamora, F. Direct On-Surface Patterning of a Crystalline Laminar Covalent Organic Framework Synthesized at Room Temperature. *Chem. - Eur. J.* **2015**, *21*, 10666–10670. (d) Auras, F.; Ascherl, L.; Hakimioun, A. H.; Margraf, J. T.; Hanusch, F. C.; Reuter, S.; Bessinger, D.; Doblinger, M.; Hettstedt, C.; Karaghiosoff, K.; Herbert, S.; Knochel, P.; Clark, T.; Bein, T. Synchronized Offset Stacking: A Concept for Growing Large-Domain and Highly Crystalline 2D Covalent Organic Frameworks. *J. Am. Chem. Soc.* **2016**, *138*, 16703–16710. (e) Vardhan, H.; Al-Enizi, A. M.; Nafady, A.; Pan, Y.; Yang, Z.; Gutiérrez, H. R.; Han, X.; Ma, S. Single-Pore versus Dual-Pore Bipyridine-Based Covalent–Organic Frameworks: An Insight into the Heterogeneous Catalytic Activity for Selective C–H Functionalization. *Small* **2021**, *17*, 200397.
4. Gutierrez, M. A.; Newkome, G. R.; Selbin, J. Cyclometallation. Palladium 2-Arylpyridine Complexes. *J. Organomet. Chem*. **1980**, *202*, 341–350.
5. Ramesh, S.; Gaddam, V.; Nagarajan R. A Flexible Approach to the Chromenoquinolines under Copper/Lewis Acid Catalysis. *Synlett* **2010**, *5*, 757–760.
6. Chan, S.-J.; Kao, J.-C.; Chou, P.-J.; Lo, Y.-C.; Chou, J.-P.; Huang, M. H. 4-Nitrophenylacetylene-Modified Cu_2O Cubes and Rhombic Dodecahedra Showing Superior Photocatalytic Activity through Surface Band Structure Modulation. *J. Mater. Chem. C* **2022**, *10*, 8422–8431.
7. VandeVondele, J.; Krack, M.; Mohamed, F.; Parrinello, M.; Chassaing, T.; Hutter, J. Quickstep: Fast and Accurate Density Functional Calculations Using a Mixed Gaussian and Plane Waves Approach. *Comput. Phys. Commun*. **2005**, *167*, 103–128.
8. Goedecker, S.; Teter, M.; Hutter, J. Separable Dual-Space Gaussian Pseudopotentials. *Phys. Rev. B* **1996**, *54*, 1703–1710.
9. Hartwigsen, C.; Goedecker, S.; Hutter, J. Relativistic Separable Dual-Space Gaussian Pseudopotentials from H to Rn. *Phys. Rev. B* **1998**, *58*, 3641–3662.
10. Krack, M.; Parrinello, M. All-electron *ab-initio* Molecular Dynamics. *Phys. Chem. Chem. Phys*. **2000**, *2*, 2105–2112.
11. VandeVondele, J.; Hutter, J. Gaussian Basis Sets for Accurate Calculations on Molecular Systems in Gas and Condensed Phases. *J. Chem. Phys*. **2007**, *127*, 114105.

12. Perdew, J. P.; Burke, K.; Ernzerhof, M. Generalized Gradient Approximation Made Simple. *Phys. Rev. Lett*. **1996**, *77*, 3865.
13. Grimme, S.; Antony, J.; Ehrlich, S.; Krieg, H. A Consistent and Accurate *ab initio* Parametrization of Density Functional Dispersion Correction (DFT-D) for the 94 Elements H-Pu. *J. Chem. Phys*. **2010**, *132*, 154104.
14. Nickerl, G.; Leistner, M.; Helten.; Bon, V.; Senkovska, I.; Kaskel, S. Integration of Accessible Secondary Metal Sites into MOFs for H_2S Removal. *Inorg. Chem. Front*. **2014**, *1*, 325–330.
15. Bloch, E. D.; Britt, D.; Lee, C.; Doonan, C. J.; Uribe-Romo, F. J.; Furukawa, H. Long, J. R.; Yaghi, O. M. Metal Insertion in a Microporous Metal−Organic Framework Lined with 2,2'-Bipyridine. *J. Am. Chem. Soc*. **2010**, *132*, 14382–14384.
16. Monastyrskyi, A.; Namelikonda, N. K.; Manetsch, R. Metal-Free Arylation of Ethyl Acetoacetate with Hypervalent Diaryliodonium Salts: an Immediate Access to Diverse 3-Aryl-4(1*H*)-quinolones. *J. Org. Chem.* **2015**, *80*, 2513–2520.
17. Bielawski, M., Aili, D., Olofsson, B. Regiospecific One-Pot Synthesis of Diaryliodonium Tetrafluoroborates from Arylboronic Acids and Aryl Iodides. *J. Org. Chem.* **2008**, *73*, 4602–4607.
18. Beaud, R.; J. Phipps, R.; Gaunt, M. J. Enantioselective Cu-Catalyzed Arylation of Secondary Phosphine Oxides with Diaryliodonium Salts toward the Synthesis of P-Chiral Phosphines. *J. Am. Chem. Soc.* **2016**, *138*, 13183–13186.
19. Olguín-Uribe, S.; Mijangos, M. V.; Amador-Sánchez, Y. A.; Sánchez-Carmona, M. A.; Miranda, L. D. Expedited Synthesis of Matrine Analogues through an Oxidative Cascade Addition/Double-Cyclization Radical Process. *Eur. J. Org. Chem.* **2017**, *2017*, 2481–2485.
20. Zhang, Y.; Lin, Z.; Ackermann, L. Electrochemical C−H Amidation of Heteroarenes with *N*-Alkyl Sulfonamides in Aqueous Medium. *Chem. - Eur. J.* **2021**, *27*, 242–246.
21. Xu, L.; Wu, X.-C.; Zhu, J.-J. Green Preparation and Catalytic Application of Pd Nanoparticles. *Nanotechnology* **2008**, *19*. 305603.
22. Deprez, N. R.; Kalyani, D.; Krause, A.; Sanford, M. S. Room Temperature Palladium-Catalyzed 2-Arylation of Indoles. *J. Am. Chem. Soc.* **2006**, *128,* 4972–4973.
23. Malmgren, J.; Nagendiran, A.; Tai, C.-K.; Bäckvall, J.-E.; Olofsson, B. C-2 Selective Arylation of Indoles with Heterogeneous Nanopalladium and Diaryliodonium Salts. *Chem. - Eur. J.* **2014**, *20*, 13531–13535.
24. Tang, D.-T. D.; Collins, K. D.; Ernst, J. B.; Glorius, F. Pd/C as a Catalyst for Completely Regioselective C–H Functionalization of Thiophenes under Mild Conditions. *Angew. Chem., Int. Ed.* **2014**, *53*, 1809–1813.
25. Campana, F.; Massaccesi, B. M.; Santoro, S.; Piermatti, O.; Vaccaro, L. Polarclean/Water as a Safe and Recoverable Medium for Selective C2-Arylation of Indoles Catalyzed by Pd/C. *ACS Sustainable Chem. Eng.* **2020**, *8*, 16441–16450.
26. Lebrasseur, N.; Larrosa, I. Room Temperature and Phosphine Free Palladium Catalyzed Direct C-2 Arylation of Indoles. *J. Am. Chem. Soc.* **2008**, *130*, 2926–2927.

8. NMR spectra

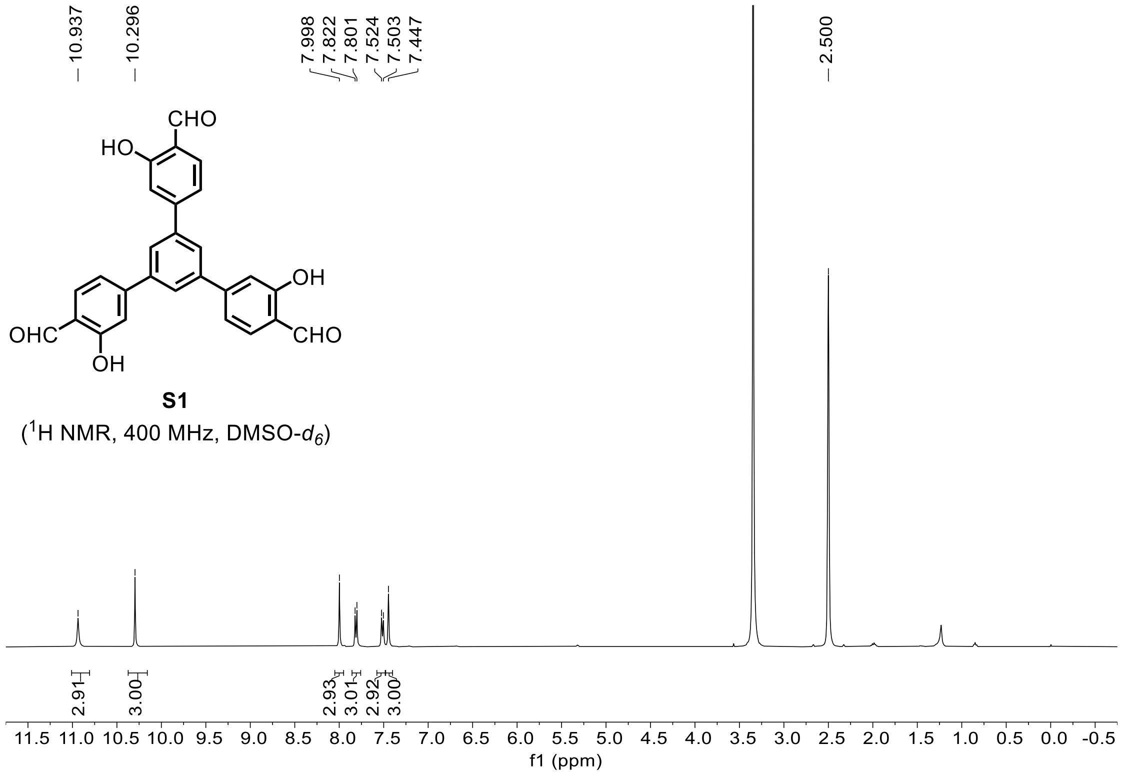

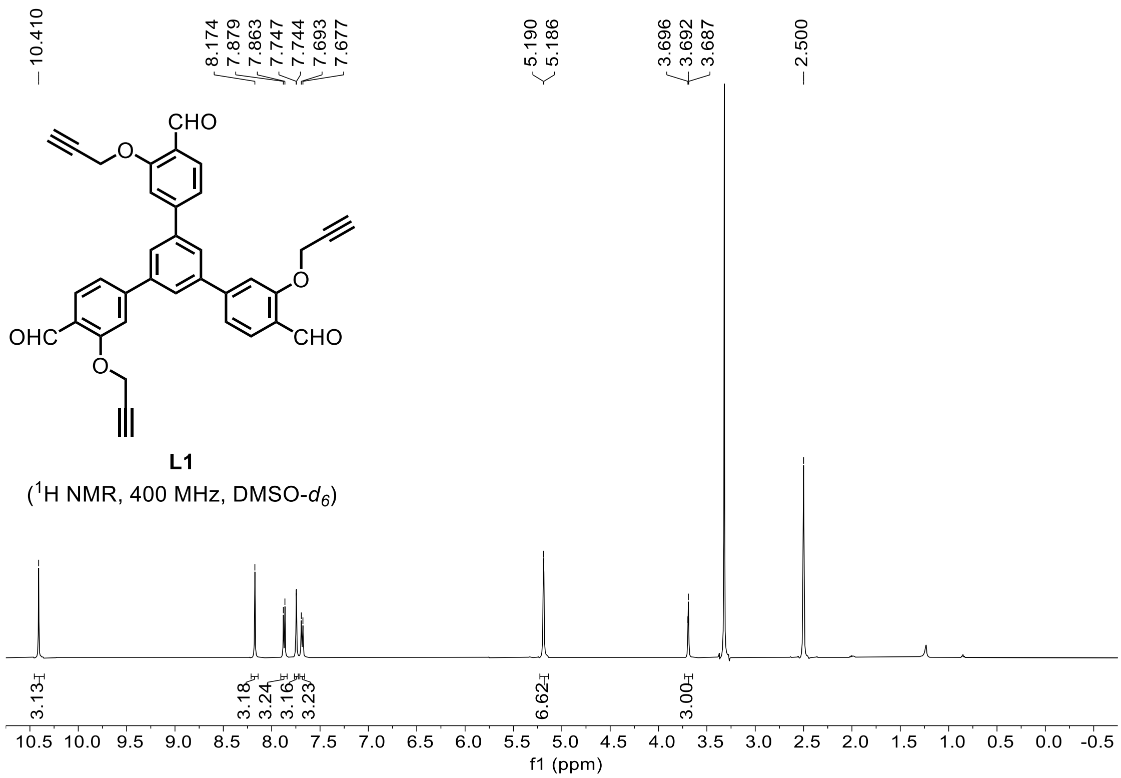
10.410
8.174
7.879
7.863
7.747
7.744
7.693
7.677
5.190
5.186
3.696
3.692
3.687
2.500
CHO
O
OHC
CHO
L1
(^{1}H NMR, 400 MHz, DMSO-d_6)
3.13
3.18
3.24
3.16
3.23
6.62
3.00
10.5 10.0 9.5 9.0 8.5 8.0 7.5 7.0 6.5 6.0 5.5 5.0 4.5 4.0 3.5 3.0 2.5 2.0 1.5 1.0 0.5 0.0 -0.5
f1 (ppm)

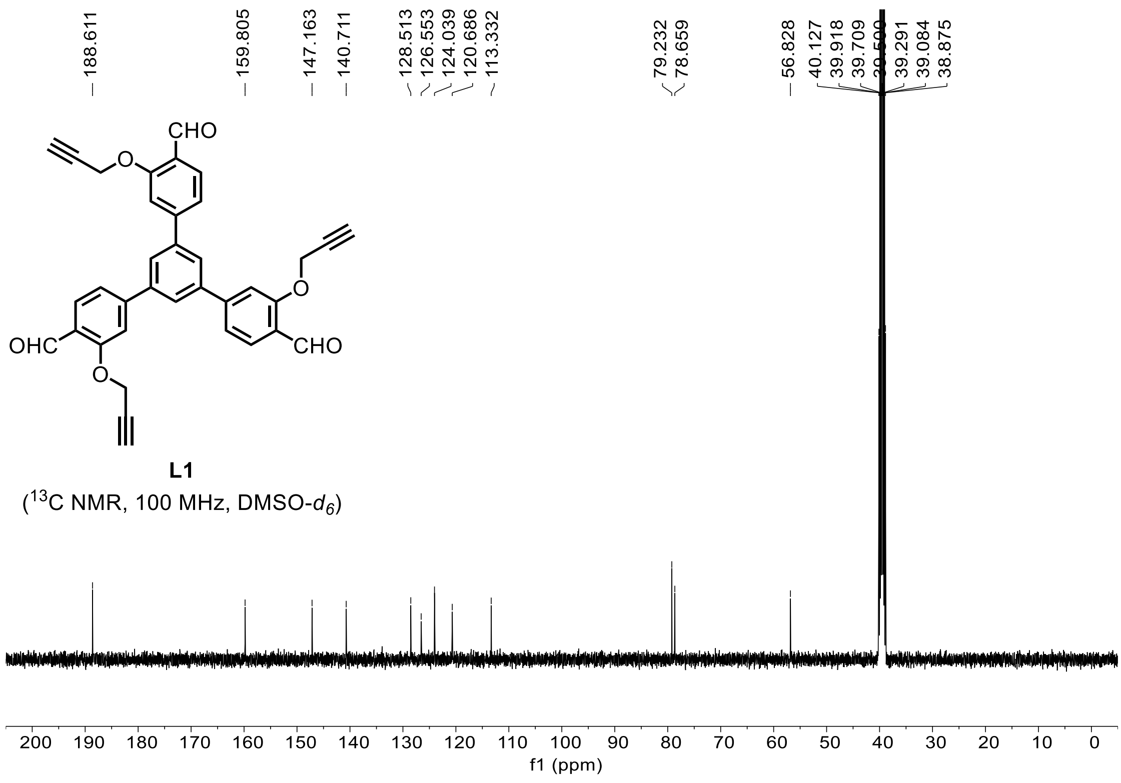
188.611
159.805
147.163
140.711
128.513
126.553
124.039
120.686
113.332
79.232
78.659
56.828
40.127
39.918
39.709
39.291
39.084
38.875
CHO
O
OHC
CHO
L1
(^{13}C NMR, 100 MHz, DMSO-d_6)
200 190 180 170 160 150 140 130 120 110 100 90 80 70 60 50 40 30 20 10 0
f1 (ppm)

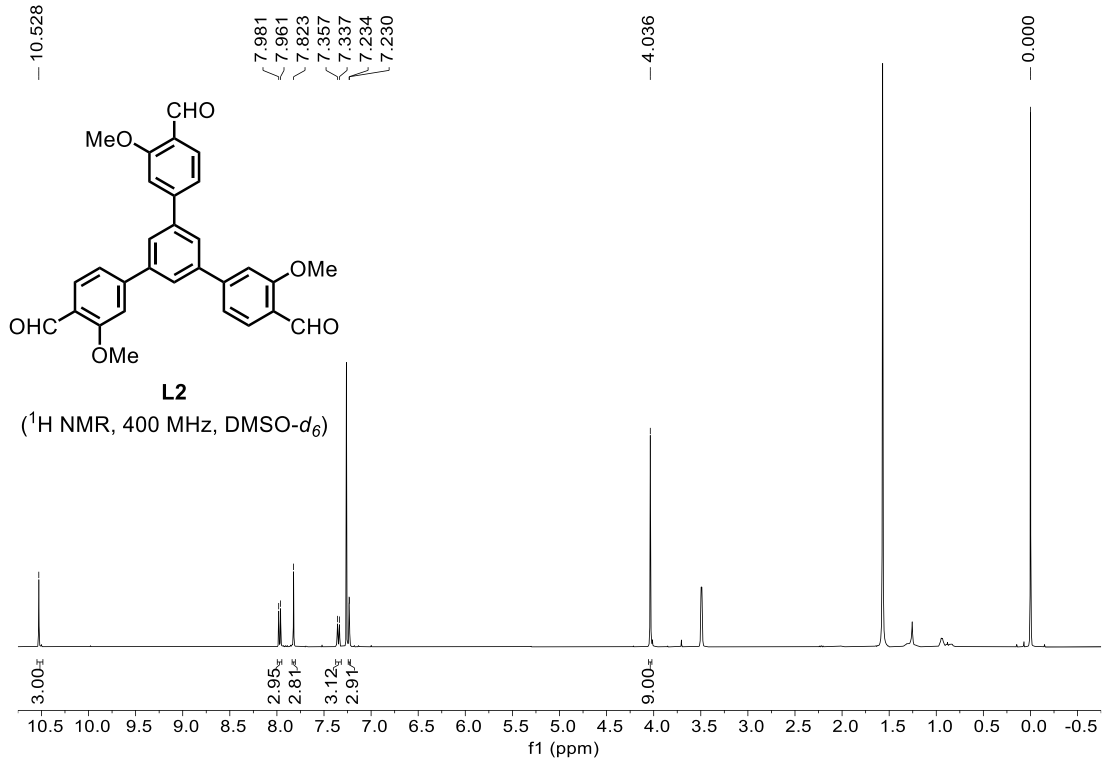

L2

(^{1}H NMR, 400 MHz, DMSO-d_6)

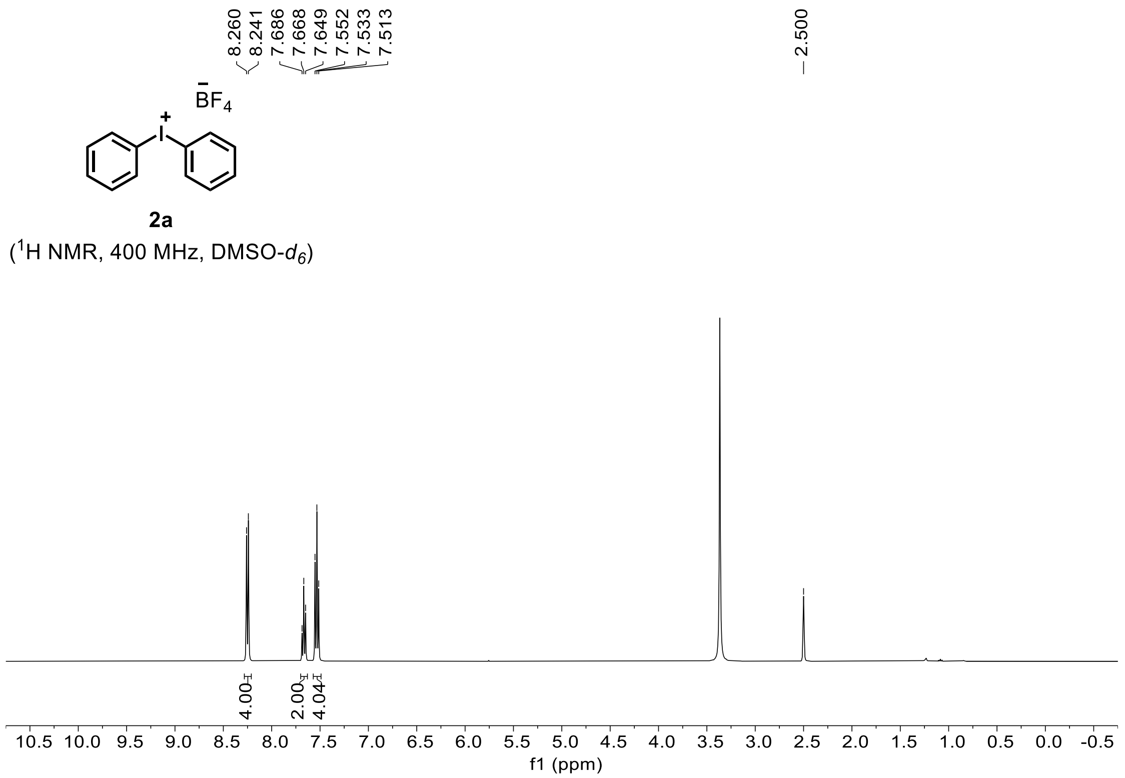

2a

(^{1}H NMR, 400 MHz, DMSO-d_6)

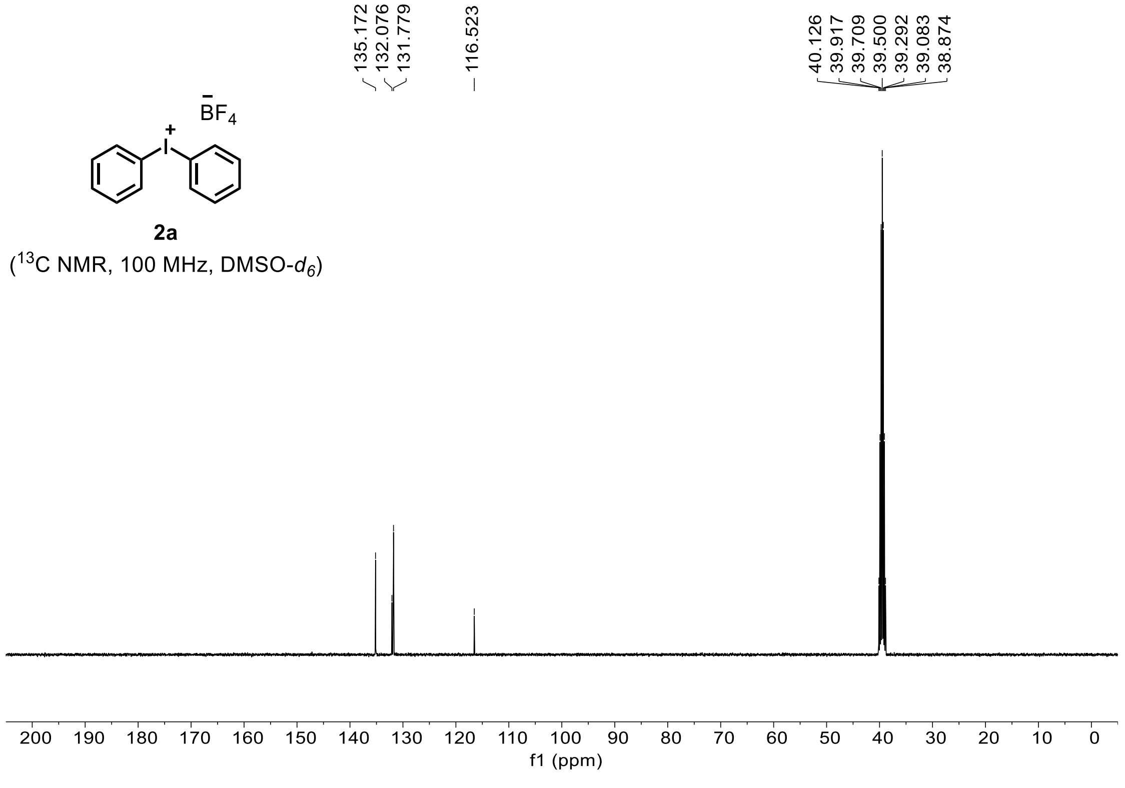

2a

(^{13}C NMR, 100 MHz, DMSO-d_6)

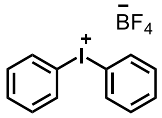

2a

(^{19}F NMR, 376 MHz, DMSO-d_6)

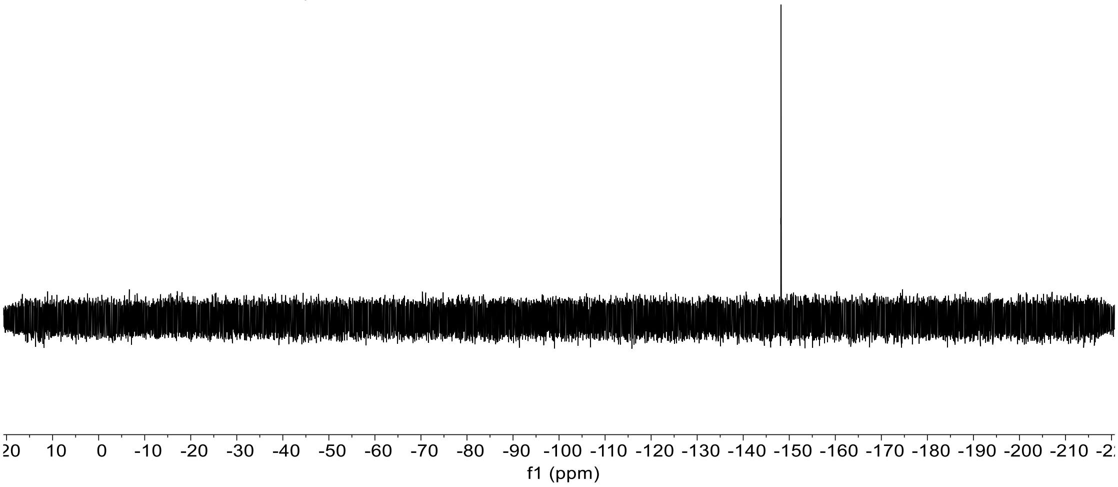

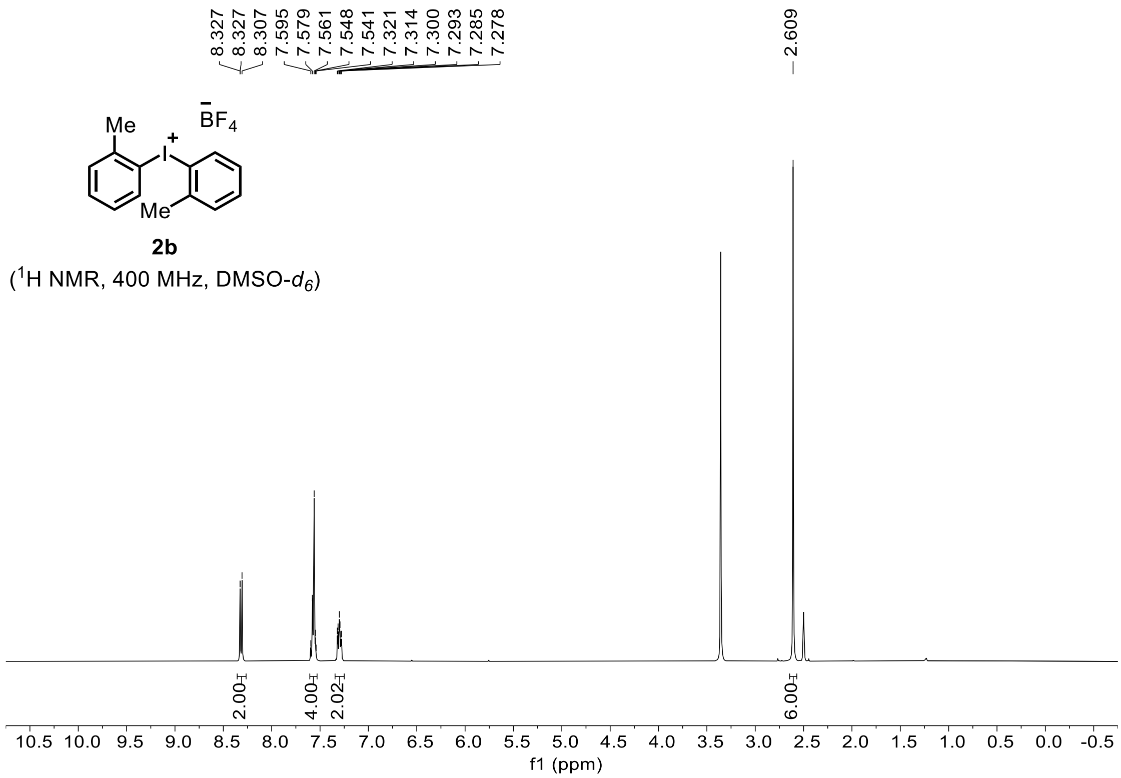

8.327
8.327
8.307
7.595
7.579
7.561
7.548
7.541
7.321
7.314
7.300
7.293
7.285
7.278
2.609
Me
BF4
Me
2b
(1H NMR, 400 MHz, DMSO-d6)
2.00
4.00
2.02
6.00
10.5 10.0 9.5 9.0 8.5 8.0 7.5 7.0 6.5 6.0 5.5 5.0 4.5 4.0 3.5 3.0 2.5 2.0 1.5 1.0 0.5 0.0 -0.5
f1 (ppm)

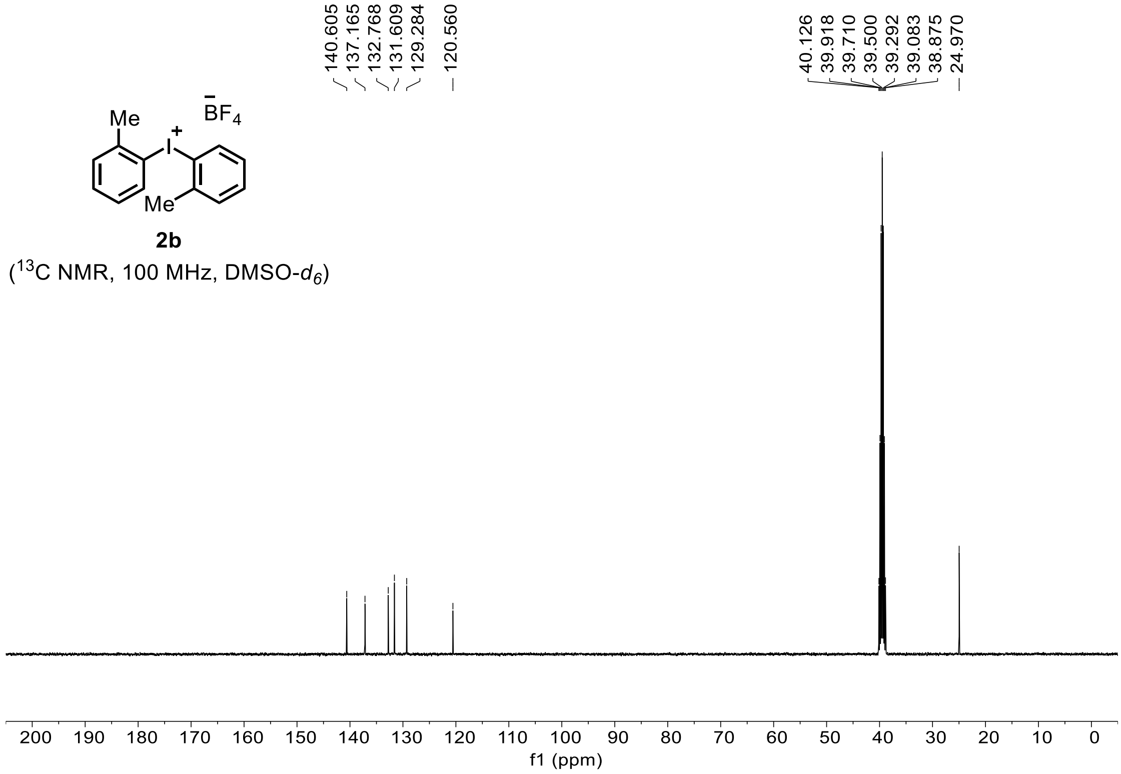

140.605
137.165
132.768
131.609
129.284
120.560
40.126
39.918
39.710
39.500
39.292
39.083
38.875
24.970
Me
BF4
Me
2b
(13C NMR, 100 MHz, DMSO-d6)
200 190 180 170 160 150 140 130 120 110 100 90 80 70 60 50 40 30 20 10 0
f1 (ppm)

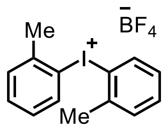

2b

(^{19}F NMR, 376 MHz, DMSO-d_6)

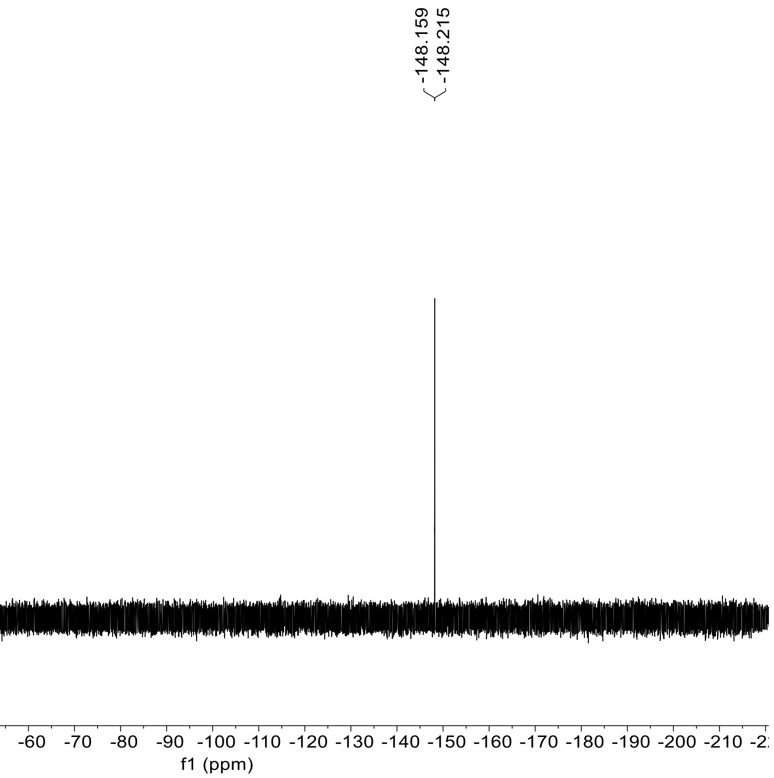

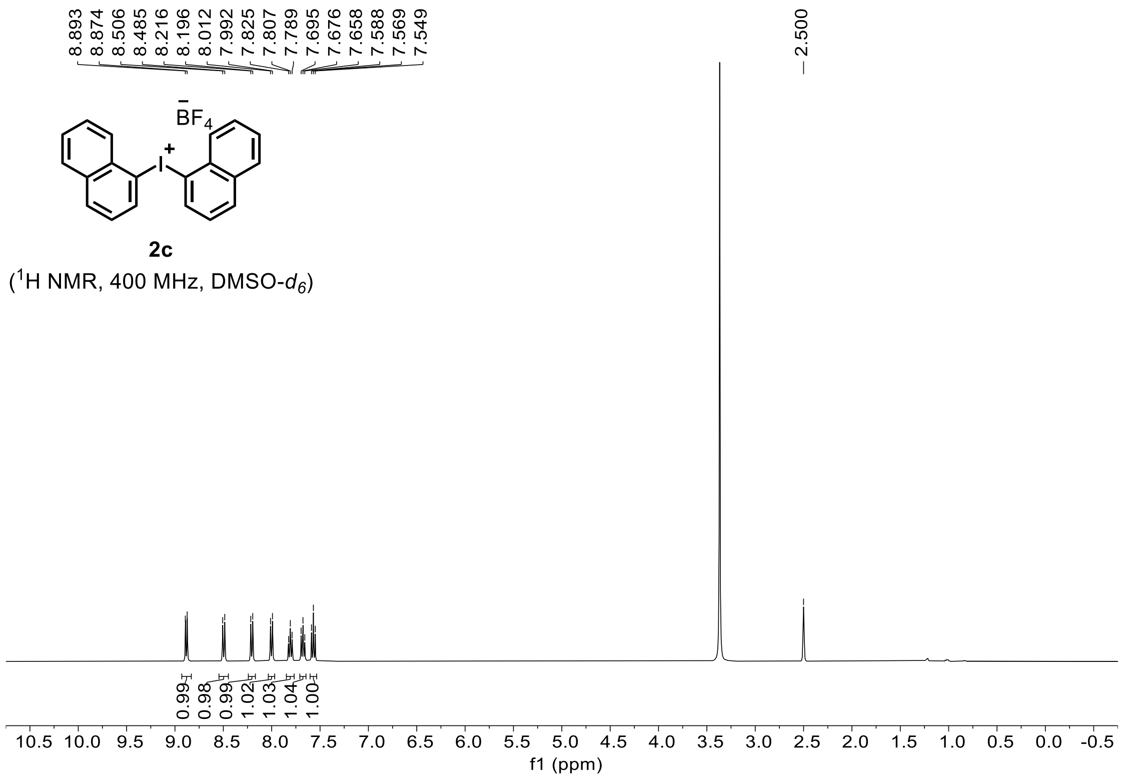

2c

(^{1}H NMR, 400 MHz, DMSO-d_6)

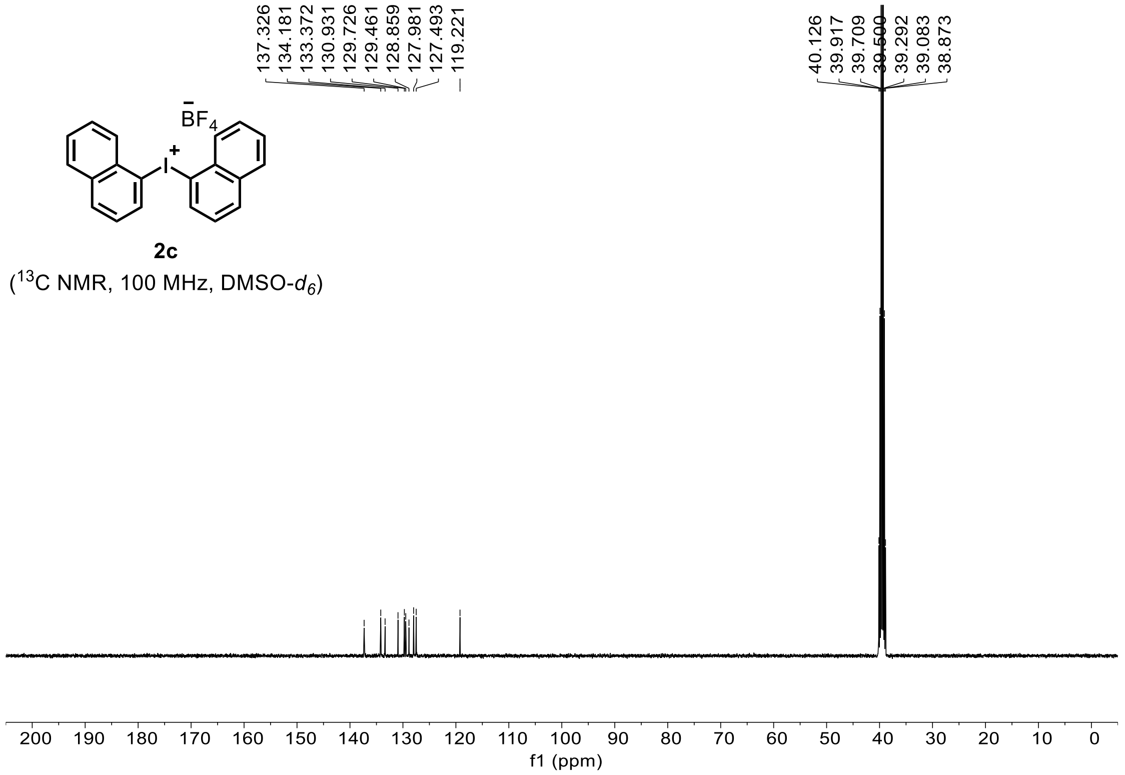

2c

(^{13}C NMR, 100 MHz, DMSO-d_6)

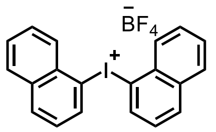

2c

(^{19}F NMR, 376 MHz, DMSO-d_6)

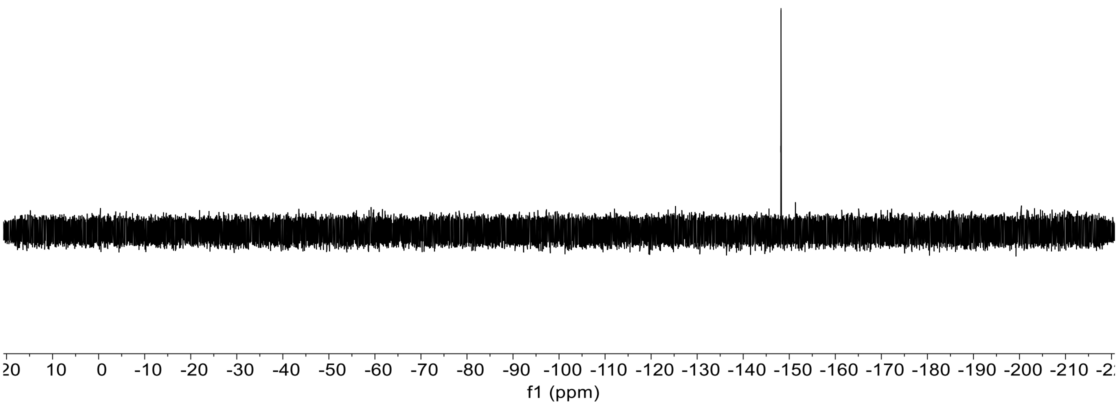

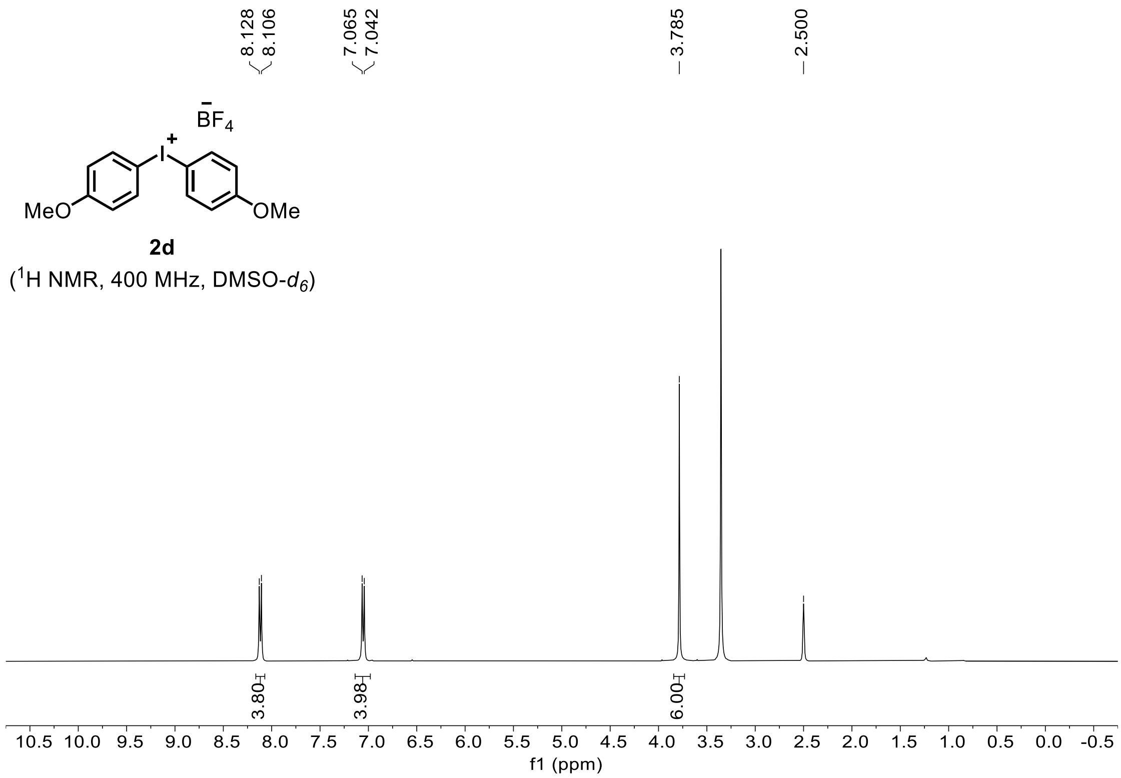
8.128
8.106
7.065
7.042
3.785
2.500
$\bar{B}F_4$
I+
MeO
OMe
2d
(^{1}H NMR, 400 MHz, DMSO-d_6)
3.80
3.98
6.00
10.5 10.0 9.5 9.0 8.5 8.0 7.5 7.0 6.5 6.0 5.5 5.0 4.5 4.0 3.5 3.0 2.5 2.0 1.5 1.0 0.5 0.0 -0.5
f1 (ppm)

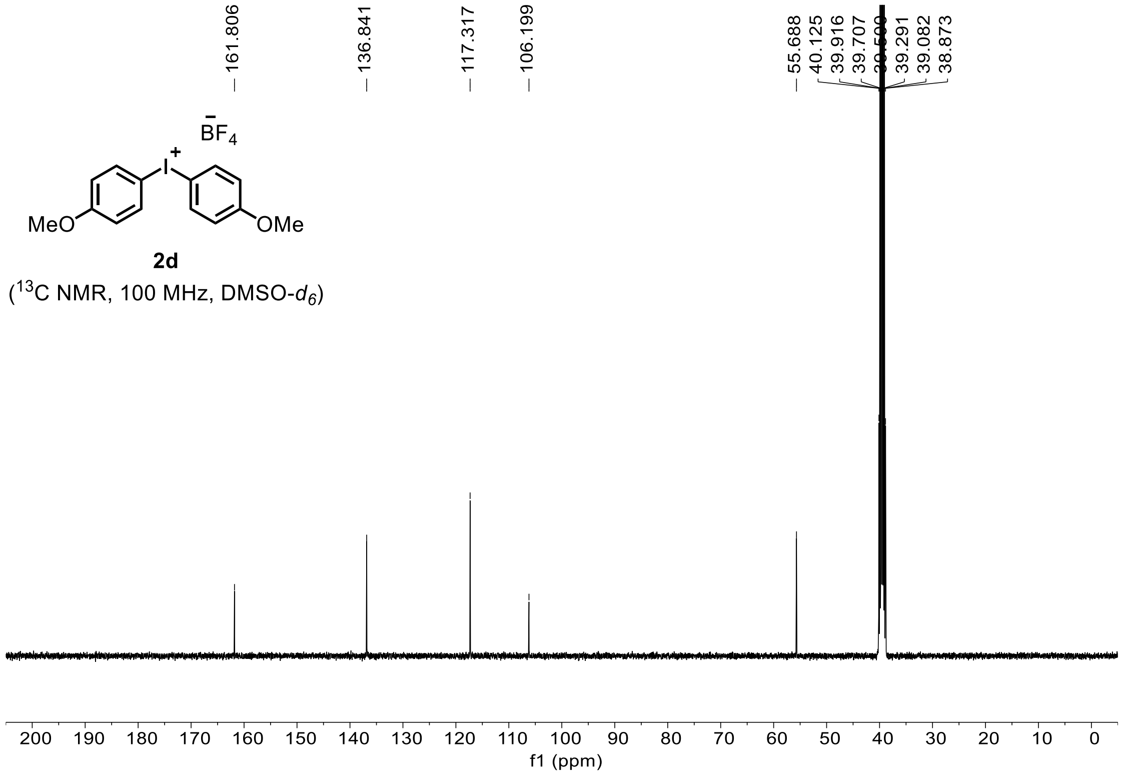
161.806
136.841
117.317
106.199
55.688
40.125
39.916
39.707
39.291
39.082
38.873
$\bar{B}F_4$
I+
MeO
OMe
2d
(^{13}C NMR, 100 MHz, DMSO-d_6)
200 190 180 170 160 150 140 130 120 110 100 90 80 70 60 50 40 30 20 10 0
f1 (ppm)

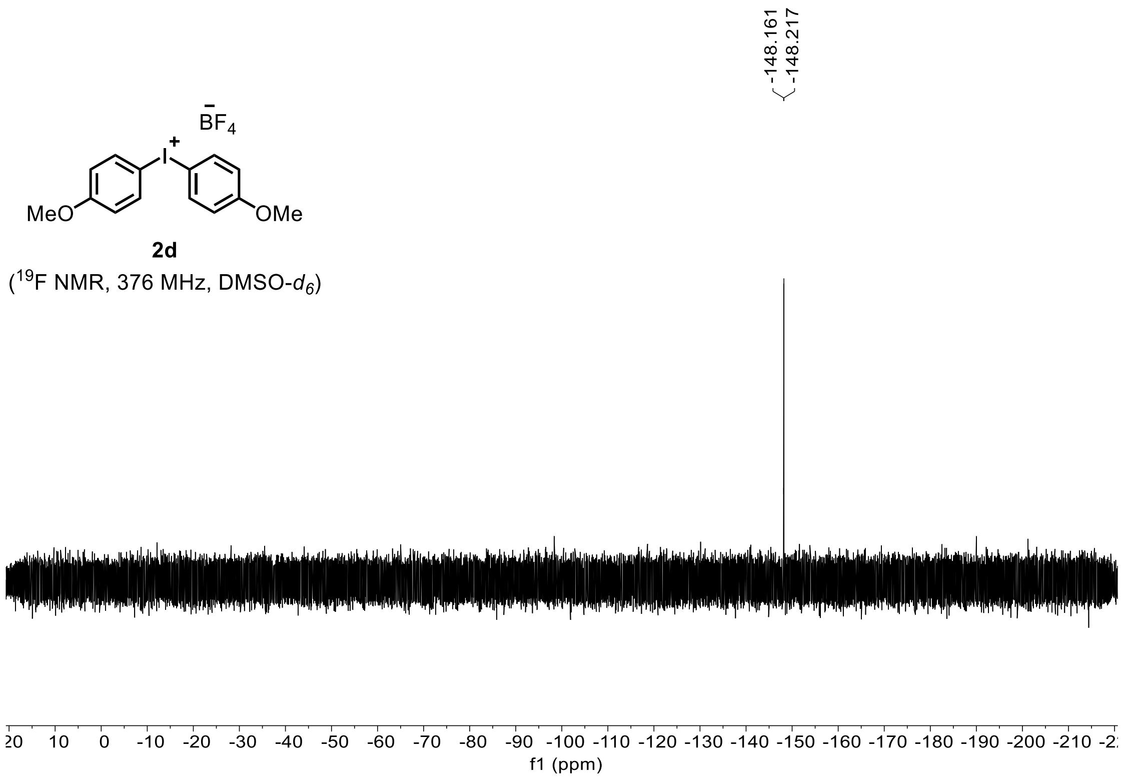

2d

(^{19}F NMR, 376 MHz, DMSO-d_6)

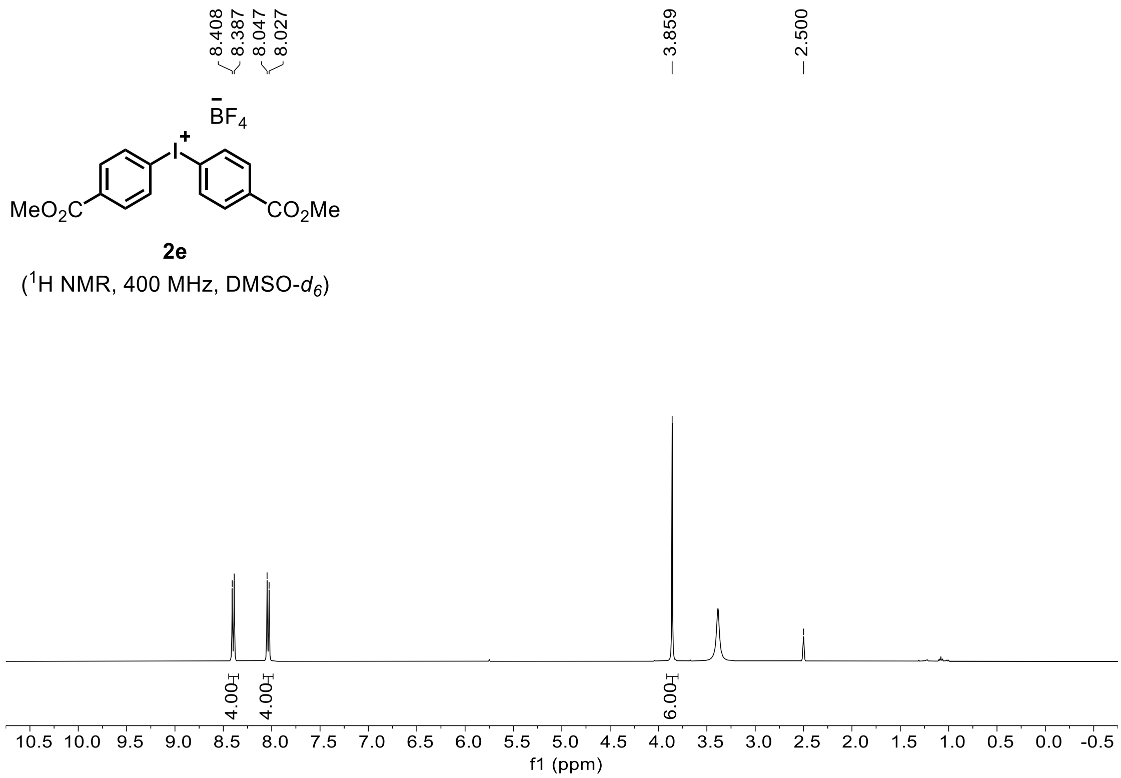
8.408
8.387
8.047
8.027
3.859
2.500
BF4
I+
MeO2C
CO2Me
2e
(1H NMR, 400 MHz, DMSO-d6)
4.00
4.00
6.00
10.5 10.0 9.5 9.0 8.5 8.0 7.5 7.0 6.5 6.0 5.5 5.0 4.5 4.0 3.5 3.0 2.5 2.0 1.5 1.0 0.5 0.0 -0.5
f1 (ppm)

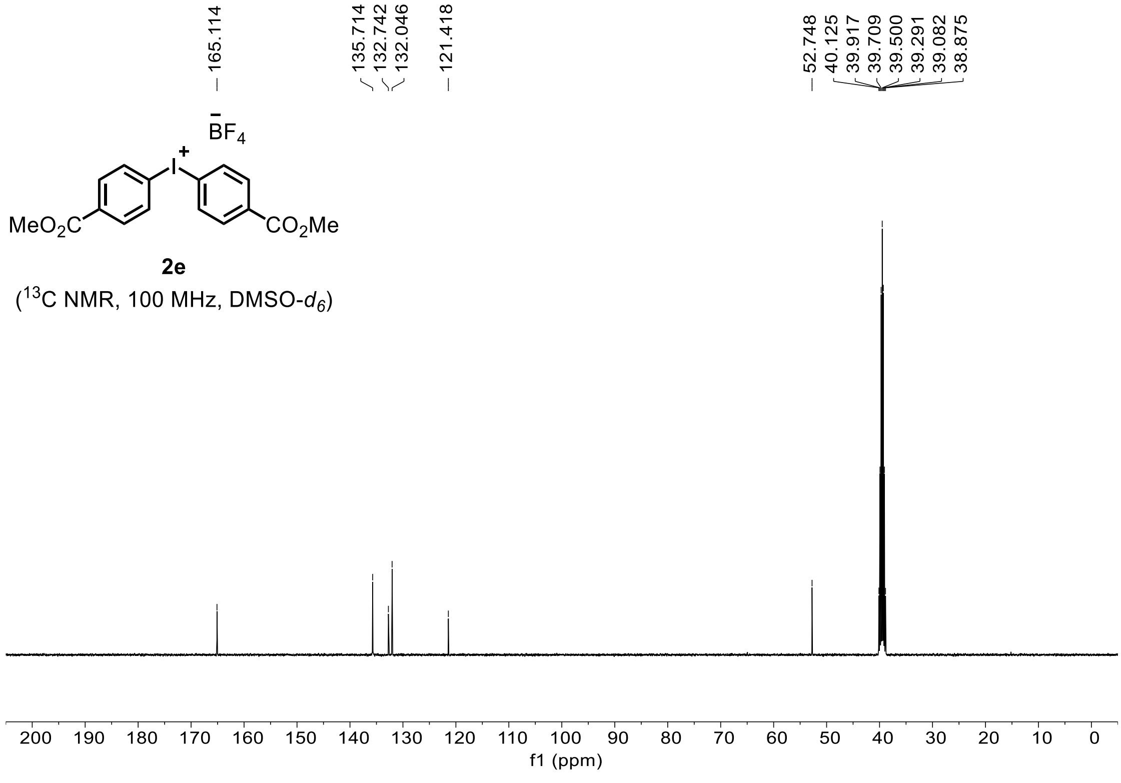
165.114
135.714
132.742
132.046
121.418
52.748
40.125
39.917
39.709
39.500
39.291
39.082
38.875
BF4
I+
MeO2C
CO2Me
2e
(13C NMR, 100 MHz, DMSO-d6)
200 190 180 170 160 150 140 130 120 110 100 90 80 70 60 50 40 30 20 10 0
f1 (ppm)

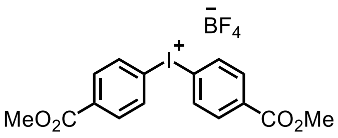

2e

(^{19}F NMR, 376 MHz, DMSO-d_6)

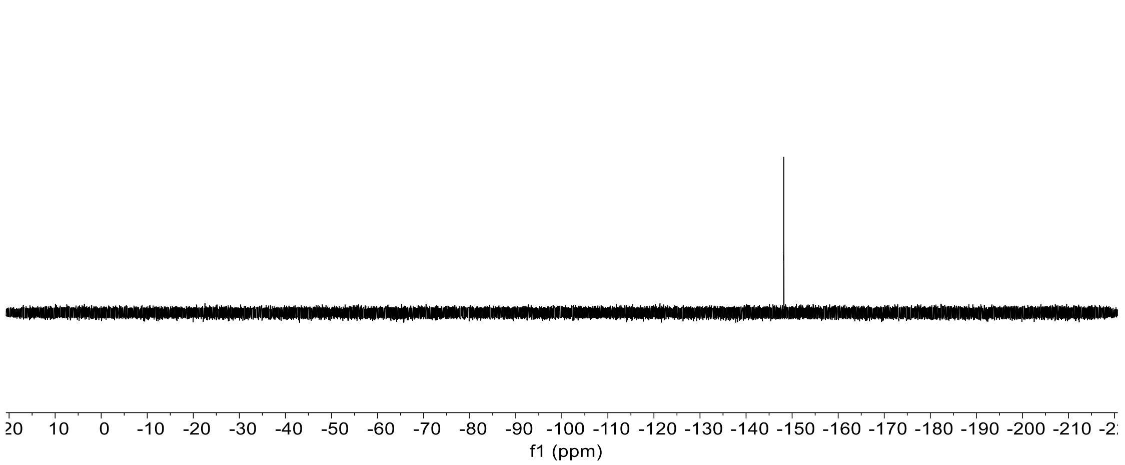

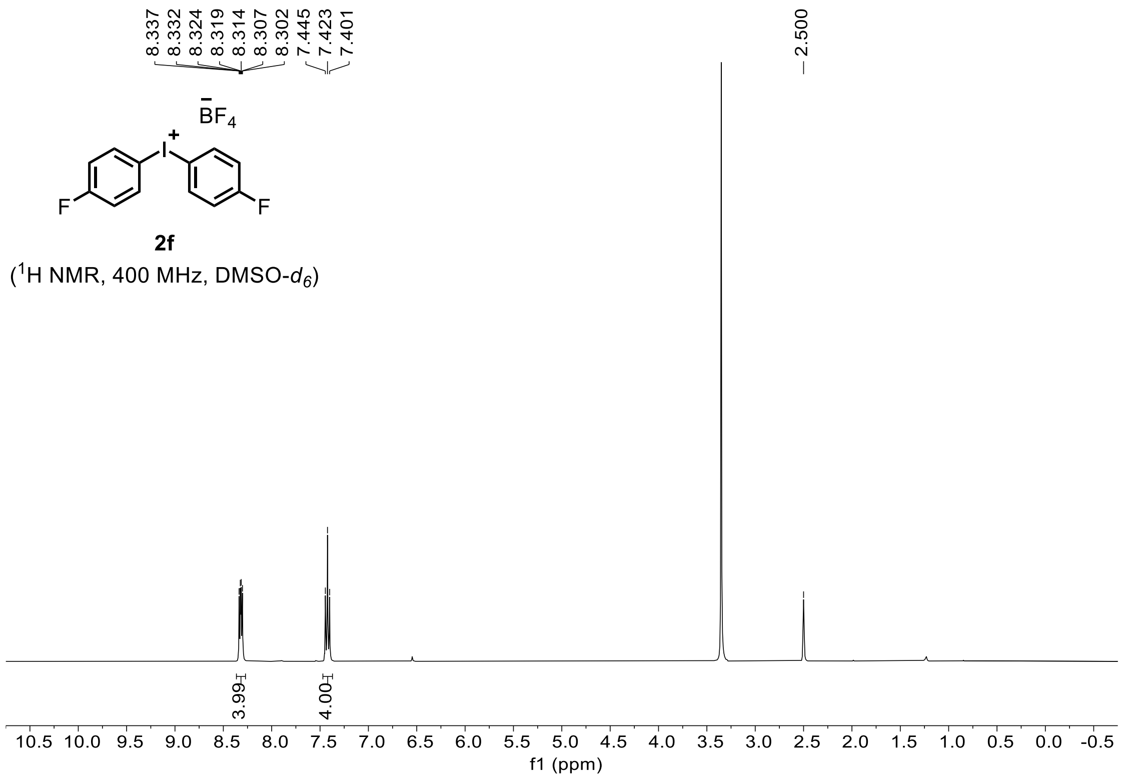
8.337
8.332
8.324
8.319
8.314
8.307
8.302
7.445
7.423
7.401
2.500
BF4
I
F
F
2f
(1H NMR, 400 MHz, DMSO-d6)
3.99
4.00
10.5 10.0 9.5 9.0 8.5 8.0 7.5 7.0 6.5 6.0 5.5 5.0 4.5 4.0 3.5 3.0 2.5 2.0 1.5 1.0 0.5 0.0 -0.5
f1 (ppm)

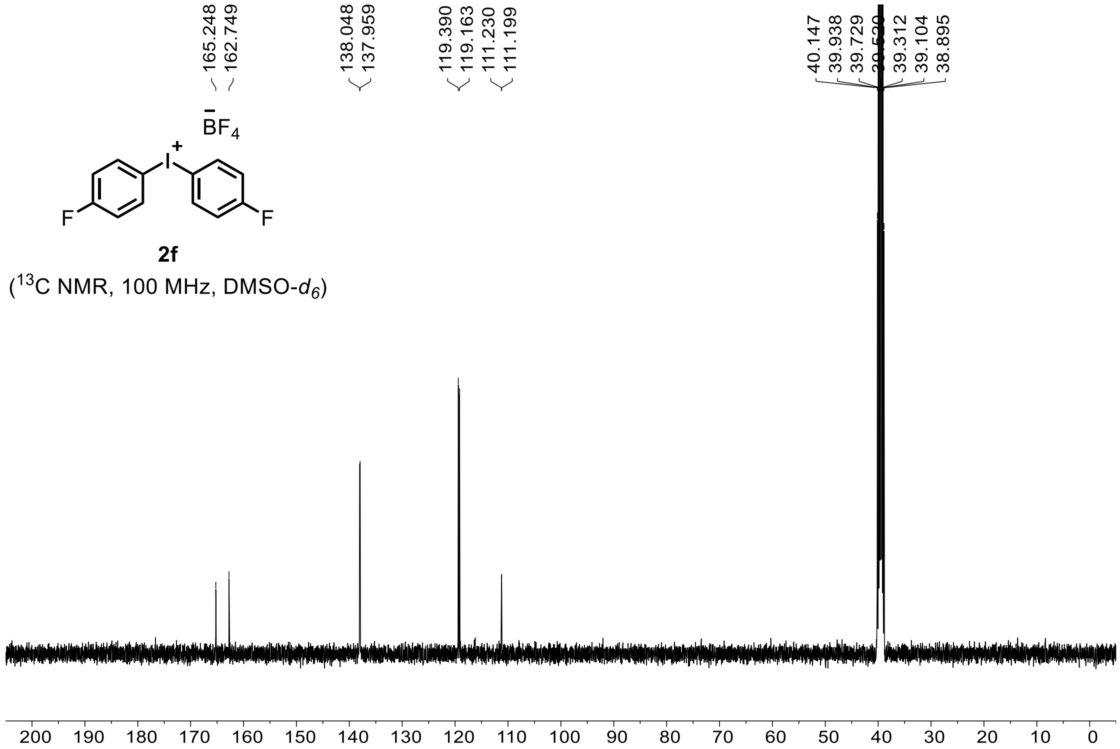
165.248
162.749
138.048
137.959
119.390
119.163
111.230
111.199
40.147
39.938
39.729
39.312
39.104
38.895
BF4
I
F
F
2f
(13C NMR, 100 MHz, DMSO-d6)
200 190 180 170 160 150 140 130 120 110 100 90 80 70 60 50 40 30 20 10 0
f1 (ppm)

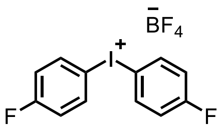

2f

(^{19}F NMR, 376 MHz, DMSO-d_6)

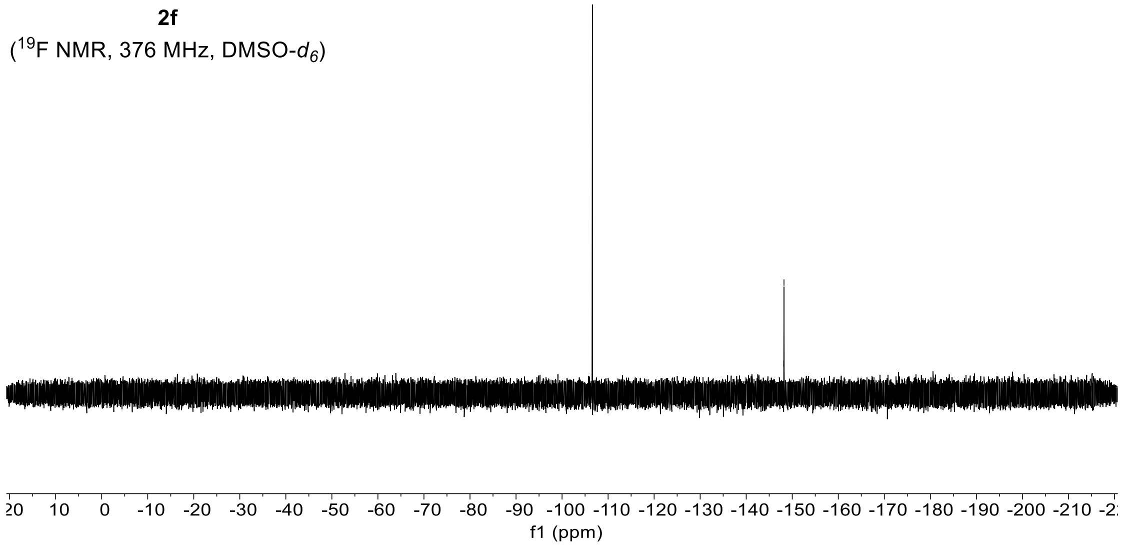

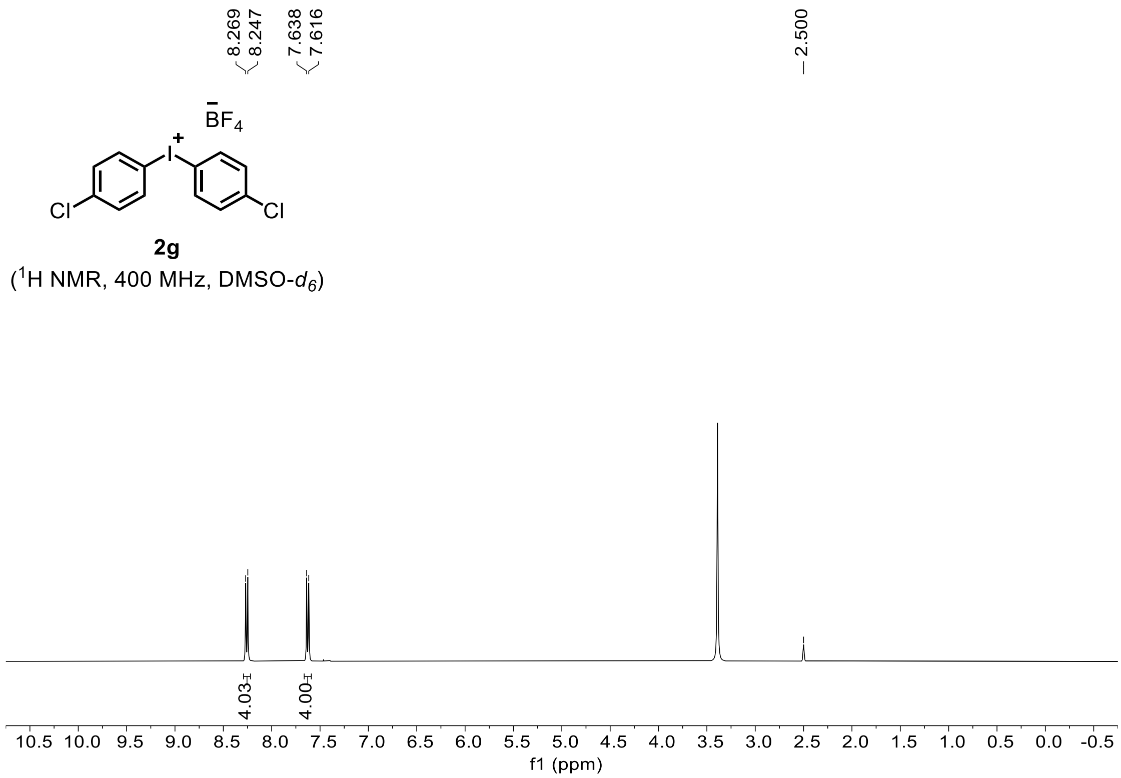

2g

(^{1}H NMR, 400 MHz, DMSO-d_6)

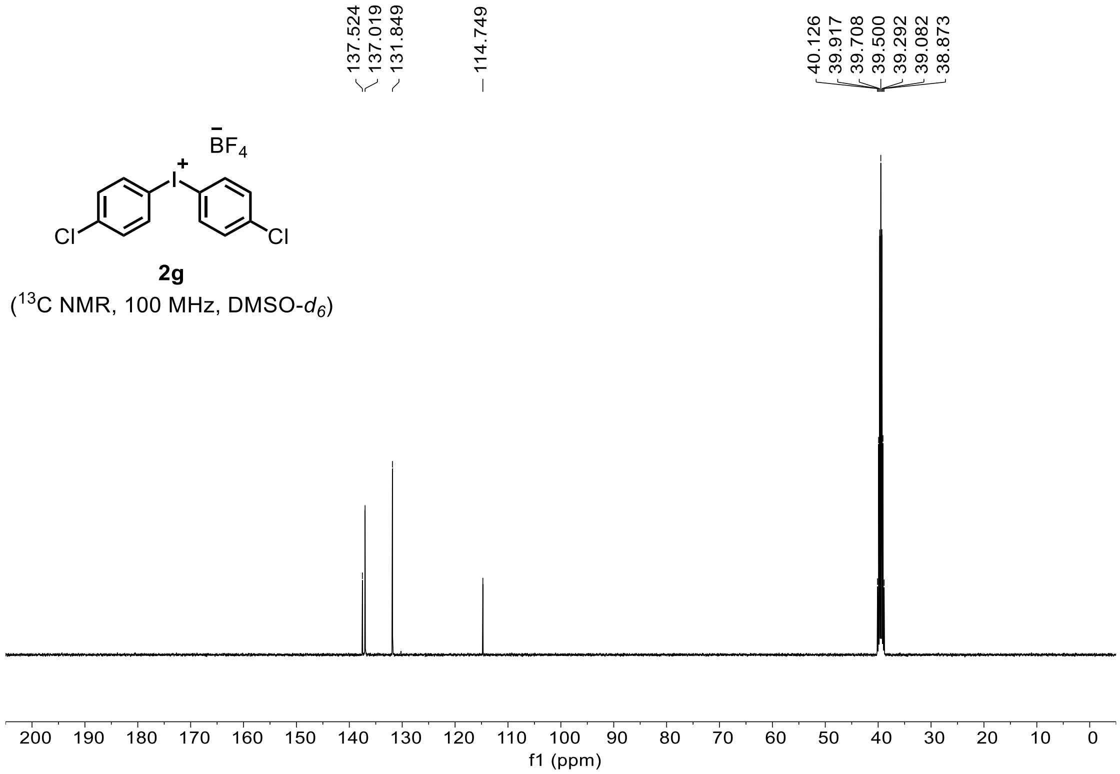

2g

(^{13}C NMR, 100 MHz, DMSO-d_6)

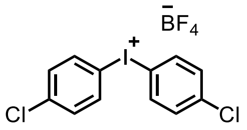

2g

(^{19}F NMR, 376 MHz, DMSO-d_6)

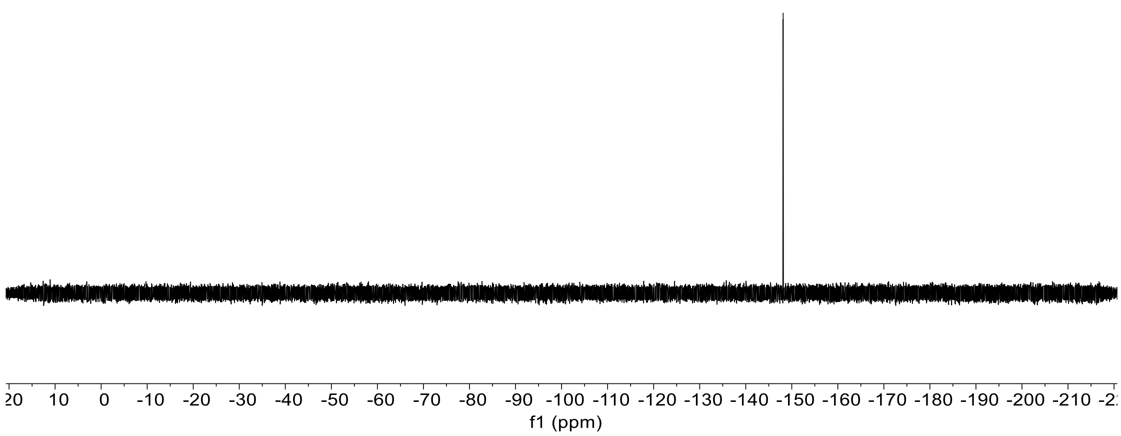

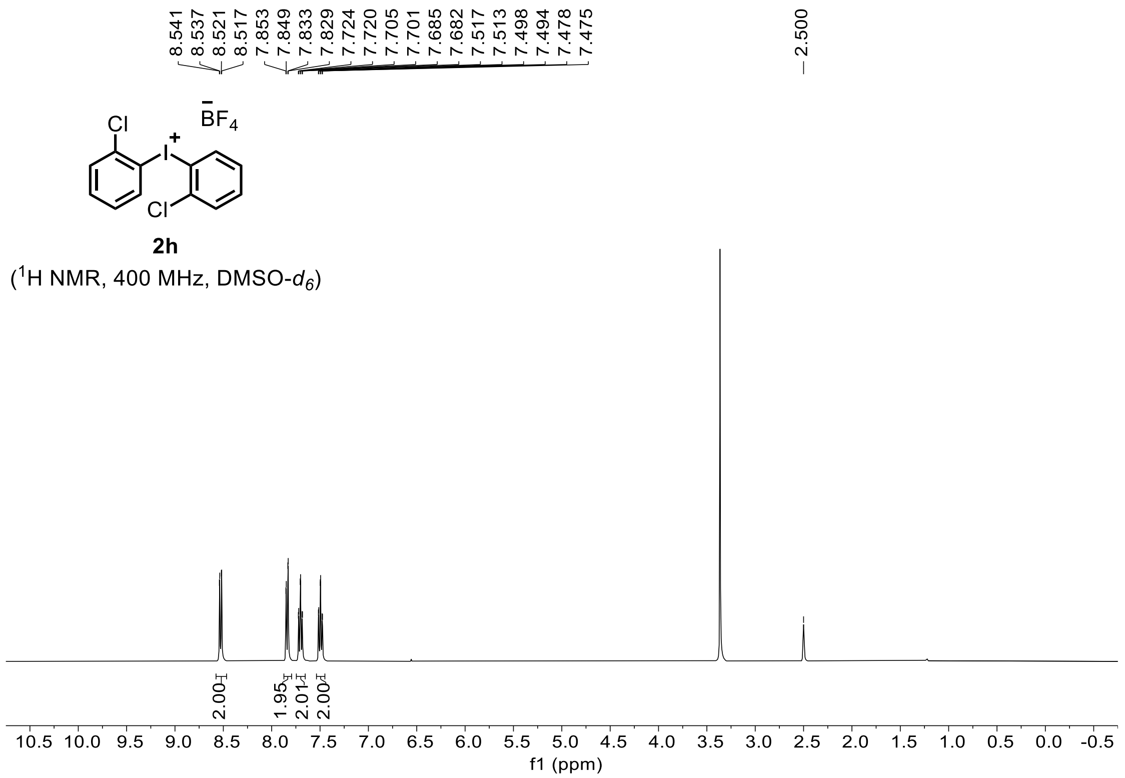
8.541
8.537
8.521
8.517
7.853
7.849
7.833
7.829
7.724
7.720
7.705
7.701
7.685
7.682
7.517
7.513
7.498
7.494
7.478
7.475
2.500
Cl
BF4
Cl
2h
(1H NMR, 400 MHz, DMSO-d6)
2.00
1.95
2.01
2.00
f1 (ppm)

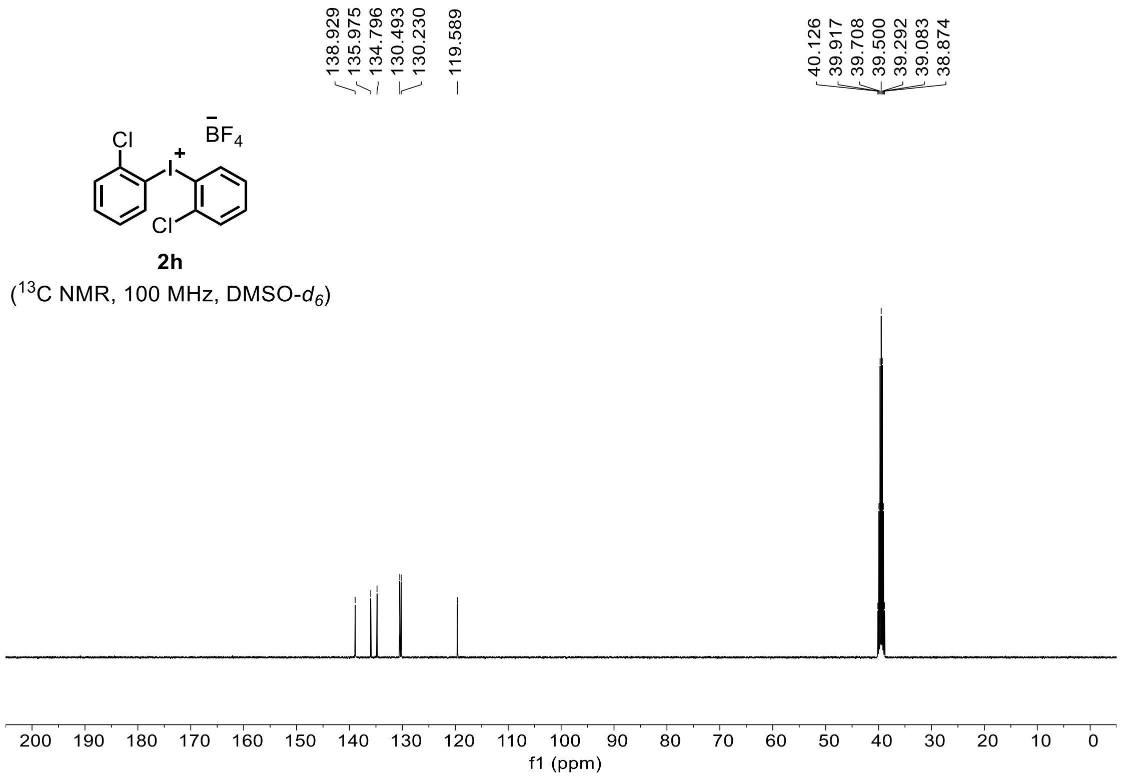
138.929
135.975
134.796
130.493
130.230
119.589
40.126
39.917
39.708
39.500
39.292
39.083
38.874
Cl
BF4
Cl
2h
(13C NMR, 100 MHz, DMSO-d6)
f1 (ppm)

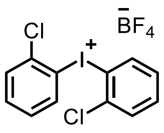

2h

(^{19}F NMR, 376 MHz, DMSO-d_6)

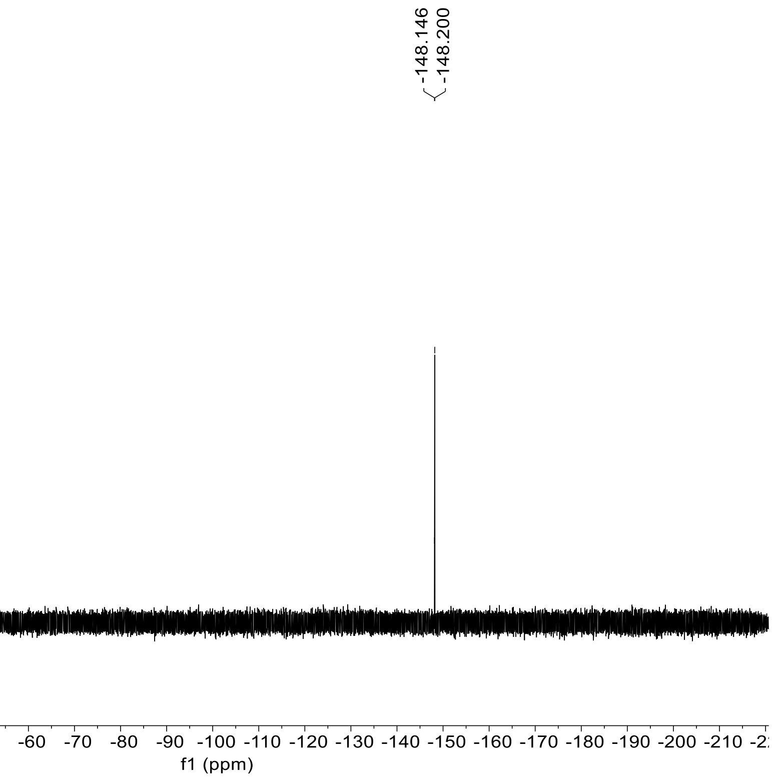

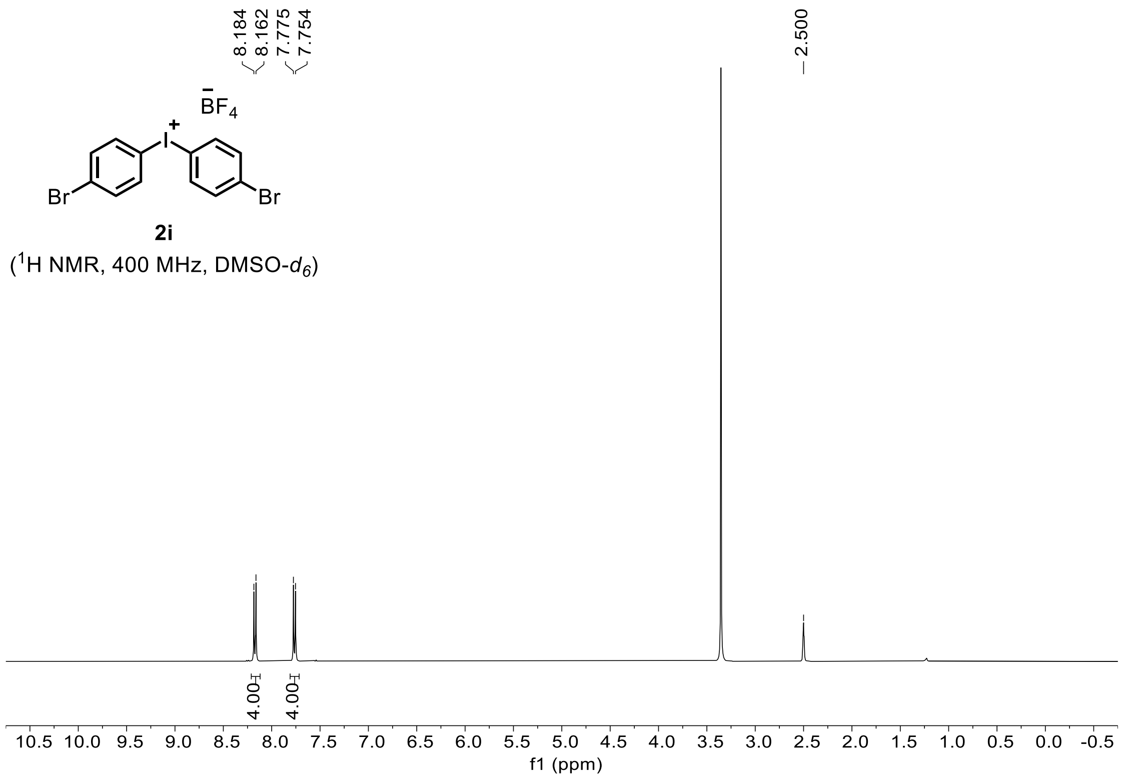
8.184
8.162
7.775
7.754
2.500
BF4
I
Br
Br
2i
(1H NMR, 400 MHz, DMSO-d6)
4.00
4.00
10.5 10.0 9.5 9.0 8.5 8.0 7.5 7.0 6.5 6.0 5.5 5.0 4.5 4.0 3.5 3.0 2.5 2.0 1.5 1.0 0.5 0.0 -0.5
f1 (ppm)

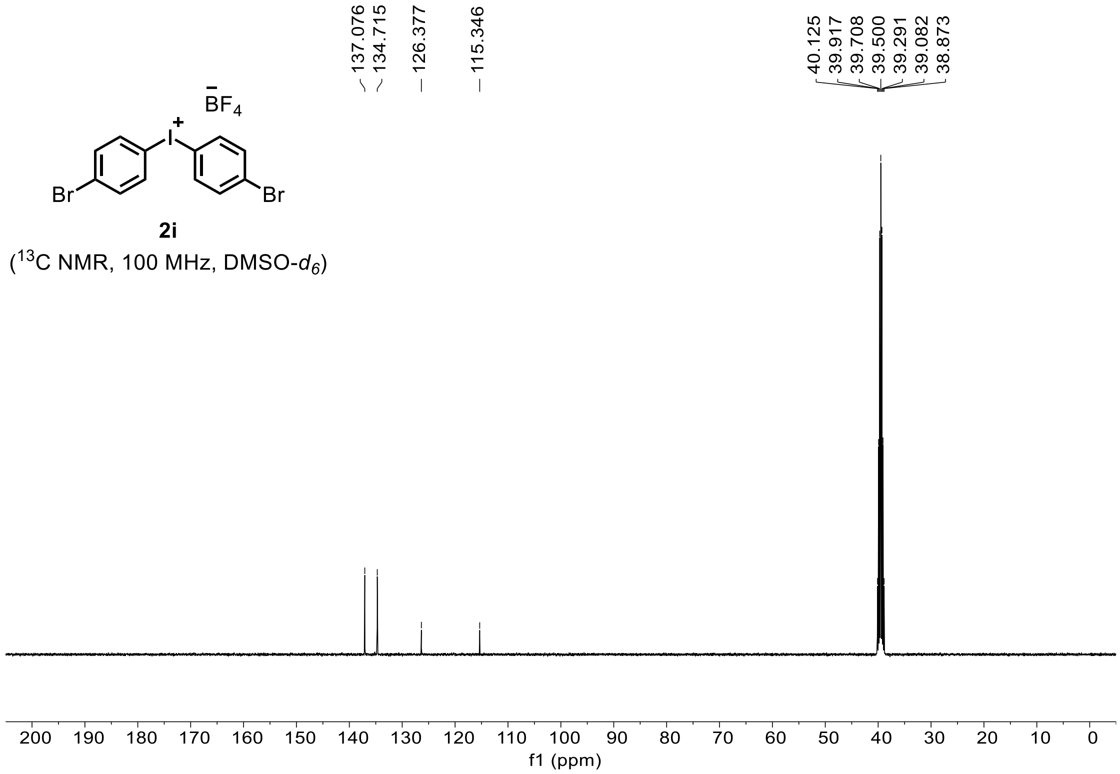
137.076
134.715
126.377
115.346
40.125
39.917
39.708
39.500
39.291
39.082
38.873
BF4
I
Br
Br
2i
(13C NMR, 100 MHz, DMSO-d6)
200 190 180 170 160 150 140 130 120 110 100 90 80 70 60 50 40 30 20 10 0
f1 (ppm)

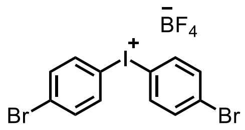

2i

(^{19}F NMR, 376 MHz, DMSO-d_6)

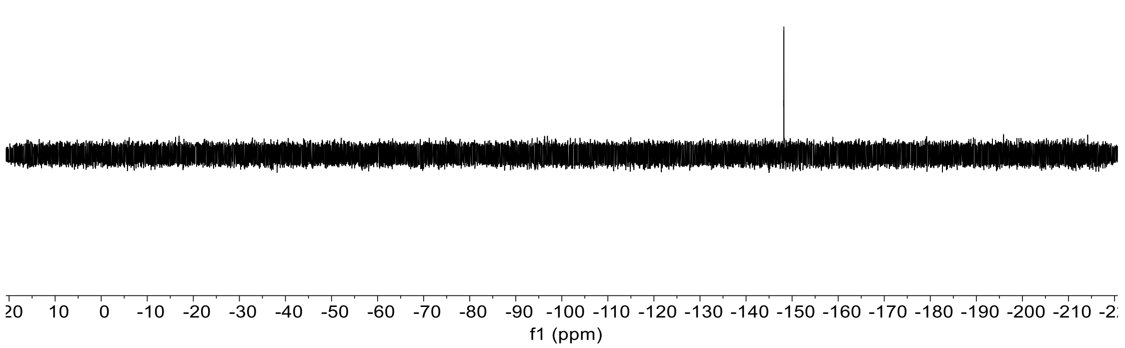

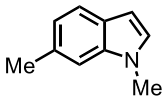

1c

(^{1}H NMR, 400 MHz, $CDCl_3$)

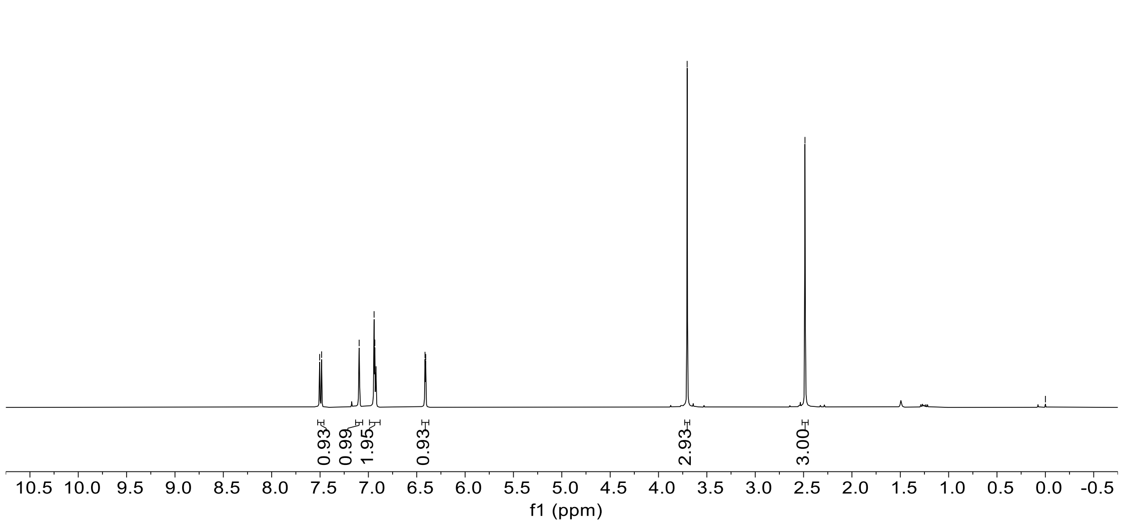

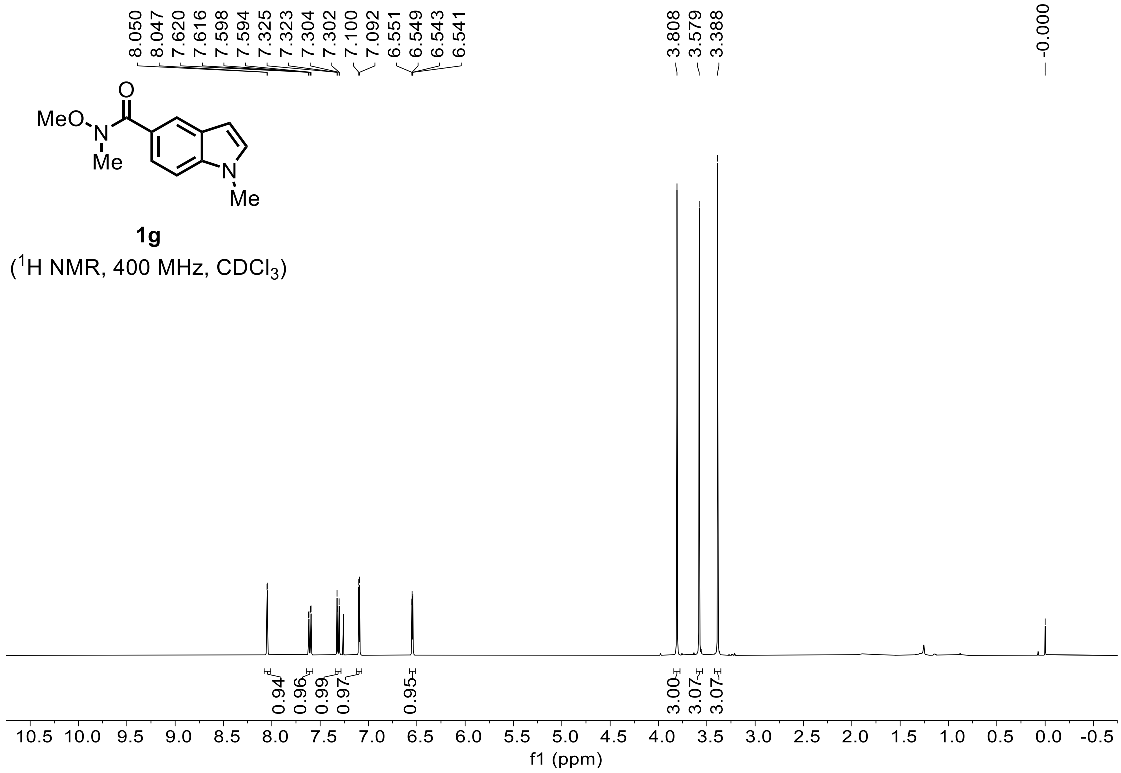

1g

(^{1}H NMR, 400 MHz, $CDCl_3$)

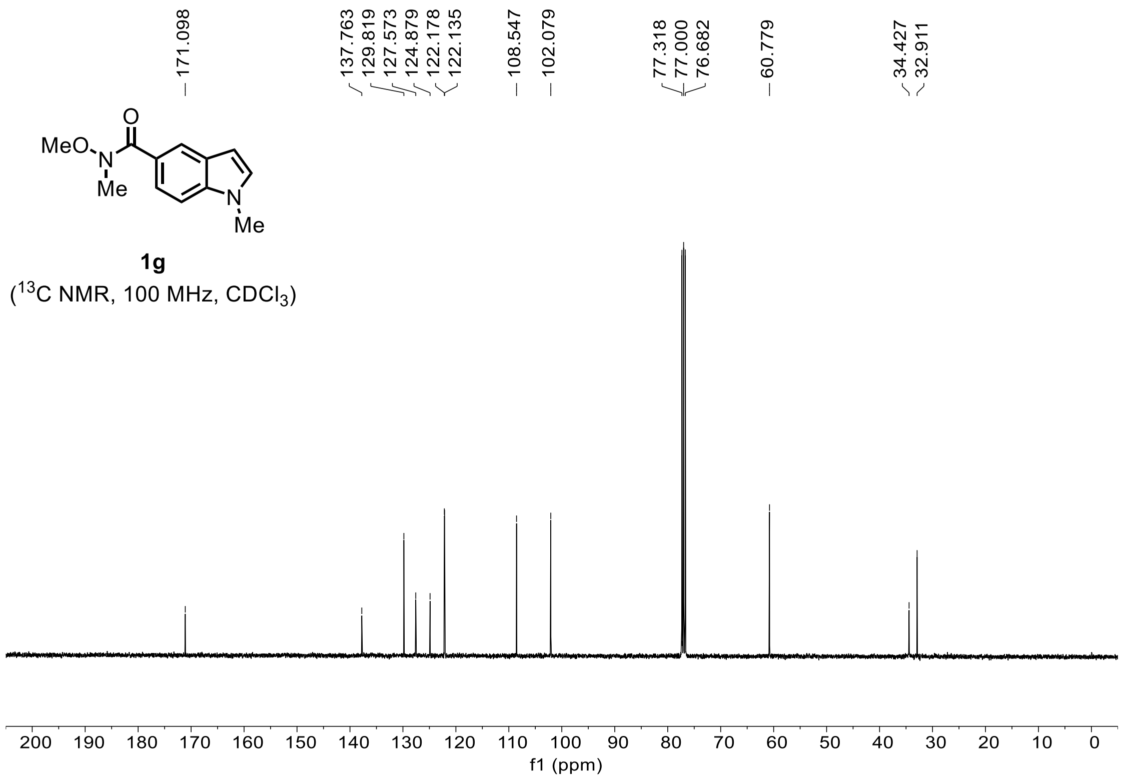

1g

(^{13}C NMR, 100 MHz, $CDCl_3$)

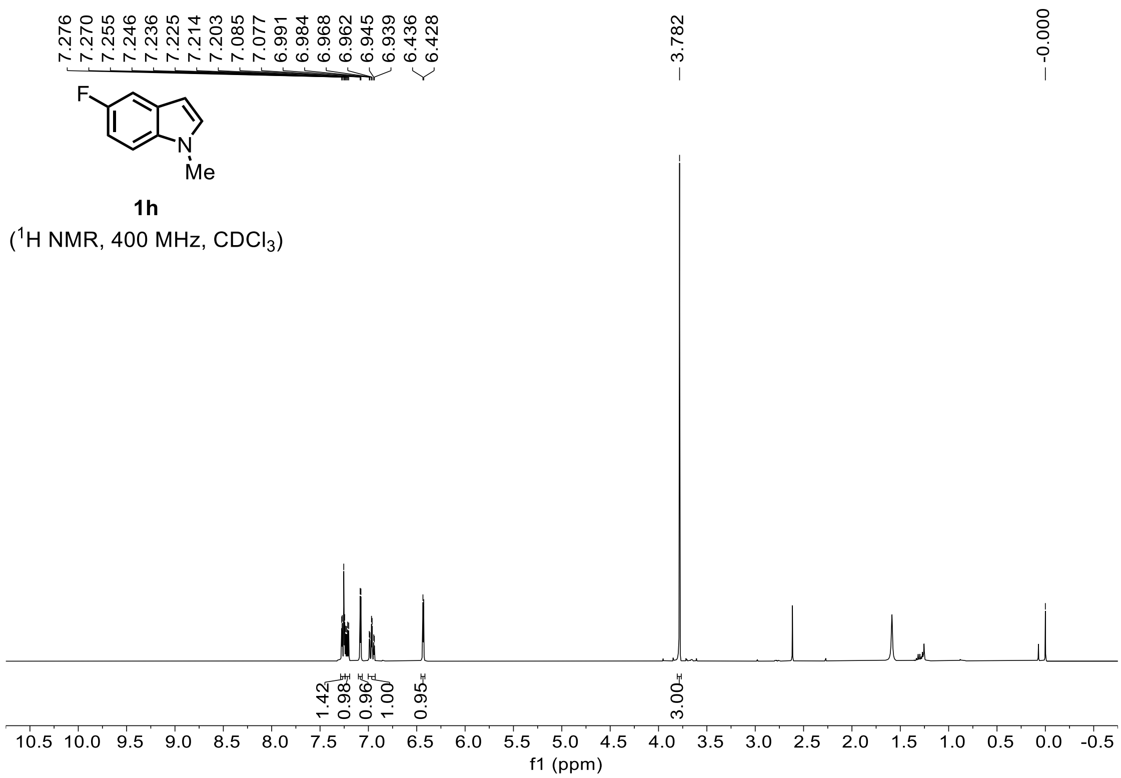
7.276
7.270
7.255
7.246
7.236
7.225
7.214
7.203
7.085
7.077
6.991
6.984
6.968
6.962
6.945
6.939
6.436
6.428
3.782
-0.000
F
N
Me
1h
(¹H NMR, 400 MHz, CDCl₃)
1.42
0.98
0.96
1.00
0.95
3.00
10.5 10.0 9.5 9.0 8.5 8.0 7.5 7.0 6.5 6.0 5.5 5.0 4.5 4.0 3.5 3.0 2.5 2.0 1.5 1.0 0.5 0.0 -0.5
f1 (ppm)

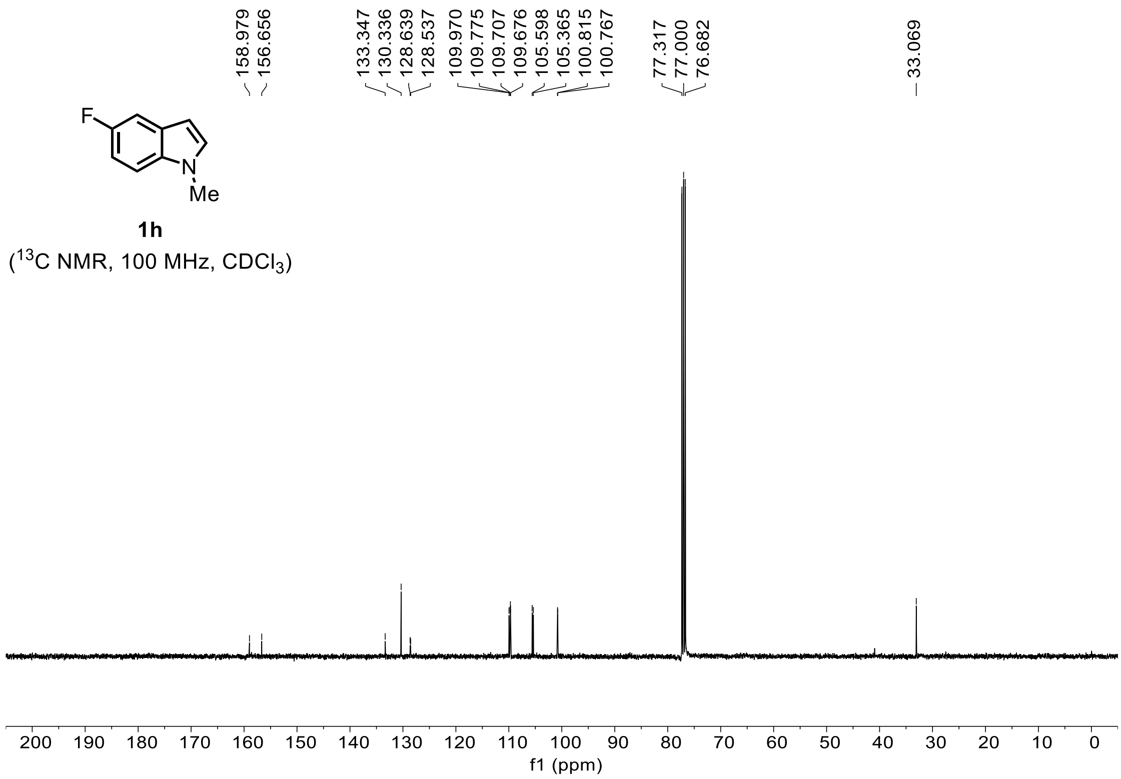
158.979
156.656
133.347
130.336
128.639
128.537
109.970
109.775
109.707
109.676
105.598
105.365
100.815
100.767
77.317
77.000
76.682
33.069
F
N
Me
1h
(¹³C NMR, 100 MHz, CDCl₃)
200 190 180 170 160 150 140 130 120 110 100 90 80 70 60 50 40 30 20 10 0
f1 (ppm)

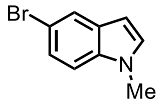

1j

(^{1}H NMR, 400 MHz, $CDCl_3$)

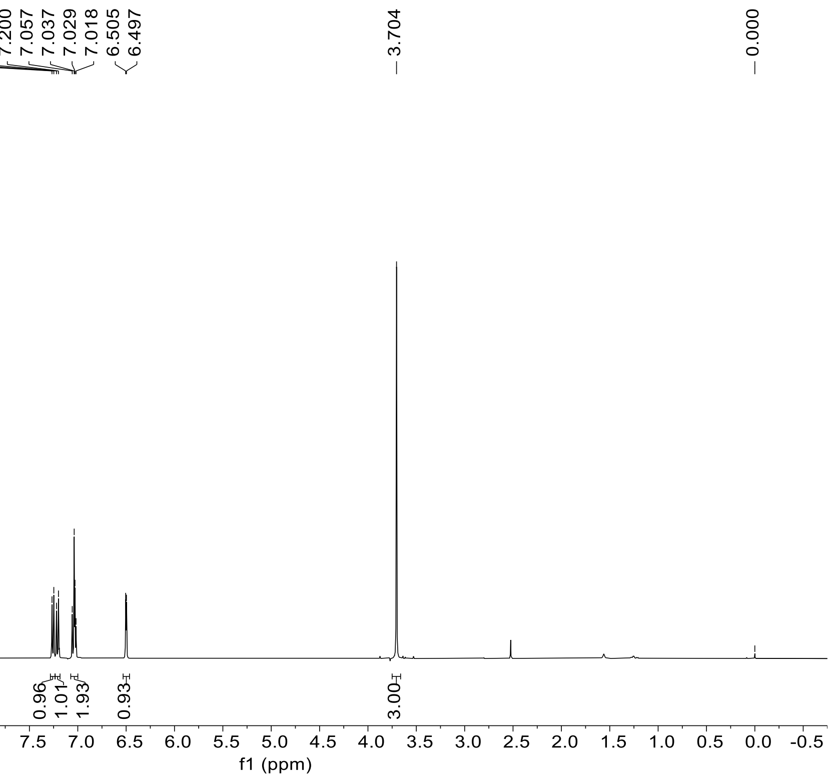

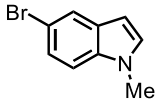

1j

(^{13}C NMR, 100 MHz, $CDCl_3$)

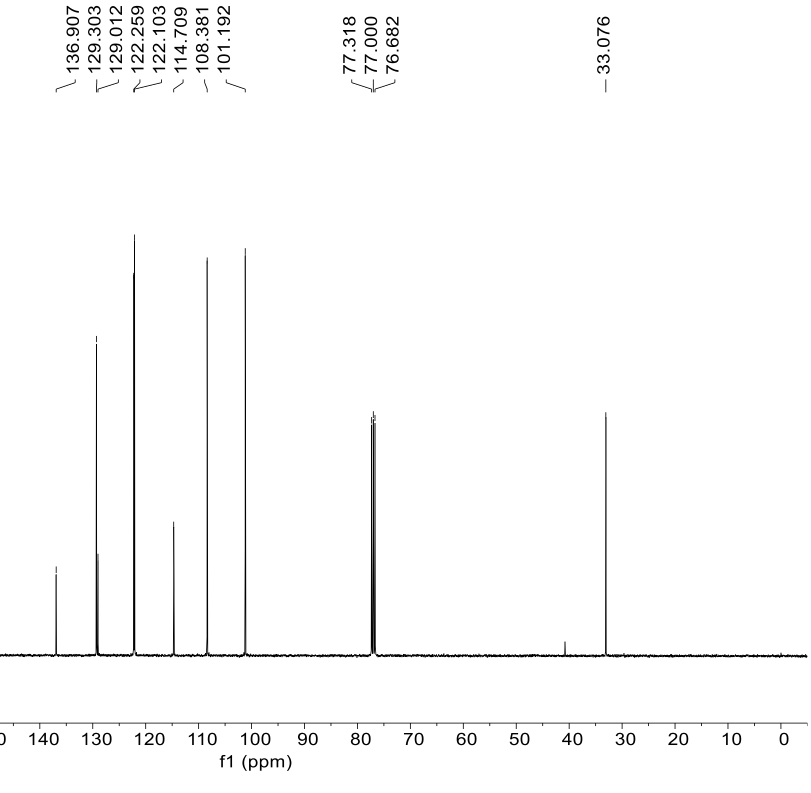

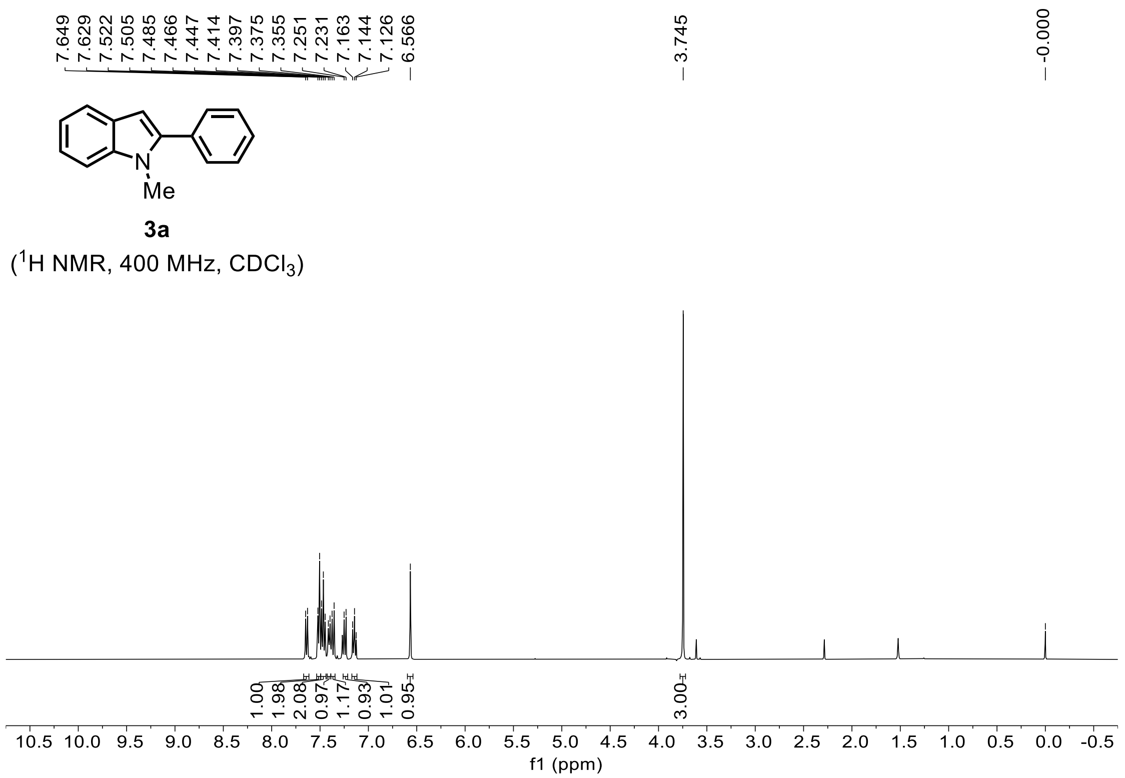

3a

(^{1}H NMR, 400 MHz, $CDCl_3$)

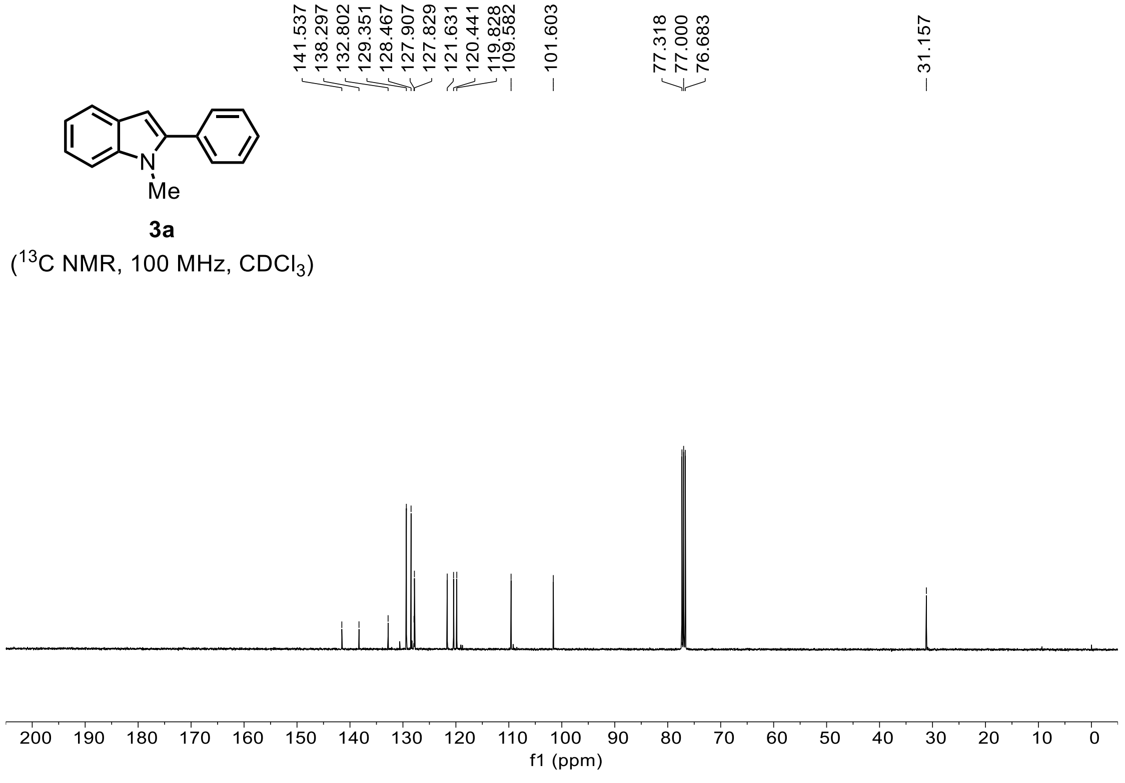

3a

(^{13}C NMR, 100 MHz, $CDCl_3$)

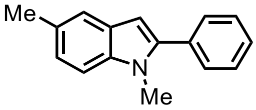

3b

(^{1}H NMR, 400 MHz, $CDCl_3$)

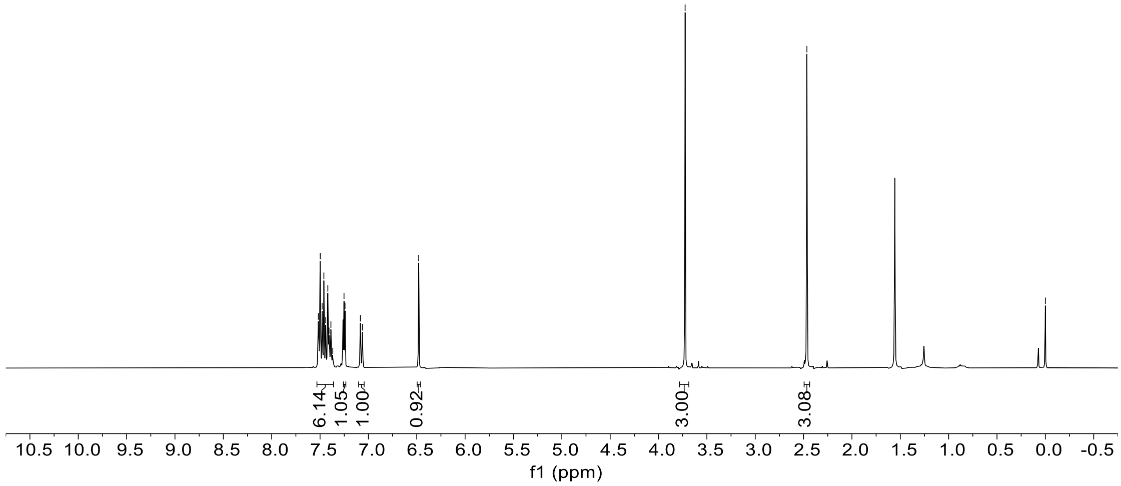

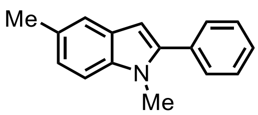

3b

(^{13}C NMR, 100 MHz, $CDCl_3$)

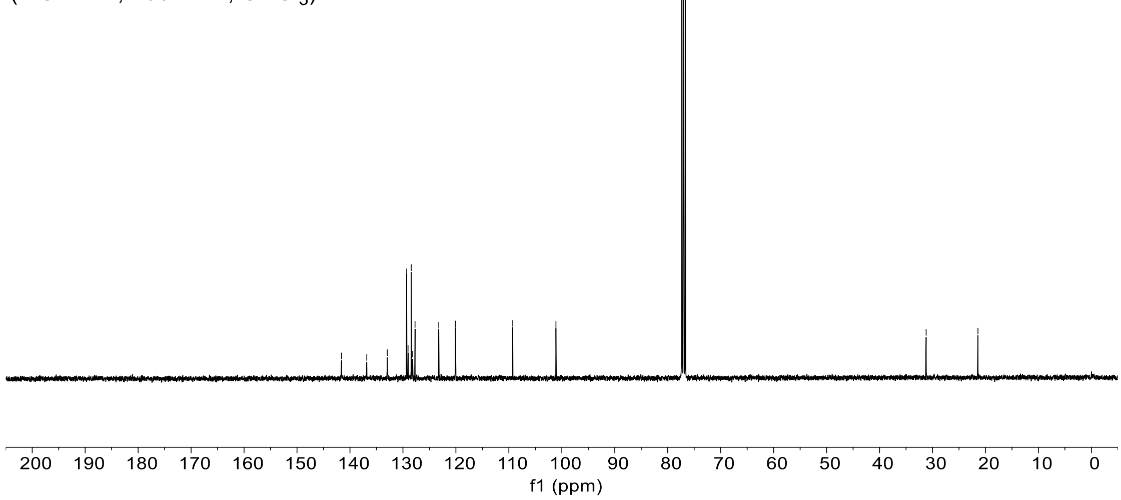

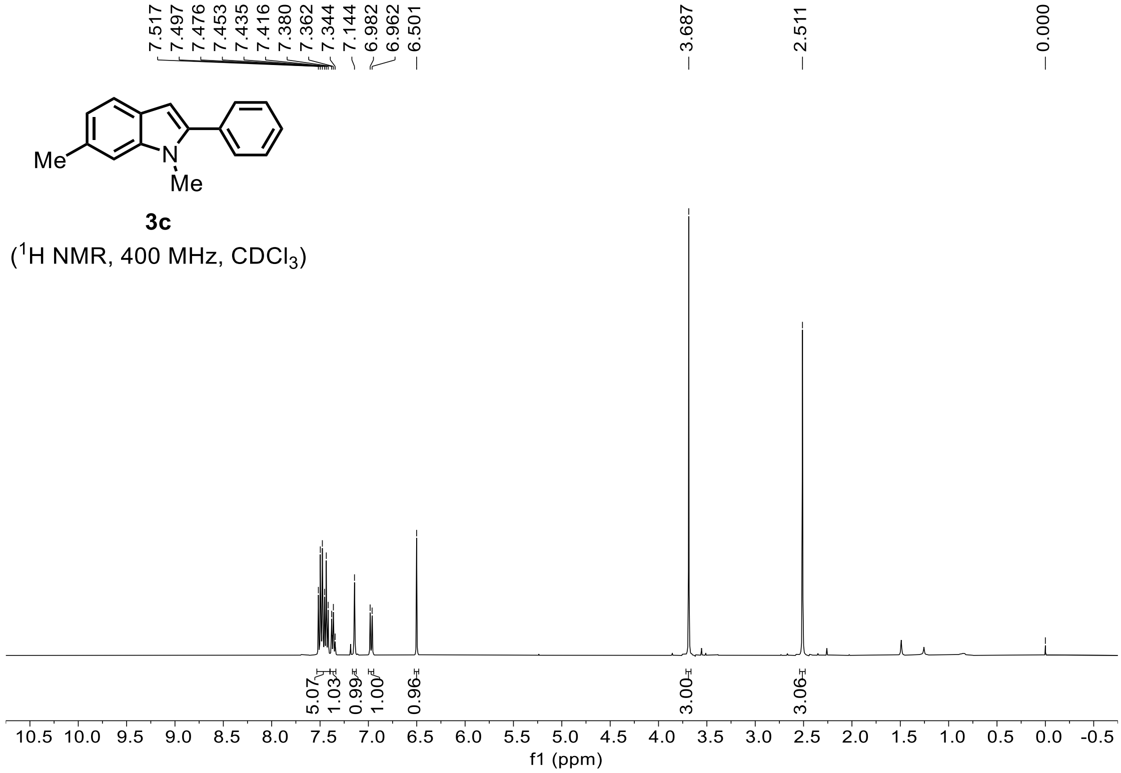
7.517
7.497
7.476
7.453
7.435
7.416
7.380
7.362
7.344
7.144
6.982
6.962
6.501
3.687
2.511
0.000
Me
N
Me
3c
(^{1}H NMR, 400 MHz, $CDCl_3$)
5.07
1.03
0.99
1.00
0.96
3.00
3.06
10.5 10.0 9.5 9.0 8.5 8.0 7.5 7.0 6.5 6.0 5.5 5.0 4.5 4.0 3.5 3.0 2.5 2.0 1.5 1.0 0.5 0.0 -0.5
f1 (ppm)

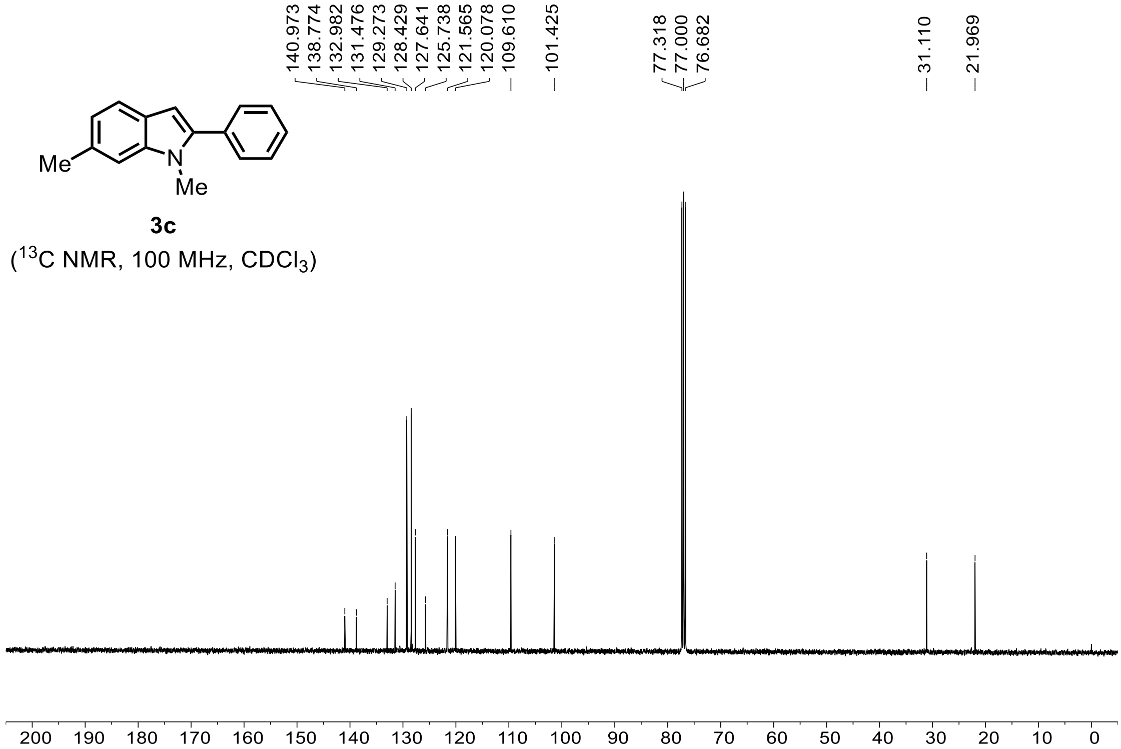
140.973
138.774
132.982
131.476
129.273
128.429
127.641
125.738
121.565
120.078
109.610
101.425
77.318
77.000
76.682
31.110
21.969
Me
N
Me
3c
(^{13}C NMR, 100 MHz, $CDCl_3$)
200 190 180 170 160 150 140 130 120 110 100 90 80 70 60 50 40 30 20 10 0
f1 (ppm)

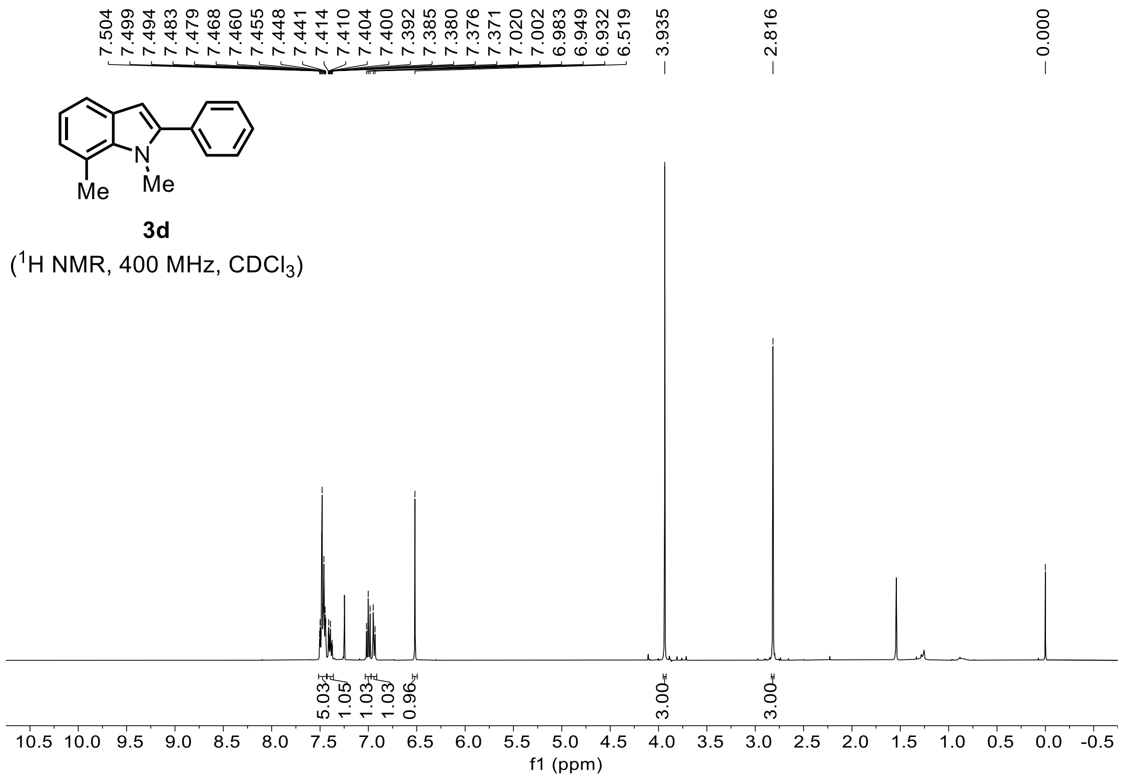

3d

(^{1}H NMR, 400 MHz, $CDCl_3$)

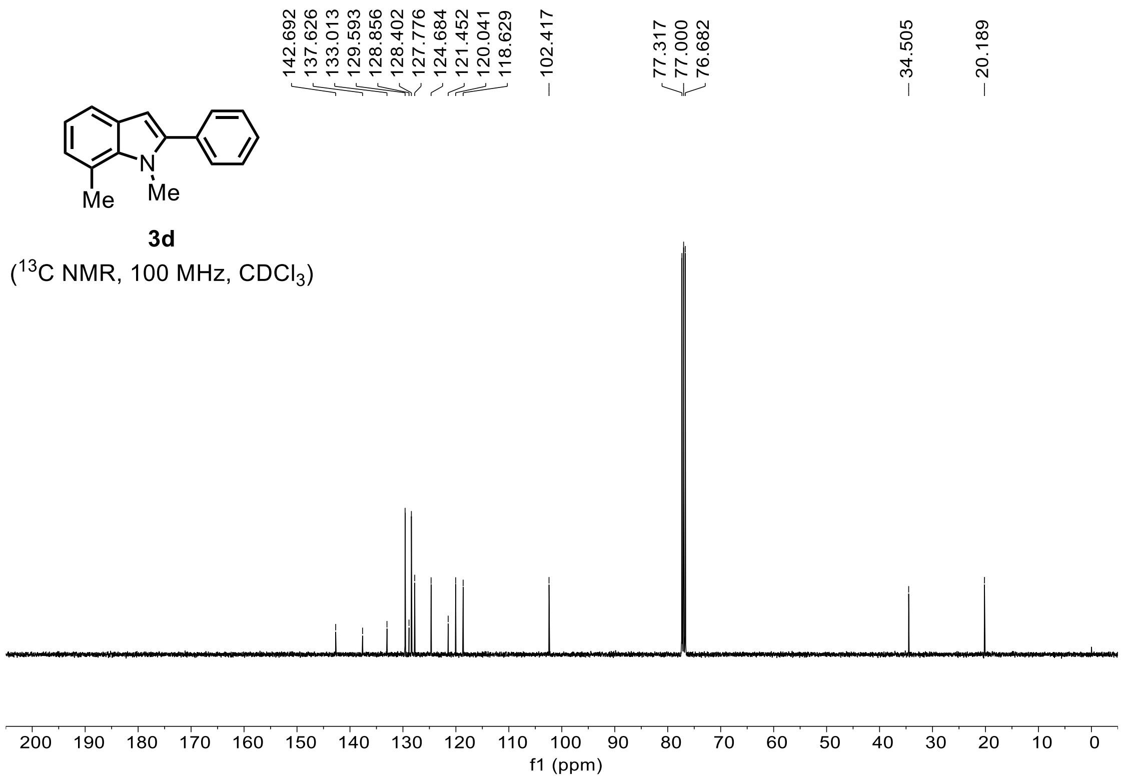

3d

(^{13}C NMR, 100 MHz, $CDCl_3$)

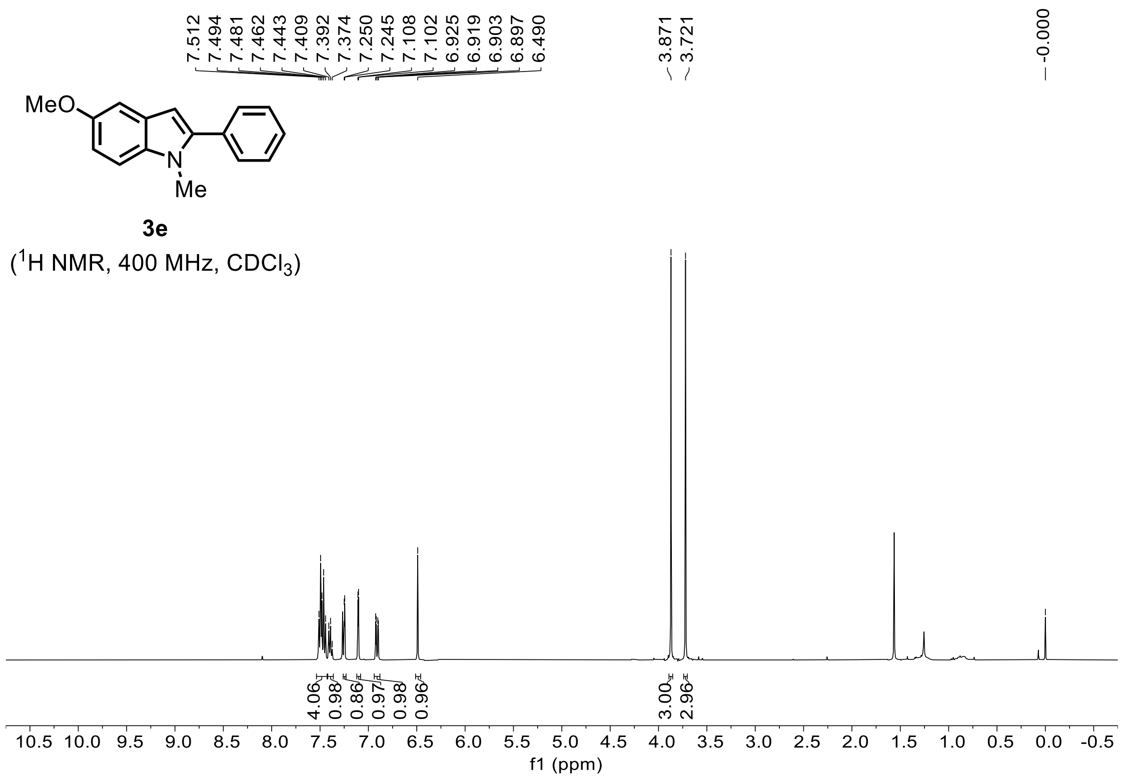
7.512
7.494
7.481
7.462
7.443
7.409
7.392
7.374
7.250
7.245
7.108
7.102
6.925
6.919
6.903
6.897
6.490
3.871
3.721
-0.000
MeO
N
Me
3e
(1H NMR, 400 MHz, CDCl3)
4.06
0.98
0.86
0.97
0.98
0.96
3.00
2.96
10.5 10.0 9.5 9.0 8.5 8.0 7.5 7.0 6.5 6.0 5.5 5.0 4.5 4.0 3.5 3.0 2.5 2.0 1.5 1.0 0.5 0.0 -0.5
f1 (ppm)

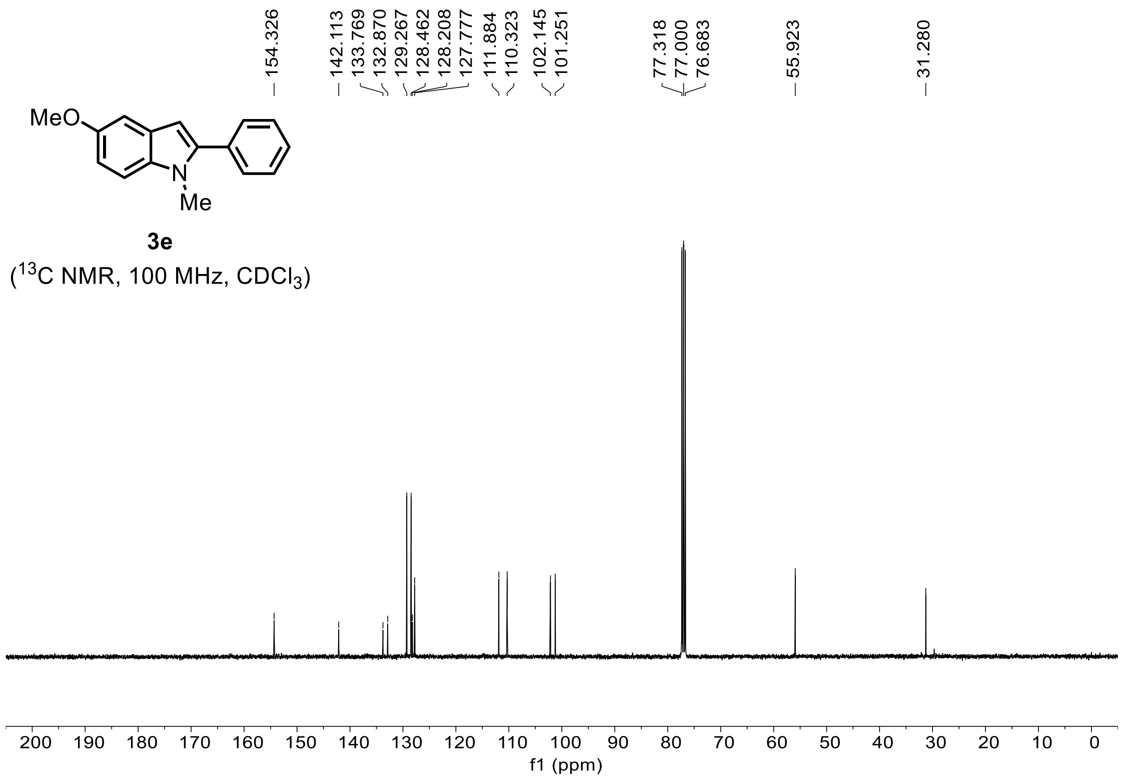
154.326
142.113
133.769
132.870
129.267
128.462
128.208
127.777
111.884
110.323
102.145
101.251
77.318
77.000
76.683
55.923
31.280
MeO
N
Me
3e
(13C NMR, 100 MHz, CDCl3)
200 190 180 170 160 150 140 130 120 110 100 90 80 70 60 50 40 30 20 10 0
f1 (ppm)

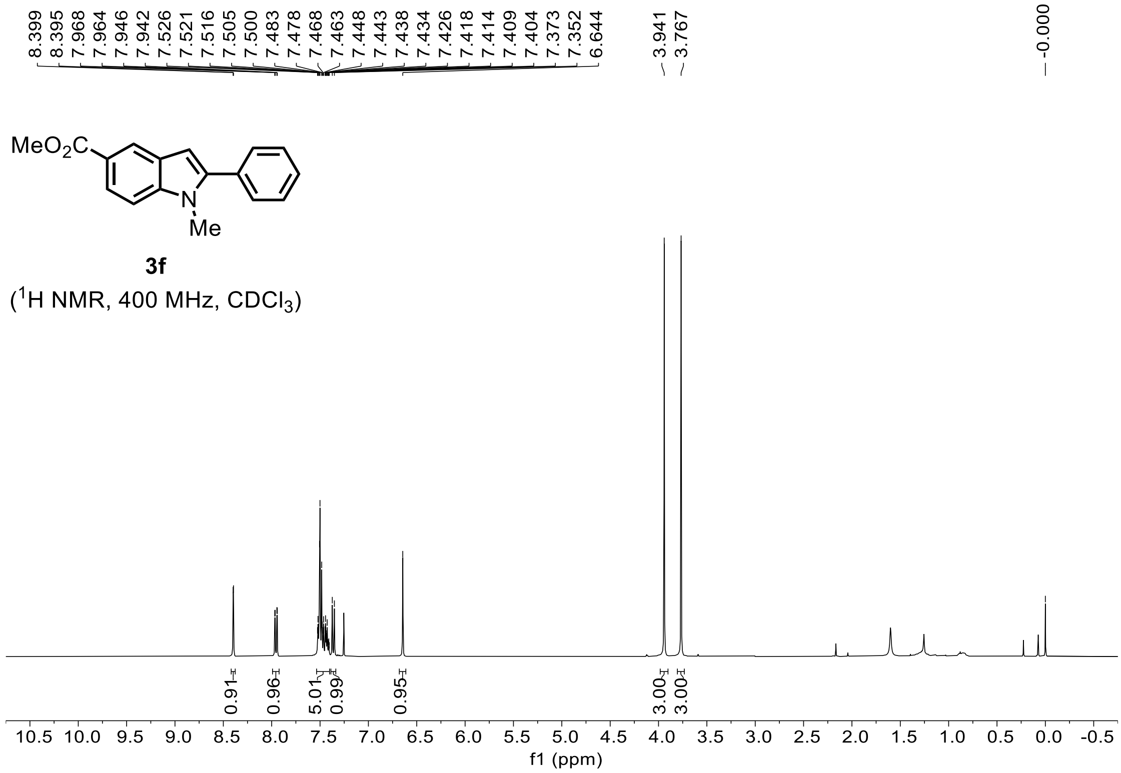

3f

(^{1}H NMR, 400 MHz, $CDCl_3$)

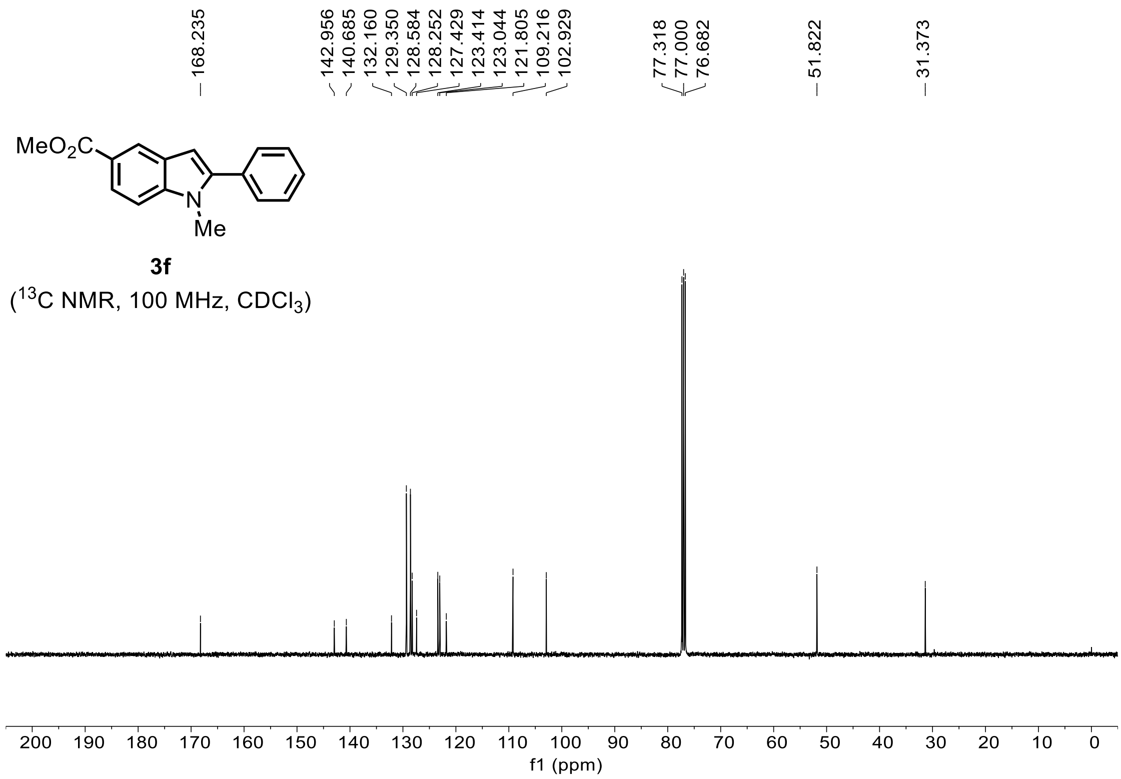

3f

(^{13}C NMR, 100 MHz, $CDCl_3$)

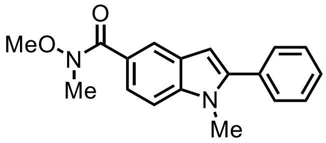

3g
(^{1}H NMR, 400 MHz, $CDCl_3$)

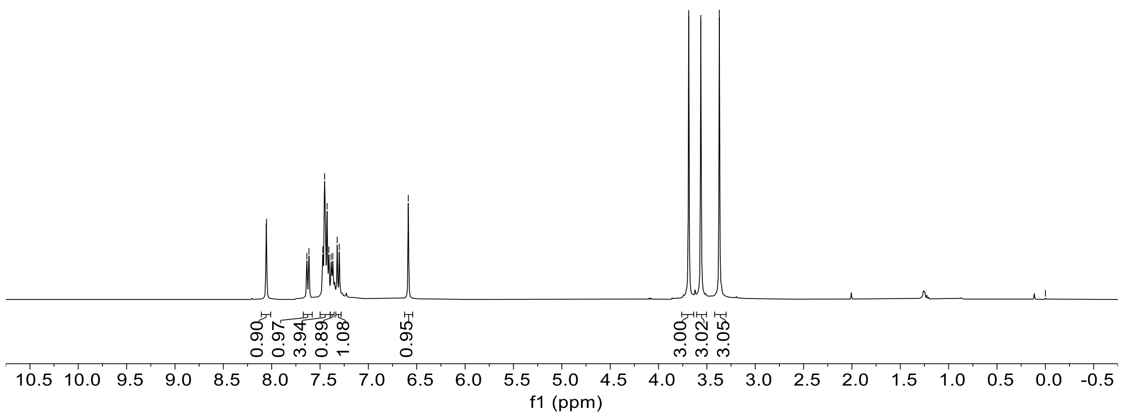

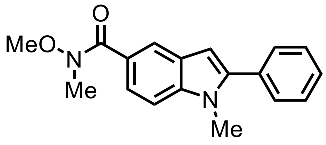

3g
(^{13}C NMR, 100 MHz, $CDCl_3$)

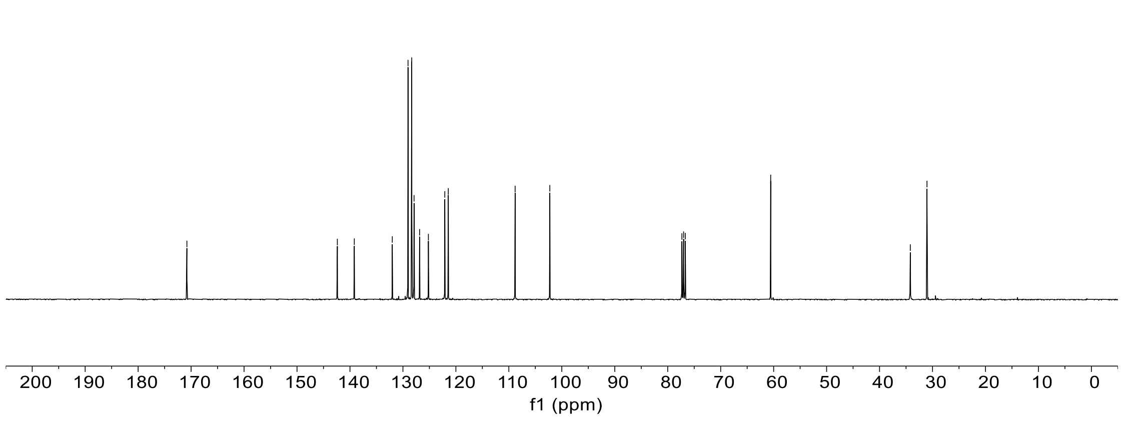

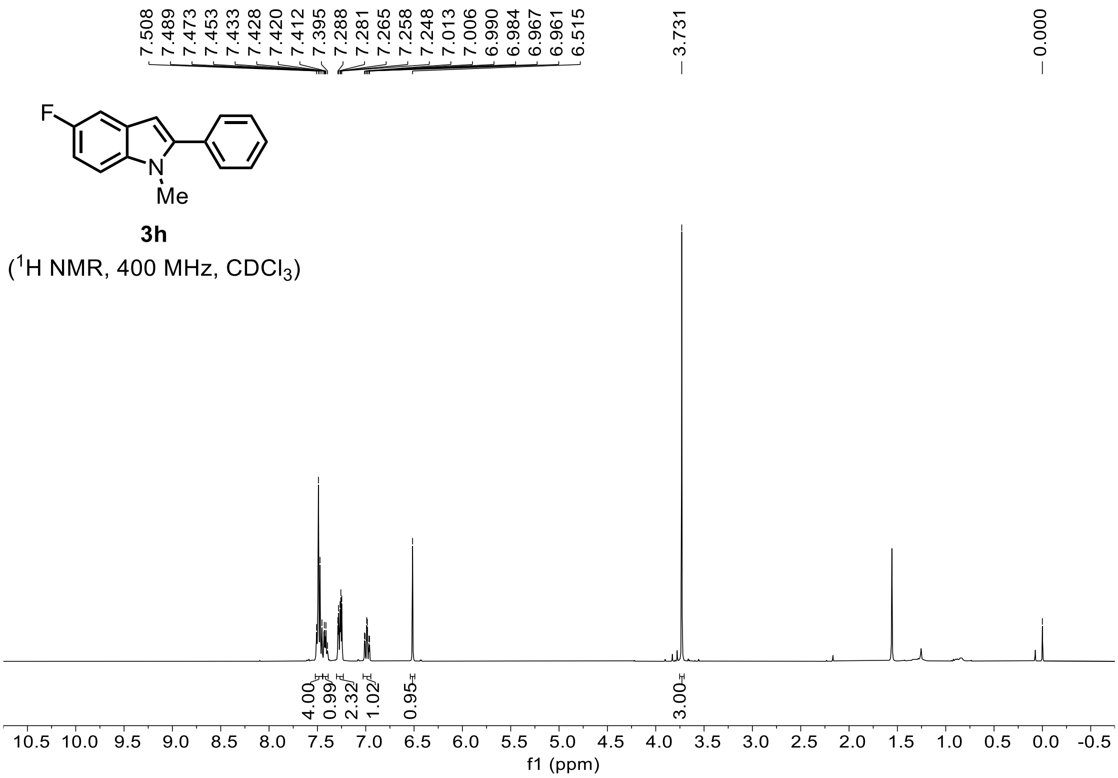

3h

(^{1}H NMR, 400 MHz, $CDCl_3$)

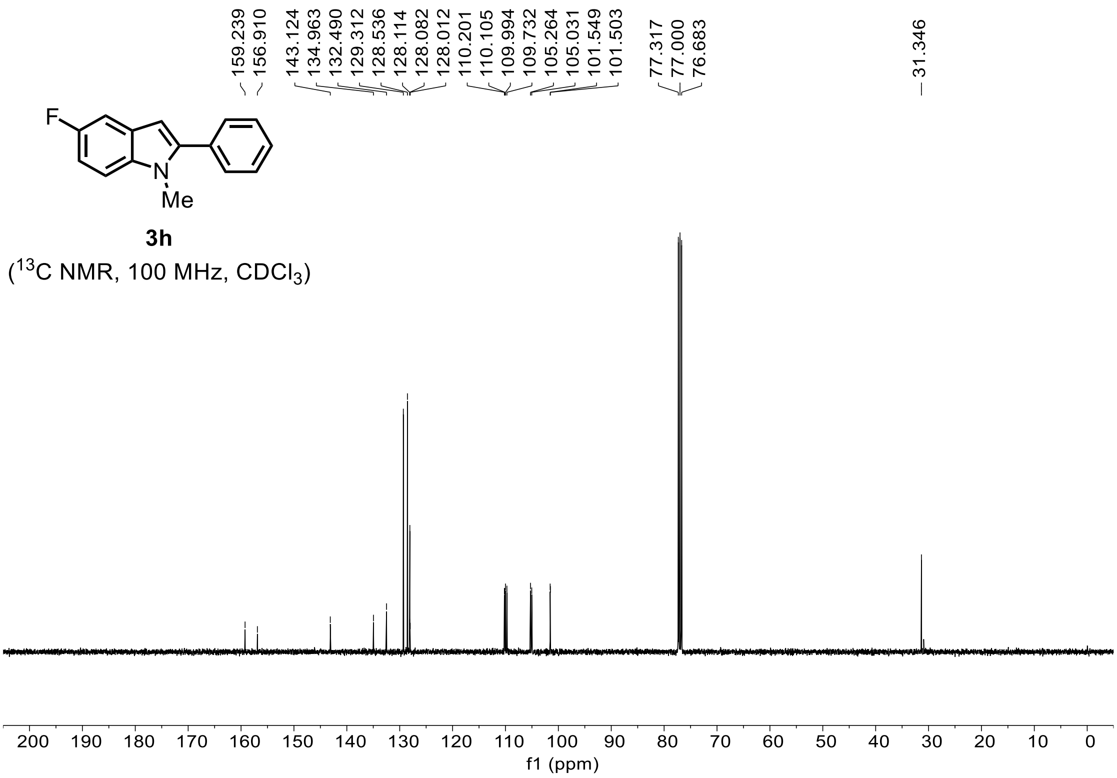

3h

(^{13}C NMR, 100 MHz, $CDCl_3$)

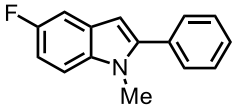

3h

(^{19}F NMR, 376 MHz, $CDCl_3$)

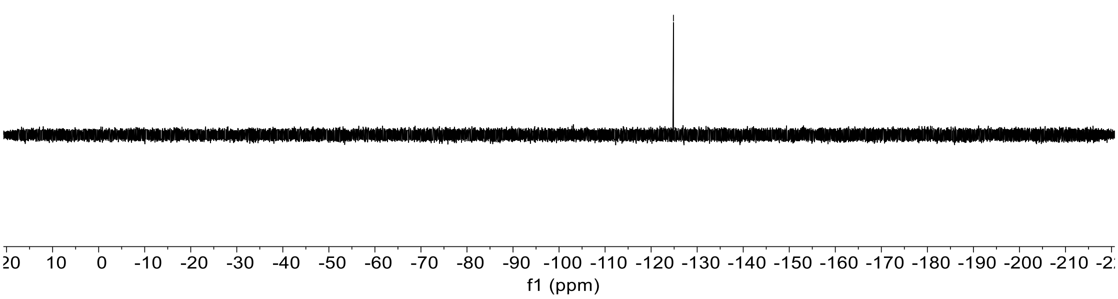

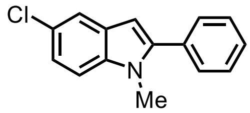

3i

(^{1}H NMR, 400 MHz, $CDCl_3$)

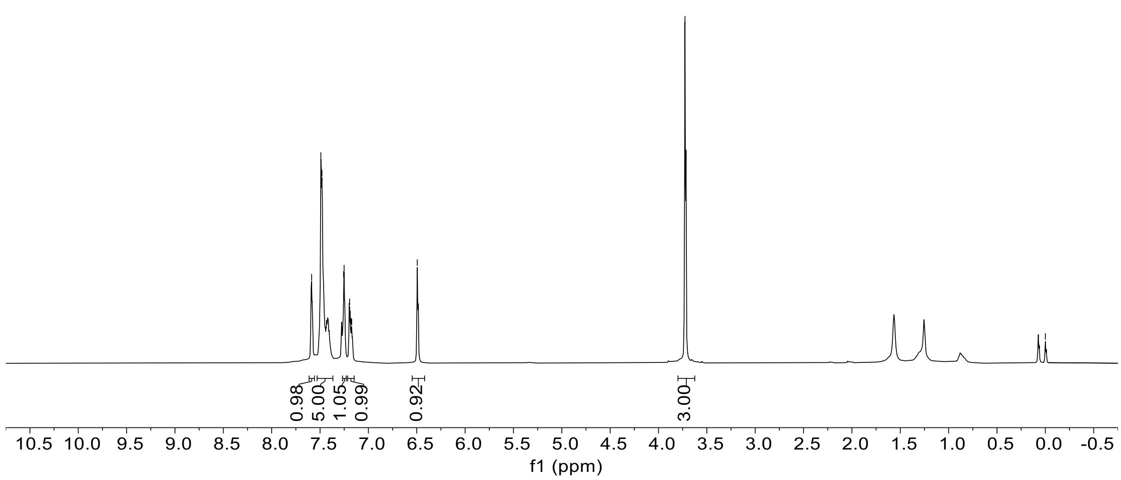

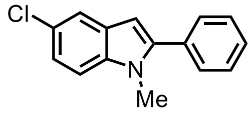

3i

(^{13}C NMR, 100 MHz, $CDCl_3$)

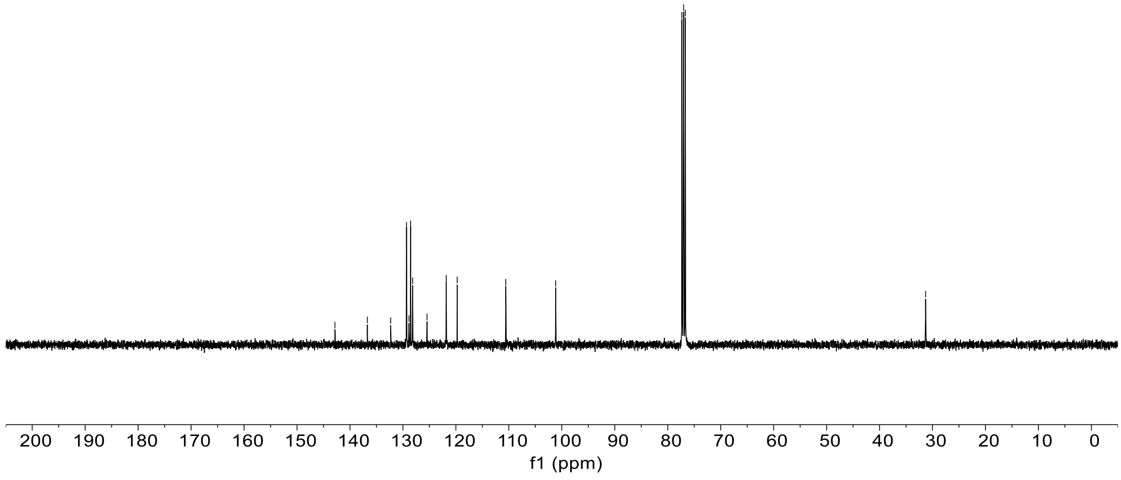

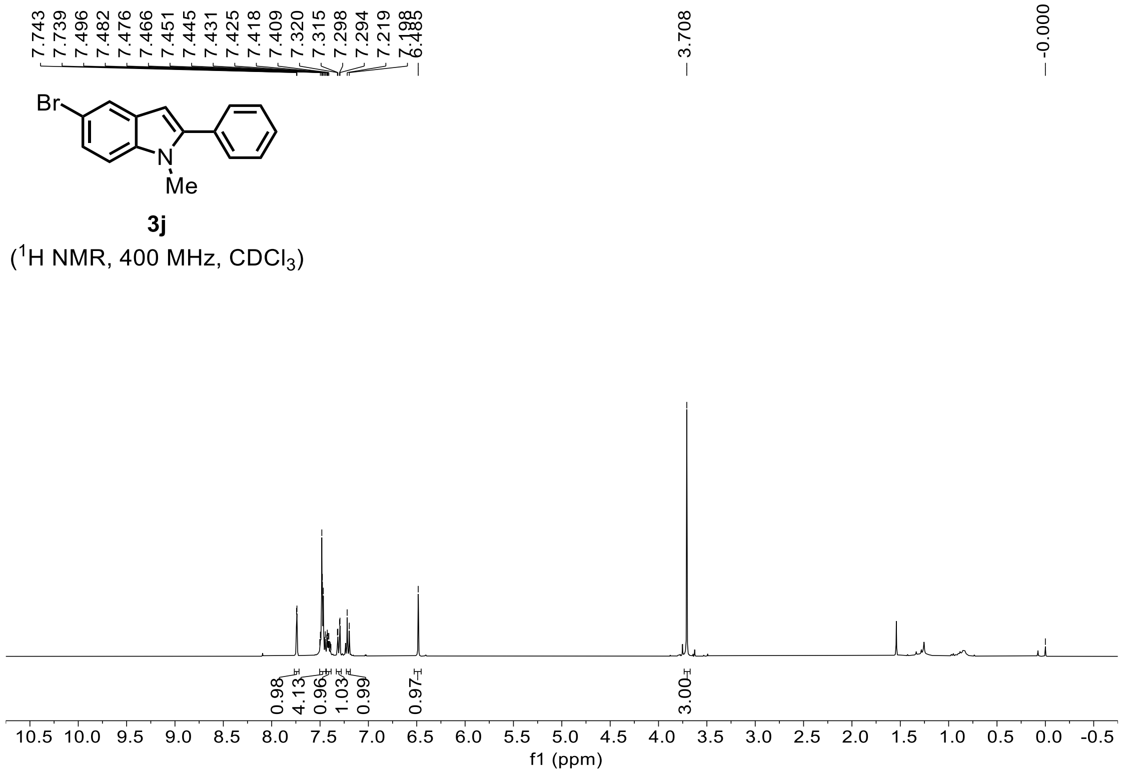

3j

(^{1}H NMR, 400 MHz, $CDCl_3$)

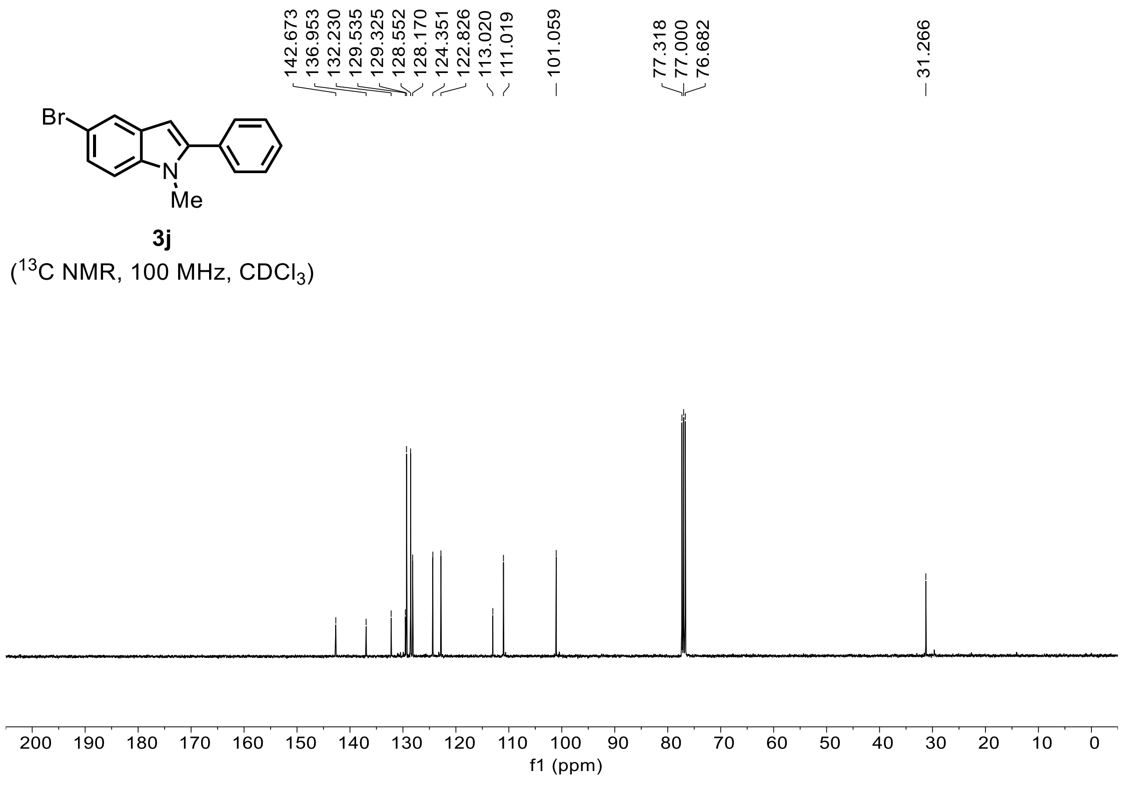

3j

(^{13}C NMR, 100 MHz, $CDCl_3$)

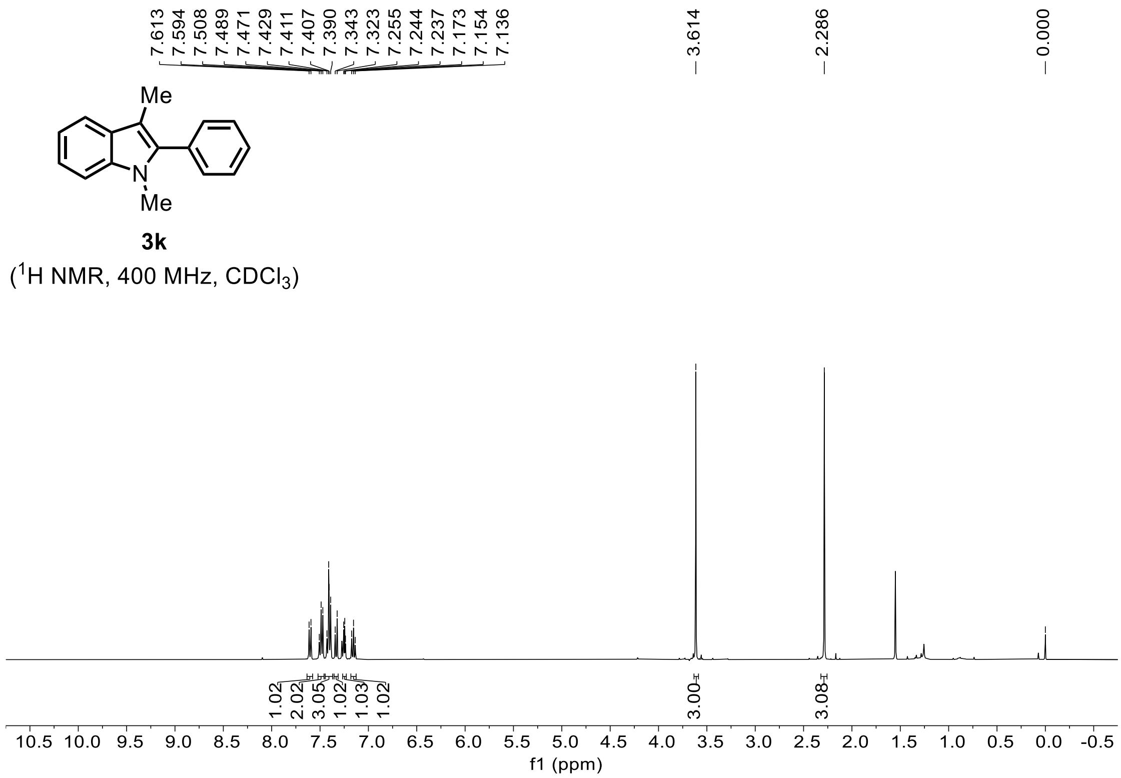

3k

(^{1}H NMR, 400 MHz, $CDCl_3$)

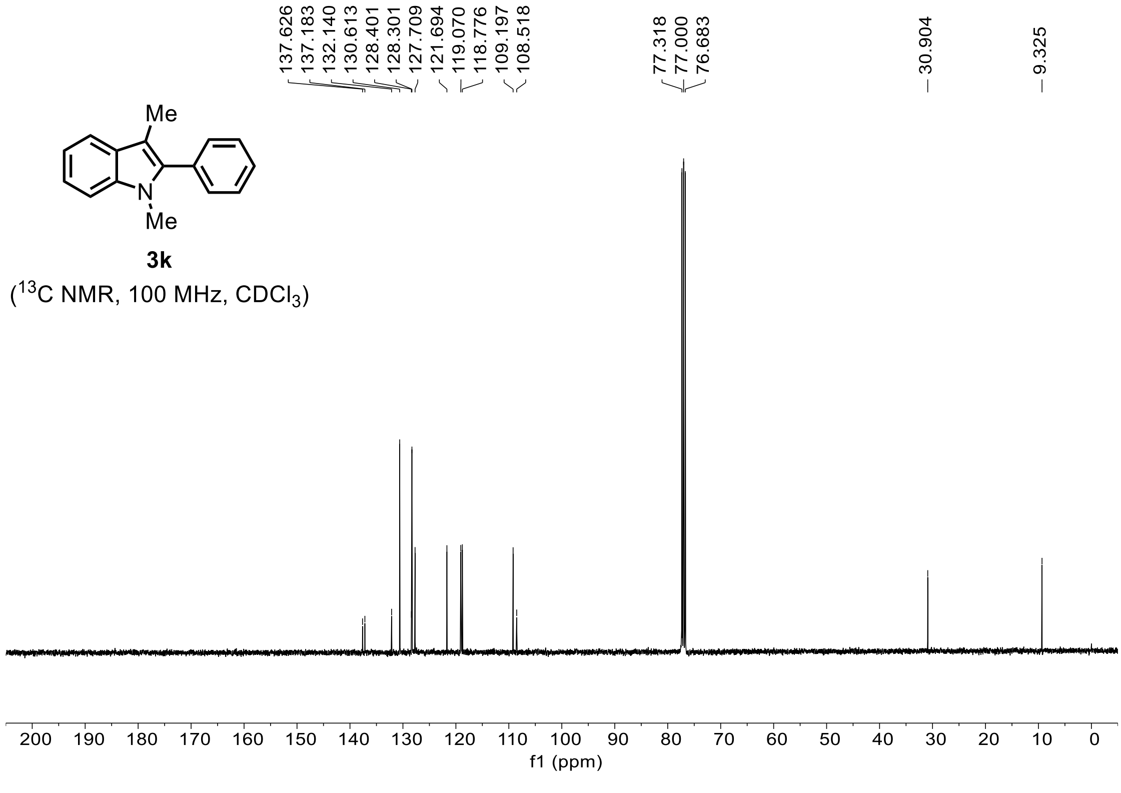

3k

(^{13}C NMR, 100 MHz, $CDCl_3$)

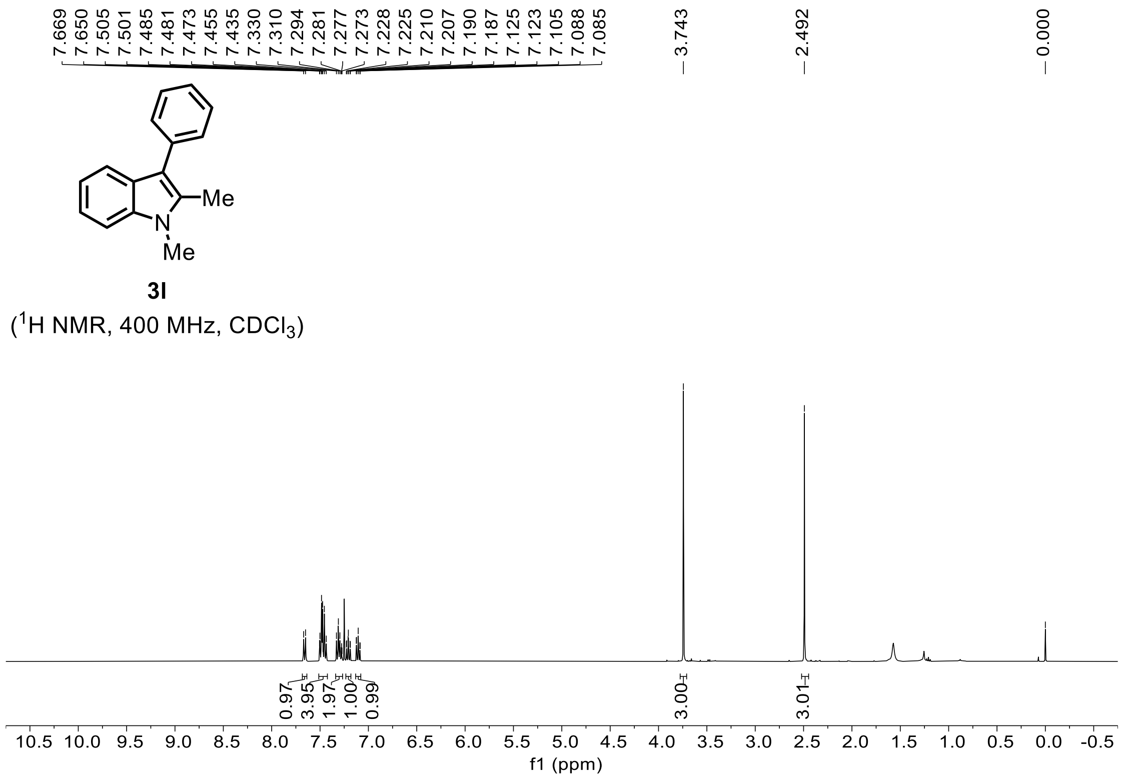

3l

(^{1}H NMR, 400 MHz, $CDCl_3$)

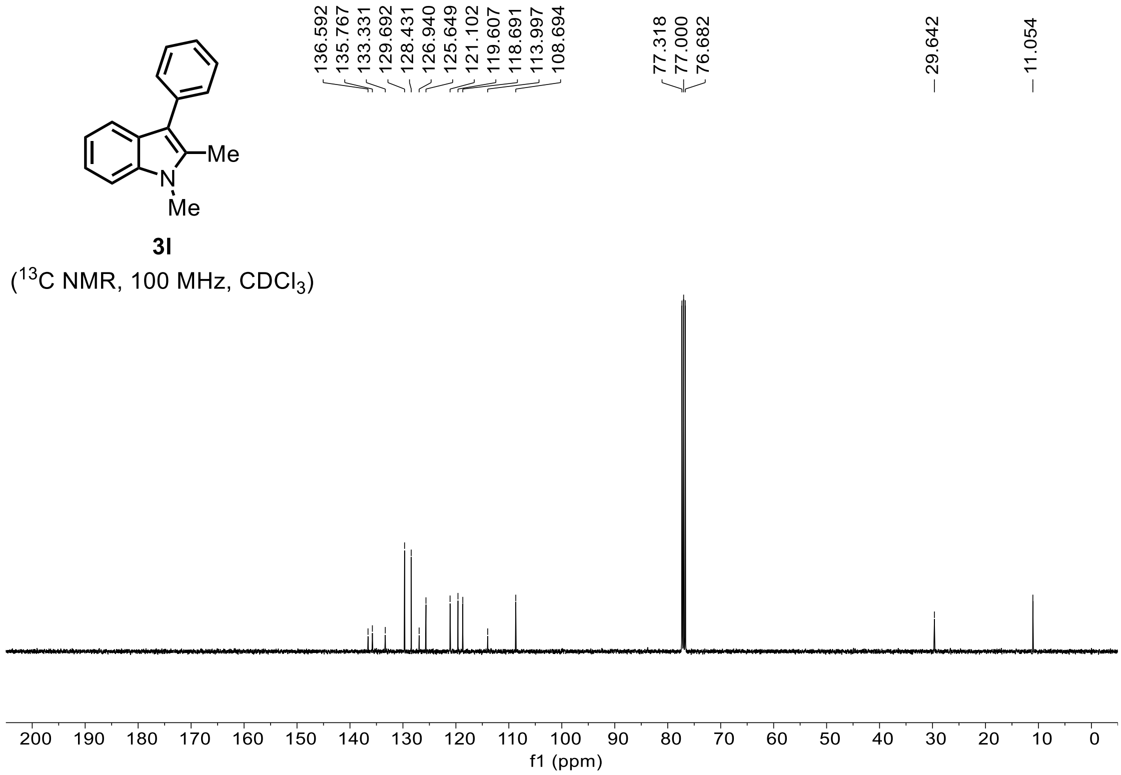

3l

(^{13}C NMR, 100 MHz, $CDCl_3$)

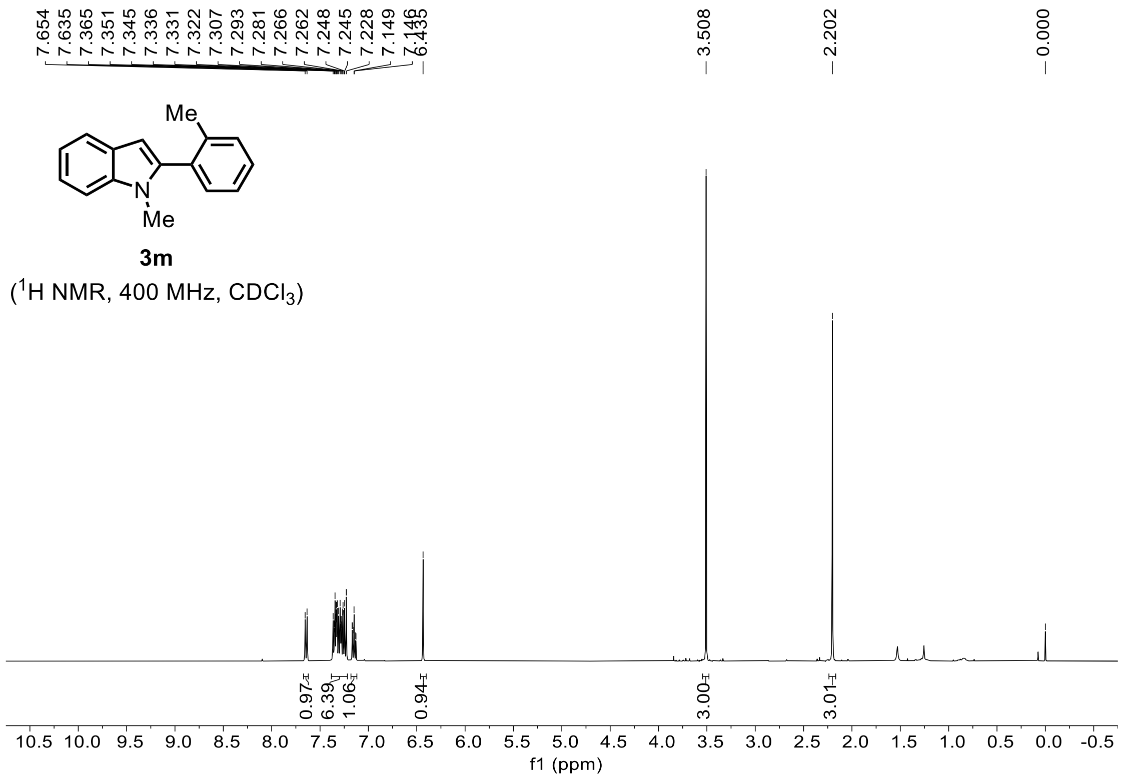

3m

(^{1}H NMR, 400 MHz, $CDCl_3$)

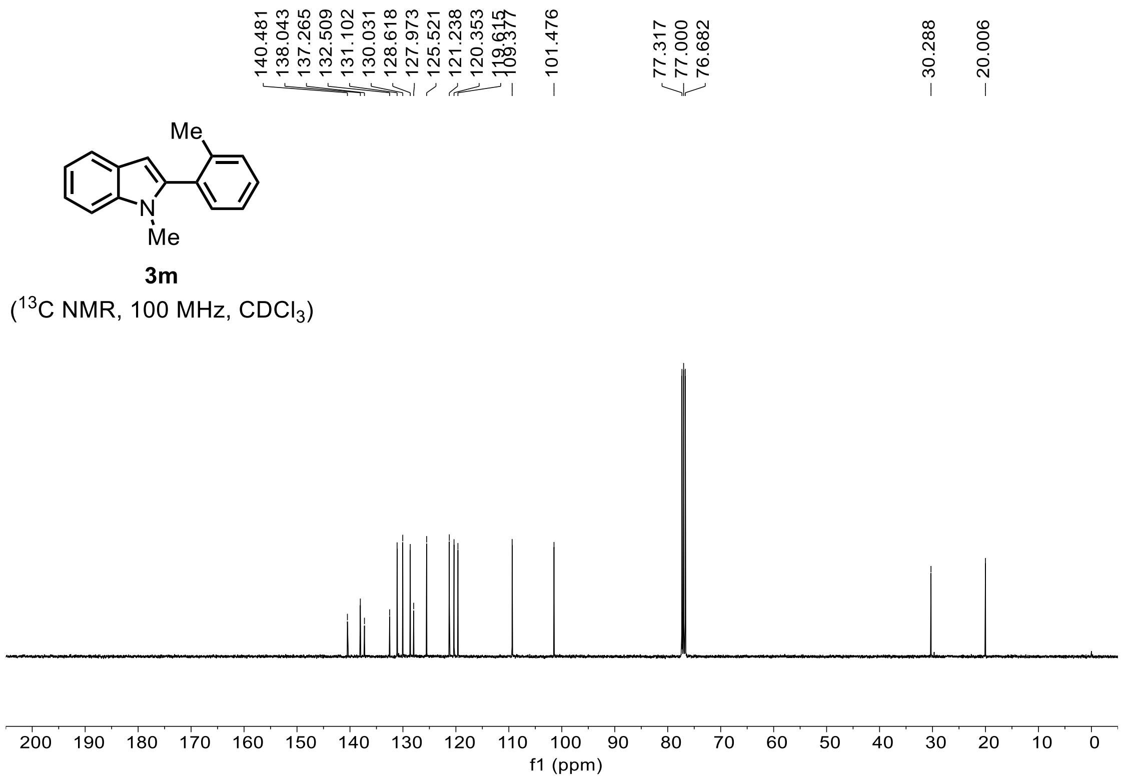

3m

(^{13}C NMR, 100 MHz, $CDCl_3$)

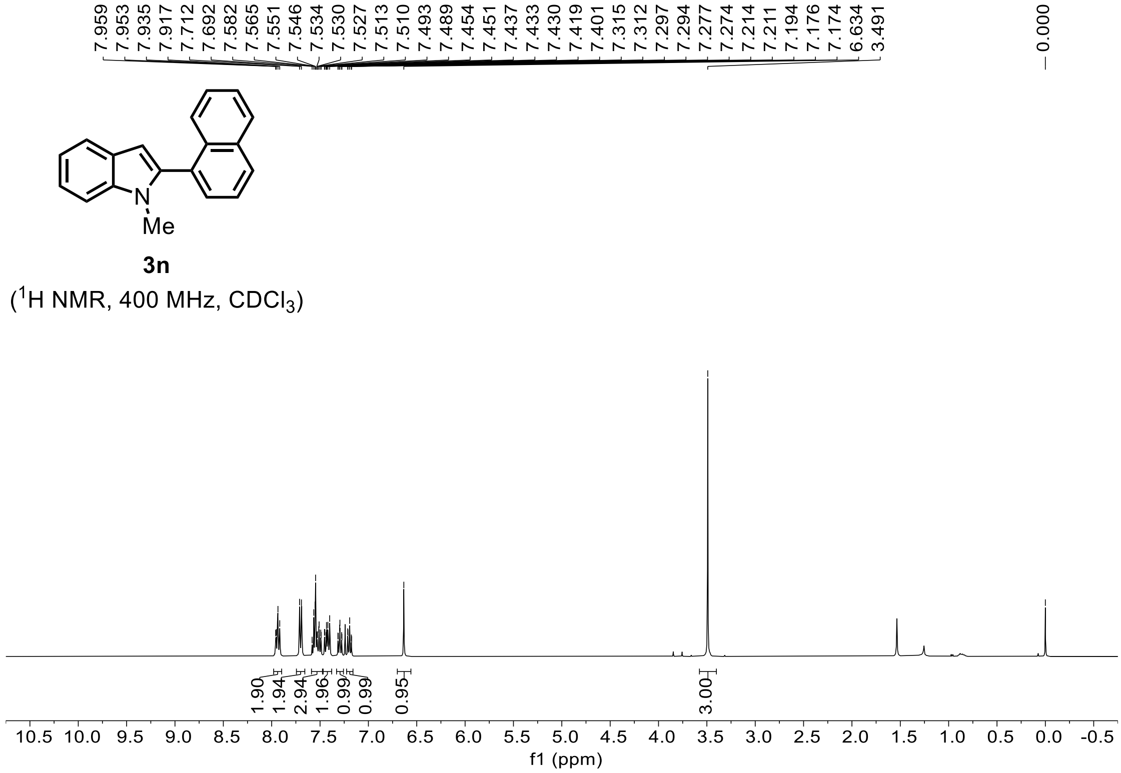

3n

(^{1}H NMR, 400 MHz, $CDCl_3$)

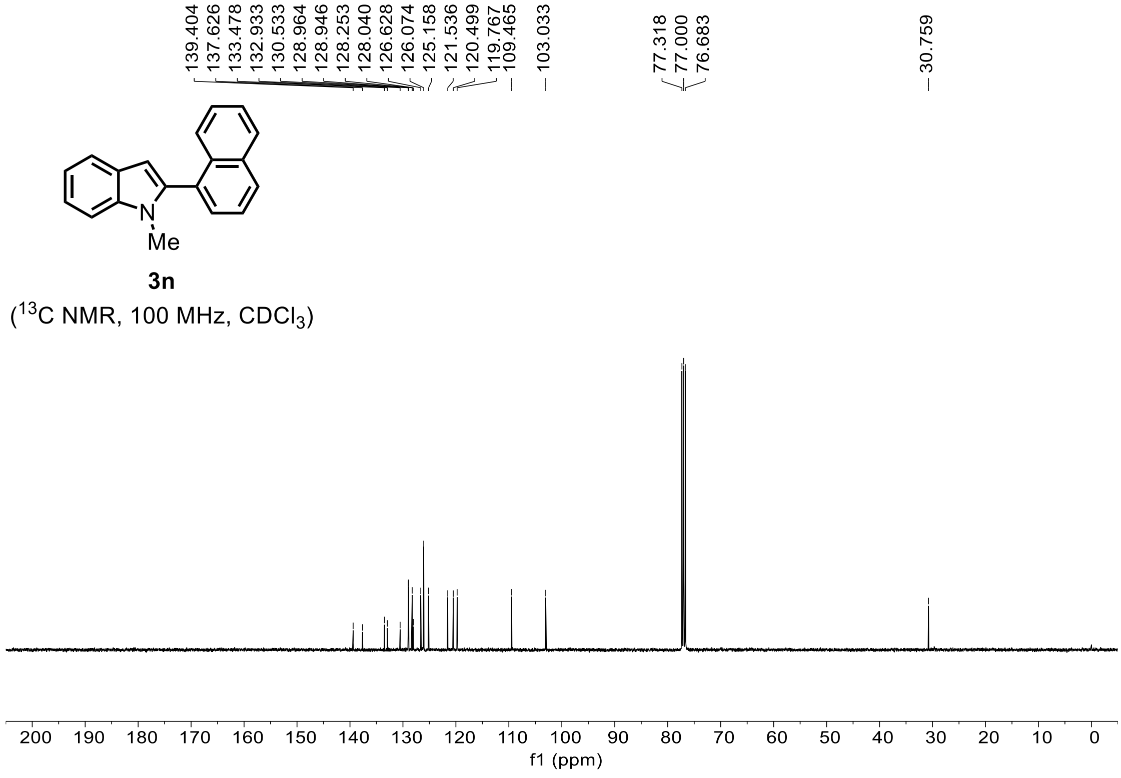

3n

(^{13}C NMR, 100 MHz, $CDCl_3$)

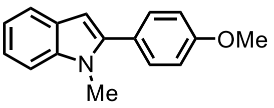

3o

(^{1}H NMR, 400 MHz, $CDCl_3$)

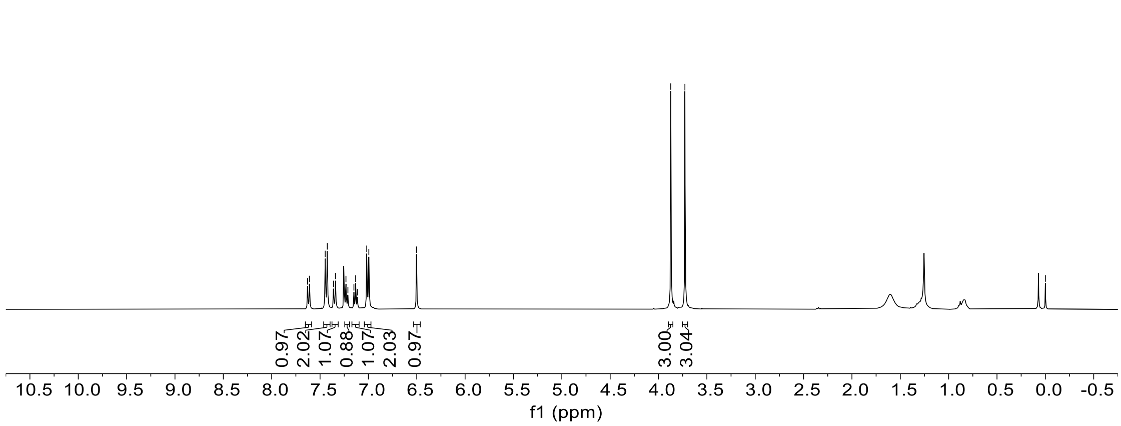

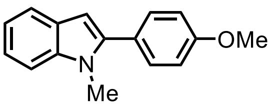

3o

(^{13}C NMR, 100 MHz, $CDCl_3$)

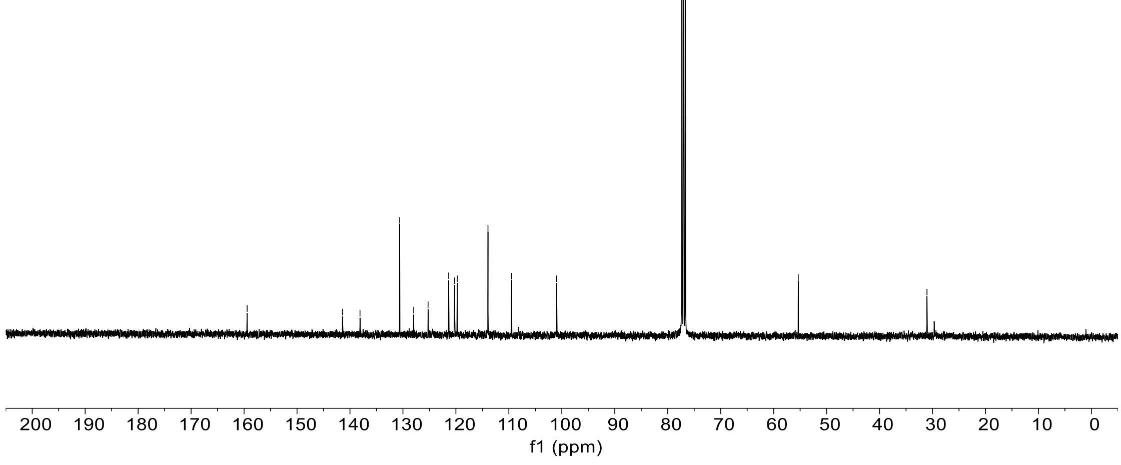

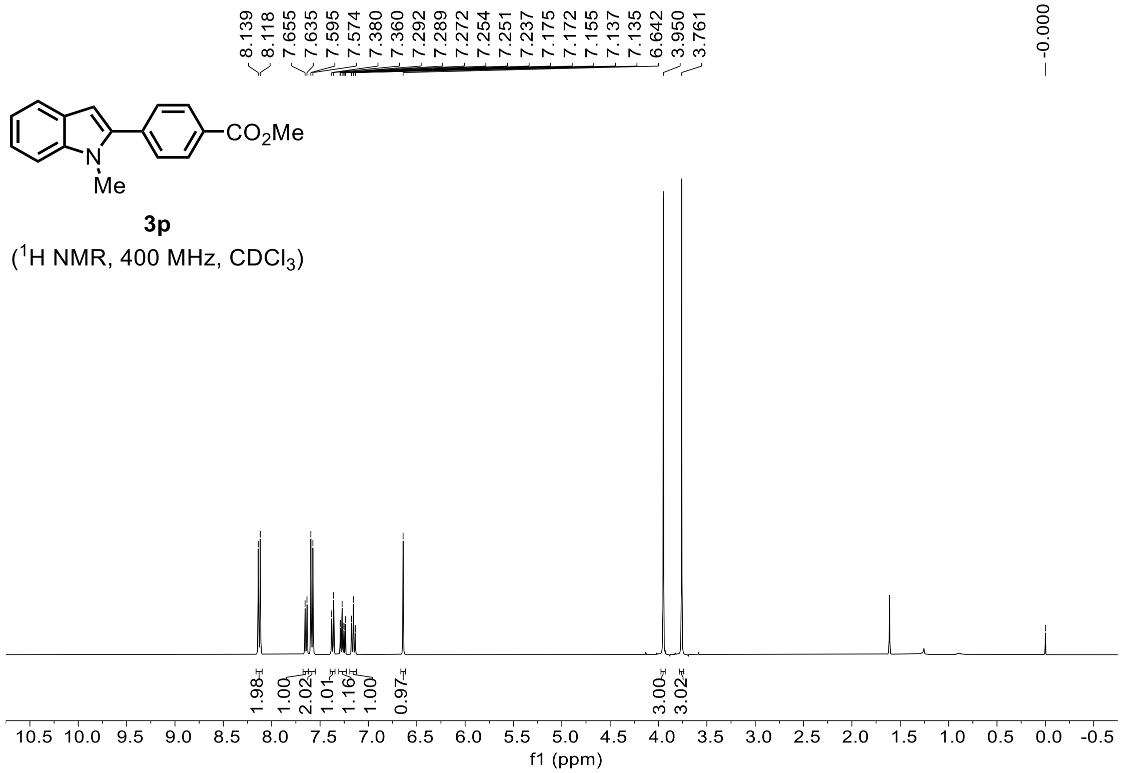
8.139
8.118
7.655
7.635
7.595
7.574
7.380
7.360
7.292
7.289
7.272
7.254
7.251
7.237
7.175
7.172
7.155
7.137
7.135
6.642
3.950
3.761
-0.000
CO2Me
N
Me
3p
(1H NMR, 400 MHz, CDCl3)
1.98
1.00
2.02
1.01
1.16
1.00
0.97
3.00
3.02
10.5 10.0 9.5 9.0 8.5 8.0 7.5 7.0 6.5 6.0 5.5 5.0 4.5 4.0 3.5 3.0 2.5 2.0 1.5 1.0 0.5 0.0 -0.5
f1 (ppm)

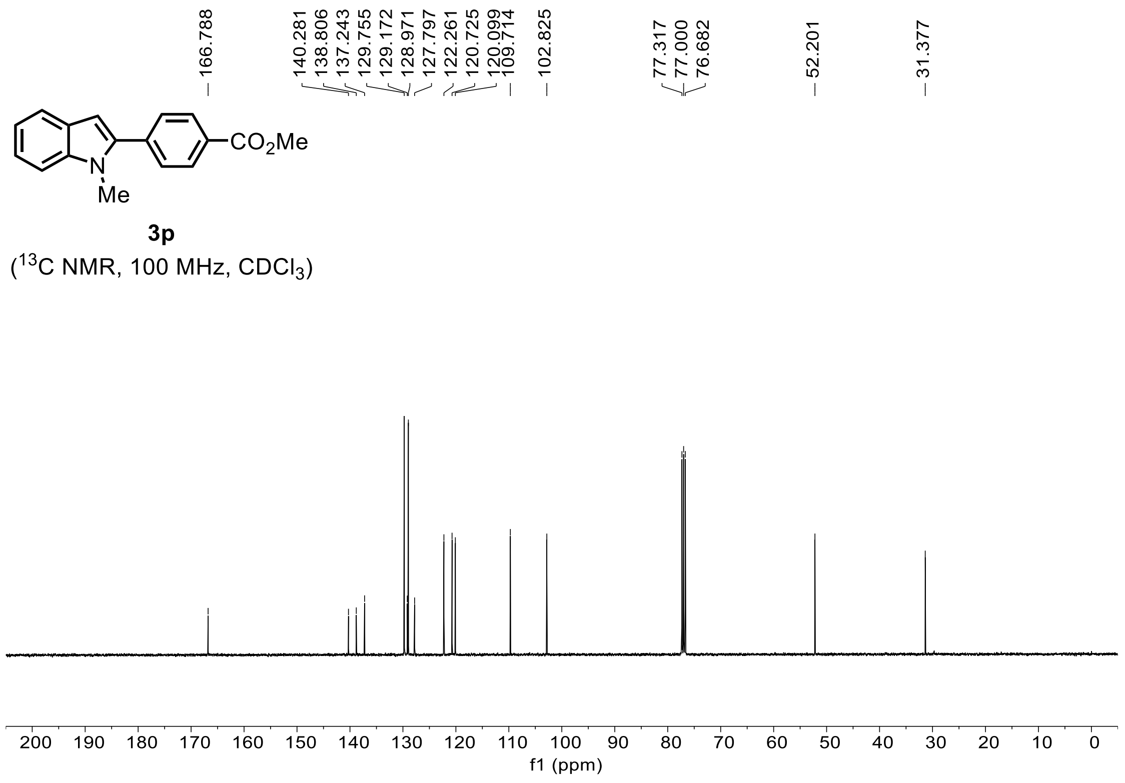
166.788
140.281
138.806
137.243
129.755
129.172
128.971
127.797
122.261
120.725
120.099
109.714
102.825
77.317
77.000
76.682
52.201
31.377
CO2Me
N
Me
3p
(13C NMR, 100 MHz, CDCl3)
200 190 180 170 160 150 140 130 120 110 100 90 80 70 60 50 40 30 20 10 0
f1 (ppm)

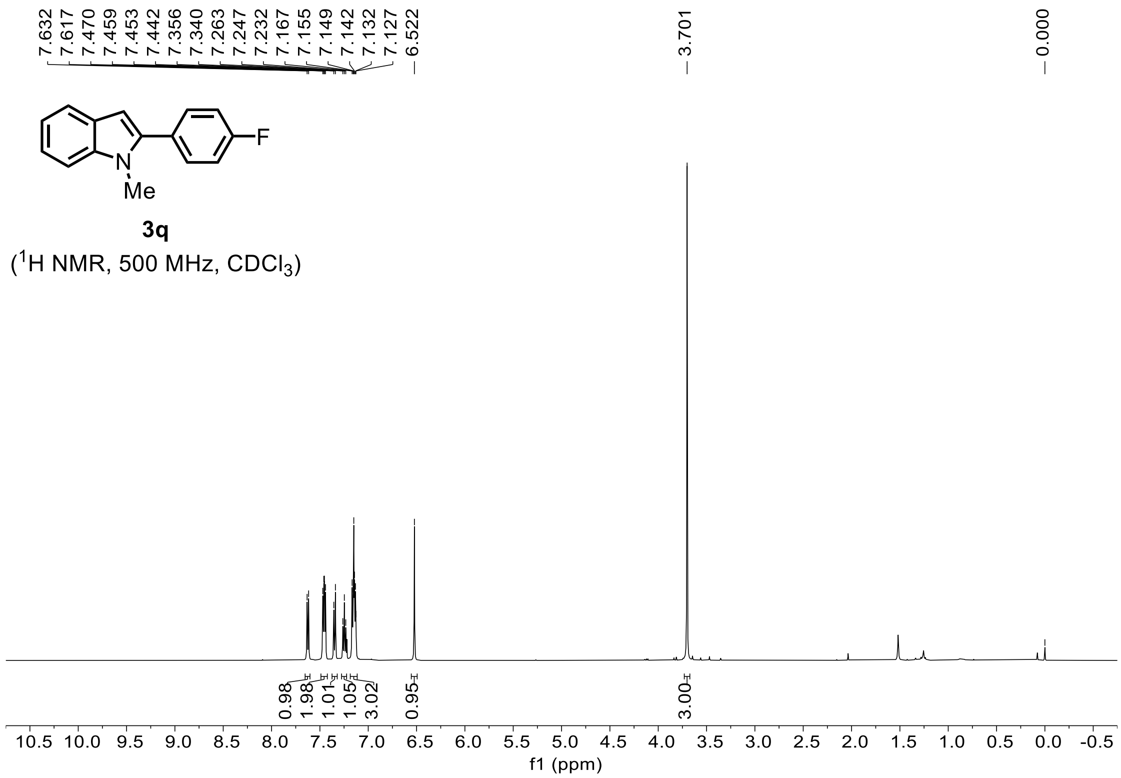

3q
(^{1}H NMR, 500 MHz, $CDCl_3$)

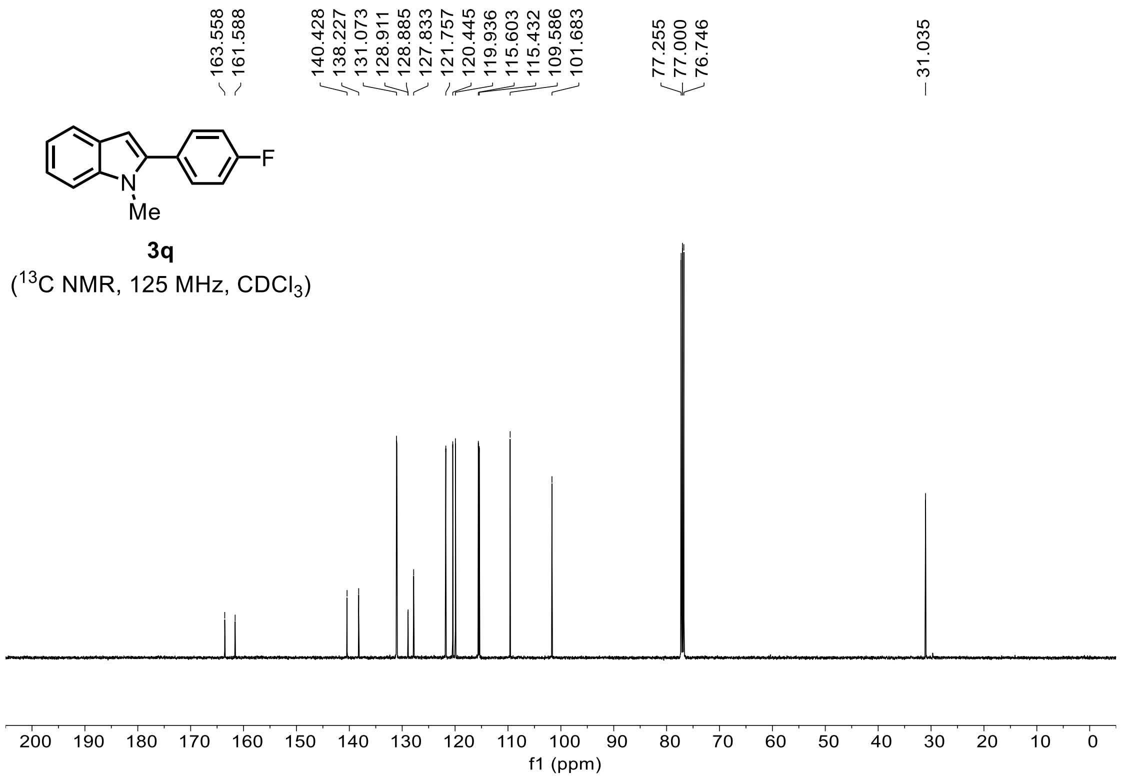

3q
(^{13}C NMR, 125 MHz, $CDCl_3$)

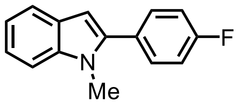

3q

(^{19}F NMR, 470 MHz, $CDCl_3$)

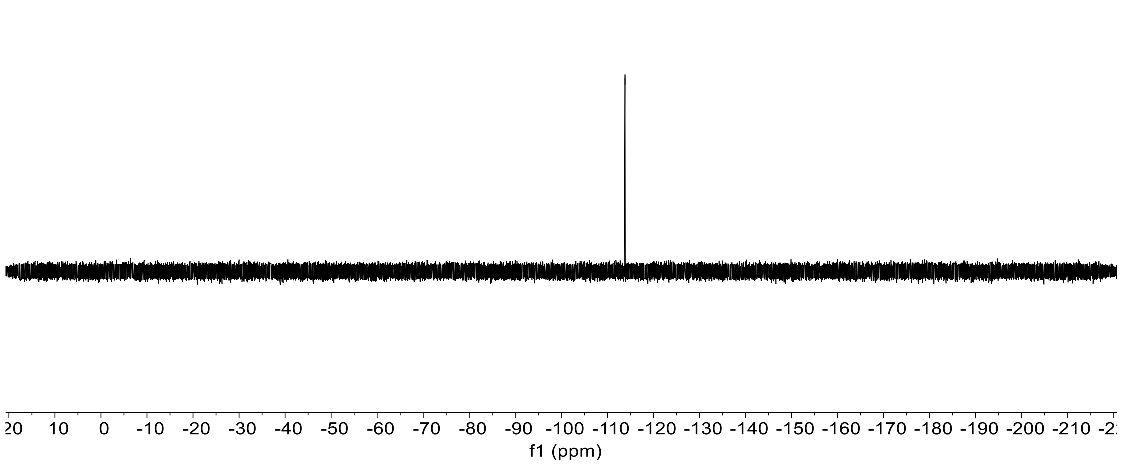

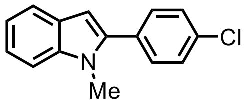

3r

(^{1}H NMR, 400 MHz, CDCl$_3$)

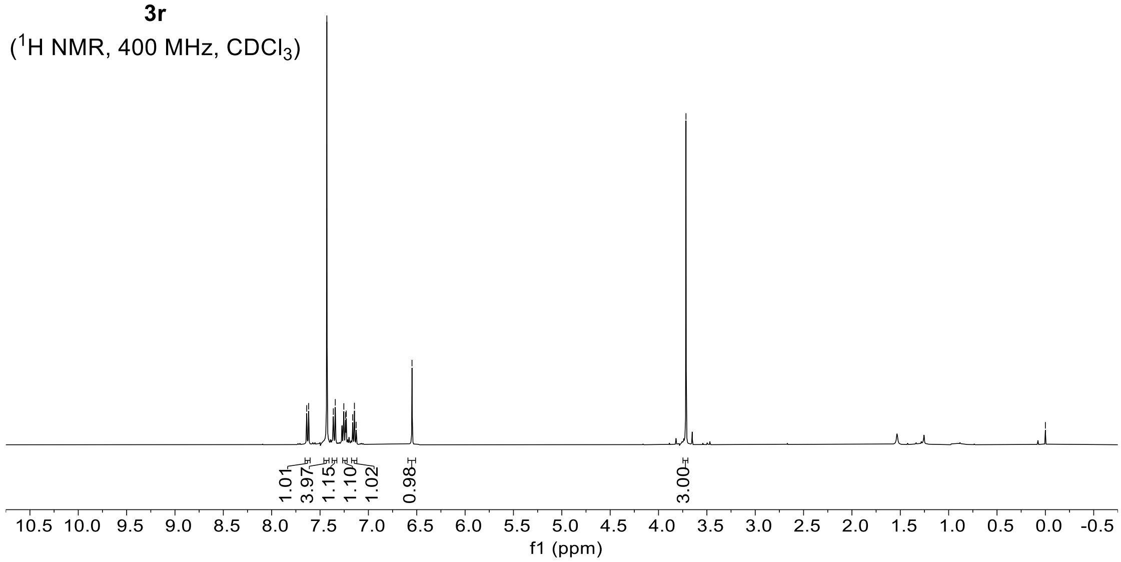

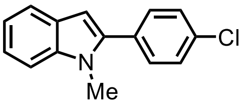

3r

(^{13}C NMR, 100 MHz, CDCl$_3$)

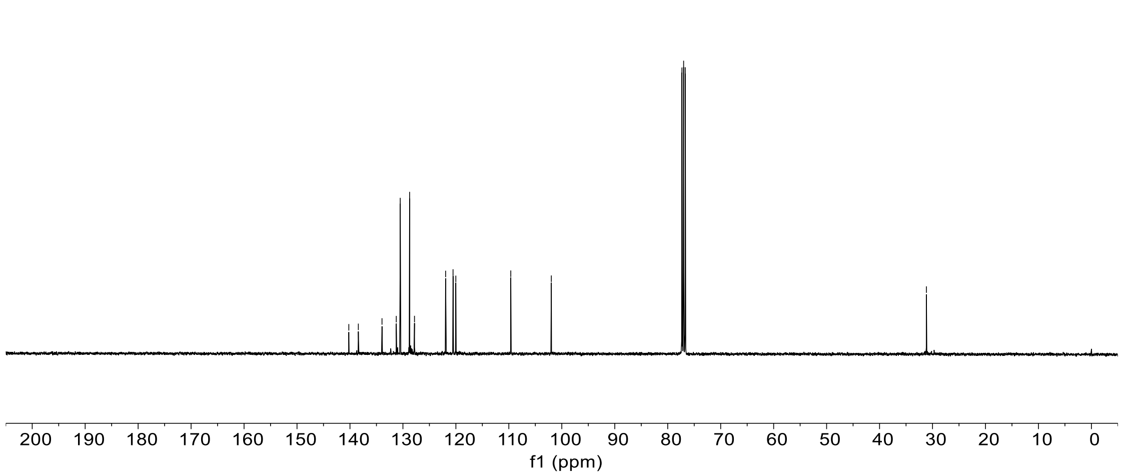

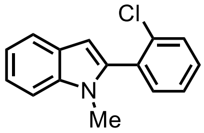

3s

(^{1}H NMR, 400 MHz, $CDCl_3$)

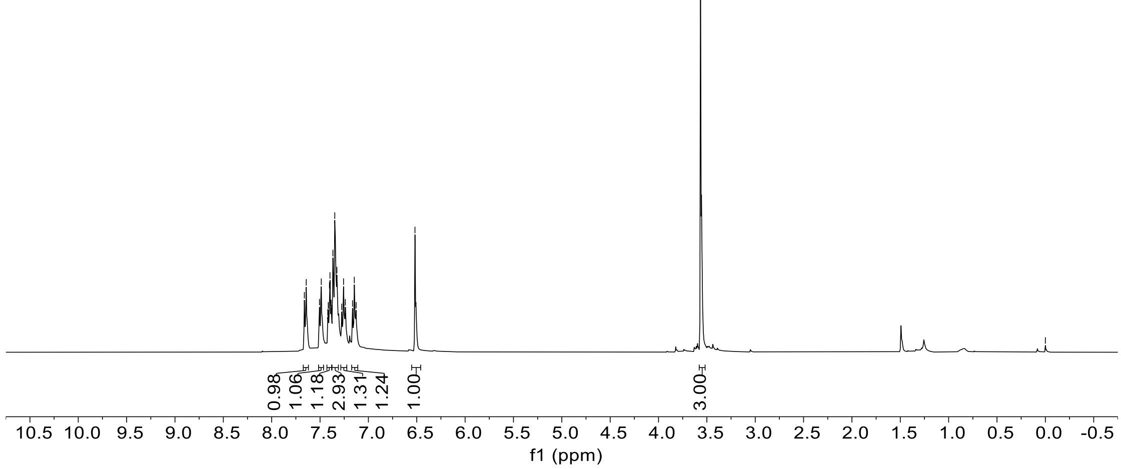

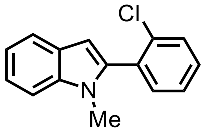

3s

(^{13}C NMR, 100 MHz, $CDCl_3$)

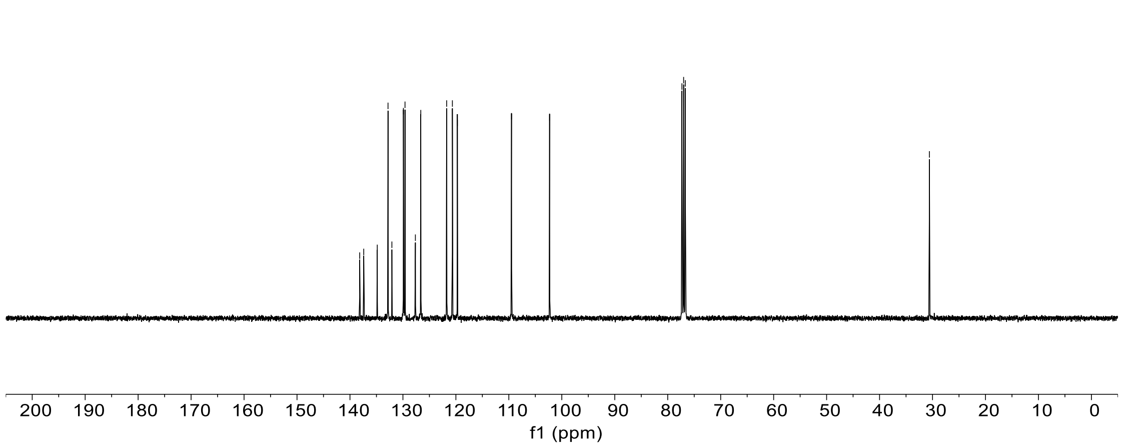

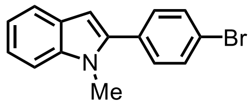

3t

(^{1}H NMR, 400 MHz, $CDCl_3$)

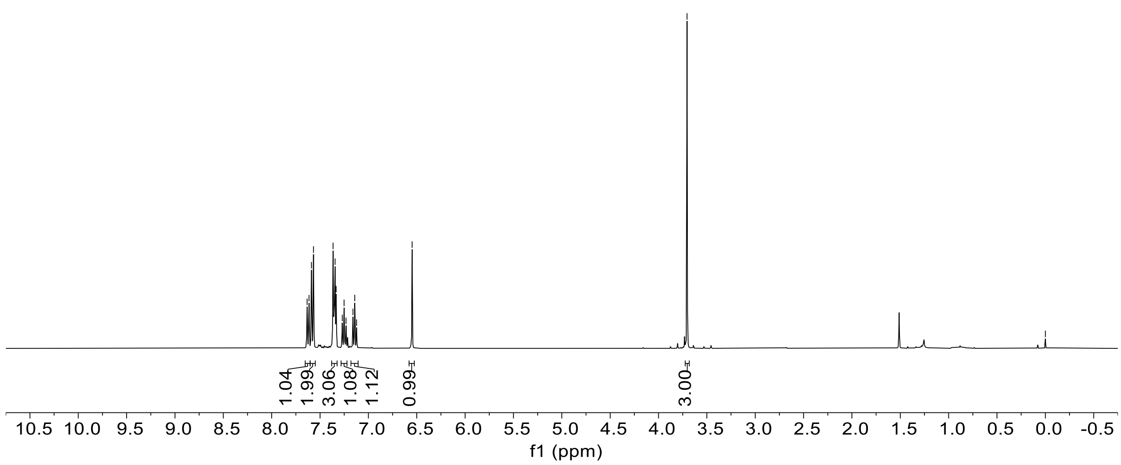

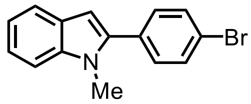

3t

(^{13}C NMR, 100 MHz, $CDCl_3$)

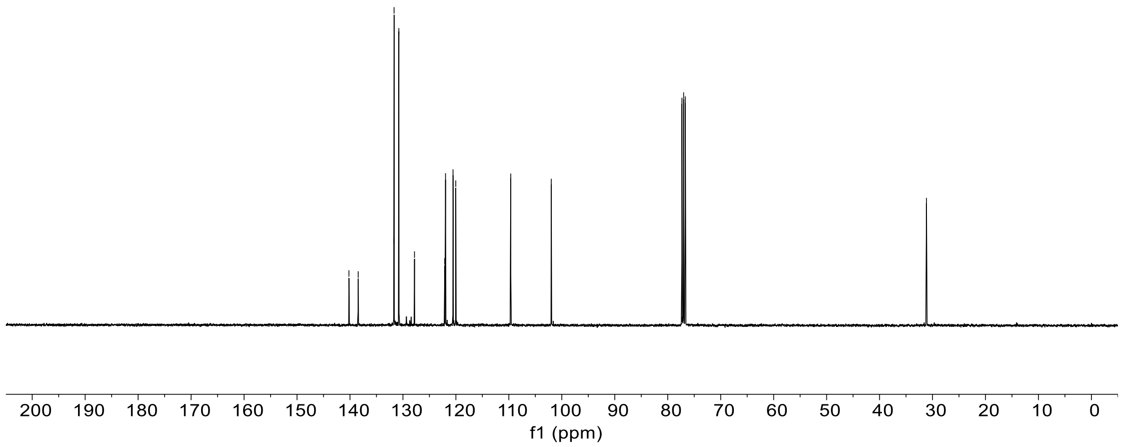

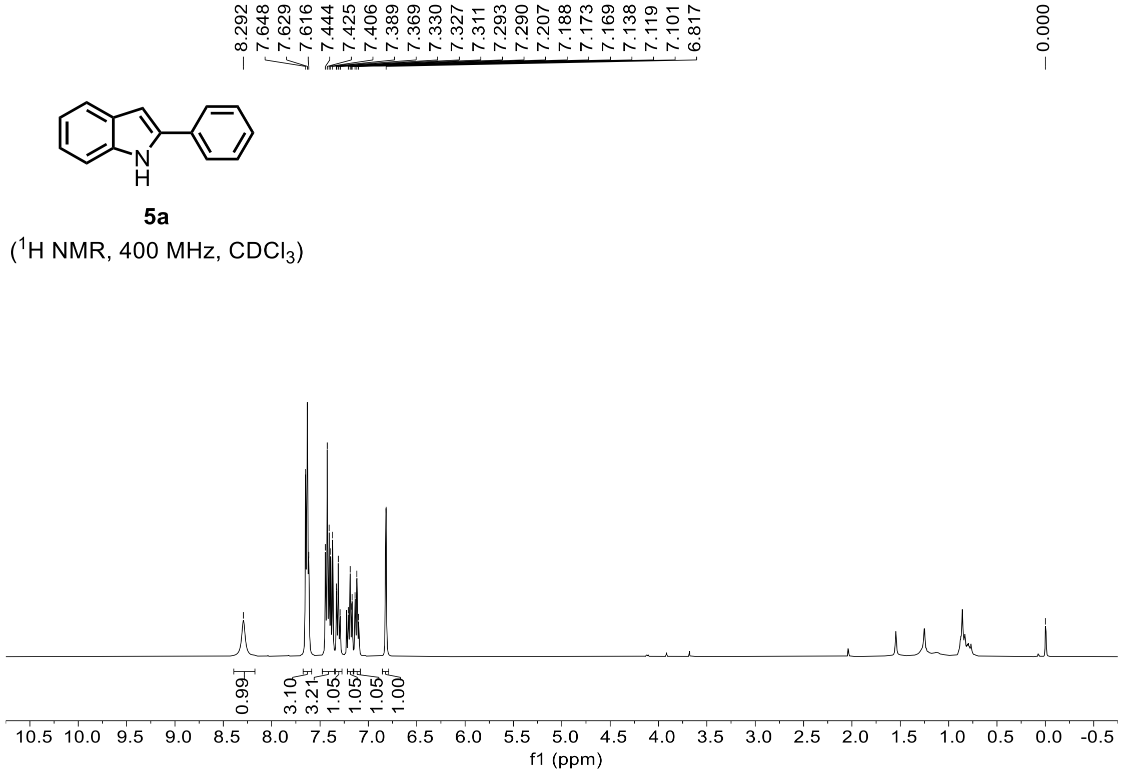

5a

(^{1}H NMR, 400 MHz, $CDCl_3$)

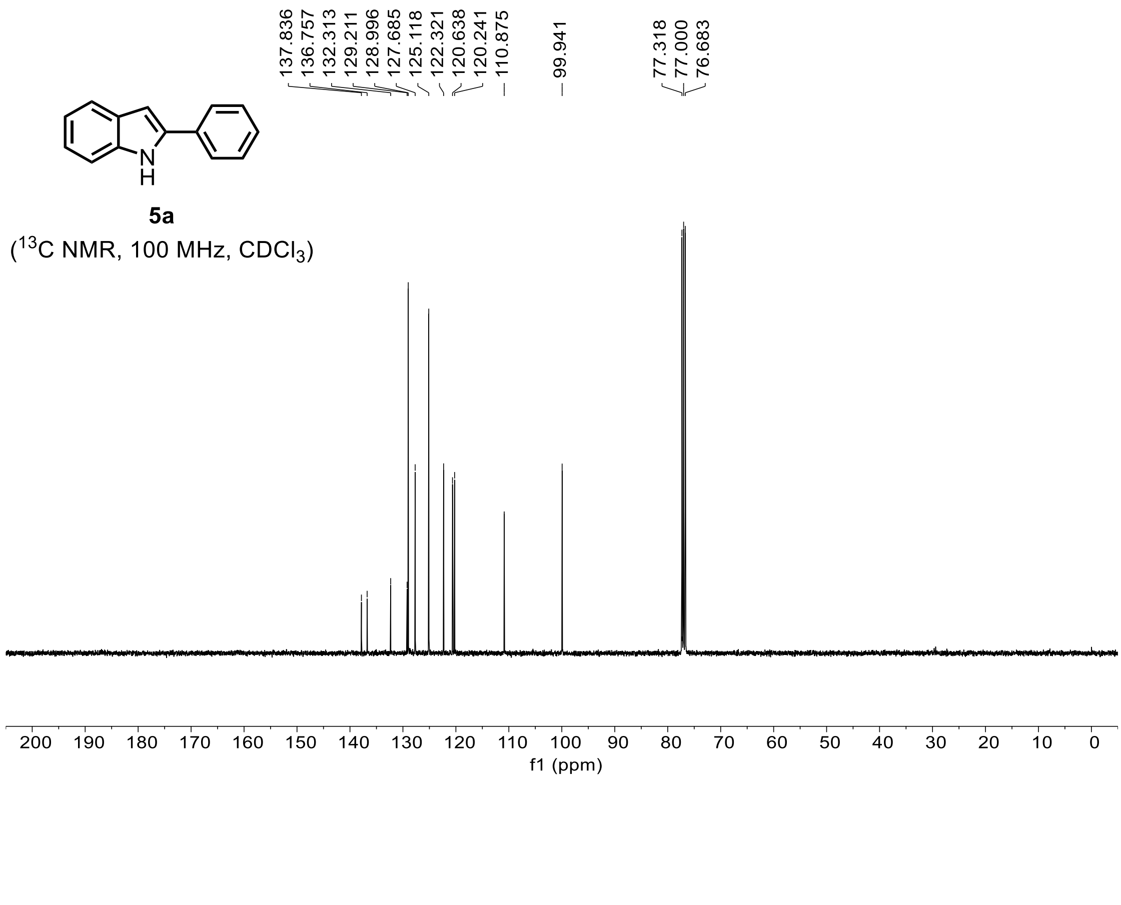

5a

(^{13}C NMR, 100 MHz, $CDCl_3$)

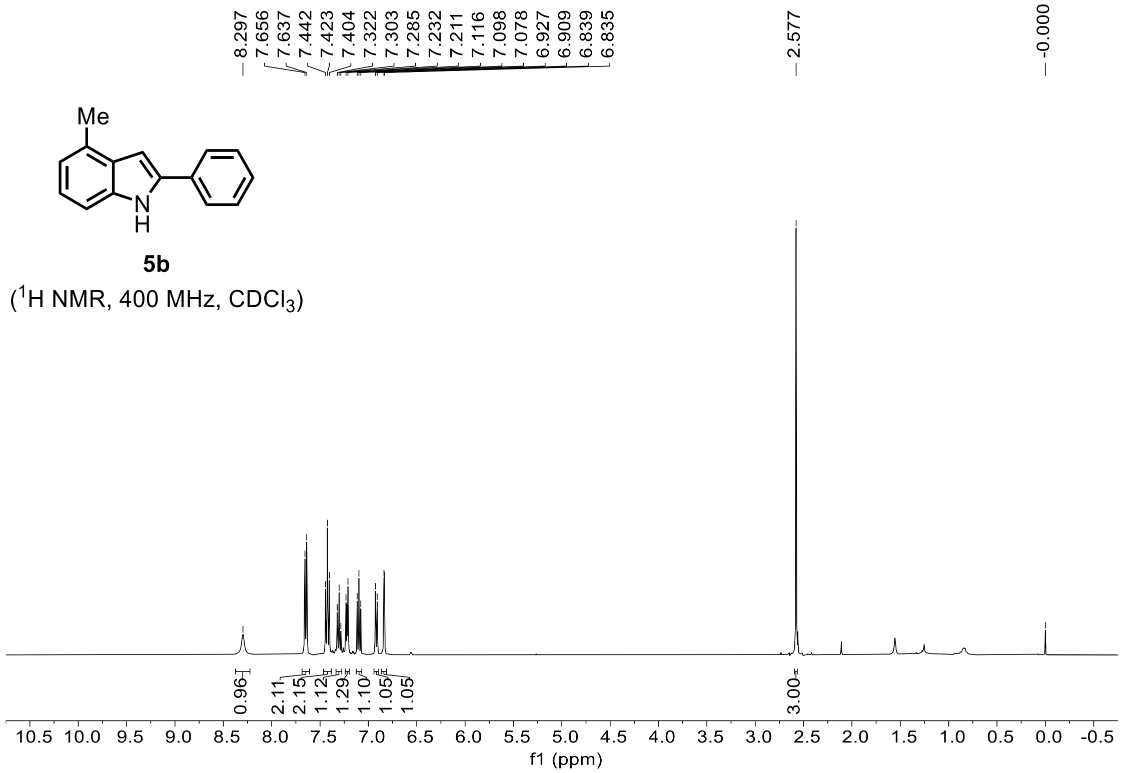

5b

(^{1}H NMR, 400 MHz, $CDCl_3$)

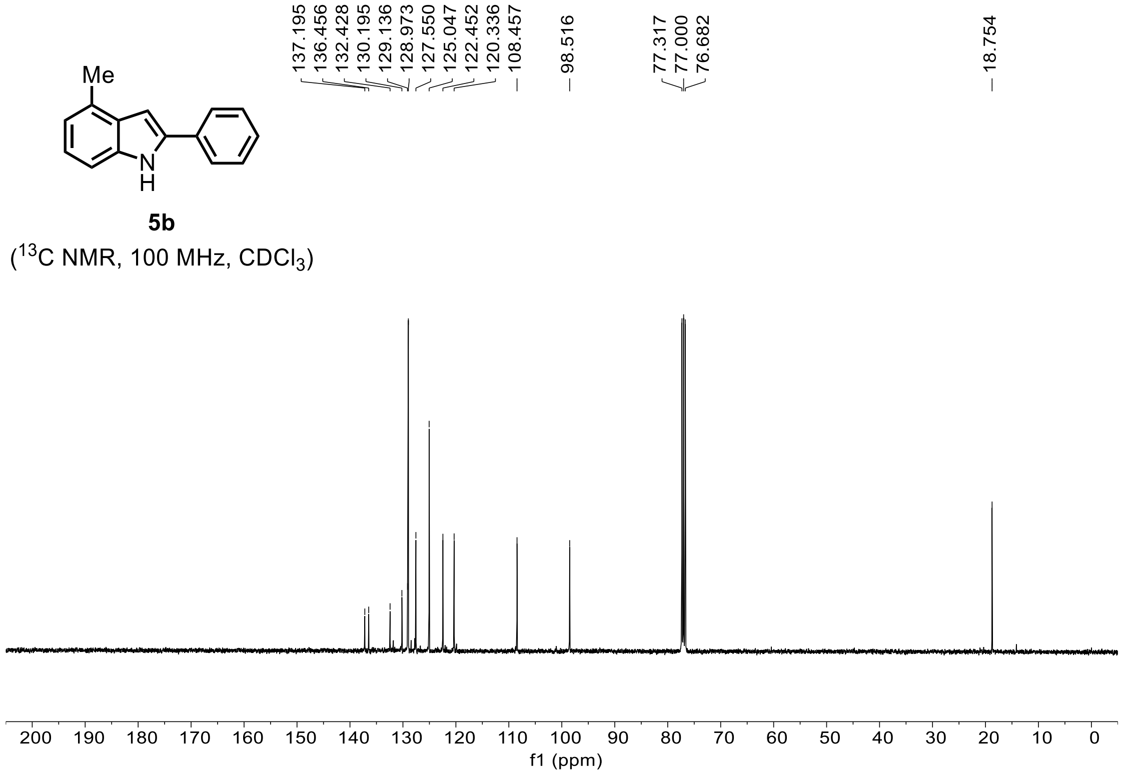

5b

(^{13}C NMR, 100 MHz, $CDCl_3$)

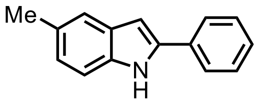

5c

(^{1}H NMR, 400 MHz, CDCl$_3$)

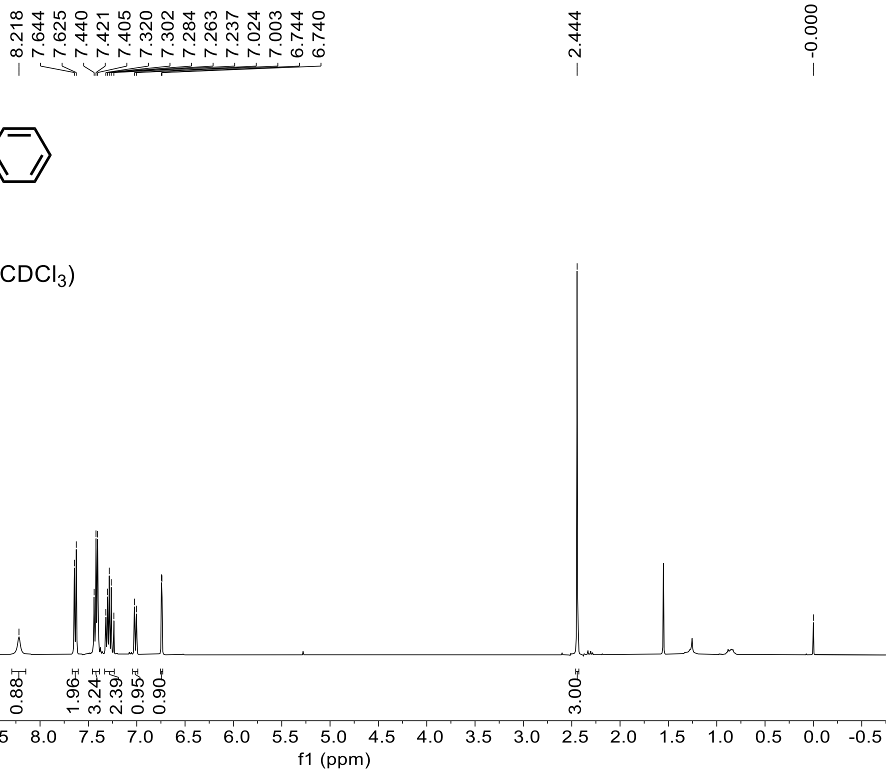

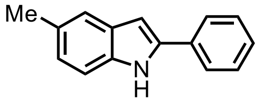

5c

(^{13}C NMR, 100 MHz, CDCl$_3$)

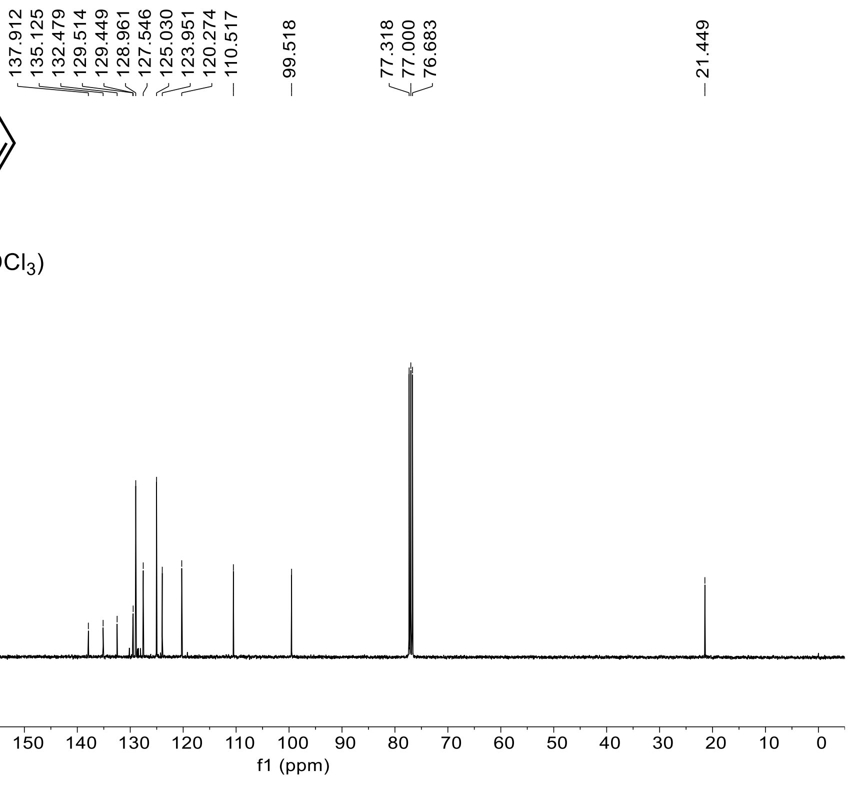

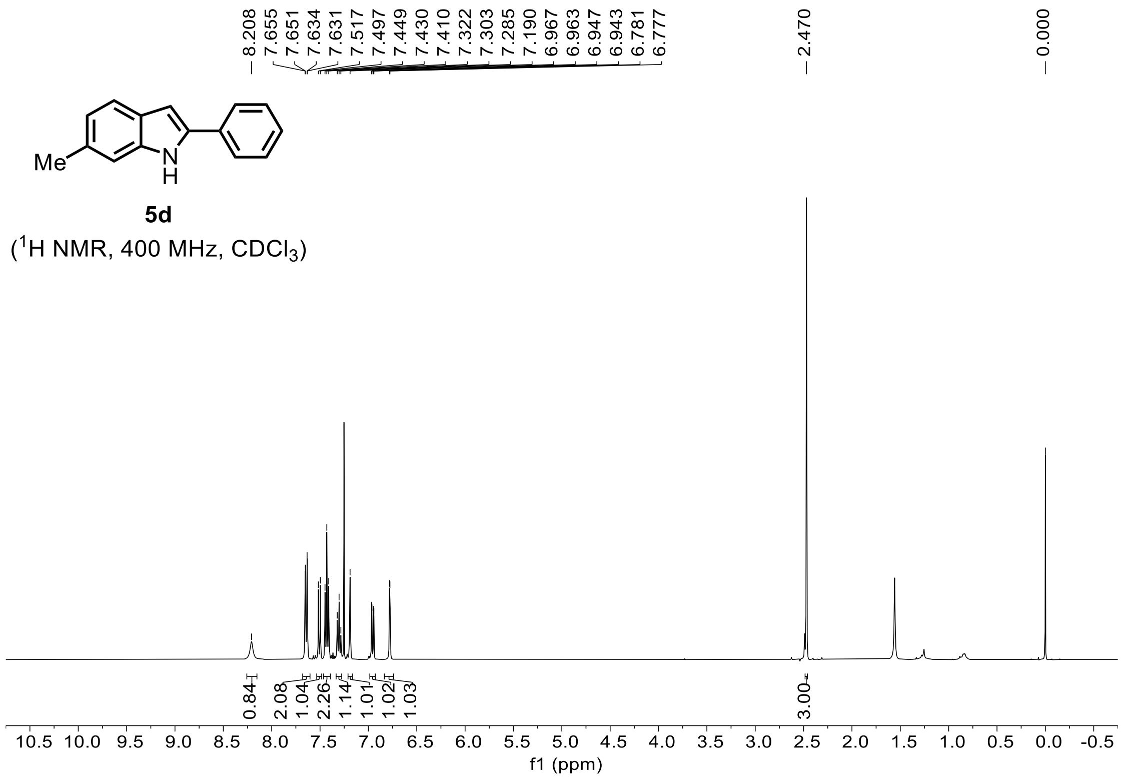

5d

(^{1}H NMR, 400 MHz, $CDCl_3$)

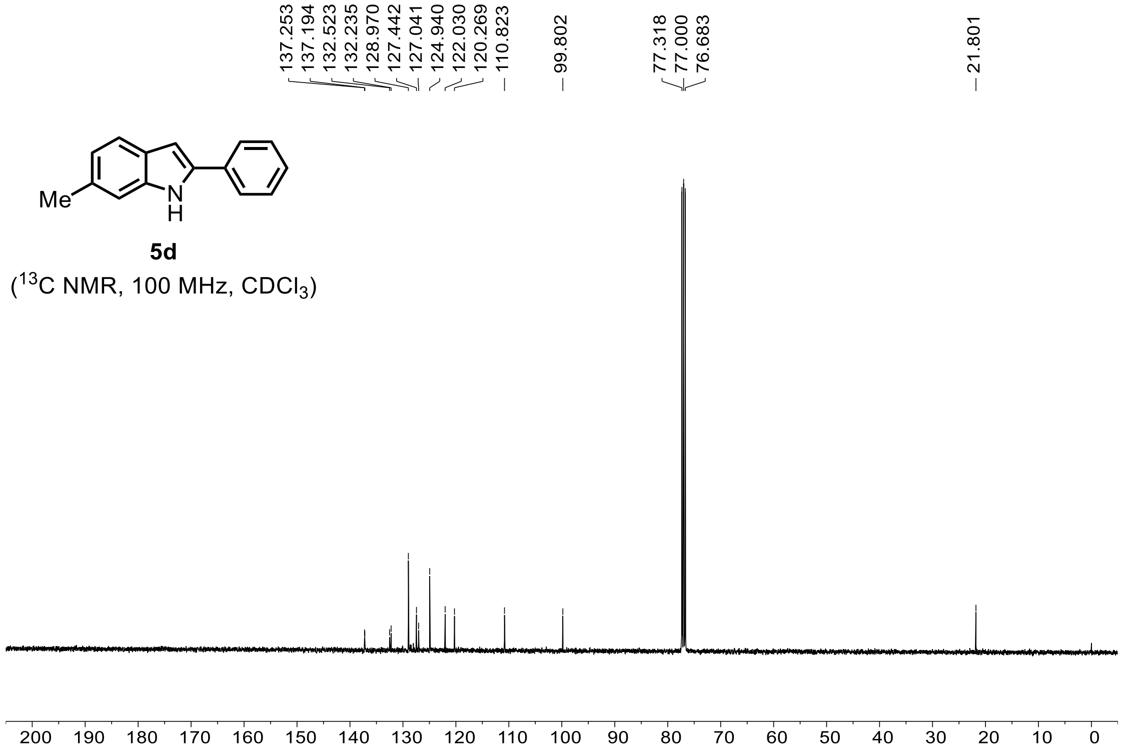

5d

(^{13}C NMR, 100 MHz, $CDCl_3$)

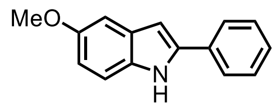

5e

(^{1}H NMR, 500 MHz, $CDCl_3$)

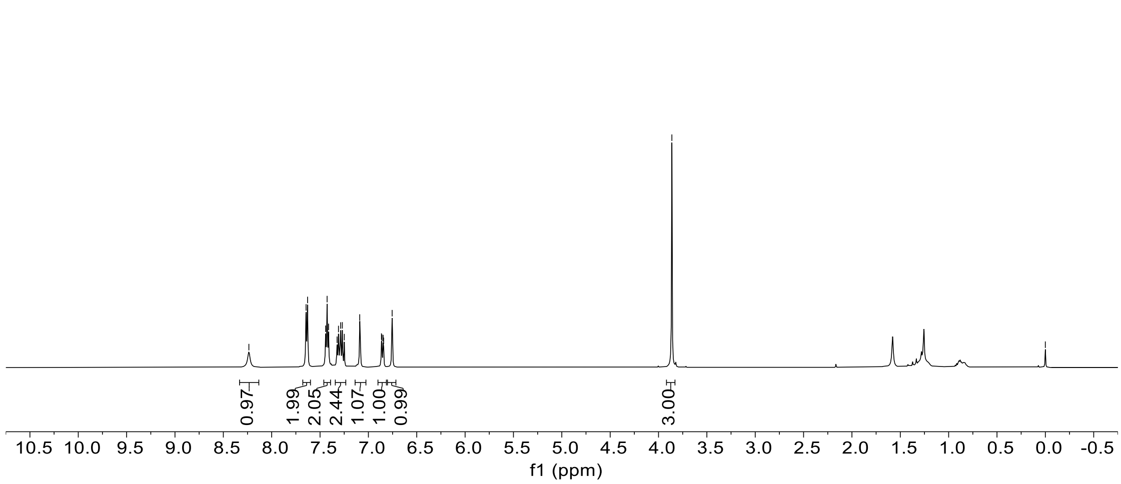

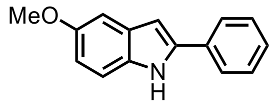

5e

(^{13}C NMR, 125 MHz, $CDCl_3$)

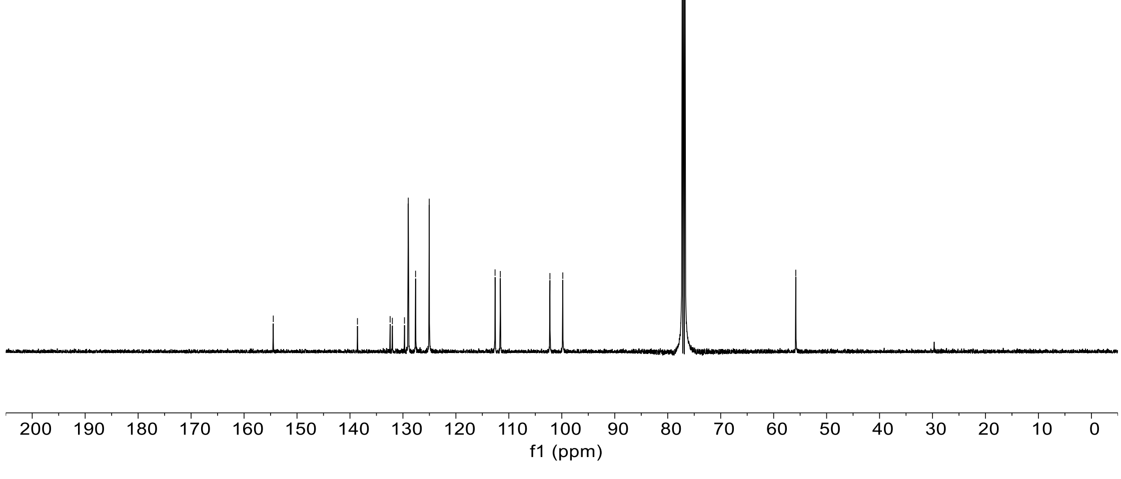

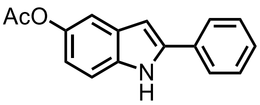

5f

(^{1}H NMR, 400 MHz, $CDCl_3$)

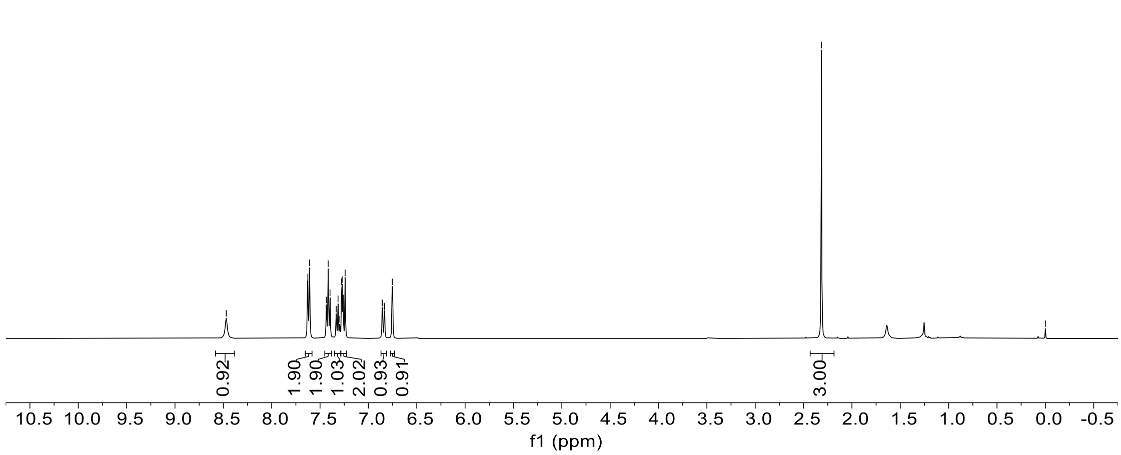

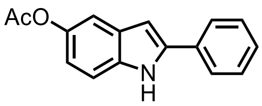

5f

(^{13}C NMR, 100 MHz, $CDCl_3$)

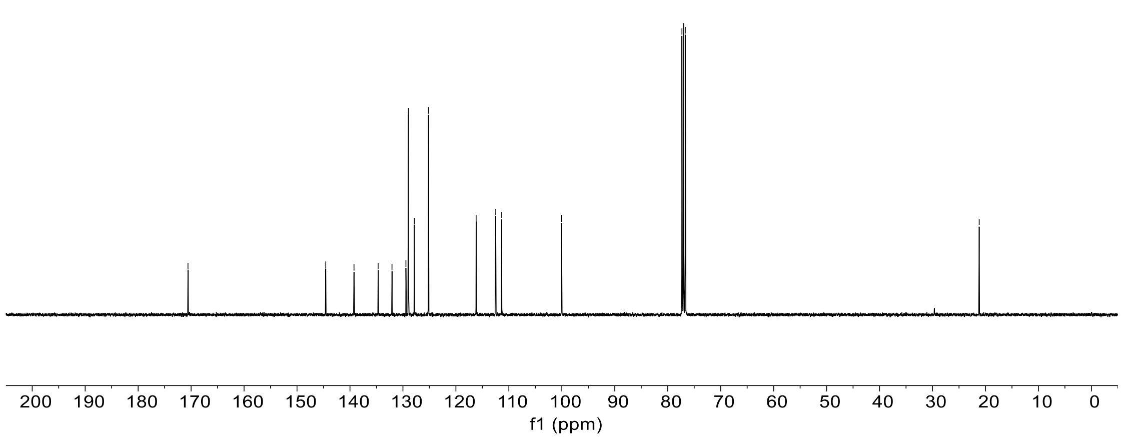

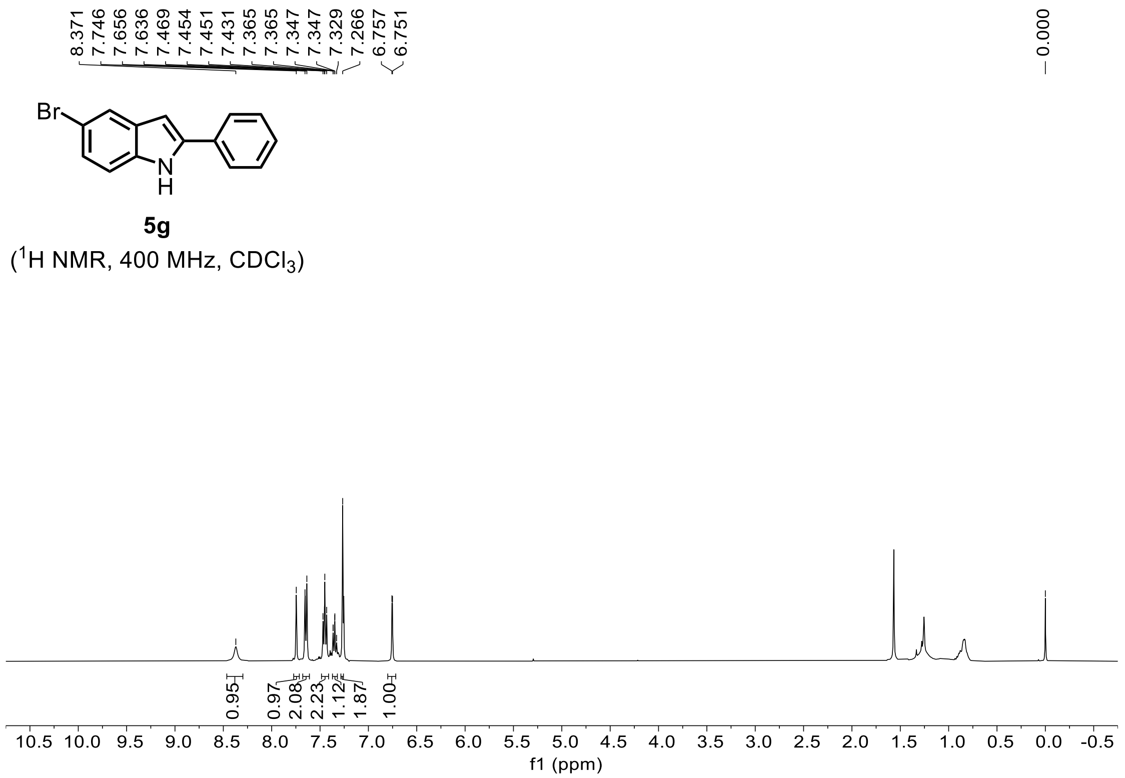

5g

(^{1}H NMR, 400 MHz, $CDCl_3$)

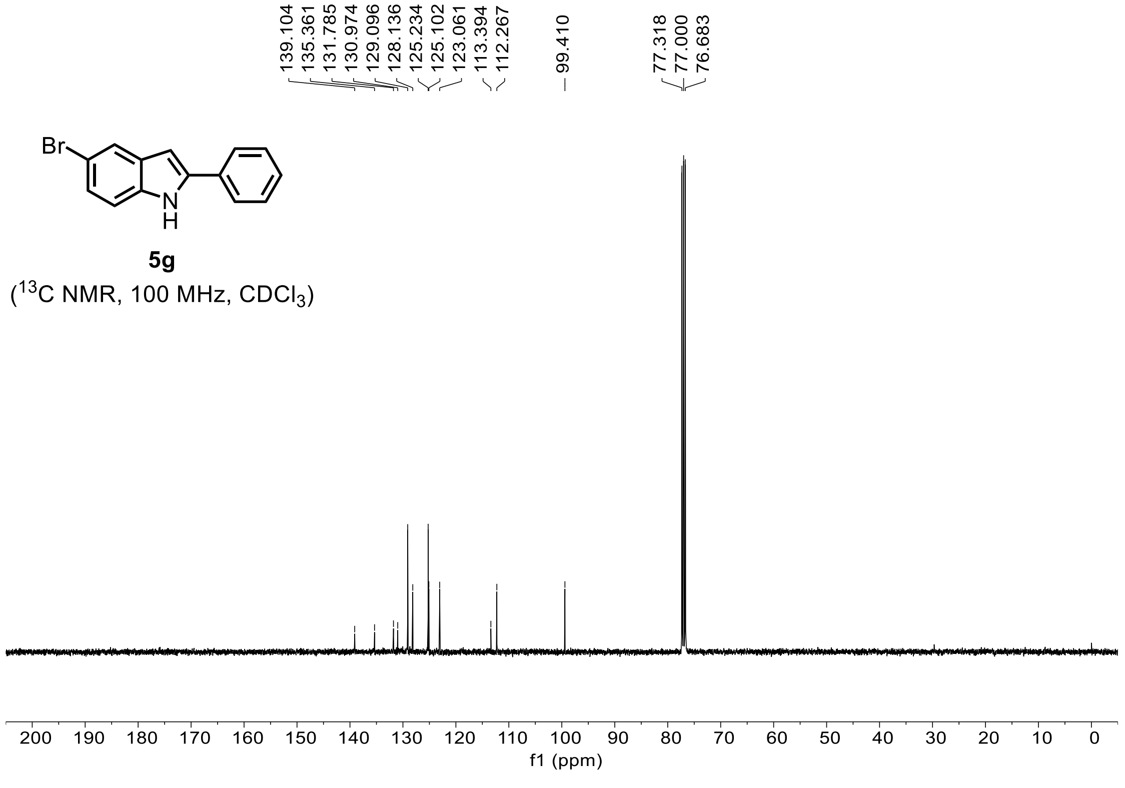

5g

(^{13}C NMR, 100 MHz, $CDCl_3$)

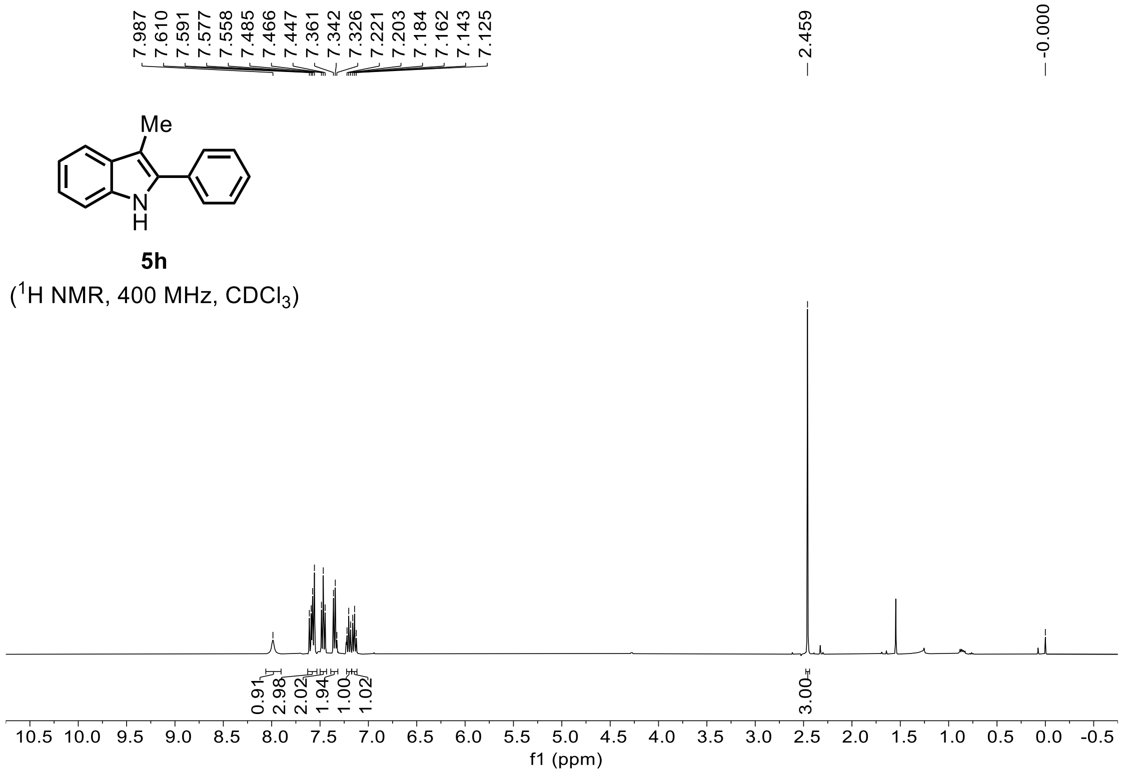
7.987
7.610
7.591
7.577
7.558
7.485
7.466
7.447
7.361
7.342
7.326
7.221
7.203
7.184
7.162
7.143
7.125
2.459
-0.000
Me
N
H
5h
(¹H NMR, 400 MHz, CDCl₃)
0.91
2.98
2.02
1.94
1.00
1.02
3.00
f1 (ppm)

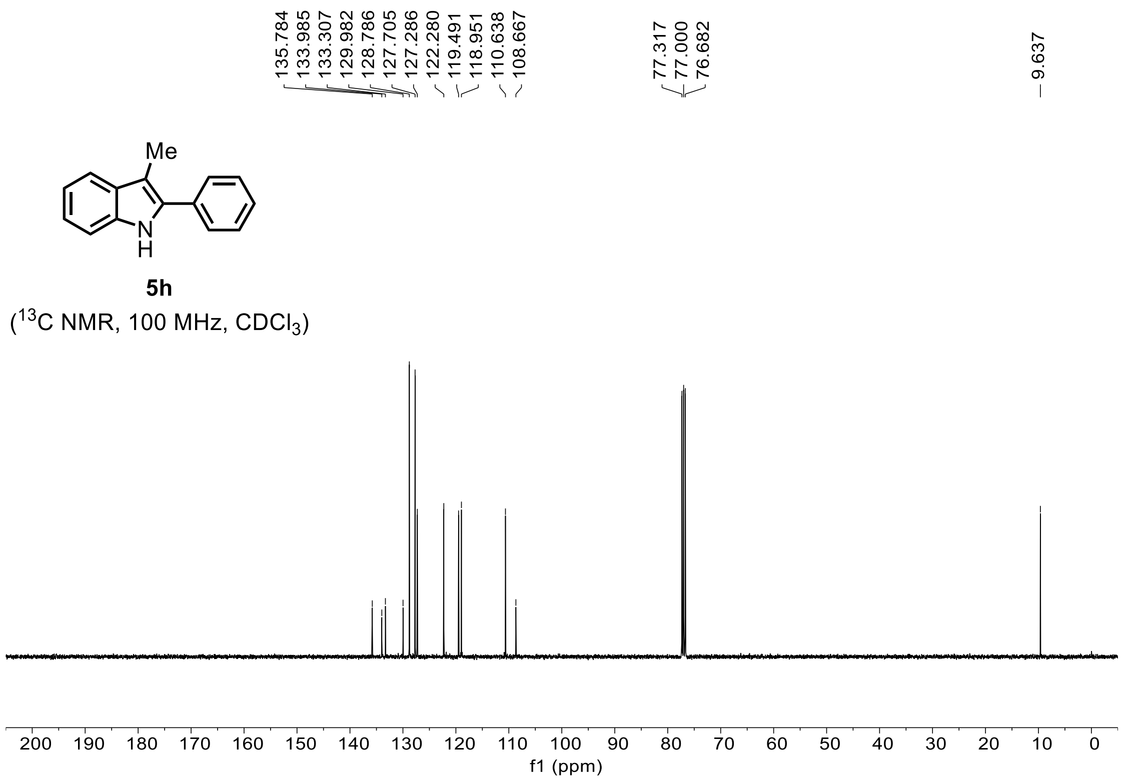
135.784
133.985
133.307
129.982
128.786
127.705
127.286
122.280
119.491
118.951
110.638
108.667
77.317
77.000
76.682
9.637
Me
N
H
5h
(¹³C NMR, 100 MHz, CDCl₃)
f1 (ppm)

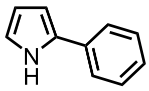

7a
(^{1}H NMR, 400 MHz, $CDCl_3$)

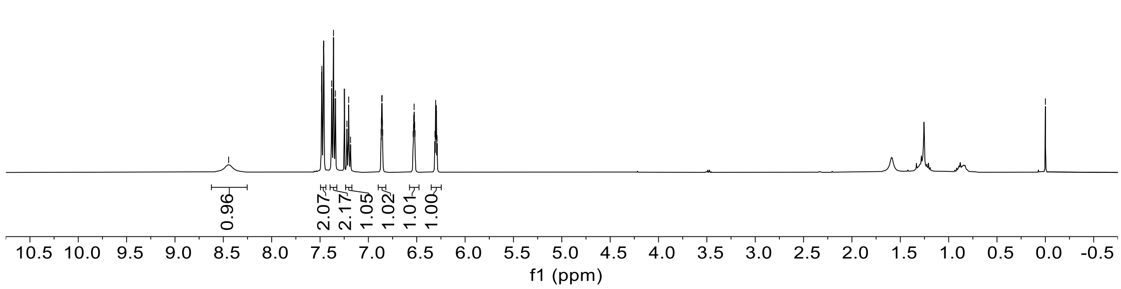

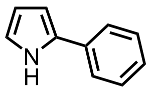

7a
(^{13}C NMR, 100 MHz, $CDCl_3$)

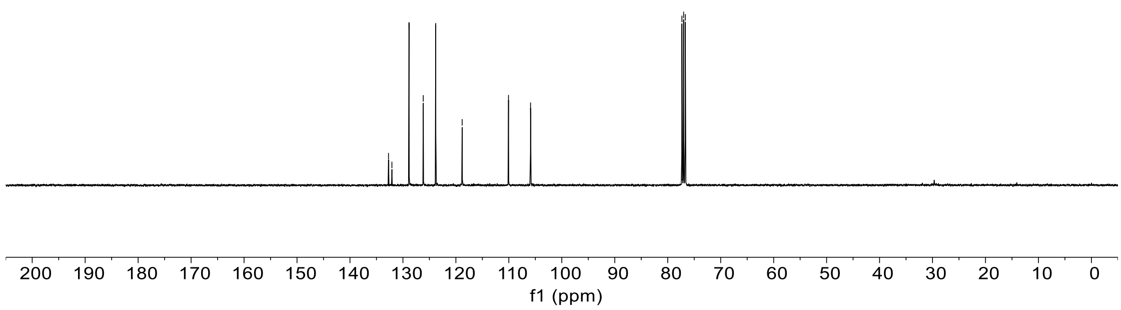

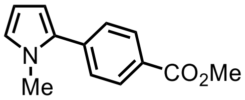

7b

(^{1}H NMR, 400 MHz, $CDCl_3$)

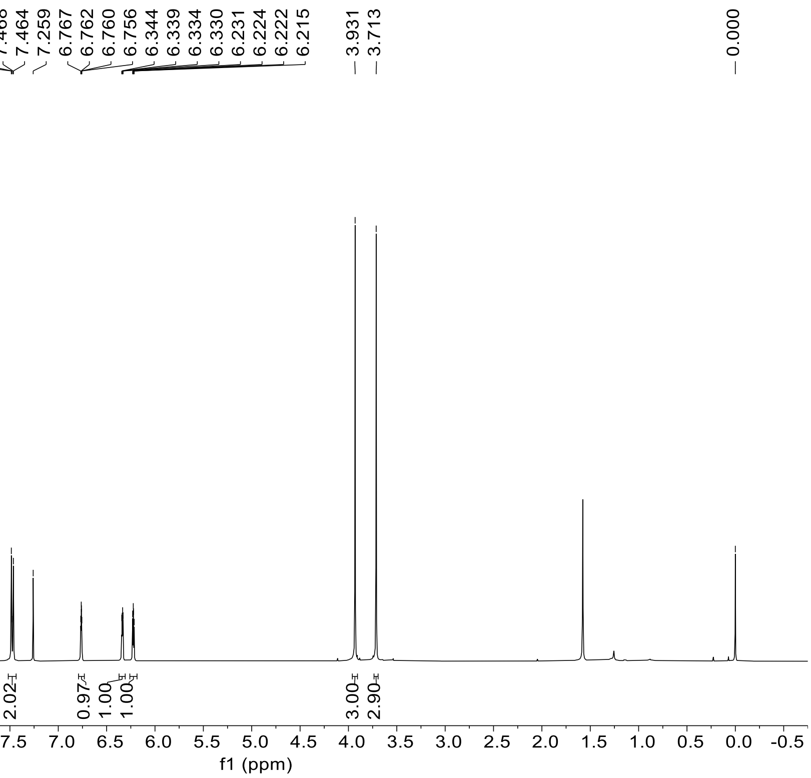

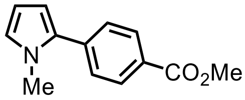

7b

(^{13}C NMR, 100 MHz, $CDCl_3$)

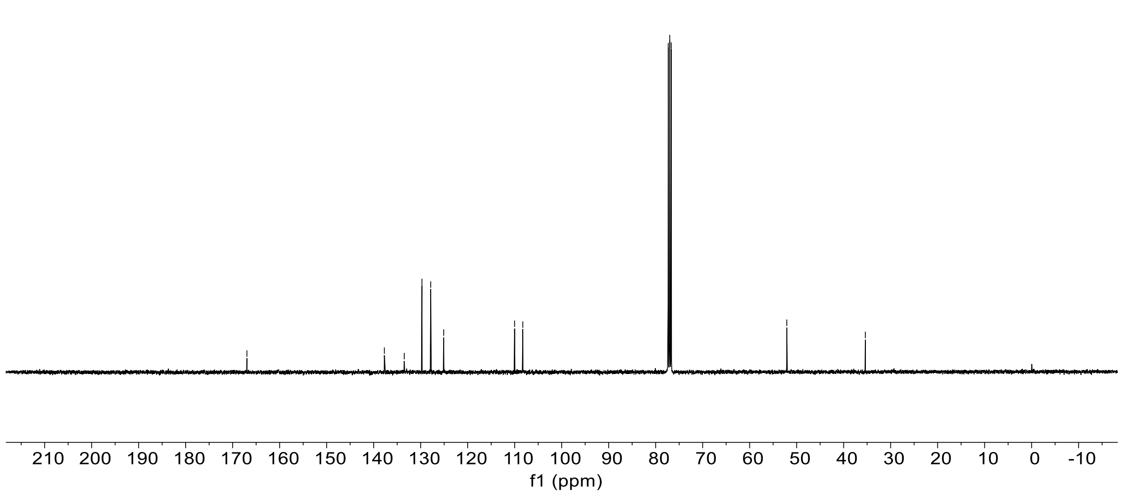

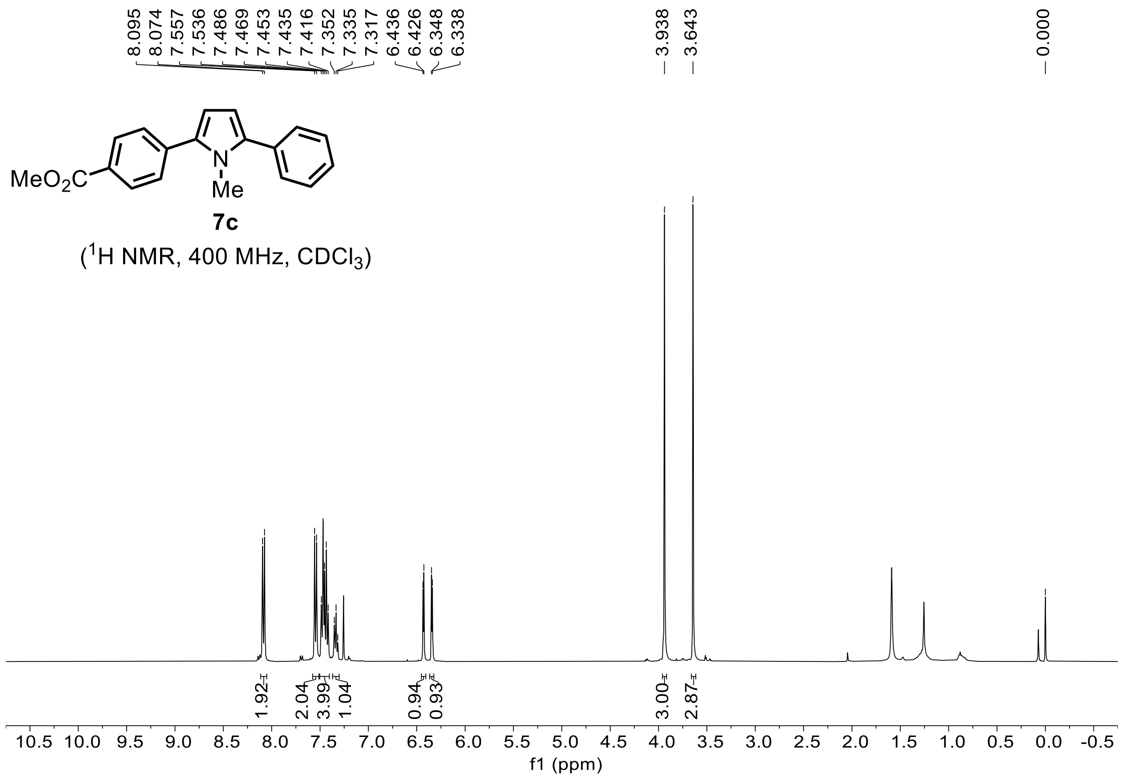
8.095
8.074
7.557
7.536
7.486
7.469
7.453
7.435
7.416
7.352
7.335
7.317
6.436
6.426
6.348
6.338
3.938
3.643
0.000
MeO2C
N
Me
7c
(1H NMR, 400 MHz, CDCl3)
1.92
2.04
3.99
1.04
0.94
0.93
3.00
2.87
10.5 10.0 9.5 9.0 8.5 8.0 7.5 7.0 6.5 6.0 5.5 5.0 4.5 4.0 3.5 3.0 2.5 2.0 1.5 1.0 0.5 0.0 -0.5
f1 (ppm)

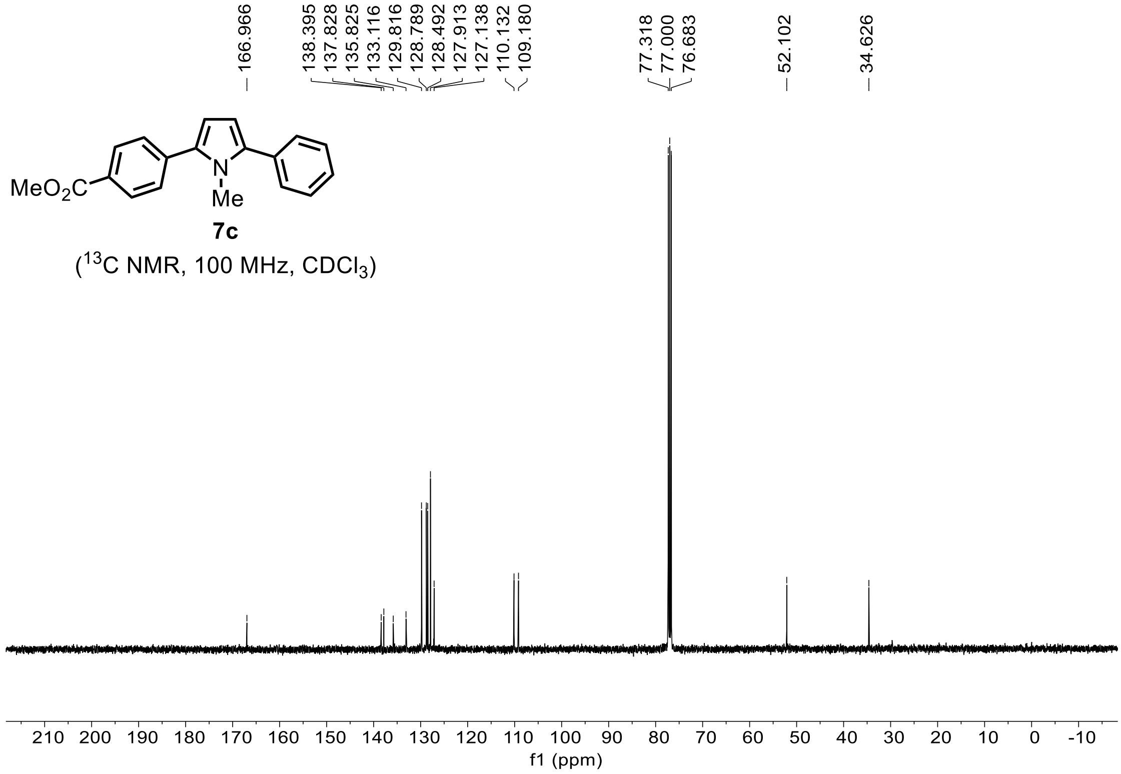
166.966
138.395
137.828
135.825
133.116
129.816
128.789
128.492
127.913
127.138
110.132
109.180
77.318
77.000
76.683
52.102
34.626
MeO2C
N
Me
7c
(13C NMR, 100 MHz, CDCl3)
210 200 190 180 170 160 150 140 130 120 110 100 90 80 70 60 50 40 30 20 10 0 -10
f1 (ppm)

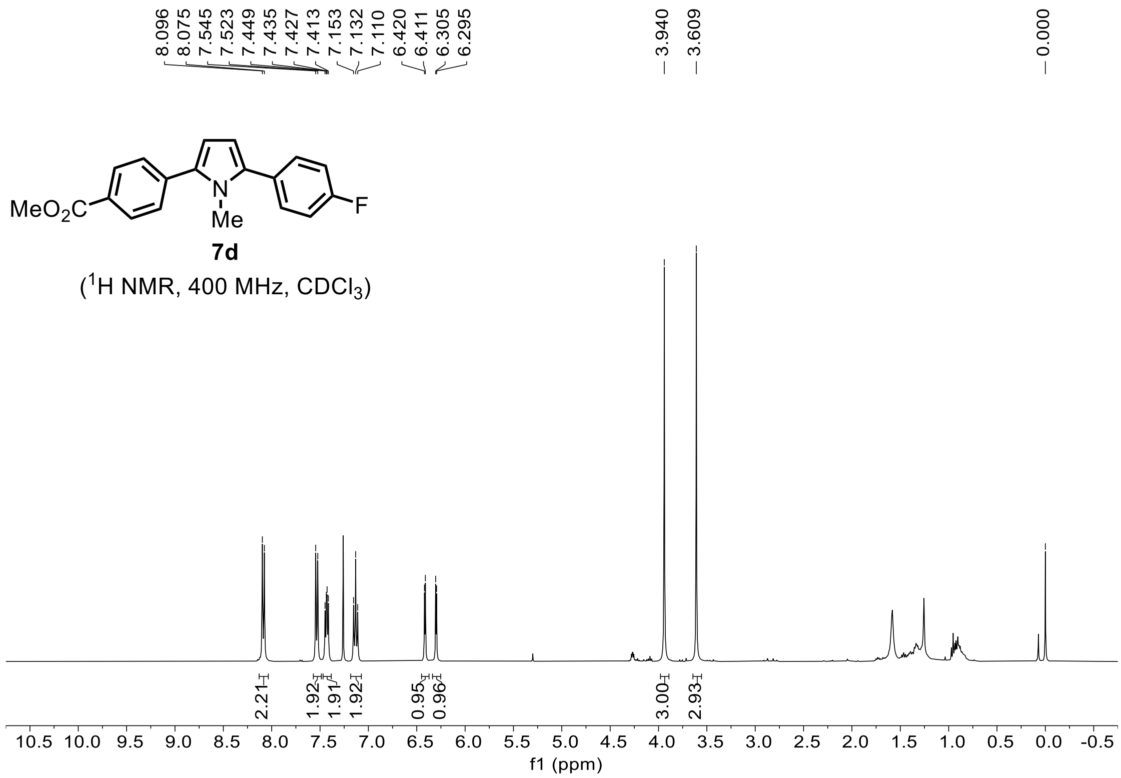
8.096
8.075
7.545
7.523
7.449
7.435
7.427
7.413
7.153
7.132
7.110
6.420
6.411
6.305
6.295
3.940
3.609
0.000
MeO2C
N
Me
F
7d
(1H NMR, 400 MHz, CDCl3)
2.21
1.92
1.91
1.92
0.95
0.96
3.00
2.93
10.5 10.0 9.5 9.0 8.5 8.0 7.5 7.0 6.5 6.0 5.5 5.0 4.5 4.0 3.5 3.0 2.5 2.0 1.5 1.0 0.5 0.0 -0.5
f1 (ppm)

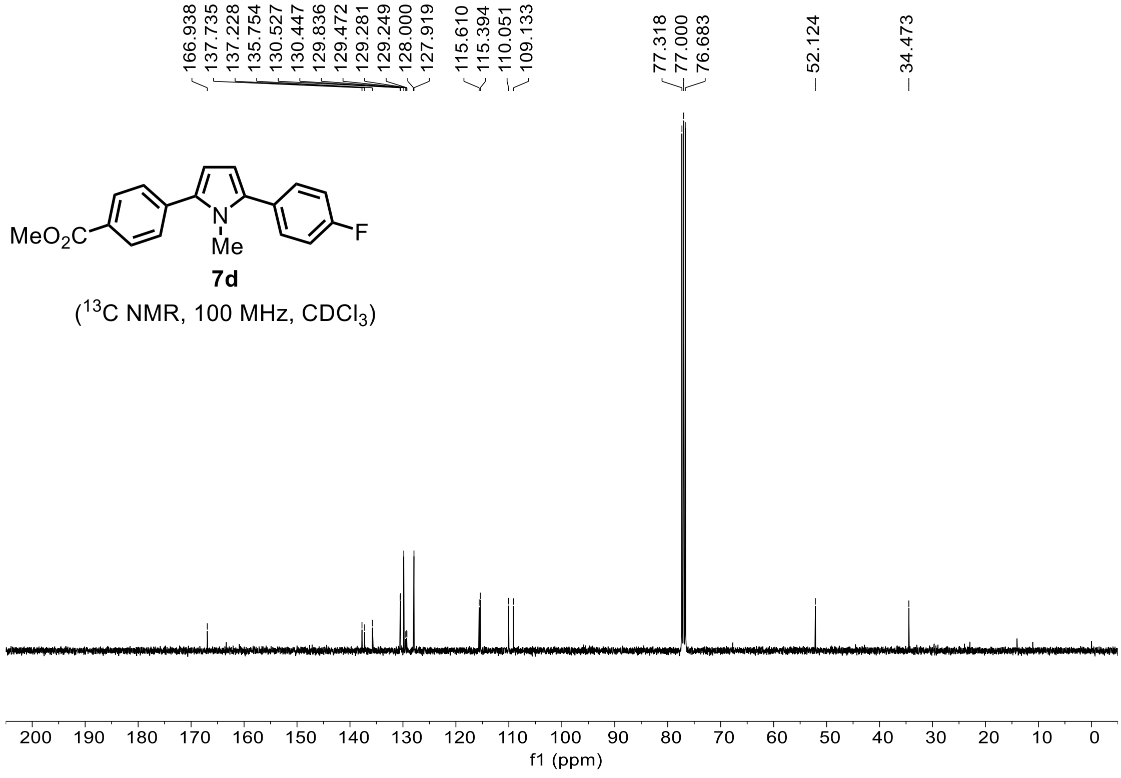
166.938
137.735
137.228
135.754
130.527
130.447
129.836
129.472
129.281
129.249
128.000
127.919
115.610
115.394
110.051
109.133
77.318
77.000
76.683
52.124
34.473
MeO2C
N
Me
F
7d
(13C NMR, 100 MHz, CDCl3)
200 190 180 170 160 150 140 130 120 110 100 90 80 70 60 50 40 30 20 10 0
f1 (ppm)

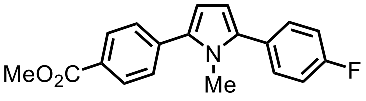

7d

(^{19}F NMR, 470 MHz, $CDCl_3$)

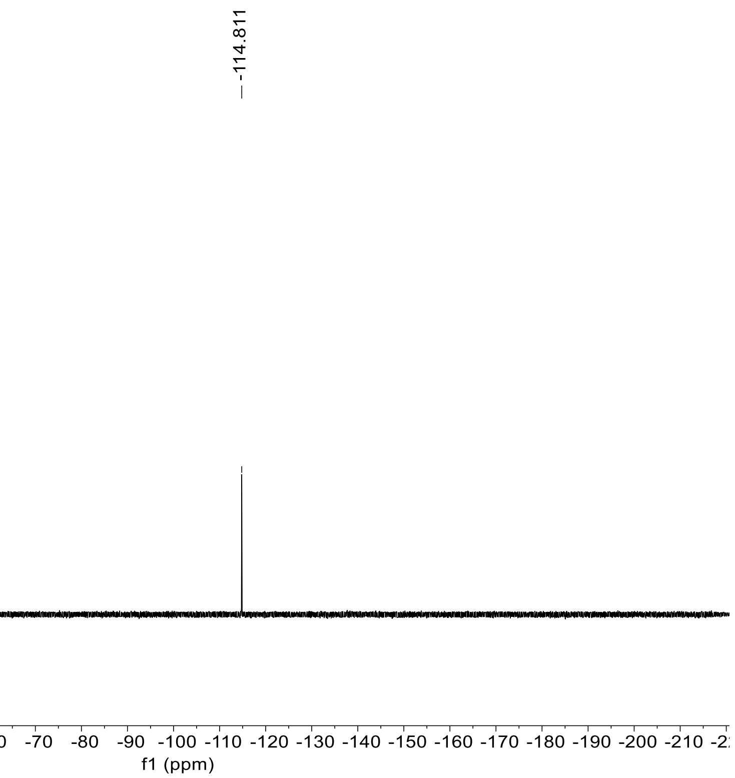

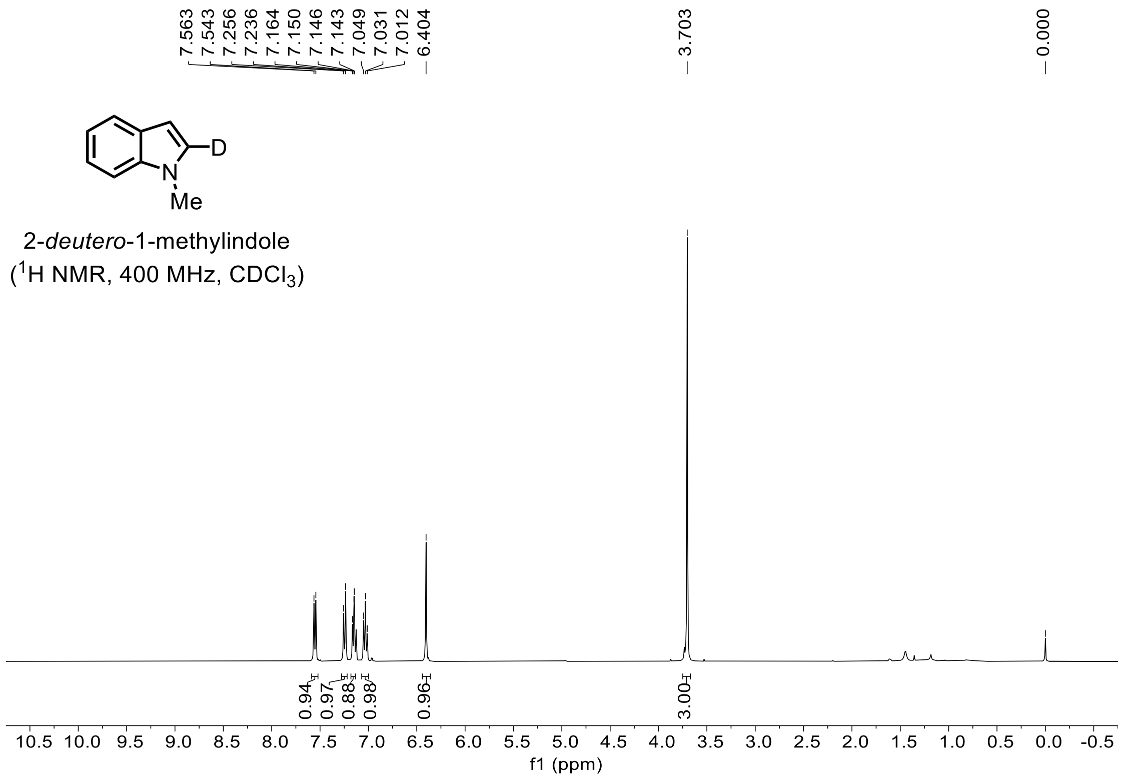
7.563
7.543
7.256
7.236
7.164
7.150
7.146
7.143
7.049
7.031
7.012
6.404
3.703
0.000
D
N
Me
2-*deutero*-1-methylindole
(^{1}H NMR, 400 MHz, $CDCl_3$)
0.94
0.97
0.88
0.98
0.96
3.00
10.5 10.0 9.5 9.0 8.5 8.0 7.5 7.0 6.5 6.0 5.5 5.0 4.5 4.0 3.5 3.0 2.5 2.0 1.5 1.0 0.5 0.0 -0.5
f1 (ppm)

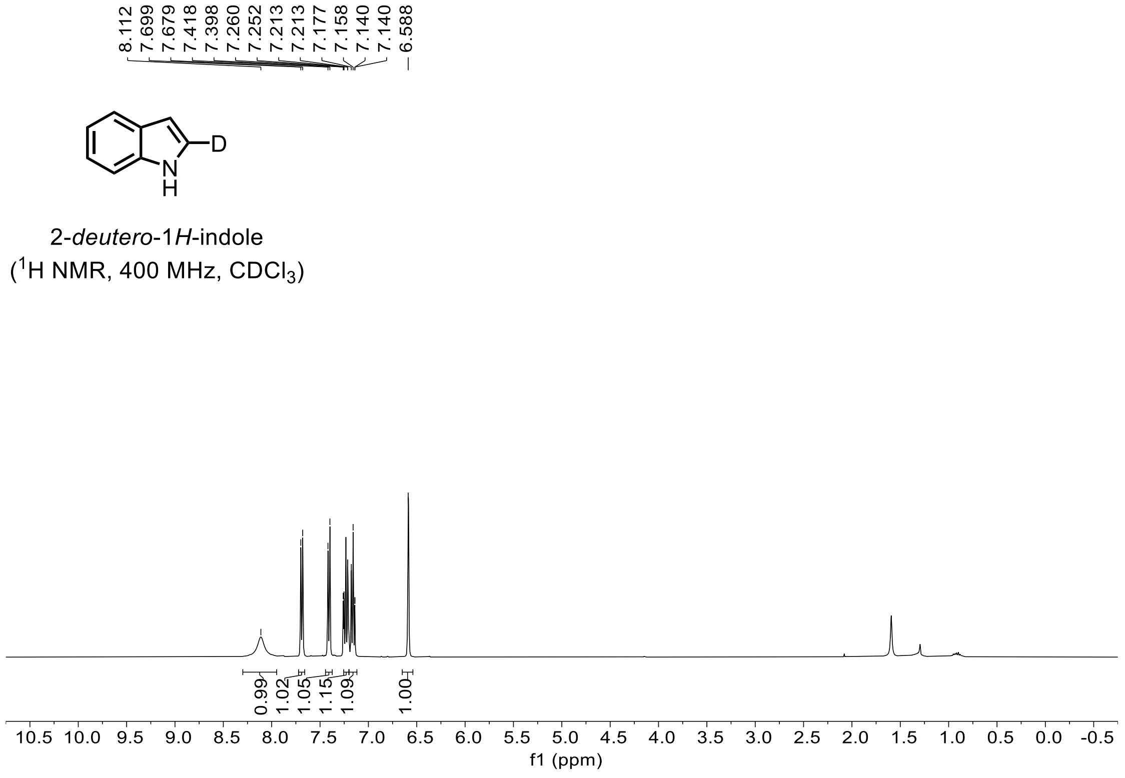
8.112
7.699
7.679
7.418
7.398
7.260
7.252
7.213
7.213
7.177
7.158
7.140
7.140
6.588
D
N
H
2-*deutero*-1*H*-indole
(^{1}H NMR, 400 MHz, $CDCl_3$)
0.99
1.02
1.05
1.15
1.09
1.00
10.5 10.0 9.5 9.0 8.5 8.0 7.5 7.0 6.5 6.0 5.5 5.0 4.5 4.0 3.5 3.0 2.5 2.0 1.5 1.0 0.5 0.0 -0.5
f1 (ppm)
