# Supplementary material for: Structural Reassignment of Covalent Organic Framework-Supported Palladium Species: Heterogenized Palladacycles as Efficient Catalysts for Sustainable C–H Activation
Source: ACS Cent Sci. 2024 Aug 28;10(10):1848–60. doi: 10.1021/acscentsci.4c00660 (PMC11503496; doi:10.1021/acscentsci.4c00660)
Supplement: Supplementary file 2 — oc4c00660_si_002.pdf [file oc4c00660_si_002.pdf]

oc-2024-006604.R1

Name: Peer Review Information for "Structural Reassignment of Covalent Organic Framework-Supported Palladium Species: Heterogenized Palladacycles as Efficient Catalysts for Sustainable C–H Activation"

First Round of Reviewer Comments

Reviewer: 1

Comments to the Author
The authors prepared quinolone and imine-linked COF materials as solid supports to prepare heterogeneous palladium catalysts including a palladacycle with a 2- arylquinoline moiety on the COF for the C–H arylation of indole and pyrrole derivatives. The manuscript is interesting and characterizations are done with due competence. I feel this suitable for publication in ACS Central Science after considering following revision.

1) The optimised reaction condition for C–H arylation of N-methyl indole with diphenyliodonium tetrafluoroborate should be carried out more elaborately considering temperature, duration and loading of Pd@Quin-COF-1. Did the authors test possibility of RT reaction, or the same within shorter time span?
2) The statement “Pd@Quin-COF-2 exhibited poor stability and reactivity after the first run of catalysis (Table 1, entry 2), possibly due to the collapse of large pores in the COF supported palladium catalyst” needs rational substantiation at the interface of design principle.
3) The authors anchored Pd(II) to the linker of COF, and the resulting catalyst shows C–H Arylation performance. Seeing these results in perspective, one wonders why the COF does not participate in one of its interesting function such as size/shape selectivity?
4) Given C–H arylation in COF is an important area, some relevant studies can be cited in the main text. For example: 10.1021/acsami.0c14678, 10.1016/j.apcatb.2021.120149, 10.1021/jacs.2c01814.

Reviewer: 2

Comments to the Author
This submission describes the synthesis of palladium-containing covalent organic frameworks, their characterization and application in catalysis. It builds on carbopalladation reactions of phenyl pyridine with palladium acetate to form acetate-bridged dimeric palladacycles, which was extensively studied from an organic synthesis perspective. The application in the design of COF materials is in my opinion an innovative approach in accessing novel catalytically active materials. However, this reviewer is not an expert in COF materials and therefore my review will more focus on the material characterization and synthesis application.

-The title of this submission suggests a structural reassignment – as organic chemist, I was expecting the reassignment of previously synthesized COF materials, where structural misassignment was made (not judging why it was made). However, in the manuscript text, I did not perceive any previous structurally misassigned COF materials, instead I got the perception that the authors analyzed a series of novel COF materials, which were then evaluated in catalysis. One of these materials, where no imine bond was present, but a quinoline instead, proved catalytically most promising. This should be revisited by the authors and put into context. What led to the term “reassignment”?

-Figure 6d suggests that the catalytic efficiency of Pd(OAc)2 is significantly reduced after 2-3 hours reaction time. How does this compare to the originally reported arylation of 2-methyl indole by Sanford (Ref. 22), which is reported to give good yields even after 5 min reaction time? The COF material requires 48 hours reaction time instead. This should be discussed by the authors. Sanford and co-workers were using acetic acid as additive in their reaction, while in the present submission, acetic acid is not required. Can the authors comment on this?

-The 2-arylation reaction of indole is a very well established transformation and numerous methods exist for this purpose. Data on the reusability of the catalyst and its catalytic performance is convincing, yet the transformation is from an organic synthesis perspective not truly exciting. This is – from an organic chemists' perspective – an innovative new catalyst, the potential of it, is however not fully considered by the authors. I would have expected more in-depth studies, e.g. in other Pd-catalyzed transformations to show the generality of this COF material in a more general sense. At present, the synthesis component is in my opinion a weak part of this submission, which should be significantly improved, e.g. are there transformations exclusive to this COF material? At present, the combination of this new material with a rather modest advancement in catalysis/synthesis is more suitable for publication in a more specialized journal.

-The authors state that crystallinity remained constant after 5-runs as shown in Figure 6f. However, I perceive significant differences in the PXRD between the original material and the material after 5 runs. Can the authors explain these?

Reviewer: 3

Comments to the Author
The development of sustainable metal catalysts for value-added transformations via C-H activation is crucial to meet the increasing demands from academia and industry. A case in point is the work by Sun et al., which advances supported imine-containing palladacycle catalysts. These catalysts are constructed through the post-synthetic palladation of various imine-linked covalent organic framework (COF) materials and have been shown to be effective in the C-H arylation of a wide range of indole and pyrrole derivatives with diaryliodonium tetrafluoroborate at C2 sites. This work enhances the capabilities of molecular Pd species and porous crystalline materials, enabling their recyclability and improving their performance in C-H arylation of heterocyclic substrates with diverse electronic properties. This advancement justifies its acceptance for publication in ACS Central Science. However, revisions are needed to address the following concerns:
1. The significance of catalytic application is not clearly defined due to insufficient background information and the lack of comparison. Therefore, a detailed introduction is necessary to elucidate the current status of C-H arylation of indole and pyrrole derivatives. Additionally, a table summarizing the performance indicators should be included to compare with existing homogeneous/heterogeneous catalysts.
2. Full-scale XPS spectra should be provided following standard discipline.
3. The study on diverse porous supports lacks conviction without providing any characterization of them. Basic measurements, such as PXRD patterns of different Pd(II)-incorporated COF materials, are suggested to be included.
4. There appears to be evidence of structural collapse during post-modification, as indicated by the drastic decrease in BET surface area and the emergence of a pore size at around 5 nm. This point requires further explanation.

5. This work focuses on sp2 C-H activation, while sp3 C-H activation is a more challenging task. It would be valuable to know whether the newly established catalysts are active toward sp3 C-H activation. Besides, the performance of COF materials incorporated with other molecular Pd species, aside from Pd(OAc)2, should be investigated further.

Author's Response to Peer Review Comments:

THE UNIVERSITY OF HONG KONG

Department of Chemistry and State Key Laboratory of Synthetic Chemistry
Room 103, Hui Oi Chow Science Building, Pokfulam Road, Hong Kong

Jian He, Ph.D.
Assistant Professor
Email: jianhe@hku.hk
Phone: (852) 3910 2193
Fax: (852) 2857 1586

June 24, 2024

We sincerely appreciate the reviewers' time and efforts in helping us improve the quality of our work. We conducted additional experiments and revised the manuscript in response to their constructive feedback and suggestions.

Reviewer #1

The authors prepared quinolone and imine-linked COF materials as solid supports to prepare heterogeneous palladium catalysts including a palladacycle with a 2-arylquinoline moiety on the COF for the C–H arylation of indole and pyrrole derivatives. The manuscript is interesting and characterizations are done with due competence. I feel this suitable for publication in ACS Central Science after considering following revision.

(1) The optimised reaction condition for C–H arylation of N-methyl indole with diphenyliodonium tetrafluoroborate should be carried out more elaborately considering temperature, duration and loading of Pd@Quin-COF-1. Did the authors test possibility of RT reaction, or the same within shorter time span?

Response: We thank the reviewer for the very important concerns. A more detailed reaction screening table was included in the updated SI.

"After systematic survey of various reaction parameters (Tables S5–S7), we were delighted to discover that *N*-methyl 2-phenylindole (**3a**) could be obtained in 97% yield with only 2 mol% of **Pd@Quin-COF-1** (Table 1, entry 1)."

Table S7. Optimization of the C2-Selective C–H Arylation using Pd@Quin-COF-1 as the Heterogeneous Catalyst

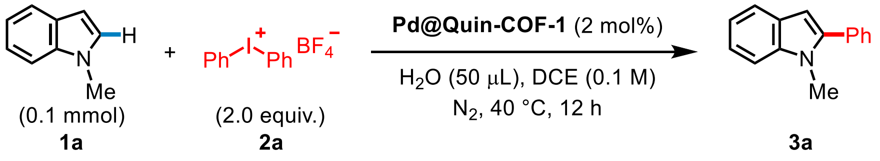

Entry	Change from "Standard Conditions"	Yield of **3a** (%)[a]
1	none	97
2	r.t., instead of 40 °C	74
3	6 h	79
4	2 h	43
5	1 h	33
6	**Pd@Quin-COF-1** (1 mol%)	59
7	**2a** (1.5 equiv.)	69
8	**2a** (1.0 equiv.)	55
9	AcOH (50 μL), instead of H_2O	66
10	under an atmosphere of air, instead of N_2	76

[a]Yield was determined by ^1H NMR of the crude product using CH_2Br_2 as an internal standard.

(2) The statement "Pd@Quin-COF-2 exhibited poor stability and reactivity after the first run of catalysis (Table 1, entry 2), possibly due to the collapse of large pores in the COF supported palladium catalyst" needs rational substantiation at the interface of design principle.

Response: We thank the reviewer for the important suggestion and revised the discussion in the manuscript as stated below:

"On the contrary, **Pd@Quin-COF-2** exhibited drastically poor reactivity after the first run of catalysis (Table 1, entry 2). Given that the crystallinity of **Quin-COF-2**, which had a large pore around 2.8 nm,[13a] was completely lost during the post-synthetic metalation step (Figure S53), the COF support could no longer stabilize palladacycle intermediates in the arylation reaction."

"The interior performance of **Pd@Im-COF-6**, **Pd@Im-COF-7**, and **Pd@Im-COF-8** is attributed to the instability of the imine-linked framework supports under the standard reaction conditions (Figure S50). In the recycling experiments, all of these heterogeneous palladium catalysts with large COF pores demonstrated a similar reactivity trend to **Pd@Quin-COF-2** (entry 2)."

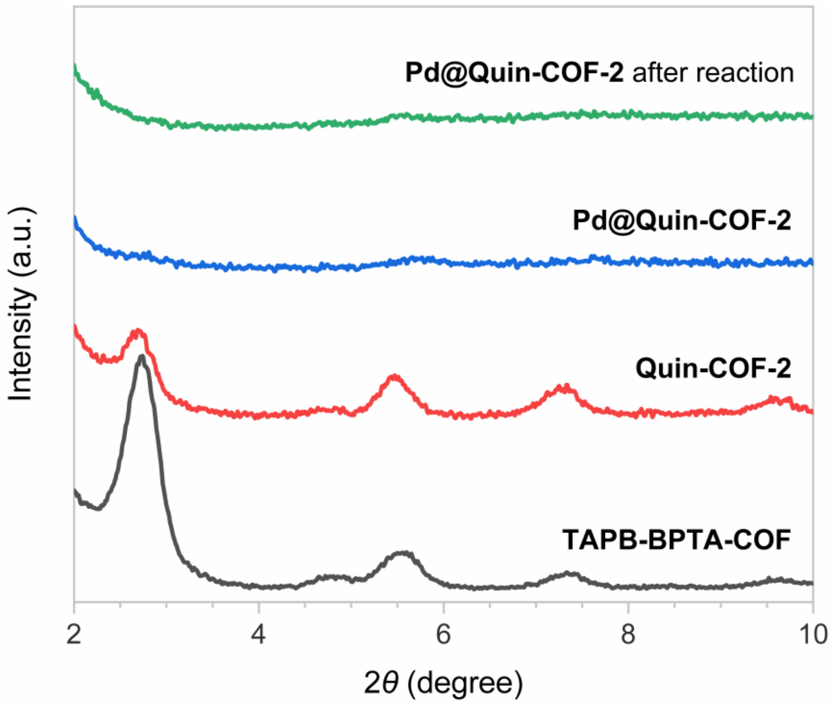

Figure S53. PXRD patterns of COF materials related to **Pd@Quin-COF-2**.

(3) The authors anchored Pd(II) to the linker of COF, and the resulting catalyst shows C–H Arylation performance. Seeing these results in perspective, one wonders why the COF does not participate in one of its interesting function such as size/shape selectivity?

Response: We thank the reviewer for the important concern. While we did not observe a significant change in reactivity when introducing various substituents into the six-membered ring, the addition of functional groups at the 3-position of indole substrates led to a decrease in reaction efficiency. Therefore, we updated the related description in the manuscript as follows:

"Upon slight modification of water loadings (Table S10), a variety of the indole substrates could be arylated in good yields (Table 3). In contrast to homogeneous catalytic systems, the arylation reaction is more susceptible to the steric hindrance at the 3-position of indoles (**5h**). When the methyl group was replaced with a larger substituent in *N*-acetyl-tryptophan methyl ester, the heterogeneous palladium catalysis became less effective, revealing size selectivity in COF-based reaction systems (Table S11)."

Table S11. Investigation of Size Selectivity in COF-Based Palladium Catalysis

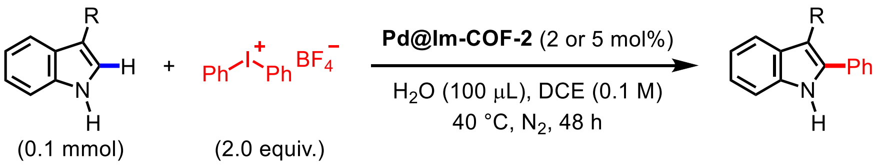

Entry	Substrate	Catalyst (mol%)	Product	Yield (%)[a]
1	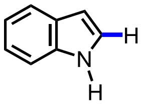	2	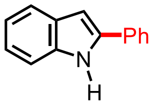	67
2	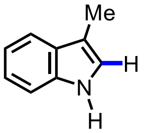	5	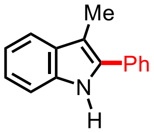	49
3	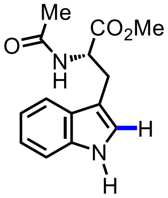	5	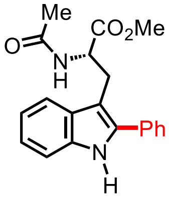	29

[a]Yield was determined by ^{1}H NMR of the crude product using CH_2Br_2 as an internal standard.

(4) Given C–H arylation in COF is an important area, some relevant studies can be cited in the main text. For example: 10.1021/acsami.0c14678, 10.1016/j.apcatb.2021.120149, 10.1021/jacs.2c01814.

Response: We thank the reviewer for the important suggestion and included the aforementioned references in Reference 8 of the manuscript.

(d) Kumar, G.; Singh, M.; Goswami, R.; Neogi, S. Structural Dynamism-Actuated Reversible CO_2 Adsorption Switch and Postmetalation-Induced Visible Light Cα−H Photocyanation with Rare Size Selectivity in N-Functionalized 3D Covalent Organic Framework. *ACS Appl. Mater. Interfaces* **2020**, *12*, 48642–48653. (e) Kumar, G.; Pillai, R. S.; Khan, N.-u. H.; Neogi, S. Structural Engineering in Pre-Functionalized, Imine-Based Covalent Organic Framework via Anchoring Active Ru(II)-Complex for Visible-Light Triggered and Aerobic Cross-Coupling of *α*-Amino Esters with Indoles. *Appl. Catal., B Environ.* **2021**, *292*, 120149. (f) Vardhan, H.; Al-Enizi, A. M.; Nafady, A.; Pan, Y.; Yang, Z.; Gutiérrez, H. R.; Han, X.; Ma, S. Single-Pore versus Dual-Pore Bipyridine-Based Covalent–Organic Frameworks: An Insight into the Heterogeneous Catalytic Activity for Selective C–H Functionalization. *Small* **2021**, *17*, 200397. (g) Salemi, H.; Debruyne, M.; Van Speybroeck, V.; Van Der Voort, P.; D'hooghe, M.; Stevens, C. V. Covalent Organic Framework Supported Palladium Catalysts. *J. Mater. Chem. A* **2022**, *10*, 20707–20729. (h) Jati, A.; Dey, K.; Nurhuda, M.; Addicoat, M. A.; Banerjee, R.; Maji, B. Dual Metalation in a Two-Dimensional Covalent Organic Framework for Photocatalytic C−N Cross-Coupling Reactions. *J. Am. Chem. Soc.* **2022**, *144*, 7822–7833.

Reviewer #2

(1) The title of this submission suggests a structural reassignment – as organic chemist, I was expecting the reassignment of previously synthesized COF materials, where structural misassignment was made (not judging why it was made). However, in the manuscript text, I did not perceive any previous structurally misassigned COF materials, instead I got the perception that the authors analyzed a series of novel COF materials, which were then evaluated in catalysis. One of these materials, where no imine bond was present, but a quinoline instead, proved catalytically most promising. This should be revisited by the authors and put into context. What led to the term "reassignment"?

Response: We sincerely thank the reviewer for pointing out his/her concerns about the structural reassignment. Following comprehensive characterization, including NMR, XPS, and ICP-AES measurements, we revise the palladium binding modes of many Pd(II)-incorporated imine-linked COF materials: the Pd(II) centers are not simply adsorbed in the interlayers of two-dimensional COFs; instead, they form palladacycle species through a facile C–H cleavage within the same COF layer (Figure R1). These findings are extremely essential for the future development of heterogeneous transition-metal catalysis.

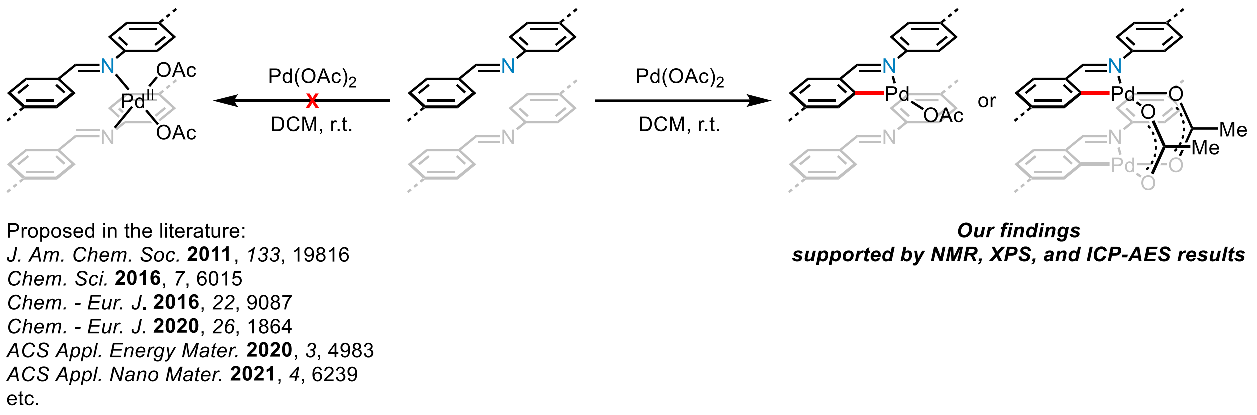

Figure R1. Reassignment of heterogenized palladium species supported by imine-linked COFs.

While we highlighted this key point in the introduction, we fully agree that the equation shown in previous Scheme 1c was not clear enough. As a result, we provided more structural reassignment information in the current version:

"Notably, because Pd(II)-mediated C–H cleavage in benzaldehyde-derived imines is favored both kinetically and thermodynamically, the post-synthetic palladation of imine-linked COF materials produces supported imine-containing palladacycle catalysts (Scheme 1c), rather than heterogenized palladium species located in the COF interlayers, as supported by a series of characterization data."

Scheme 1. Development of Catalytically Active Palladacycles in COF Supports with Nitrogen-Based Linkages

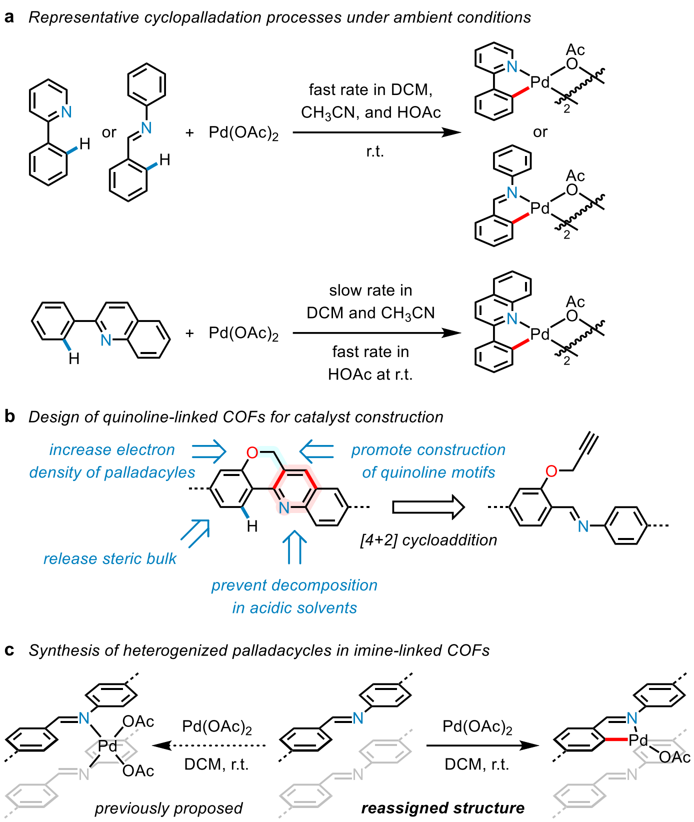

(2) Figure 6d suggests that the catalytic efficiency of Pd(OAc)2 is significantly reduced after 2-3 hours reaction time. How does this compare to the originally reported arylation of 2-methyl indole by Sanford (Ref. 22), which is reported to give good yields even after 5 min reaction time? The COF material requires 48 hours reaction time instead. This should be discussed by the authors. Sanford and co-workers were using acetic acid as additive in their reaction, while in the present submission, acetic acid is not required. Can the authors comment on this?

Response: We thank the reviewer for the important concern. Due to the mass transfer in the pores of COF supports, the heterogenized palladacycle catalysts gave slower rates compared to their homogeneous counterparts. However, the COF-supported palladacycles are significantly more stable than $Pd(OAc)_2$, which readily forms palladium nanoparticles in both DCE and HOAc (Figures 6c and S52). According to Professor Sanford's original paper, when HOAc was used as solvent, $Pd(OAc)_2$ deactivated even within 5 min, despite the fact that the yield could reach 49%. In the third paragraph of *JACS* **2006**, *128*, 4972:

"We were pleased to discover that the desired 2-phenylated product **1a** was obtained in 49% yield, under extremely mild conditions—within 5 min at room temperature. … Interestingly, starting material **1** was not completely consumed under these conditions (with $Pd(OAc)_2$ as the catalyst); however, the use of longer reaction times and/or elevated temperatures did not afford increased yields, suggesting that catalyst deactivation might be occurring in this system."

Basically, without tuning external ligands or employing suitable framework supports, the product yields are difficult to improve.

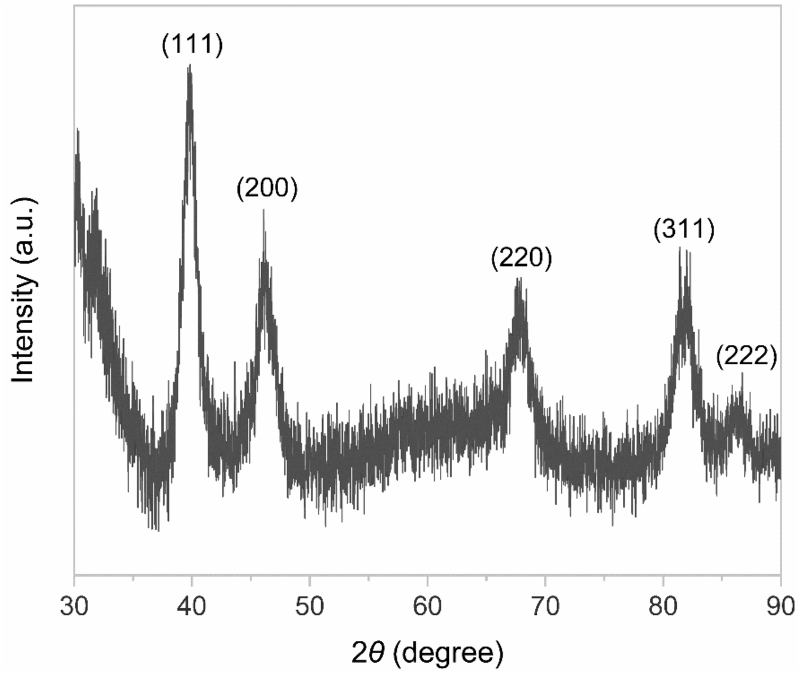

Figure S52. PXRD patterns of recycled palladium species in homogenous catalysis with $Pd(OAc)_2$ in acetic acid.

In response to the reviewer's great suggestion, we included the following discussion in the manuscript:

"The reaction with $Pd(OAc)_2$ slowed down considerably after one hour, and the catalyst became completely inactive after being subjected to the arylation conditions for three hours. A similar deactivation scenario was also observed in the previous homogeneous catalysis (Figure S52), which utilized acetic acid as an optimal solvent to achieve a relatively higher initial rate.[22]"

In contrast to the homogeneous catalytic system developed by Professor Sanford, acetic acid is not the optimal solvent or additive in the heterogeneous catalysis for C–H arylation. Please see the results in Table S5, entry 2 and Table S7, entry 9.

Table S5. Effect of Solvent in the C2-Selective C–H Arylation of *N*-methyl Indole 1a with Diphenyliodonium Tetrafluoroborate 2a

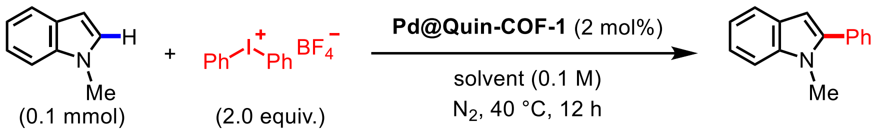

Entry	Solvent (1 mL)	Yield of **3a** (%)[a]

1	DCM	48
2	AcOH	19
3	toluene	11
4	acetonitrile	7
5	hexanes	<5
6	anhydrous THF	<5
7	DMF	<5
8	DMSO	<5
9	dioxane	<5
10	anhydrous DCE	66
11[b]	anhydrous DCE	54

[a]Yield was determined by ^{1}H NMR of the crude product using CH_2Br_2 as an internal standard. [b]In air.

Table S7. Optimization of the C2-Selective C–H Arylation using Pd@Quin-COF-1 as the Heterogeneous Catalyst

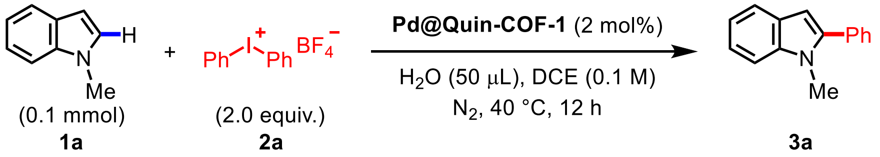

Entry	Change from “Standard Conditions”	Yield of **3a** (%)[a]
1	none	97
2	r.t., instead of 40 °C	74
3	6 h	79
4	2 h	43

5	1 h	33
6	**Pd@Quin-COF-1** (1 mol%)	59
7	**2a** (1.5 equiv.)	69
8	**2a** (1.0 equiv.)	55
9	AcOH (50 µL), instead of H_2O	66
10	under an atmosphere of air, instead of N_2	76

[a]Yield was determined by ^{1}H NMR of the crude product using CH_2Br_2 as an internal standard.

(3) The 2-arylation reaction of indole is a very well established transformation and numerous methods exist for this purpose. Data on the reusability of the catalyst and its catalytic performance is convincing, yet the transformation is from an organic synthesis perspective not truly exciting. This is – from an organic chemists' perspective – an innovative new catalyst, the potential of it, is however not fully considered by the authors. I would have expected more in-depth studies, e.g. in other Pd-catalyzed transformations to show the generality of this COF material in a more general sense. At present, the synthesis component is in my opinion a weak part of this submission, which should be significantly improved, e.g. are there transformations exclusive to this COF material? At present, the combination of this new material with a rather modest advancement in catalysis/synthesis is more suitable for publication in a more specialized journal.

Response: We appreciate the reviewer's very important concerns. While the vast majority of heterogeneous catalytic organic transformations have already been well established in the corresponding homogeneous systems, developing a suitable framework support for C–H arylation is not trivial. None of the prior homogeneous or heterogeneous catalytic systems can prevent Pd(II) from generating inactive Pd(0) nanoparticles, regardless of the organic ligands or MOF/carbon materials used. Therefore, it is of significance to design novel COF materials to support palladacycle species for sustainable C–H activation with high catalyst recyclability. We believe the achievement in our current work is an important step toward making C–H activation methodologies practical.

Taking advantage of the great thermal stability of **Pd@Quin-COF-1**, we developed two additional types of C–H activation reactions with 2-phenylpyridine as the substrate. The results have been included in the revised manuscript and the SI.

"The exceptionally low palladium catalyst loadings underline the importance of the heterogenization strategy for the future development of C–H activation reactions. To demonstrate its broad applicability, we employed **Pd@Quin-COF-1** to achieve C–H bromination and acetoxylation of 2-phenylpyridine in synthetically useful yields (see the Supporting Information for details). The PXRD patterns were well maintained after the catalytic reactions (Figure S68)."

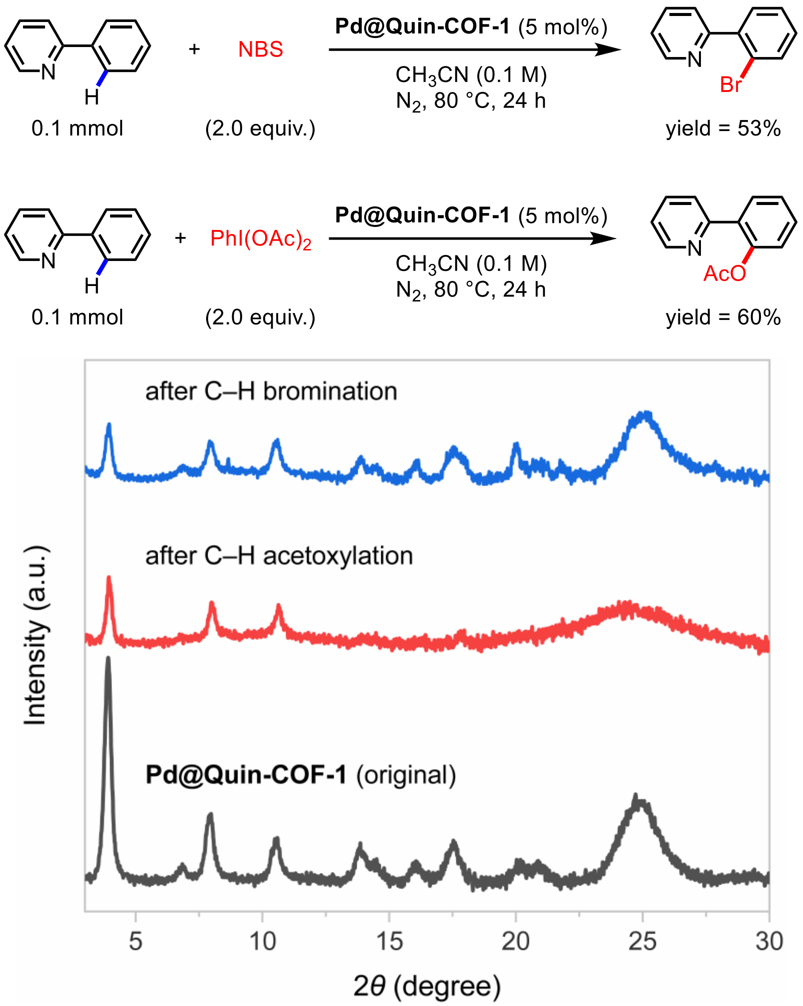

Figure S68. PXRD patterns of **Pd@Quin-COF-1** recycled from C–H bromination and acetoxylation.

(4) The authors state that crystallinity remained constant after 5-runs as shown in Figure 6f. However, I perceive significant differences in the PXRD between the original material and the material after 5 runs. Can the authors explain these?

Response: We thank the reviewer for the important concern. Actually, the PXRD data of the recycled **Pd@Quin-COF-1** highly resembled those of the original **Quin-COF-1** displayed in previous Figure 4a. In order to minimize potential confusion, we prepared fresh batches of **Quin-COF-1** and then synthesized **Pd@Quin-COF-1** via post-synthetic metalation. In the revised manuscript, we provided the PXRD patterns after each catalytic run. Figures 4 and 6 were updated accordingly.

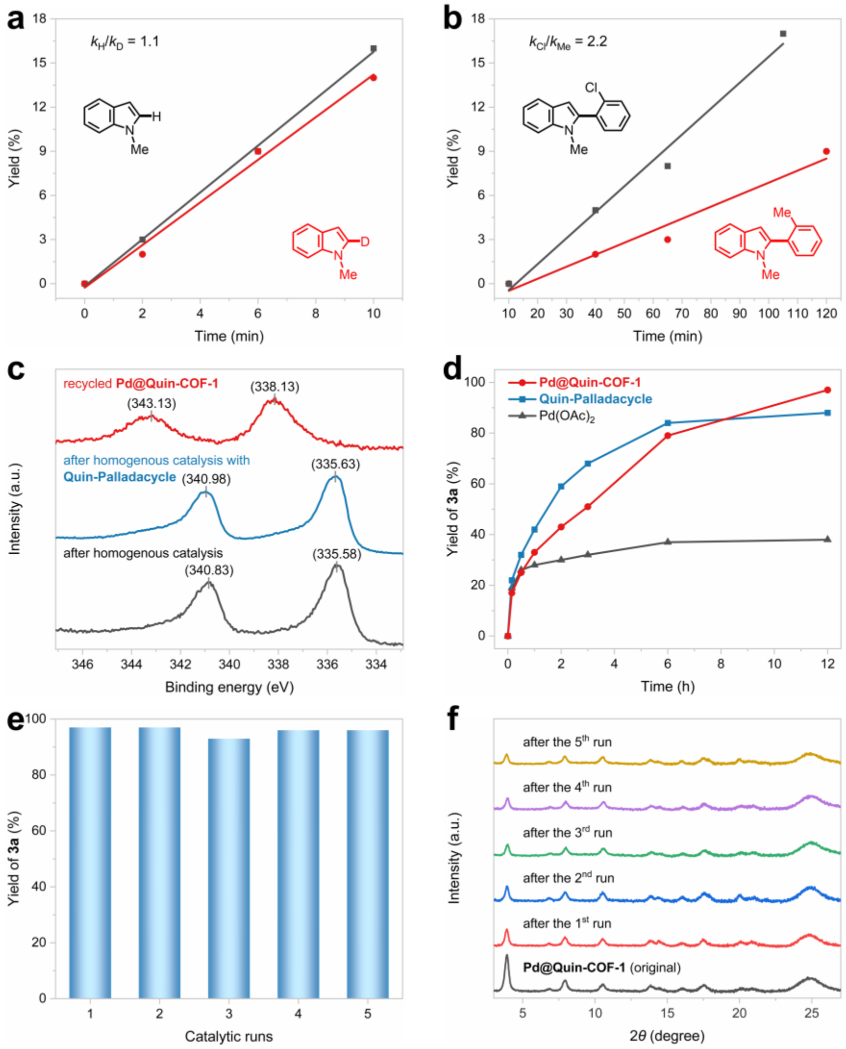

Figure 6. (a) KIE of the heterogeneous palladium-catalyzed C–H arylation. (b) Effects of diaryliodonium salts on the reaction rate. (c) Pd3*d* XPS spectra of **Pd@Quin-COF-1** after 5 catalytic runs and recovered palladium species after the first run of homogeneous catalysis revealing that the Pd centers are in the +2 and 0 oxidation states, respectively. (d) Reaction profiles with the addition of **Pd@Quin-COF-1** (2 mol%), **Quin-Palladacycle** (2 mol%), and $Pd(OAc)_2$ (2 mol%), respectively. (e) Recycling experiments for the synthesis of **3a**. (f) PXRD patterns of **Pd@Quin-COF-1** before and after catalysis.

Reviewer #3

The development of sustainable metal catalysts for value-added transformations via C-H activation is crucial to meet the increasing demands from academia and industry. A case in point is the work by Sun et al., which advances supported imine-containing palladacycle catalysts. These catalysts are constructed through the post-synthetic palladation of various imine-linked covalent organic framework (COF) materials and have been shown to be effective in the C-H arylation of a wide range of indole and pyrrole derivatives with diaryliodonium tetrafluoroborate at C2 sites. This work enhances the capabilities of molecular Pd species and porous crystalline materials, enabling their recyclability and improving their performance in C-H arylation of heterocyclic substrates with diverse electronic properties. This advancement justifies its acceptance for publication in ACS Central Science. However, revisions are needed to address the following concerns:

(1) The significance of catalytic application is not clearly defined due to insufficient background information and the lack of comparison. Therefore, a detailed introduction is necessary to elucidate the current status of C-H arylation of indole and pyrrole derivatives. Additionally, a table summarizing the performance indicators should be included to compare with existing homogeneous/heterogeneous catalysts.

Response: We are very grateful for the reviewer's constructive suggestions. A detailed introduction and a summary table was included in the revised manuscript and SI, respectively:

"With a series of COF-supported palladium catalysts in hand, we began to test our hypothesis using the C–H arylation of *N*-methyl indole (**1a**) with diphenyliodonium tetrafluoroborate (**2a**) as a template reaction.[22] While the corresponding homogeneous palladium catalysis could be promoted through the use of an *N*-heterocyclic carbene ligand, catalyst recycling remains quite difficult to achieve, even with diverse solid matrices. As a result, it is vital to employ the novel heterogenized palladacycles to explore sustainable C–H arylation with high catalyst recyclability."

Table S14. Representative C–H Arylation of Indoles with Homogeneous and Heterogeneous Palladium Catalysts

Entry	Catalyst & Additive	Indole Substrate (R = Me or H)	Counter partner (equiv.)	Solvent	Temp. (°C)	TON (1st Run)	TON (2nd Run)	TON (3rd Run)	Ref.
1	2 mol% **Pd@Quin-COF-1**	Me	Ph_2IBF_4 (2.0)	DCE/ H_2O	40	48.5	48.5	46.5	This work
2	2 mol% **Pd@Im-COF-2**	H	Ph_2IBF_4 (2.0)	DCE/ H_2O	40	37	38.5	37	This work
3	5 mol% IMesPd(OAc)$_2$	Me	Ph_2IBF_4 (2.0)	AcOH	25	17.2	N.A.	N.A.	22
4	5 mol% IMesPd(OAc)$_2$	H	Ph_2IBF_4 (2.0)	AcOH	25	16.2	N.A.	N.A.	22
5	2.5 mol% Pd^0-AmP-MCF	Me	Ph_2IBF_4 (2.0)	H_2O	25	32			23

6	2.5 mol% Pd^0-AmP-MCF	H	Ph_2IBF_4 (2.0)	H_2O	25	36.4	32[a]	26.8[a]	23
7	5 mol% Pd/C	H	Ph_2IBF_4 (1.4)	EtOH	60	8			24
8	10 mol% Pd/C, Polarclean	Me	Ph_2IBF_4 (1.25)	H_2O	70	9.2			25
9	10 mol% Pd/C, Polarclean	H	Ph_2IBF_4 (1.25)	H_2O	70	9.3	9.3	9.3	25
10	5 mol% $Pd(OAc)_2$, Ag_2O (0.75 equiv.) *o*-$NO_2C_6H_4CO_2H$ (1.5 equiv.)	Me	PhI (2.0)	DMF	25	18.4	N.A.	N.A.	26
11	5 mol% $Pd(OAc)_2$, Ag_2O (0.75 equiv.) *o*-$NO_2C_6H_4CO_2H$ (1.5 equiv.)	H	PhI (2.0)	DMF	50	12.2	N.A.	N.A.	26

[a]TON values was calculated based on ^{1}H NMR yields rather than isolated yields.

22. Deprez, N. R.; Kalyani, D.; Krause, A.; Sanford, M. S. Room Temperature Palladium-Catalyzed 2-Arylation of Indoles. *J. Am. Chem. Soc.* **2006**, *128*, 4972–4973.
23. Malmgren, J.; Nagendiran, A.; Tai, C.-K.; Bäckvall, J.-E.; Olofsson, B. C-2 Selective Arylation of Indoles with Heterogeneous Nanopalladium and Diaryliodonium Salts. *Chem. - Eur. J.* **2014**, *20*, 13531–13535.
24. Tang, D. -T. D.; Collins, K. D.; Ernst, J. B.; Glorius, F. Pd/C as a Catalyst for Completely Regioselective C–H Functionalization of Thiophenes under Mild Conditions. *Angew. Chem., Int. Ed.* **2014**, *53*, 1809–1813.
25. Campana, F.; Massaccesi, B. M.; Santoro, S.; Piermatti, O.; Vaccaro, L. Polarclean/Water as a Safe and Recoverable Medium for Selective C2-Arylation of Indoles Catalyzed by Pd/C. *ACS Sustainable Chem. Eng.* **2020**, *8*, 16441–16450.
26. Lebrasseur, N.; Larrosa, I. Room Temperature and Phosphine Free Palladium Catalyzed Direct C-2 Arylation of Indoles. *J. Am. Chem. Soc.* **2008**, *130*, 2926–2927.

(2) Full-scale XPS spectra should be provided following standard discipline.

Response: We thank the reviewer for the valuable suggestion. We added the full-scale XPS spectra of the COF materials in the SI.

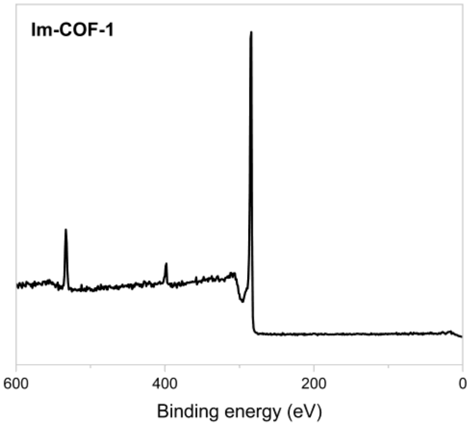

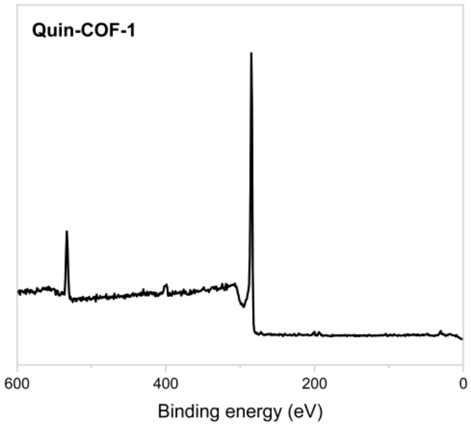

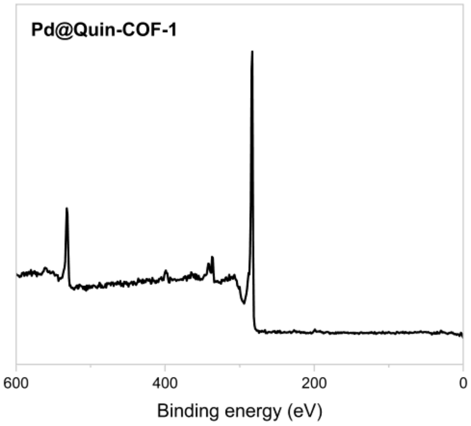

Figure S14. Full-scale XPS spectra of **Im-COF-1**, **Quin-COF-1**, and **Pd@Quin-COF-1**.

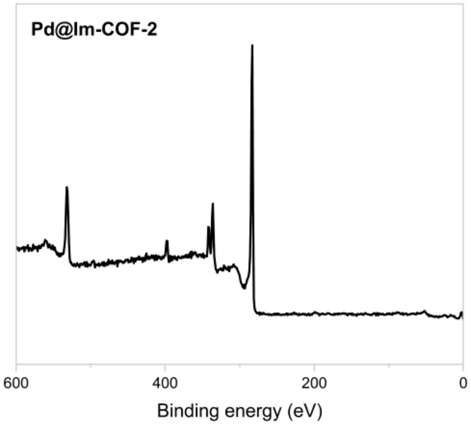

Figure S16. Full-scale XPS spectrum of **Pd@Im-COF-2**.

(3) The study on diverse porous supports lacks conviction without providing any characterization of them. Basic measurements, such as PXRD patterns of different Pd(II)-incorporated COF materials, are suggested to be included.

Response: We thank the reviewer for the valuable suggestion. We measured all the PXRD patterns of COF materials before and after post-synthetic metalation and included them in the SI:

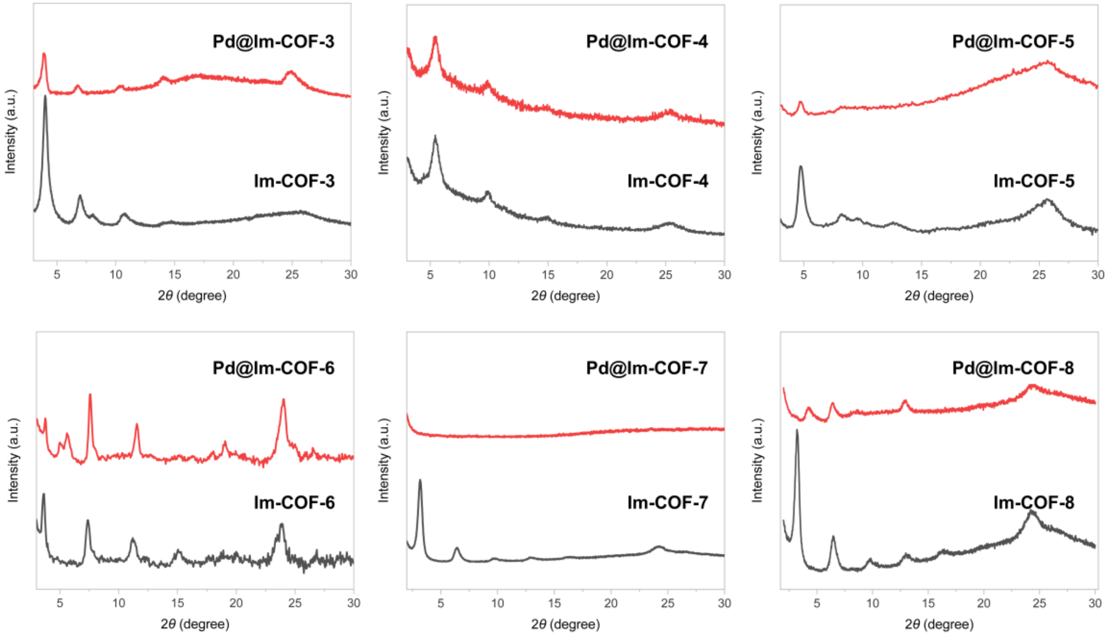

Figure S11. PXRD patterns of **Im-COF-3** to **Im-COF-8** before and after metalation with $Pd(OAc)_2$.

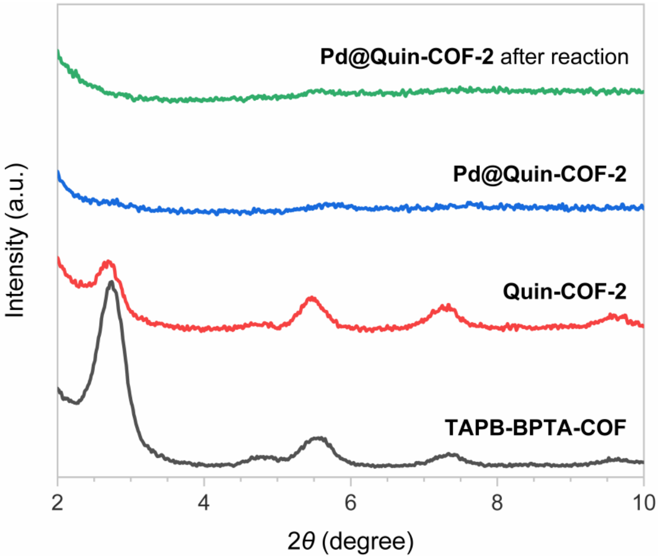

Figure S53. PXRD patterns of COF materials related to **Pd@Quin-COF-2**.

(4) There appears to be evidence of structural collapse during post-modification, as indicated by the drastic decrease in BET surface area and the emergence of a pore size at around 5 nm. This point requires further explanation.

Response: We thank the reviewer for the important concern. We believe the structural collapse occurred during the BET measurements, rather than resulting from post-synthetic modification. The PXRD patterns of **Pd@Quin-COF-1** remained consistent with those of **Quin-COF-1**. However, following the BET measurements, the same sample completely lost crystallinity (Figure R2), which explains the emergence of a pore at around 5 nm. We included the following information in the legends.

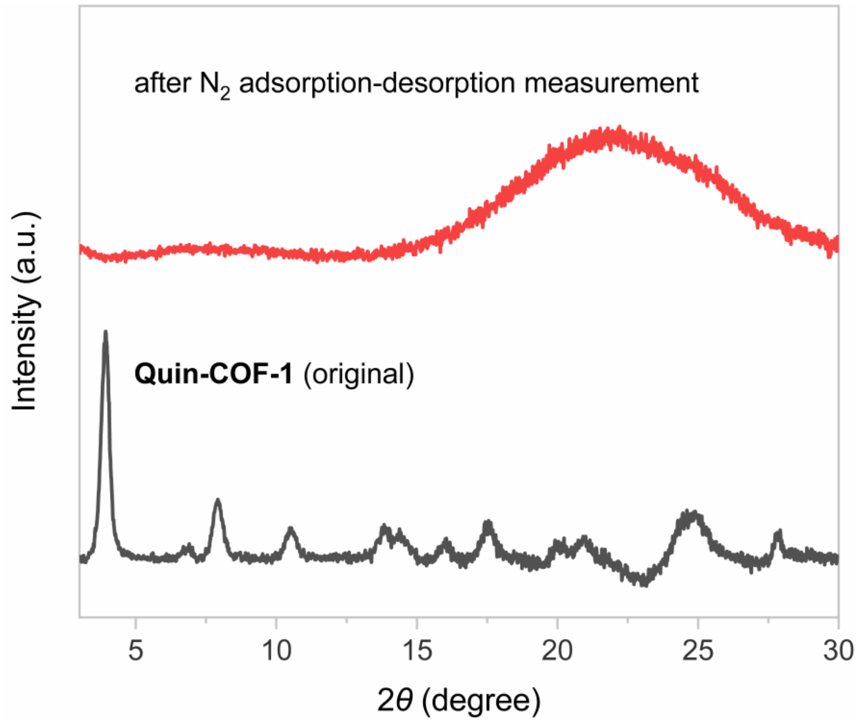

Figure R2. PXRD patterns of **Pd@Quin-COF-1** before and after N_2 adsorption/desorption isotherm measurements.

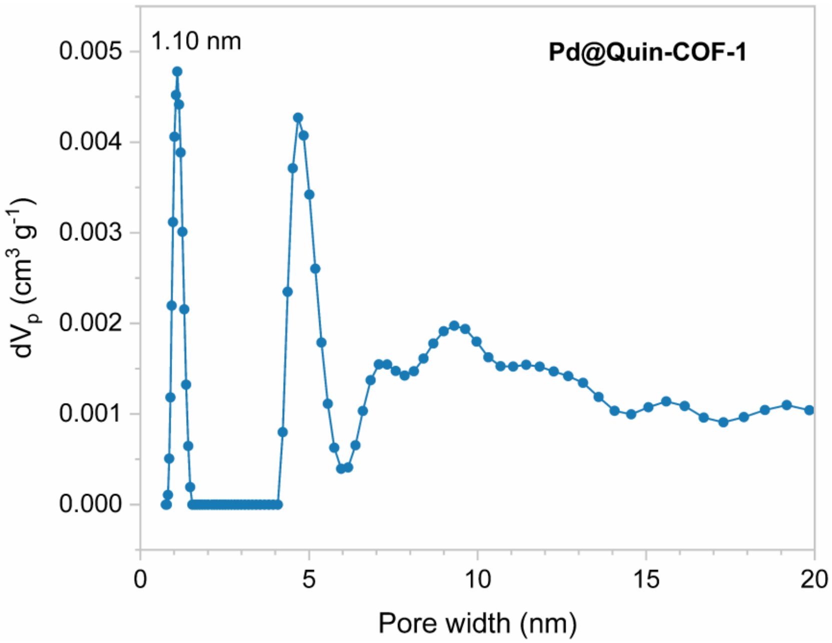

Figure S44. Pore size distribution profiles of **Pd@Quin-COF-1**. The pore at around 5 nm could be generated by **Pd@Quin-COF-1**'s partial structural collapse during N_2 adsorption/desorption isotherm measurements.

(5) This work focuses on sp2 C-H activation, while sp3 C-H activation is a more challenging task. It would be valuable to know whether the newly established catalysts are active toward sp3 C-H activation. Besides, the performance of COF materials incorporated with other molecular Pd species, aside from Pd(OAc)2, should be investigated further.

Response: We thank the reviewer for the constructive suggestions. As sp^3 C–H activation typically requires a high temperature (100–150 °C), the newly synthesized COF matrices are still not quite tolerant of these harsh conditions. Therefore, we are currently investigating photoinduced processes via heterogeneous palladium catalysis for the development of sp^3 C–H activation under ambient conditions. Despite the difficulties of establishing sp^3 C–H activation reactions under thermal conditions, we explored other types of sp^2 C–H activation reactions to further demonstrate the robustness of heterogenized palladacycle catalysts:

"The exceptionally low palladium catalyst loadings underline the importance of the heterogenization strategy for the future development of C–H activation reactions. To demonstrate its broad applicability, we employed **Pd@Quin-COF-1** to achieve C–H bromination and acetoxylation of 2-phenylpyridine in synthetically useful yields (see the Supporting Information for details). The PXRD patterns were well maintained after the catalytic reactions (Figure S68)."

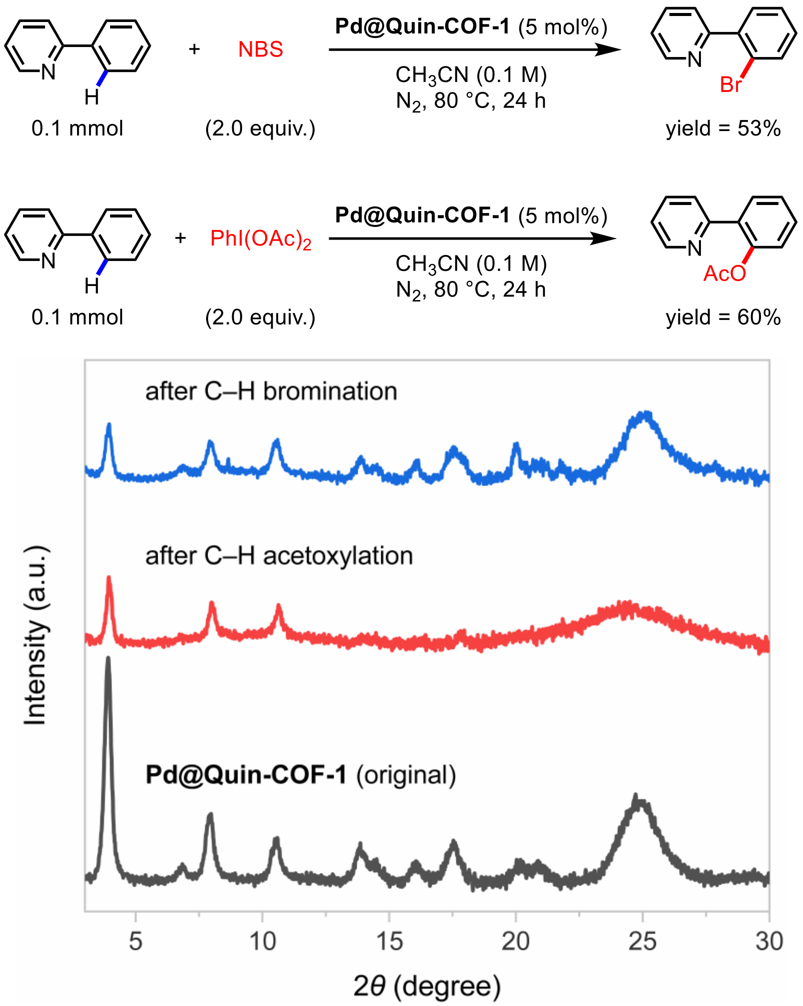

Figure S68. PXRD patterns of **Pd@Quin-COF-1** recycled from C–H bromination and acetoxylation.

Following the reviewer's excellent idea, we also prepared several **Quin-COF-1**-based palladium catalysts and tested their reactivity in the C–H arylation of *N*-methyl indole. The results were included in the updated SI:

General procedure for the synthesis of COF-supported catalysts using different palladium salts: The palladium salt was dissolved in DCM (5 mL) to prepare a saturated solution. Upon removal of the undissolved solid, **Quin-COF-1** (25 mg) was added. The mixture was stirred slowly at 60 °C for 48 h. After reaction completion, the resulting solid was isolated by filtration and washed with DCM (3 × 50 mL), then dried at 70 °C for 12 h to yield the corresponding COF-supported palladium catalyst.

Table S12. Post-Synthetic Metalation of Quin-COF-1 with Different Palladium Salts

Entry	PdX_n@Quin-COF-1	Color	Pd Loading (wt%)[a]	Pd/N Ratio (experimental data)	Pd/N Ratio (theoretical data)
1	**$Pd(TFA)_2$@Quin-COF-1**	brown	7.2	1:4.3	1:1
2	**$PdCl_2$@Quin-COF-1**	dark red	1.3	1:30	1:1
3	**$PdBr_2$@Quin-COF-1**	dark red	0.1	1:290	1:1
4	**K_2PdCl_4@Quin-COF-1**	dark red	0.2	1:220	1:1

[a]Loading was determined by ICP-AES.

Table S13. Effect of Palladium Salts in Heterogeneous Catalysis for C–H Arylation

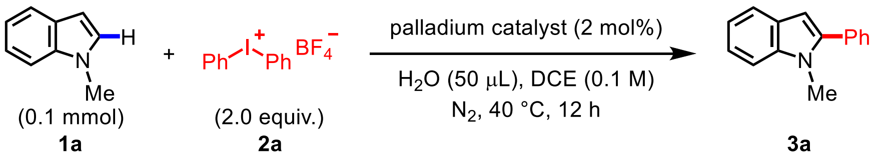

Entry	Catalyst	1st run yield (%)[a]	2nd run yield (%)[a]
1	**Pd@Quin-COF-1**	97	97
2	**$Pd(TFA)_2$@Quin-COF-1**	54	57
3	**$PdCl_2$@Quin-COF-1**	85	69

[a]Yield was determined by ^1H NMR of the crude product using CH_2Br_2 as an internal standard.

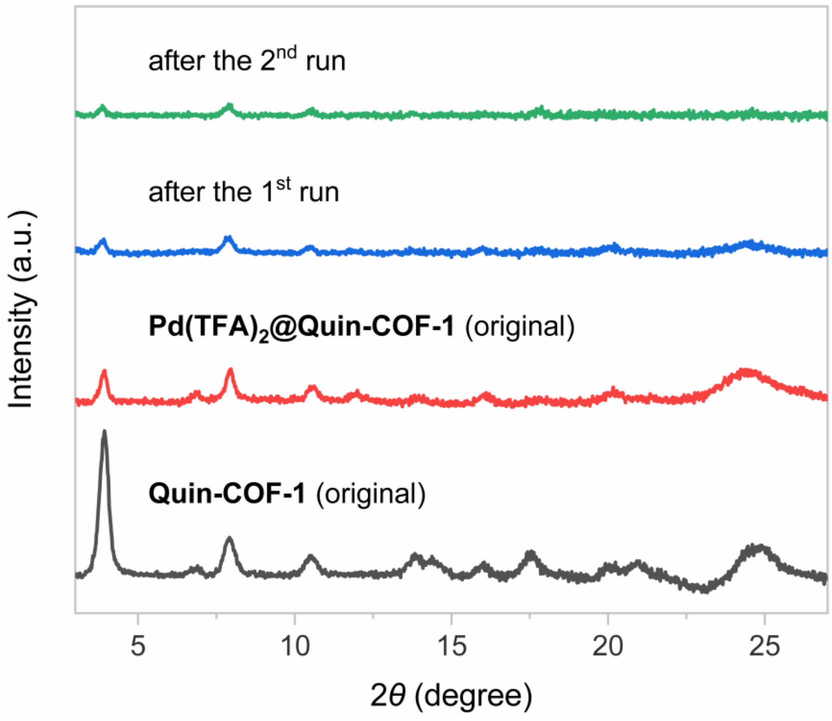

Figure S55. PXRD patterns of **$Pd(TFA)_2$@Quin-COF-1** before and after reaction.

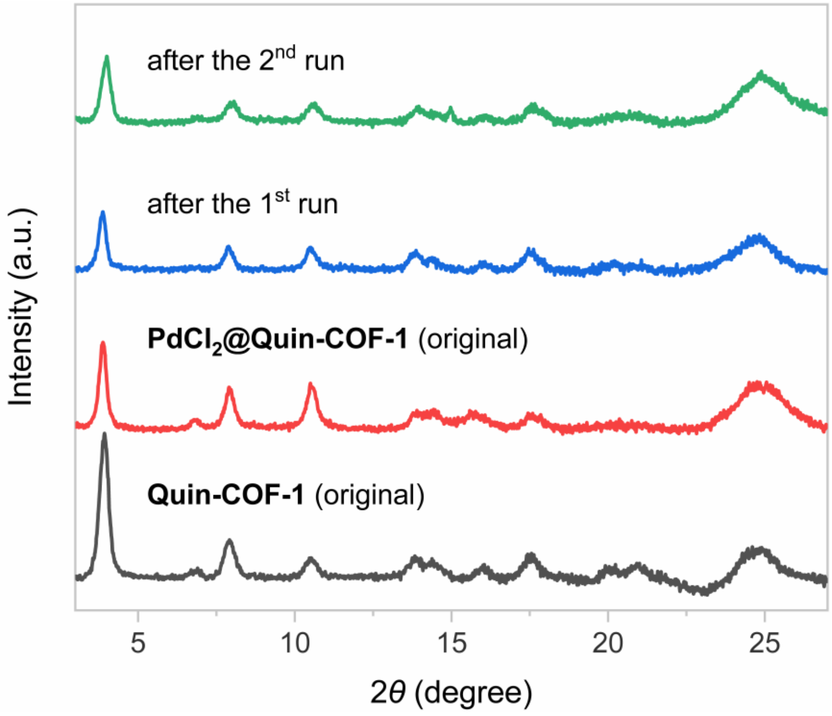

Figure S56. PXRD patterns of **$PdCl_2$@Quin-COF-1** before and after reaction.

oc-2024-006604.R2

Name: Peer Review Information for "Structural Reassignment of Covalent Organic Framework-Supported Palladium Species: Heterogenized Palladacycles as Efficient Catalysts for Sustainable C–H Activation"

Second Round of Reviewer Comments

Reviewer: 1

Comments to the Author
The authors satisfactorily responded my commends and revised the work accordingly. I recommend acceptance in its present form.

Reviewer: 3

Comments to the Author
The authors have satisfactorily addressed all the comments from the reviewer and the manuscript can now be accepted in its current form.

Reviewer: 4

Comments to the Author
The authors synthesized porous covalent organic frameworks (COF) in this work. Pd is embedded in the COF through N and C-ligands for indole and pyrrole derivatives' C–H arylation reactions. Incorporating N and C-ligand-chelated palladacycles into covalent organic frameworks (COFs) is an interesting approach. The authors have done various characterizations to study the formation of COF and Pd chelation. However, multiple concerns have to be addressed before recommending for publication.
Comment to the authors.
1. How does this method compare to other strategies aimed at improving the stability and recyclability of palladium catalysts? What are the unique advantages of using COFs over other support materials? This should be discussed and highlighted in the introduction.
2. The reactions performed only with indole and pyrrole derivatives. Are there any limitations in terms of substrate scope for this methodology? How generalizable is this approach to other types of C–H activation reactions and substrates?
3. The PXRD patterns show a prominent peak at 3.92° and several relatively weak peaks between 6° and 11°. What are those peaks, and how do they relate to the crystallinity of the COF materials?
4. There is an apparent shift (> 3 eV) in the Pd 3d xps after the recycle reaction with Pd@Quin-COF-1, why does it happen?
5. The characteristic peak intensity of COF at a low angle (<5°) in PXRD decreases after each recycle test, which means the crystallinity of COF decreases.
6. The BET studies should be provided after the recycling test to confirm that COF possesses pores.
7. As per the authors, no Pd leaching was detected through ICP-AES measurements. then how did the reaction rate decrease during the recycling test?
8. The deactivation of Pd(OAc)2 after 3 h contrasts with the sustained activity of Pd@Quin-COF-1. What are the reasons for the rapid deactivation of Pd(OAc)2?

9. The Quin-Palladacycle achieved the highest rate in the first two hours, while Pd(OAc)2 slowed down considerably after one hour. What factors might contribute to the performance of Quin-Palladacycle compared to Pd(OAc)2?
10. The manuscript needs to be carefully checked for typos.

Author's Response to Peer Review Comments:

THE UNIVERSITY OF HONG KONG

Department of Chemistry and State Key Laboratory of Synthetic Chemistry
Room 103, Hui Oi Chow Science Building, Pokfulam Road, Hong Kong

Jian He, Ph.D.
Assistant Professor
Email: jianhe@hku.hk
Phone: (852) 3910 2193
Fax: (852) 2857 1586

August 14, 2024

We sincerely appreciate the reviewers' time and efforts in helping us improve the quality of our work. We conducted additional experiments and revised the manuscript in response to their constructive feedback and suggestions.

Reviewer #1

The authors satisfactorily responded my commends and revised the work accordingly. I recommend acceptance in its present form.

Response: We truly value the reviewer's positive feedbacks and prior constructive suggestions.

Reviewer #3

The authors have satisfactorily addressed all the comments from the reviewer and the manuscript can now be accepted in its current form.

Response: We truly value the reviewer's positive feedbacks and prior constructive suggestions.

Reviewer #4

The authors synthesized porous covalent organic frameworks (COF) in this work. Pd is embedded in the COF through N and C-ligands for indole and pyrrole derivatives' C–H arylation reactions. Incorporating N and C-ligand-chelated palladacycles into covalent organic frameworks (COFs) is an interesting approach. The authors have done various characterizations to study the formation of COF and Pd chelation. However, multiple concerns have to be addressed before recommending for publication.

(1) How does this method compare to other strategies aimed at improving the stability and recyclability of palladium catalysts? What are the unique advantages of using COFs over other support materials? This should be discussed and highlighted in the introduction.

Response: We are very grateful for the reviewer's questions and important suggestions. The current method provides the first example of employing in-situ generated palladacycle complexes to promote heterogeneous catalysis for C–H activation. Since the COF linkers were utilized to form electron-rich LX-type chelating ligands for palladacycle synthesis, the palladium catalysts become more stable and difficult to get removed from the COF supports. To help readers better understand how support materials affect catalyst stability and recyclability, we included the following dicussion in the introduction:

"We envisioned that the construction of nitrogen-donor ligand motifs in the rigid linkers of porous crystalline covalent organic frameworks (COFs)[8] and the subsequent introduction of a chelating metal coordination mode through post-synthetic cyclometalation would fundamentally address the aforementioned issues. Compared to metal–organic framework materials,[9] which are constructed through coordination interactions between organic linkers and metal nodes, the COF supports typically exhibit superior chemical stability, including water stability. In addition, it is anticipated that other commonly used heterogeneous catalysts, such as palladium on charcoal, are unable to prevent palladium leaching due to a lack of suitable ligands to stabilize Pd(II). Therefore, we primarily focused on the modification of COF materials to explore heterogeneous palladium-catalyzed C–H activation."

(2) The reactions performed only with indole and pyrrole derivatives. Are there any limitations in terms of substrate scope for this methodology? How generalizable is this approach to other types of C–H activation reactions and substrates?

Response: We thank the reviewer for the valuable questions. Similar to other heterogeneous catalytic systems, this methodology is somewhat sensitive to steric hindrance of indole substrates, but the scope of diaryliodonium salts is quite broad. In addition, we demonstrated the generalizability of the current approach by developing pyridine-directed C–H bromination and acetoxylation reactions. We have added the corresponding discussion in the manuscript:

"In contrast to homogeneous catalytic systems, the arylation reaction is more susceptible to the steric hindrance at the 3-position of indoles (**5h**). When the methyl group was replaced with a larger substituent in *N*-acetyl-tryptophan methyl ester, the heterogeneous palladium catalysis became less effective, revealing size selectivity in COF-based reaction systems (Table S11)."

"To demonstrate its broad applicability, we employed **Pd@Quin-COF-1** to achieve C–H bromination and acetoxylation of 2-phenylpyridine in synthetically useful yields (see the Supporting Information for details), showcasing the potentials of COF-supported palladacycles in catalyzing directed C–H activation/C–heteroatom bond forming reactions. The PXRD patterns were well maintained after the catalytic reactions (Figure S70)."

(3) The PXRD patterns show a prominent peak at 3.92° and several relatively weak peaks between 6° and 11°. What are those peaks, and how do they relate to the crystallinity of the COF materials?

Response: We thank the reviewer for the important questions. In PXRD patterns, the strength and width of the peak indicate the degree of crystallinity and the size of the crystal grains. We have updated the manuscript with the peak assignments and the related discussion of crystallinity:

“As depicted in Figure 2, the PXRD patterns feature a prominent peak at 3.92° and three relatively weak peaks between 6° and 11°, which correspond to the (100), (110), (200), and (210) reflection planes, respectively.[11b] The PXRD data revealed that the imine- and quinoline-linked COFs both had good crystallinity and reasonably large crystal grain sizes, with narrow full width at half-maximum values for the sharp (100) peaks.”

(4) There is an apparent shift (> 3 eV) in the Pd 3d xps after the recycle reaction with Pd@Quin-COF-1, why does it happen?

Response: We thank the reviewer for the important concern. The previous Figure 6c was a bit confusing; the XPS shift of > 3 eV came from filtered solids after homogeneous catalysis, not recycled **Pd@Quin-COF-1**. This evident discrepancy suggests that soluble palladium catalysts will eventually decompose into palladium nanoparticles. To avoid any confusion to readers, we have updated Figure 6c by adding the XPS spectrum of original **Pd@Quin-COF-1**.

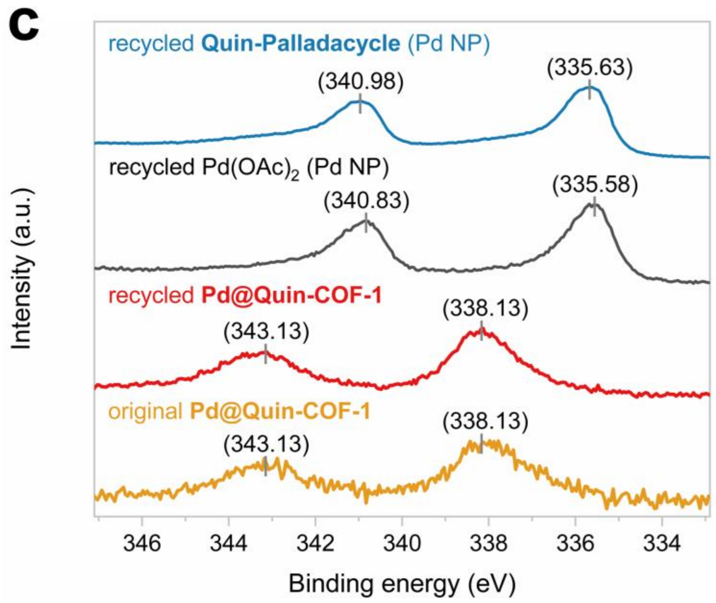

Figure 6c. Pd3*d* XPS spectra of **Pd@Quin-COF-1** after 5 catalytic runs (red line) and recovered palladium species after the first run of homogeneous catalysis (black and blue lines) revealing that the Pd centers are in the +2 and 0 oxidation states, respectively.

(5) The characteristic peak intensity of COF at a low angle (<5°) in PXRD decreases after each recycle test, which means the crystallinity of COF decreases.

Response: We appreciate the reviewer pointing out this issue. After the first recycling test, there was a moderate decrease in intensity. This is because the COF was not post-treated after recycling, but rather filtered and recovered, thus there was some solvent in the pores, which influenced the PXRD intensity. The subsequent cycles showed no noticeable decrease, indicating the catalyst’s high stability in the C–H arylation. We have included the following discussion in the manuscript:

"The catalytic activity of the COF-supported palladacycle remained constant after a 5-run recycling experiment, and the sharp reflection peaks were retained in the PXRD patterns, albeit a minor decrease in intensity (Figures 6e and 6f). Nevertheless, the COF support displayed nearly identical pores after the first run of heterogeneous catalysis (Figures S63 and S64)."

(6) The BET studies should be provided after the recycling test to confirm that COF possesses pores.

Response: We thank the reviewer for the constructive suggestion. Accordingly, we carried out the BET studies of the recycled catalyst following the C–H arylation of the template substrate. The surface area and pore size data are available now in the SI:

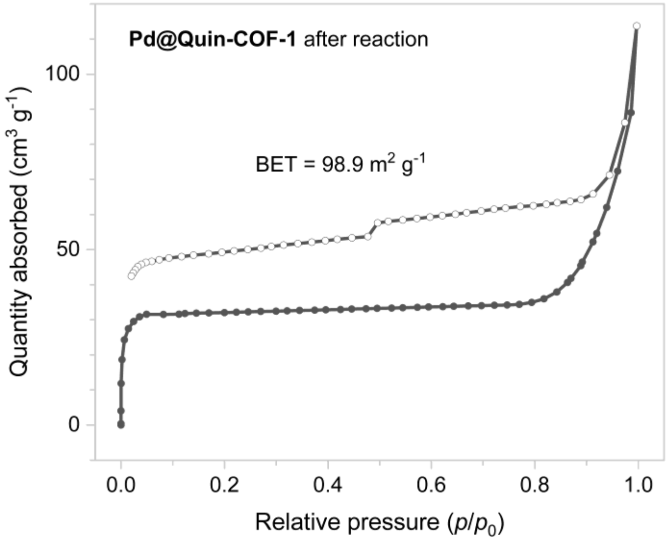

Figure S63. N_2 adsorption/desorption isotherms of **Pd@Quin-COF-1** after reaction recorded at 77 K.

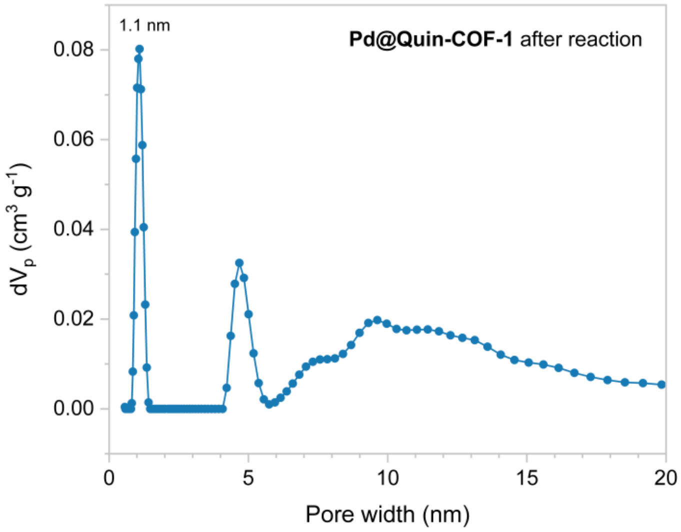

Figure S64. Pore size distribution profiles of **Pd@Quin-COF-1** after reaction.

(7) As per the authors, no Pd leaching was detected through ICP-AES measurements. then how did the reaction rate decrease during the recycling test?

Response: We appreciate the reviewer's important concern. The reactions in the recycling test were performed under the identical conditions, and the rates were comparable in each run. Within the same experiment, the reaction rate decreased as the starting materials were consumed.

(8) The deactivation of Pd(OAc)2 after 3 h contrasts with the sustained activity of Pd@Quin-COF-1. What are the reasons for the rapid deactivation of Pd(OAc)2?

Response: We thank the reviewer for this fundamental question in palladium catalysis. Inactive Pd(0) species are frequently formed during C–H activation, as substrates and additives can act as reductants, transferring electrons to Pd(II) (Figure R1). Therefore, the inclusion of suitable ligands is critical for achieving high turnover numbers and catalyst stability in the majority of palladium-catalyzed C–H activation reactions.

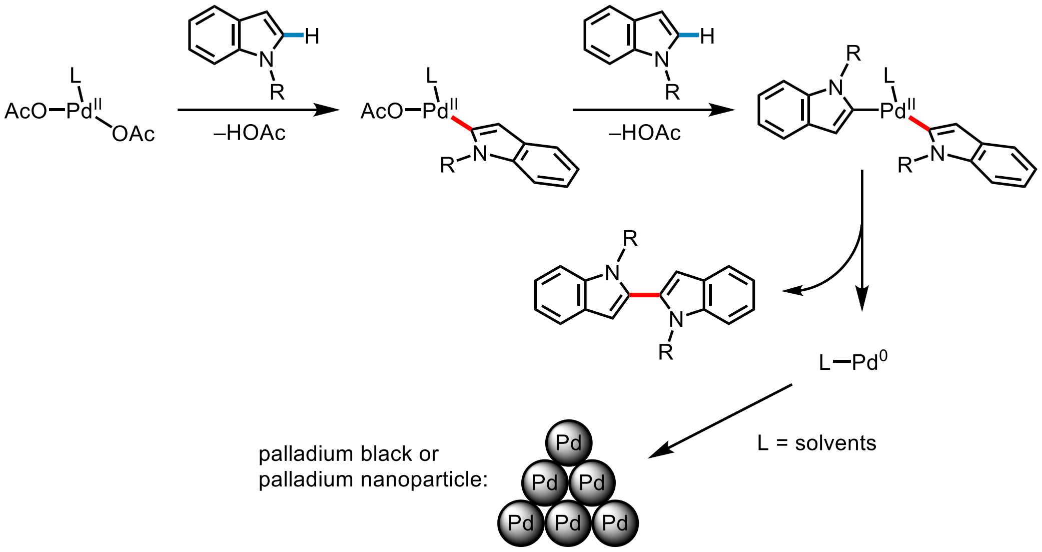

Figure R1. Plausible mechanism for the formation of inactive Pd(0) species.

"The reaction with $Pd(OAc)_2$ slowed down considerably after one hour, and the catalyst completely lost its activity after being subjected to the arylation conditions for three hours, owing to the inevitable and rapid formation of palladium nanoparticles (Figure 6c)."

(9) The Quin-Palladacycle achieved the highest rate in the first two hours, while Pd(OAc)2 slowed down considerably after one hour. What factors might contribute to the performance of Quin-Palladacycle compared to Pd(OAc)2?

Response: We thank the reviewer for the important question. According to the mechanistic studies, the heterogeneous catalysis most likely involves a Pd(II)/Pd(IV) redox manifold, and the formation of the highly reactive Pd(IV) intermediate is the rate-determining step (Scheme 2). The use of electron-rich palladacycle is beneficial to this key step. Moreover, the chelating ligand in the

palladacycle slows the process of Pd(0) formation by imposing a significantly larger enthalpy penalty (Figure R2). We have added a detailed discussion to the manuscript.

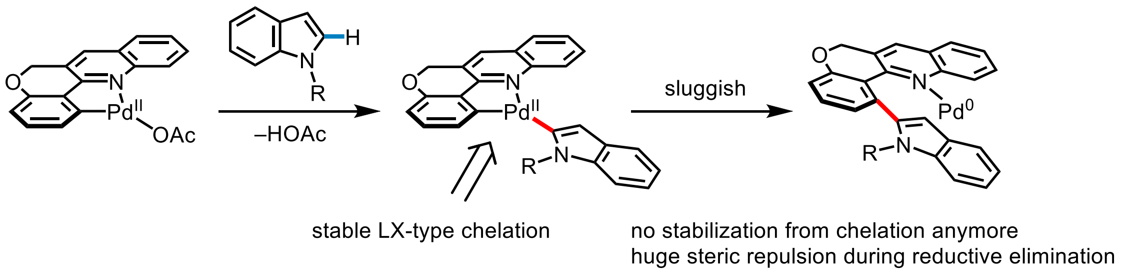

Figure R2. A schematic representation of how the palladacycle slows down the previous process.

"**Quin-Palladacycle** with a strong ligand chelation effect achieved the highest rate in the first two hours, revealing its remarkable catalytic performance in the non-directed C–H activation by stabilizing the critical Pd(IV) intermediate and suppressing the generation of inactive Pd(0) species.[28]"

(10) The manuscript needs to be carefully checked for typos.

Response: We appreciate the reviewer's important comment. One typo was found in the manuscript, one in the reference section. They have been corrected in the current version.

"While the alkynyl moiety is responsible for the carbon signals around 78 ppm, the sp^3-hybridized carbon centers adjacent to oxygen give chemical shifts of 56 and 54 ppm, respectively."

"Sun, Q.; Aguila, B.; Perman, J.; Nguyen, N.; Ma, S. Flexibility Matters: Cooperative Active Sites in Covalent Organic Framework and Threaded Ionic Polymer. *J. Am. Chem. Soc.* **2016**, *138*, 15790–15796."
